# BAG5 regulates HSPA8-mediated protein folding required for sperm head-tail coupling apparatus assembly

**DOI:** 10.1038/s44319-024-00112-x

**Published:** 2024-03-07

**Authors:** Shiming Gan, Shumin Zhou, Jinzhe Ma, Mengneng Xiong, Wenjing Xiong, Xu Fan, Kuan Liu, Yiqian Gui, Bei Chen, Beibei Zhang, Xiaoli Wang, Fengli Wang, Zhean Li, Wei Yan, Meisheng Ma, Shuiqiao Yuan

**Affiliations:** 1 Institute of Reproductive Health, Tongji Medical College, Huazhong University of Science and Technology, Wuhan, 430030 China; 2 Department of Urology & Andrology, Sir Run Run Shaw Hospital, Zhejiang University School of Medicine, Hangzhou, 310016 China; 3 Department of Histology and Embryology, Tongji Medical College, Huazhong University of Science and Technology, Wuhan, 430030 China; 4 Reproductive Medicine Center, Renmin Hospital of Wuhan University, Wuhan, 430060 China; 5 Laboratory of Animal Center, Huazhong University of Science and Technology, Wuhan, 430030 China; 6 The Lundquist Institute for Biomedical Innovation at Harbor-UCLA, Torrance, CA 90502 USA; 7 Cell Architecture Research Center, Huazhong University of Science and Technology, Wuhan, 430030 China

**Keywords:** BAG5, Infertility, Protein-folding, Acephalic sperm, HTCA, Cell Adhesion, Polarity & Cytoskeleton, Molecular Biology of Disease, Translation & Protein Quality

## Abstract

Teratozoospermia is a significant cause of male infertility, but the pathogenic mechanism of acephalic spermatozoa syndrome (ASS), one of the most severe teratozoospermia, remains elusive. We previously reported Spermatogenesis Associated 6 (SPATA6) as the component of the sperm head-tail coupling apparatus (HTCA) required for normal assembly of the sperm head-tail conjunction, but the underlying molecular mechanism has not been explored. Here, we find that the co-chaperone protein BAG5, expressed in step 9-16 spermatids, is essential for sperm HTCA assembly. BAG5-deficient male mice show abnormal assembly of HTCA, leading to ASS and male infertility, phenocopying SPATA6-deficient mice. In vivo and in vitro experiments demonstrate that SPATA6, cargo transport-related myosin proteins (MYO5A and MYL6) and dynein proteins (DYNLT1, DCTN1, and DNAL1) are misfolded upon BAG5 depletion. Mechanistically, we find that BAG5 forms a complex with HSPA8 and promotes the folding of SPATA6 by enhancing HSPA8’s affinity for substrate proteins. Collectively, our findings reveal a novel protein-regulated network in sperm formation in which BAG5 governs the assembly of the HTCA by activating the protein-folding function of HSPA8.

## Synopsis

Abnormal assembly of the head-tail coupling apparatus (HTCA) results in “headless sperm” syndrome. HSPA8-mediated protein folding is required for the assembly of the head-tail coupling apparatus (HTCA) and the integrity of spermatozoa.

- The inactivation of *Bag5* in mice leads to failure in the assembly of the HTCA during spermiogenesis and the production of headless spermatozoa.
- BAG5 serves as a co-chaperone for HSPA8 to modulate protein folding and proteostasis in developing spermatids.
- BAG5 and HSPA8 work in concert to promote the folding of HSPA8 clients such as SPATA6 and other proteins involved in HTCA assembly.

## Abstract (web summary)

Abnormal assembly of the head-tail coupling apparatus (HTCA) results in “headless sperm” syndrome. HSPA8-mediated protein folding is required for the assembly of the head-tail coupling apparatus (HTCA) and the integrity of spermatozoa.

## Graphical abstract

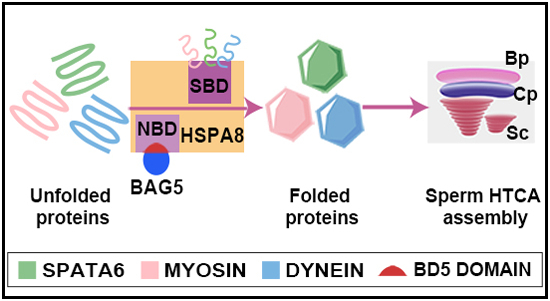

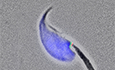

## Introduction

The head-tail coupling apparatus (HTCA) is the tight linkage of sperm head and tail, which is a centrosome-based structure consisting of two cylindrical microtubule-based centrioles and associated components (Wu et al, 2020; Zhang et al, 2021). The failure of HTCA assembly results in the separation of sperm head from sperm tail, rendering acephalic spermatozoa syndrome (ASS) and male infertility (Hetherington et al, 2016; Kim et al, 2018; Wang et al, 2022b; Zhu et al, 2018; Zhu et al, 2016). ASS refers to sperm in the semen with intact flagella but without sperm head (Wang et al, 2023; Yuan et al, 2015). In 2015, we first reported that genetic ablation of SPATA6 in mice could result in sperm connecting piece structure disassembly and eventually cause ASS (Yuan et al, 2015). Subsequently, Li et al, reported that SUN5, PMFBP1, and CENTLEIN are arranged in a spatially head-to-tail manner, connecting the head and tail of the sperm through direct protein interaction in the connecting piece (Shang et al, 2017; Zhang et al, 2021; Zhu et al, 2018). Sperm connecting piece assembly relies on the transport of cargo proteins through microtubule and microfilament systems in the manchette structure (Yuan et al, 2015; Zheng et al, 2019). Intramanchette transport (IMT) and Intraflagellar transport (IFT) are the main protein transport systems in elongating and elongated spermatids with have similar cytoskeleton components, including microtubules and microfilaments (Kierszenbaum et al, 2002; Pleuger et al, 2020; Yap et al, 2022). During the transport process of sperm protein, myosin along microfilaments and dynein along microtubules act as motor proteins essential for spermatogenesis and male fertility (Kierszenbaum et al, 2003; Teves et al, 2020). For example, MYO5A, a myosin protein, is located in the manchette structure, playing an important role during spermatogenesis (Hayasaka et al, 2008; Kierszenbaum et al, 2003). Abnormal expression and localization of dynein protein DYNLT1 could lead to male infertility (Indu et al, 2015). In addition, recent studies reported that proteins transported by manchette structure were associated with HTCA assembly (Pasek et al, 2016; Tapia Contreras and Hoyer-Fender, 2019; Wang et al, 2023; Zheng et al, 2019). However, the molecular mechanism of the assembly of HTCA remains largely unknown.

Protein folding is the process by which proteins acquire their functional structure and conformation (Bhatia and Udgaonkar, 2022; Moore et al, 2022). In this process, chaperone proteins prevent newly synthesized polypeptide chains and subunits from erroneously forming nonfunctional structures (Kim et al, 2013; Saibil, 2013), while heat shock proteins (HSPs) act as molecular chaperones and form complexes with co-chaperones to regulate protein folding (Hubbard and Sander, 1991; Kim et al, 2013). Previous studies showed that HSPA8 (also known as HSC70) is a chaperone protein and belongs to the HSP70 protein family (Hakui et al, 2022; Stricher et al, 2013; Zhang et al, 2022b). Notably, BAG5 has been reported to act as a co-chaperone by regulating HSP70-mediated protein folding in Parkinson’s disease (PD) and HSPA8-mediated proteostasis in dilated cardiomyopathy (De Snoo et al, 2019; Hakui et al, 2022; Kalia et al, 2004). However, it is still unknown how BAG5 is involved in protein folding and its function in the reproductive system, especially in spermatogenesis.

Here, we first report that loss of BAG5 in mice results in acephalic spermatozoa syndrome and male infertility, accompanied by disorganization of the HTCA. Further, we found that the SPATA6, myosin proteins (MYO5A and MYL6), and dynein proteins (DYNLT1, DCTN1, and DNAL1) were misfolded when BAG5 was depletion in vivo or in vitro. In addition, we discovered that BAG5 could interact with the HSPA8 chaperone and form a complex with HSPA8 to regulate its affinity with SPATA6. Together, these results indicate that BAG5 serves as a co-chaperon of chaperon protein HSPA8 to modulate the proteostasis of myosin proteins (MYO5A and MYL6) and dynein proteins (DYNLT1, DCTN1, and DNAL1) and ablation of BAG5 in mice disrupts the functional motor proteins (myosin and dynein proteins) in sperm manchette structure, which results in some core proteins (e.g., SPATA6) involved in the assembly of HTCA fail to transport into HTCA, thereby leading to the separation of sperm head and tail.

## Results

### BAG5 interacts with SPATA6 in the manchette but does not co-localize in the connecting piece

**Figure 1:**
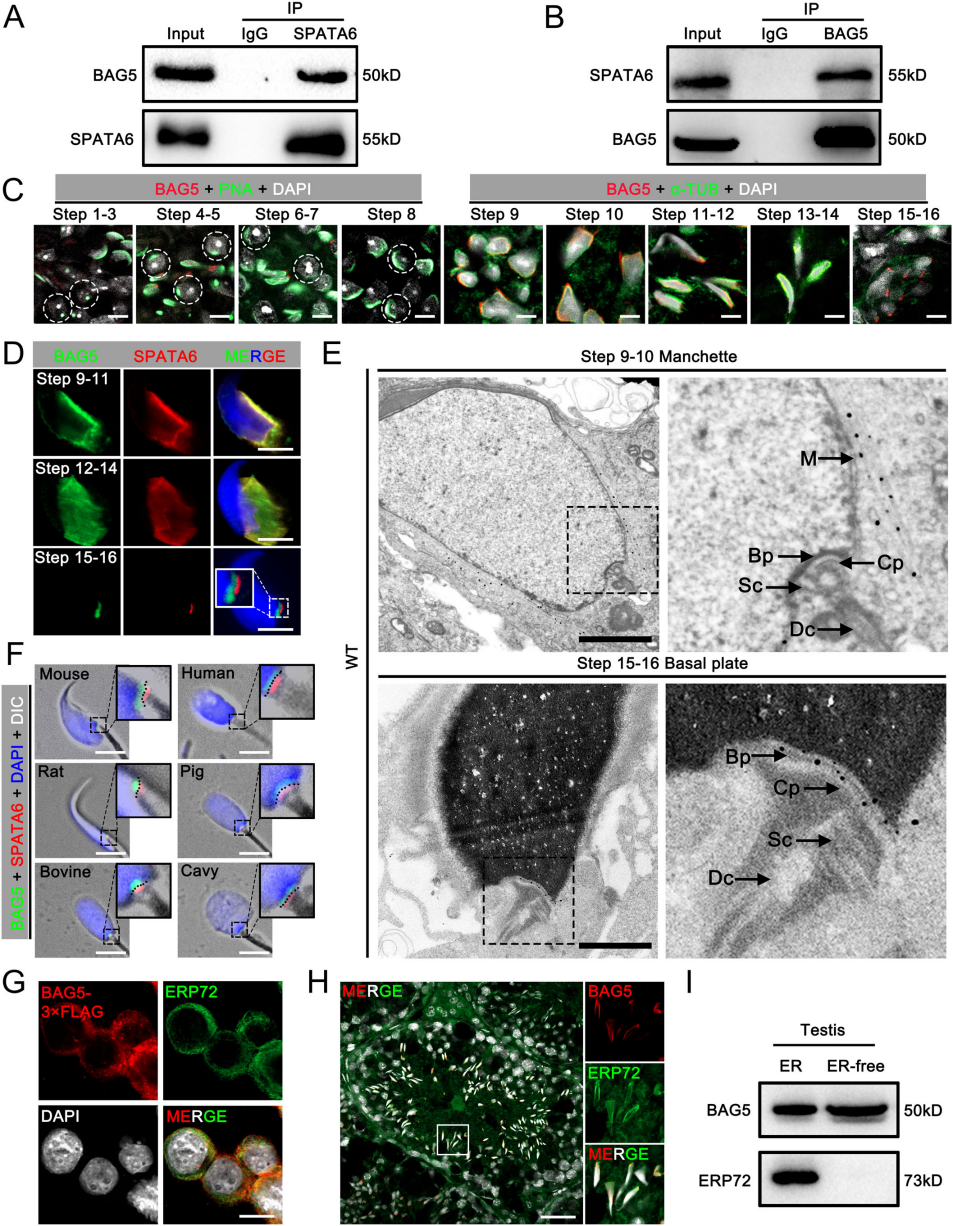
BAG5 interacts with SPATA6 and is expressed in steps 9–16 of the spermatids. (**A**, **B**) Co-immunoprecipitation (Co-IP) assays show the interactions between BAG5 and SPATA6 in mouse testes. IgG was used as a negative control. (**C**) Representative images of the expression of BAG5 (red) during spermiogenesis. PNA (green) marks the acrosome of the spermatid, and α-TUB (green) marks the manchette of the spermatid. DAPI (white) marks the nuclei. Scale bars = 5 μm. (**D**) Representative images of BAG5 (green) and SPATA6 (red) immunodetection on different steps of spermatids are shown. Scale bars = 5 μm. (**E**) Immunogold staining shows the subcellular localization of BAG5 in mouse testicular spermatids. The specific gold particles were detected in the manchette and basal plate regions in wild-type (WT) sperm. M manchette, Bp basal plate, Cp capitulum, Sc segmented column, Dc distal centriole. Scale bars = 1 μm. (**F**) The localization of BAG5 and SPATA6 in sperm from multiple species, including Mouse, Human, Rat, Pig, Bovine, and Cavy. Scale bars = 5 μm. (**G**) Representative images of BAG5-3x FLAG (red) and ERP72 (green) immunostaining on HEK293F cells are shown. HEK293F cells were transfected with BAG5-3x FLAG. ERP72 marks endoplasmic reticulum (ER). Scale bars = 10 μm. (**H**) A representative image of BAG5 (red) and ERP72 (green) immunostaining on testicular cross-section is shown. Scale bar = 50 μm. (**I**) Representative Western blot images show that BAG5 protein was detected in the endoplasmic reticulum of testes. ER-free was the remaining proteins that served as a negative control after extracted ER proteins from mouse testis. Data information: Data in (**A**–**I**) represent results from three independent biological replicate experiments. See also Fig. EV1 and Appendix Figs. S1, S2. Source data are available online for this figure.

Since we have reported ablation of SPATA6 in mice led to sperm connecting piece disassembly and acephalic spermatozoa (Yuan et al, 2015), we performed immunoprecipitation-mass spectrometry (IP/MS) using SPATA6 antibody in this study to identify its interacting proteins in mouse testes. We identified an interesting protein and abundant, BAG5, from the IP/MS data (Fig EV1A and Dataset EV1), which was verified to interact with SPATA6 by Co-IP assays (Fig. 1A,B). We thus focused on BAG5 and examined its expression pattern in multiple mouse organs and spermatogenesis. The results showed that both mRNA and protein of BAG5 were predominantly expressed in the testis (Fig. EV1B,C). To clearly define the expression of BAG5 during spermatogenesis, we characterized the expression of BAG5 in adult testes by immunofluorescence (IF) assays. The IF results showed that BAG5 was absent in steps 1–8 spermatids but highly expressed in steps 9–16 spermatids (Fig. 1C and EV1D), suggesting BAG5 plays a critical role during sperm formation. Intriguingly, BAG5 and SPATA6 were co-localized in steps 9–14 but only shared a spatially adjacent localization, not co-localization at the connecting piece in steps 15–16 spermatids (Fig. 1D). To further determine the subcellular localization of BAG5 in spermatids, we performed immunogold labeling followed by transmission electron microscopy (IG-TEM) on mouse testicular cells (Fig. 1E). Gold particles were detected mainly in the manchette of steps 9–10 spermatids and the basal plate structure of steps 15–16 late spermatids (Fig. 1E). No specific gold particles were detected in *Bag5*-null spermatids stained with the BAG5 antibodies, confirming the sublocalization of BAG5 in the manchette during spermiogenesis and basal plate of spermatozoa (Appendix Fig. S1).

**Figure EV1:**

BAG5 interacts with SAPTA6 and is expressed in testes with high conservation among nine vertebrate species. (**A**) A list of representative SPATA6-interacting partners identified from adult WT mouse testes. (**B**) qRT-PCR analyses of *Bag5* mRNA levels in various mouse tissues. *Bag5* is highly expressed in mouse testes. Data were presented as mean ± SEM. (**C**) Representative western blot shows the expression pattern of BAG5 in various mouse tissues. GAPDH served as the loading control. (**D**) Representative microscopy images of BAG5 (red) and α-TUB (green) immunodetection from adult WT mice are shown on stage IX–X (left) and stage IV–VI (right) seminiferous tubules. Nuclei were stained with DAPI (white). Scale bars = 25 μm. (**E**) Phylogenetic tree of BAG5 orthologs among nine vertebrate species. (**F**) Amino acid sequence similarities among nine vertebrate species. Data information: Data in **B**–**D** represent results from three independent biological replicate experiments. Source data are available online for this figure.

Further comparative analysis of homology and phylogenetic tree based on amino acid sequence showed that BAG5 is a highly conserved protein among multiple vertebrate species, especially BAG5 shares 91.3% amino acid sequences between humans and mice (Fig. EV1E,F; Appendix Fig. S2), suggesting that findings in mouse model also mirror human biology. Considering the high similarities of amino acid sequences, we used epididymis spermatozoa from other mammals to stain BAG5 (Fig. 1F). Similar to the localization of BAG5 in mouse spermatozoa, BAG5 localized to the connecting piece of spermatozoa in human, rat, pig, bovine, and cavy, but also not co-localized with SPATA6 at the connecting piece (Fig. 1F), suggesting that BAG5 also is a structural protein localized at the basal plate with distinctly SPATA6 spatial localization at sperm capitulum (Cp) and segmented column (Sc) in mammals.

Given that BAG5 is a co-chaperon protein and its biological locations are cytoplasm and endoplasmic reticulum (Bruchmann et al, 2013; Hakui et al, 2022; Zhu et al, 2021), we then examined if BAG5 is co-localized with the markers (ERP72) of the endoplasmic reticulum using IF assays. To this end, we first transfected BAG5-3X Flag into the HEK293F cell line in vitro and found that BAG5 could co-localize with ERP72 (Fig. 1G). This localization was further confirmed by in vivo experiments in which BAG5 co-localizes with ERP72 in elongating spermatids of mouse testes (Fig. 1H). In addition, to explore the actual import of BAG5 into the endoplasmic reticulum (ER), we extracted the ER protein from the testis and the results showed that BAG5 was detected in the ER (Fig. 1I). Taken together, these data demonstrate that BAG5 is an evolutionarily conserved protein that interacts with SPATA6 and localizes in the endoplasmic reticulum, manchette structure, and basal plate of sperm connecting piece.

### Inactivation of *Bag5* in mice leads to acephalic spermatozoa syndrome (ASS) and male infertility

**Figure 2:**
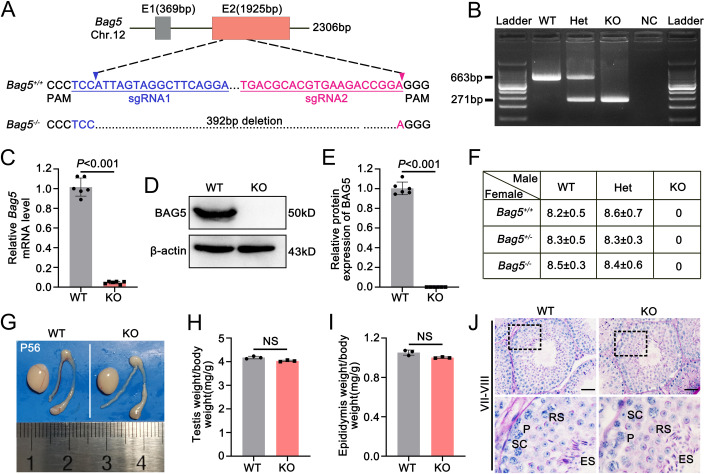
Inactivation of *Bag5* leads to male infertility in mice. (**A**) The schematic diagram for generating *Bag5* knockout (KO) mice by targeting the exon 2 of the *Bag5* gene using the CRISPR/CAS9 strategy. (**B**) Representative PCR-based genotyping of WT (Wildtype), Het (*Bag5*^+/−^), and KO (*Bag5*^−/−^) mice. NC represents negative control. (**C**) qRT-PCR analyses of the expression of mRNA level of *Bag5* in WT and KO mouse testes. Data were presented as mean ±  SD. *P* values (Mann–Whitney test, two-sided). (**D**) Representative western blot showing that BAG5 protein was detected in WT testes but not in KO testes. β-actin served as a loading control. (**E**) Quantification of BAG5 protein level in WT and KO testes. Data were presented as mean ±  SD. *P* values (Student *t*-test, two-sided). (**F**) Fertility test of WT, Het, and KO mice. *Bag5* KO male mice were completely infertile, while the female mice were fertile. Data were presented as mean ± SD. (**G**) Representative gross morphological images of testes, epididymides, and vas deferens from WT and KO mice at P56 are shown. (**H**, **I**) Quantification of testis/body (**H**) and epididymis/body (**I**) weight between WT and KO mice. Data were presented as mean ± SD. NS not significant. *P* values (Student *t*-test, two-sided). (**J**) Representative images of the periodic acid-Schiff (PAS) staining of WT and KO mouse seminiferous tubules in stages VII–VIII are shown. Scale bars = 50 μm. SC sertoli cell, P pachytene spermatocyte, RS round spermatid, ES elongating spermatid. Data information: Data in (**B**–**F**) represent results from six independent experiments (two technical and three biological replicates). Data in (**G**–**J**) represent results from three independent biological replicate experiments. See also Fig EV2. Source data are available online for this figure.

To explore the physiological role of BAG5, we generated a *Bag5* global knockout mouse model using the CRISPER/Cas9 strategy that targeted exon 2 to mutate the *Bag5* gene (Fig. 2A). A heterozygous mutated mouse with 392 base pair deletion of exon 2 was used as an F0 founder mouse mated with a wild-type (WT) mouse to generate heterozygous F1 mice. The resulting F1 heterozygotes (herein designated as *Bag5*^+/−^) were interbred to generate homozygous mice (herein defined as *Bag5*^−/−^ or KO), and all mice were genotyped by genomic DNA PCR (Fig. 2B). The mRNA decreased and protein of BAG5 was not detected in *Bag5*^−/−^ mice using *Bag5*^+/+^ mice as a positive control (herein designated as WT) (Fig. 2C–E), indicating that BAG5 was completely inactivated in KO mice. The complete ablation of BAG5 protein is also supported by the result showing the absence of BAG5 signals in the KO testis of IG-TEM analyses (Appendix Fig. S1).

**Figure EV2:**

Histological analyses of ovaries and testes in WT and KO mice. (**A**) HE staining of WT and KO ovaries. The development of primordial follicle, primary follicle, secondary follicle, and antral follicle was normal in KO ovaries. Scale bars = 400 μm. (**B**) PAS-staining of WT and KO mouse testes. The development of seminiferous epithelia was divided into 12 stages. Scale bars = 50 μm. (**C**) Quantifying the percentage of seminiferous tubules from WT and KO mice at different stages is shown. Data were presented as mean ± SD. NS not significant. *P* values (Student *t*-test, two-sided). (**D**) Quantifying the percentage of seminiferous tubules with vacuoles in WT and KO mice is shown. Data were presented as mean ± SD. NS not significant. *P* values (Student *t*-test, two-sided). (**E**) Representative images of sperm morphology at different steps of spermatids in WT and KO mice are shown. Scale bars = 5 μm. (**F**) Quantifying the ratio of different steps of spermatids with abnormal acrosome in WT and KO mice is shown. Data were presented as mean ± SD. NS not significant. *P* values (Student *t*-test, two-sided). Data information: Data in (**A–C**) represent results from three independent biological replicate experiments. Data in (**D**) represent results from six independent replicate experiments (two technical and three biological replicates). Data in (**E**, **F**) represent results from five independent biological replicate experiments. Source data are available online for this figure.

**Figure 3:**
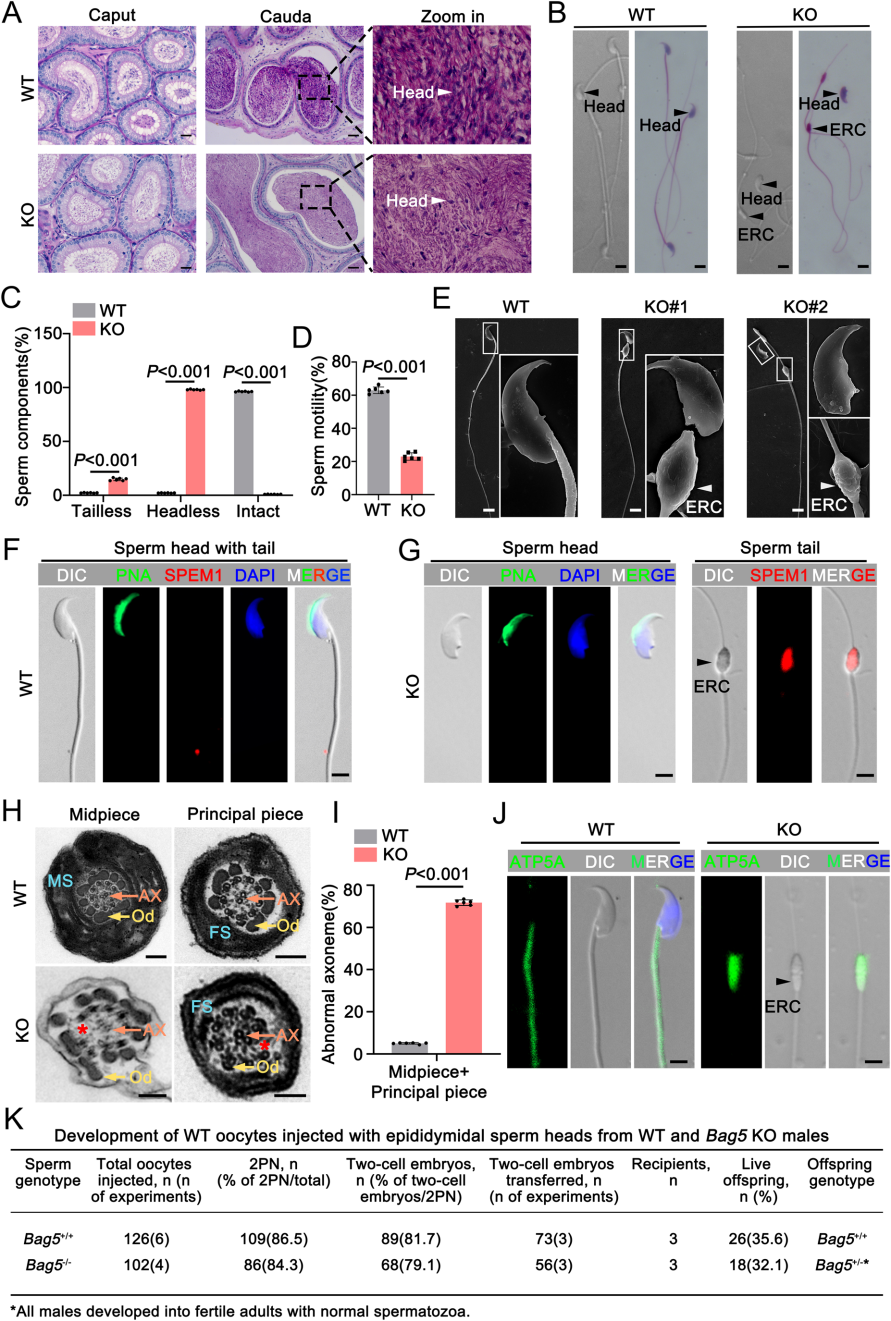
Ablation of BAG5 leads to acephalic spermatozoa in mice. (**A**) Histological analyses of WT and KO epididymides. The sperm head in the caput and cauda of epididymides were significantly decreased. Scale bars = 50 μm. (**B**) Representative microscopy images of differential interference contrast (DIC) and H&E staining on WT and KO epididymal sperm smears are shown. ERC excess residual cytoplasm. Scale bars = 5 μm. (**C**) Histogram shows the proportion of tailless, headless, and intact spermatozoa from WT and KO mouse cauda epididymis. 200 spermatozoa were counted for each mouse. Data were presented as mean ±  SD. *P* values (Student *t*-test, two-sided). (**D**) Significantly decreased motility of *Bag5* KO epididymal sperm. Data were presented as mean ±  SD. See also related Movies EV1 and EV2. *P* values (Mann–Whitney test, two-sided). (**E**) Scanning electron microscopy (SEM) ultrastructural analyses of WT (left) and KO (right) epididymal sperm. Insets are the higher-magnification images of the head and excess residual cytoplasm (ERC) of the sperm. Scale bars = 5 μm. (**F**) Representative images of PNA (green) and SPEM1 (red) immunostaining on WT cauda epididymal intact sperm are shown. SPEM1 marks the cytoplasmic droplets (CDs). Scale bar = 5 μm. (**G**) Representative images of PNA (green) and SPEM1 (red) immunostaining on KO epididymal sperm head and tail are shown. SPEM1 marks the cytoplasmic droplets (CDs). ERC excess residual cytoplasm. Scale bars = 5 μm. (**H**) Transmission electron microscopy (TEM) showing the ultrastructure of the midpiece and principal piece of WT and KO sperm flagella. AX axoneme, FS fiber sheath, Od outer dense fiber, MS mitochondrial sheath. The red asterisk indicates the missing microtubule doublets of the axoneme in KO spermatozoa. Scale bars = 200 nm. (**I**) Quantification of the percentage of the abnormal axoneme (midpiece + principal piece) in WT and KO mouse spermatozoa flagella. 100 midpieces + 100 principal pieces were counted for each mouse. Data were presented as mean ±  SD. *P* values (Student *t*-test, two-sided). (**J**) Representative images of ATP5A (green) immunostaining on WT and KO cauda epididymal sperm are shown. ATP5A marks the mitochondria located in the midpiece of the sperm flagellum. ERC: Excess residual cytoplasm. Scale bar = 5 μm. (**K**) Statistical analysis of offspring development after injection of WT and KO sperm heads into WT oocytes by ICSI. Data information: Data in (**A**–**J**) represent results from six independent experiments (two technical and three biological replicates). Source data are available online for this figure.

Further fertility tests revealed that all the adult KO females were fertile, and no abnormality was observed in the ovarian development of KO females, whereas the adult KO males did not produce pups after breeding with fertility-proven adult WT female mice for 6 months, suggesting that KO male mice are sterile (Fig. 2F and EV2A). To uncover the cause of the infertile phenotype of KO males, we examined the testis and epididymis from WT and KO mice at both gross and histological levels. No differences in both testis and epididymis of size and weight were observed between WT and KO mice (Fig. 2G–I). Further histological analyses by PAS-staining revealed that the seminiferous epithelia of KO mice contained all types of germ cells and all stages of seminiferous tubules, and the percentage of stages of seminiferous tubules was comparable between WT and KO mice (Fig. 2J and EV2B–D). In addition, analyses of spermatids step by step revealed normal sperm acrosome biogenesis and head condensing in KO mice (Fig. EV2E,F). Interestingly, although the seminiferous tubules and the biogenesis of sperm acrosome were normal in *Bag5* KO mice, the epididymides of KO mice appeared to be more weakly stained with hematoxylin stains compared with the WT controls (Fig. 3A). Specifically, sperm heads stained with Mayer’s hematoxylin were rarely observed in KO cauda epididymides (Fig. 3A). Further analysis of epididymal spermatozoa revealed an exciting phenomenon of the sperm head-to-tail separation in KO mice (Fig. 3B; Movies EV1 and EV2), suggesting *Bag5* KO mice exhibit an acephalic spermatozoa syndrome which phenocopied *Spata6* KO mice (Yuan et al, 2015). Quantitative analyses revealed that the counts of tailless-head and headless-tail in the *Bag5* KO mice were significantly increased compared with WT control mice, and the intact spermatozoa were almost absent in KO cauda epididymis (Fig. 3C). Like *Spata6* KO mice, those headless sperm flagella collected from the *Bag5* KO mice displayed limited lower progressive motility through video recording and statistical analysis (Fig. 3D; Movie EV1 and EV2). Moreover, an excess residual cytoplasm (ERC) was observed at the mid-principal piece junction of sperm flagella in *Bag5* KO mice (Fig. 3B).

Next, we analyzed the ultrastructure of epididymal spermatozoa collected from WT and *Bag5* KO mice using scanning electron microscopy (SEM) to identify the morphologically structural defects. Consistent with H&E staining observation, the spermatozoa of WT mice were well connected at head and tail without abnormalities, whereas the spermatozoa of KO mice displayed head-to-tail separation with ERC at the tails akin to *Sptata6* KO spermatozoa (Fig. 3E). Further immunostaining of SPEM1, a marker protein for cytoplasmic droplet (CD), confirmed that the ERC structure of *Bag5* KO sperm flagella was indeed an abnormal enlarged CD-like structure (Fig. 3F,G). TEM revealed that the midpiece of WT spermatozoa is characterized by a well-defined mitochondrial sheath (MS) enclosing nine outer dense fibers (ODFs). In contrast, *Bag5* KO spermatozoa exhibited missed MS containing disorganized ODFs and abnormal axoneme (Fig. 3H,I). In addition, the axoneme comprising the typical “9 + 2” microtubules configuration in WT spermatozoa, whereas the axonemal microtubules of *Bag5* KO spermatozoa were partially missing, showing the “8 + 2” or “7 + 2” microtubular composition in midpiece and principal piece (Fig. 3H,I), which can explain why the *Bag5* KO spermatozoa displaying reduced motility. Due to the absence of MS observed in the midpiece (Fig. 3H), we performed an immunofluorescent experiment using ATP5A, a marker of mitochondria, to assess the mitochondrial sheath arrangements in *Bag5* KO spermatozoa. We found that ATP5A was located in excess residual cytoplasm in *Bag5* KO spermatozoa, whereas the ATP5A-positive signal distribution was in the midpiece of WT spermatozoa (Fig. 3J), further confirming that the mitochondrial sheath was lost in the midpiece of Bag5 KO spermatozoa.

Since previous studies revealed that ASS in *Spata6, Sun5*, and *Pmfbp1* mutation-associated ASS in mice could all be successfully overcome by intracytoplasmic sperm injection (ICSI) (Shang et al, 2017; Yuan et al, 2015; Zhu et al, 2018), we asked whether the sperm heads of *Bag5* KO mice are biologically functional after detaching from flagella. As expected, after injection of KO sperm heads into WT oocytes, fertilized oocytes were able to complete preimplantation development (two-cells to blastocyst stages) and give birth to viable *Bag5*^+/−^ pups (Fig. 3K), suggesting that the *Bag5* KO sperm heads have fertilization potential as well.

### The head-tail coupling apparatus (HTCA) assembly was impaired in *Bag5*-null spermatids

**Figure 4:**
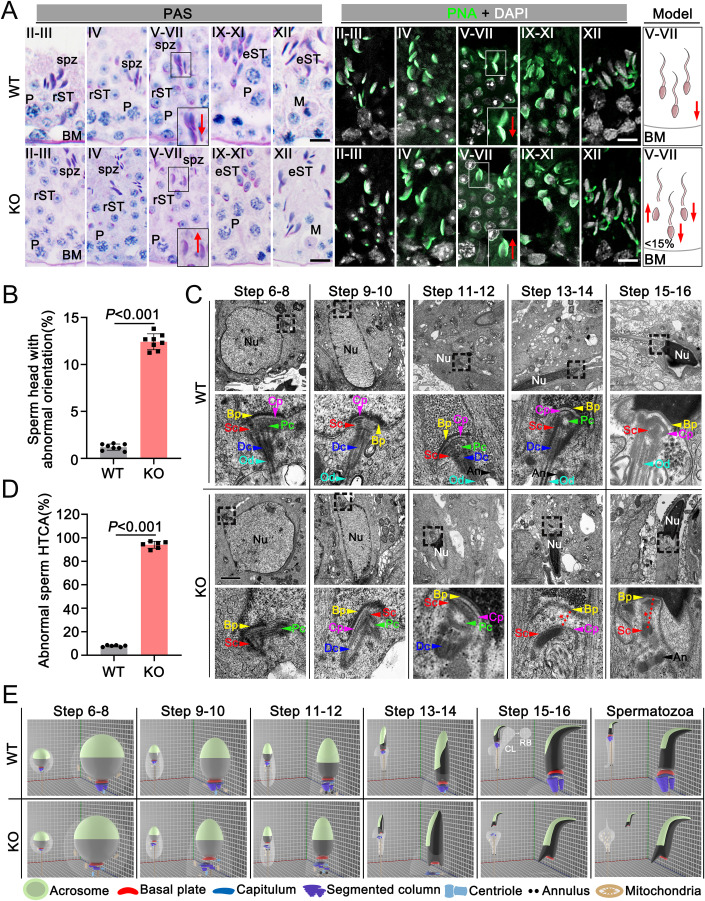
Sperm HTCA fails to assemble normally in *Bag5* KO mice. (**A**) PAS (*left*) and PNA (*middle*) staining of WT and KO mouse seminiferous tubules at different stages are shown. The red arrows indicate the orientation of sperm heads. The right model shows all sperm heads toward the basement membrane of seminiferous tubules in WT and partially toward the lumen in KO mice, respectively. BM basal membrane, P pachytene, rST round spermatid, Spz spermatozoa, eST elongating/elongated spermatid, M metaphase. Scale bars = 10 μm. (**B**) Quantification of the ratio of spermatids with abnormal orientation in WT and KO mouse seminiferous tubules. 100 sperm were counted for each mouse. Data were presented as mean ±  SD. *P* values (Student *t*-test, two-sided). (**C**) TEM analyses of the stepwise development of the coupling apparatus in WT and KO mouse spermatids. The coupling apparatus is well assembled in WT but disorganized in KO spermatids. The red dotted line indicates the gap between the basal plate (Bp) and disorganized HTCA. Nu nuclear, Bp basal plate, Cp capitulum, Sc segmented column, Pc proximal centriole, Dc distal centriole, An annulus, Od outer dense fibers. (**D**) Histogram shows the percentage of abnormal sperm HTCA in WT and KO spermatids. About 30 sperms were counted for each mouse. Data were presented as mean ±  SD. *P* values (Student *t*-test, two-sided). (**E**) The 3D schematic diagram shows the stepwise development of the sperm HTCA in WT and KO mice based on the TEM analyses. CL cytoplasmic lobes, RB residual bodies. Data information: Data in (**A**, **B**) represent results from eight independent experiments (two technical and four biological replicates). Data in (**C**, **D**) represent results from six independent experiments (two technical and three biological replicates). See also Fig. EV3 and Appendix Figs. S3, Fig S4. Source data are available online for this figure.

To clarify the specific period of sperm head-to-tail separation, we performed 12-stage luminal staging analyses based on the PAS-stained and PNA-stained slices of testicular sections. Interestingly, at stages V–VII, unlike the sperm heads were all toward the basal membrane direction of seminiferous tubules in WT mice, the orientation of partial sperm heads appeared to be toward the lumen of the seminiferous tubules in *Bag5* KO mice, while the orientation of sperm flagellum was relatively arranged normally (Fig. 4A; Appendix Fig. S3). Quantitative analysis indicated that the proportion of abnormal head orientation was as high as ~12% in KO mice, while only ~1% in WT mice (Fig. 4B). We then used transmission electron microscopy (TEM) to examine the spermatids step by step within testes to define the underlying ultrastructure defects of HTCA. In WT step 6–8 spermatids, the HTCA consisted of the following components: basal plate (Bp), capitulum (Cp), segmented column (Sc), proximal centriole (Pc), and distal centriole (Dc) (Fig. 4C). With spermatids differentiation, the flagellum was attached to the nuclear envelope (NE) via HTCA at the implantation fossa (Fig. 4C). In KO mice, the Cp and Sc were abnormally developed from steps 6–8 of spermatids, and the distance between the Bp and the Cp increased at steps 11–12 (Fig. 4C). During the mature stage of spermatids (steps 13–16), the Cp and Sc cannot be assembled correctly, and the abnormal percentage of HTCA reaches ~98% (Fig. 4C,D). Additionally, as observed in WT spermatids, the axoneme extends outwards from the cell surface and reaches the tubular lumen. The centrioles and the axoneme invaginate the cell and cause the cell membrane to become infolded. However, no cytoplasmic surface was seen at the posterior end of the HTCA in *Bag5* KO spermatids, suggesting that cytoplasmic invagination is abnormal in *Bag5* KO mice. Interestingly, the quantitative analysis of TEM images revealed that ~63% of *Bag5* KO spermatids showed abnormal cytoplasmic invagination (Appendix Fig. S4). Ultrastructural analyses, taken together, revealed that the failure of the HTCA assembly leads to the instability of the sperm neck region, ultimately resulting in the detachment of sperm heads from flagella. To better understand the assembly of HTCA, we drew a 3D diagram to visualize the stepwise development of WT and *Bag5* KO spermatids (Fig. 4E).

**Figure EV3:**

The analyses of acephalic spermatozoa-related protein expression in WT and KO mice. (**A**) Representative immunofluorescence images of BAG5 (red) and SPATA6 (green) in steps 13–15 of spermatids from WT and KO mice are shown. Nuclei were stained with DAPI (blue). The double arrow represents the separation of sperm head and tail. Scale bar = 2 μm. (**B**) Representative immunofluorescence images of SUN5 (red) and SPATA6 (green) in steps 13–15 of spermatids from WT and KO mice are shown. Nuclei were stained with DAPI (blue). The double arrow represents the separation of sperm head and tail. Scale bar = 2 μm. (**C**) Representative immunofluorescence images of CENTLEIN (red) and PMFBP1 (green) in steps 13–15 of spermatids from WT and KO mice are shown. Nuclei were stained with DAPI (blue). The double arrow represents the separation of sperm head and tail. Scale bar = 2 μm. (**D**–**G**) In vitro Co-IP assays to examine the interaction between FLAG-BAG5 and MYC-SPATA6 (**D**), MYC-SUN5 (**E**), MYC-CENTLENIN (**F**), and MYC-PMFBP1 (**G**). Data information: Data in (**A**–**G**) represent results from three independent biological replicate experiments. Source data are available online for this figure.

Given that SUN5, CENTLEIN, PMFBP1, and SPATA6 were all located at the HTCA and SPATA6 was specifically located in the Cp and Sc (Yuan et al, 2015; Zhang et al, 2021), we next asked if the localizations of those HTCA proteins are affected on *Bag5* KO spermatids by IF experiments. Interestingly, SPATA6 was found to be localized in the decapitated sperm tail of *Bag5* KO mice, suggesting that Cp and Sc detached from the sperm head upon BAG5 depletion (Fig. EV3A,B). However, the SUN5, CENTLEIN, and PMFBP1 were all left on the detached sperm head of *Bag5* KO mice (Fig. EV3B, C), indicating that SUN5, CENTLEIN, and PMFBP1 component structures remain in the sperm head upon *Bag5* mutation. These results raised a possible protein interaction network among these HTCA proteins in regulating sperm head-to-tail linkage. To test this, we performed Co-IP assays in the HEK293T cell line with transfected an indicated gene plasmid and found that BAG5 could interact with SPATA6 but not with SUN5, CENTLEIN, and PMFBP1 (Fig. EV3D–G). Taken together, these data hint that BAG5 may regulate SPATA6 to be involved in the assembly of the HTCA.

### Ablation of BAG5 leads to misfolding of SPATA6, myosin, and dynein proteins

**Figure 5:**
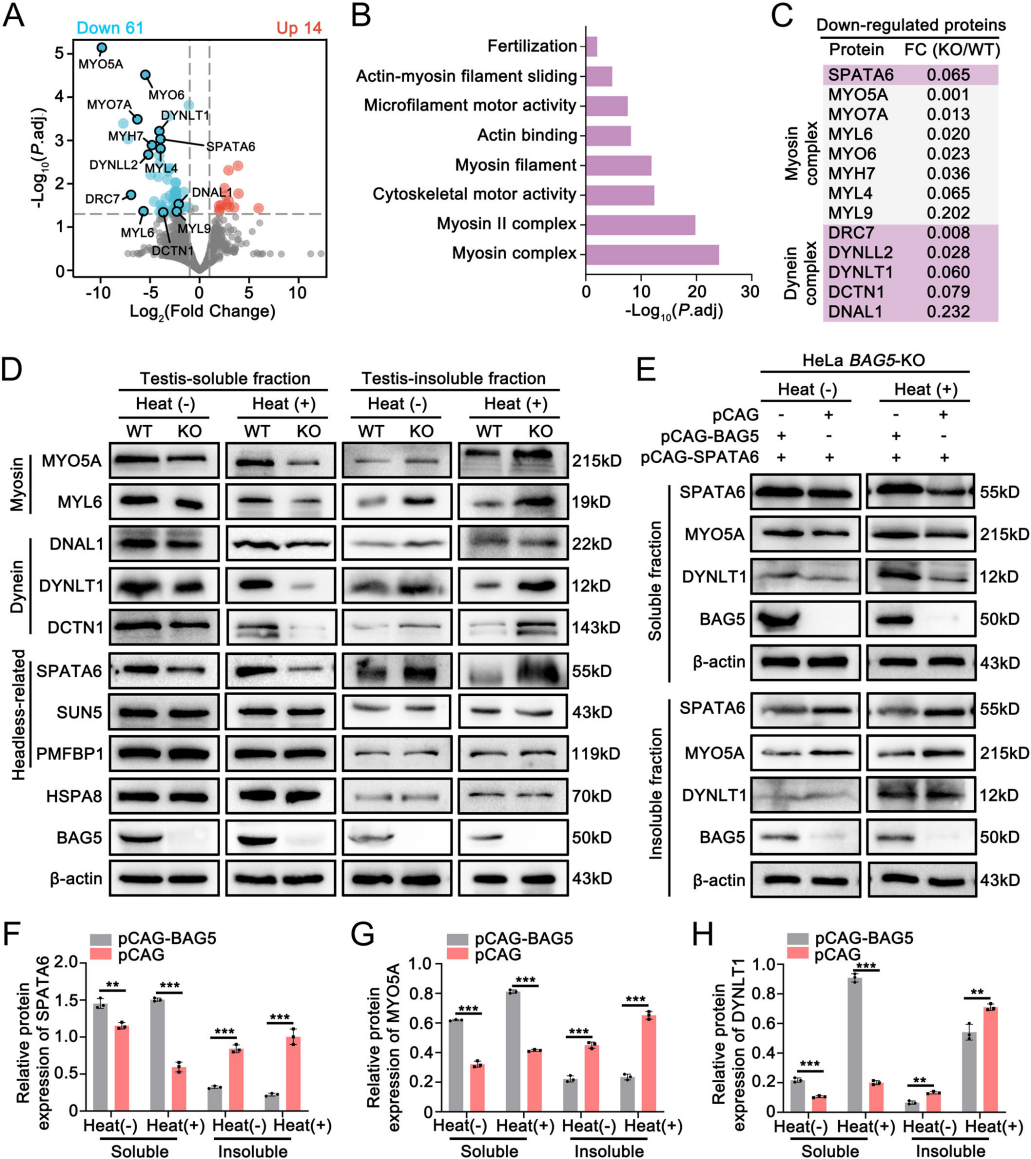
BAG5 regulates the protein folding of SPATA6, myosin, and dynein proteins. (**A**) Volcano plot showing the downregulated and upregulated proteins in *Bag5* KO testes compared with WT testes. *n* = 2 (*n* represents number of mice). *P* values (Student *t*-test, two-sided). (**B**) GO term enrichment analyses of the downregulated proteins in *Bag5* KO testes. *P* values (Hypergeometric distribution test). (**C**) A list of downregulated proteins associated with myosin and dynein complex. (**D**) The protein abundance in soluble and insoluble fractions of WT and *Bag5* KO mouse testes with or without 42 °C heat shock (2 h per day for 5 days). β-actin serves as a loading control. (**E**) The protein abundance in soluble and insoluble fractions of WT and *BAG5* KO HeLa cells with or without 42 °C heat shock (2 h per day for 5 days) after co-transfected with pCAG-BAG5 (or pCAG) and pCAG-SPATA6 plasmids. β-actin serves as a loading control. (**F**–**H**) Quantitative analyses of the protein expression level of SPATA6, MYO5A, and DYNLT1. Data were presented as mean ±  SD. **P < 0.01, ***P < 0.001. *P* values (Student *t*-test, two-sided). Data information: Data in (**D**–**H**) represent results from three independent biological replicate experiments. See also Fig. EV4 and Appendix Figs. S5, S6. Source data are available online for this figure.

To uncover the underlying molecular reasons for the impairment of sperm HTCA in *Bag5* KO mice, we lysed proteins from WT and KO testes and took soluble supernatants for protein mass spectrometry analysis. Compared to WT testes, we identified 14 upregulated and 61 downregulated proteins in KO testes (Fig. 5A; Dataset EV2). GO enrichment analysis of 61 downregulated proteins showed that these downregulated proteins were enriched in myosin complex and cytoskeletal motor activity (Fig. 5B,C). We then chose myosin proteins (MYO5A and MYL6) and dynein proteins (DNAL1, DYNLT1, and DCTN1) to assess their expression in both the soluble fractions (containing correctly folded protein) and insoluble fractions (containing misfolded protein). Interestingly, we found that these five selected proteins were reduced in supernatant-soluble fractions and increased in aggregation-insoluble fractions in KO testes compared with WT testes (Fig. 5D; Appendix Fig. S5A–E), suggesting that the protein folding of myosin and dynein proteins was disturbed in *Bag5* KO mice. Given that heat stress could aggravate the protein misfolded and aggregation of insufficiently folding proteins (Wilkening et al, 2018), we treated the mouse testes with heat shock in a 42 °C water bath (5 days, 2 h per day) to further examine the effect of impaired protein folding. As expected, correctly folded proteins decreased more obviously, while the insoluble misfolding protein significantly increased after heat shock treatment (Fig. 5D; Appendix Fig. S5A–E). We next examined if the protein folding of HTCA essential proteins SPATA6, SUN5, and PMFBP1 was affected upon BAG5 depletion. The results showed that in the absence of BAG5, the protein solubility of SPATA6 decreased, especially under heat shock stress conditions, while SUN5 and PMFBP1 protein remained soluble and did not display any changes (Fig. 5D; Appendix Fig S5F–H), suggesting that BAG5 could regulate the protein folding of essential HTCA protein SPATA6, not SUN5 and PMFBP1. To rule out the effect of transcription on protein expression levels, we detected the mRNA expression of those genes and confirmed that their mRNA levels were comparable between WT and KO testes, either in normal or heat shock conditions (Appendix Fig. S6A–I).

**Figure EV4:**
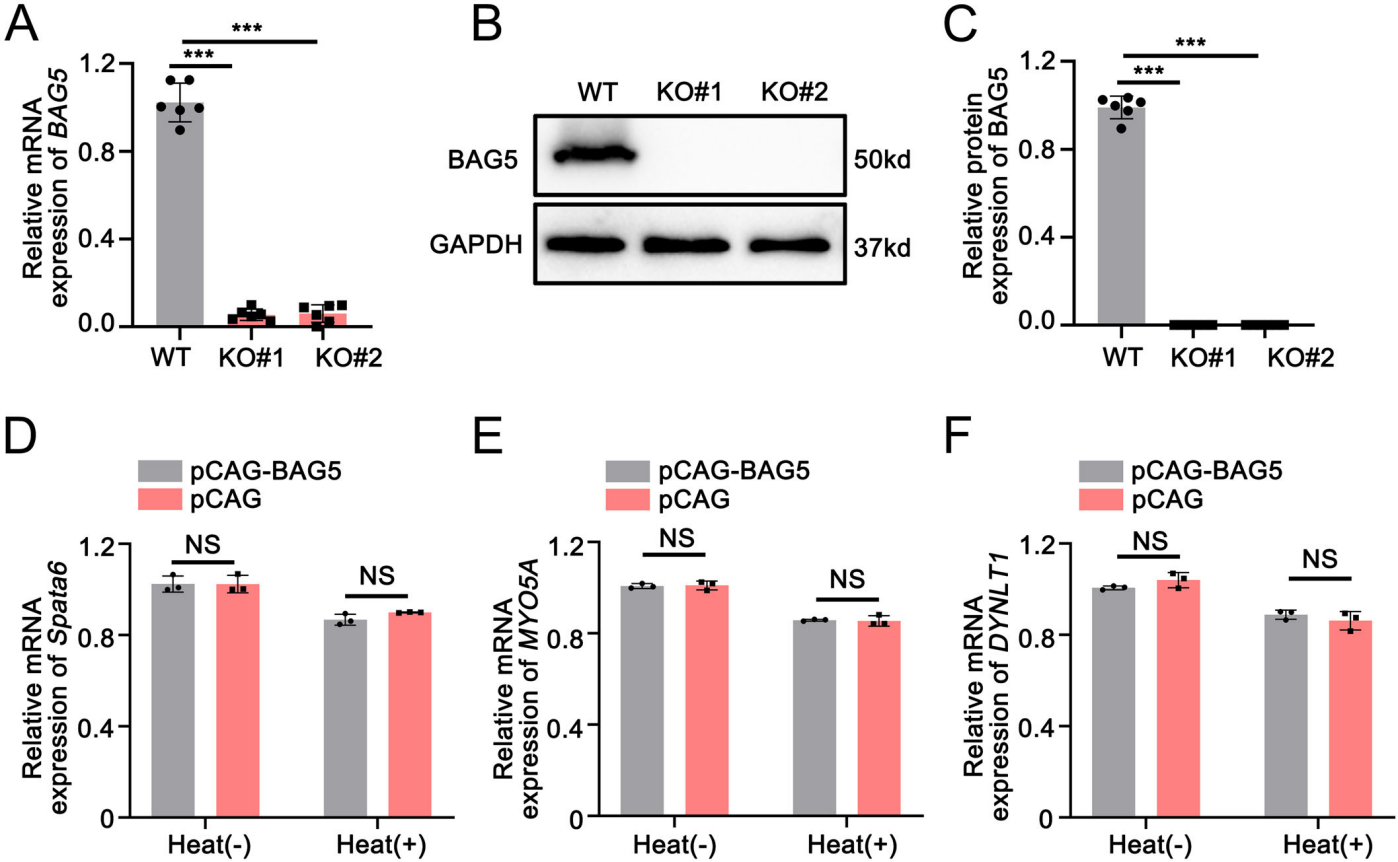
Establishment of *BAG5* KO cell line. (**A**) Histogram shows the relative *BAG5* mRNA expression level in WT and two *BAG5* KO HeLa cell lines. Data were presented as mean ± SD. *P* values (Student *t*-test, two-sided). ***P < 0.001. (**B**) The representative images of the protein expression of BAG5 in WT and two *BAG5*-KO HeLa cell lines are shown. (**C**) The BAG5 protein expression level is quantified in WT and two *BAG5* KO HeLa cell lines in (**B**). Data were presented as mean ± SD. *P* values (Student *t*-test, two-sided). ***P < 0.001. (**D**–**F)** Histograms showing the mRNA expression level of genes (*Spata6, Myo5a*, and *Dynlt1*) in Fig. 5E. NS not significant. Data were presented as mean ± SD. *P* values (Student *t*-test, two-sided). Data information: Data in (**A**–**C**) represent results from six independent experiments (two technical and three biological replicates). Data in (**D**–**F**) represent results from three independent biological replicate experiments. Source data are available online for this figure.

To further confirm the protein folding function of BAG5, we generated the *BAG5* KO HeLa cell line (Fig. EV4A–C) and performed the above experiments in vitro. Consistent with in vivo analyses in testes, in the *BAG5* KO cell line, the transcription of SPATA6, myosin proteins (MYO5A), and dynein proteins (DYNLT1) was normal (Fig. EV4D–F), while the protein expression level of these proteins was downregulated in supernatants and increased in aggregates, and this trend was more pronounced after 42 °C heat shock treatment (5 days, 2 h per day) (Fig. 5E–H). In conclusion, these data indicate that BAG5 could regulate the folding of SPATA6, myosin, and dynein proteins.

### BAG5 interacts with chaperone protein HSPA8 to mediate protein folding

**Figure 6:**
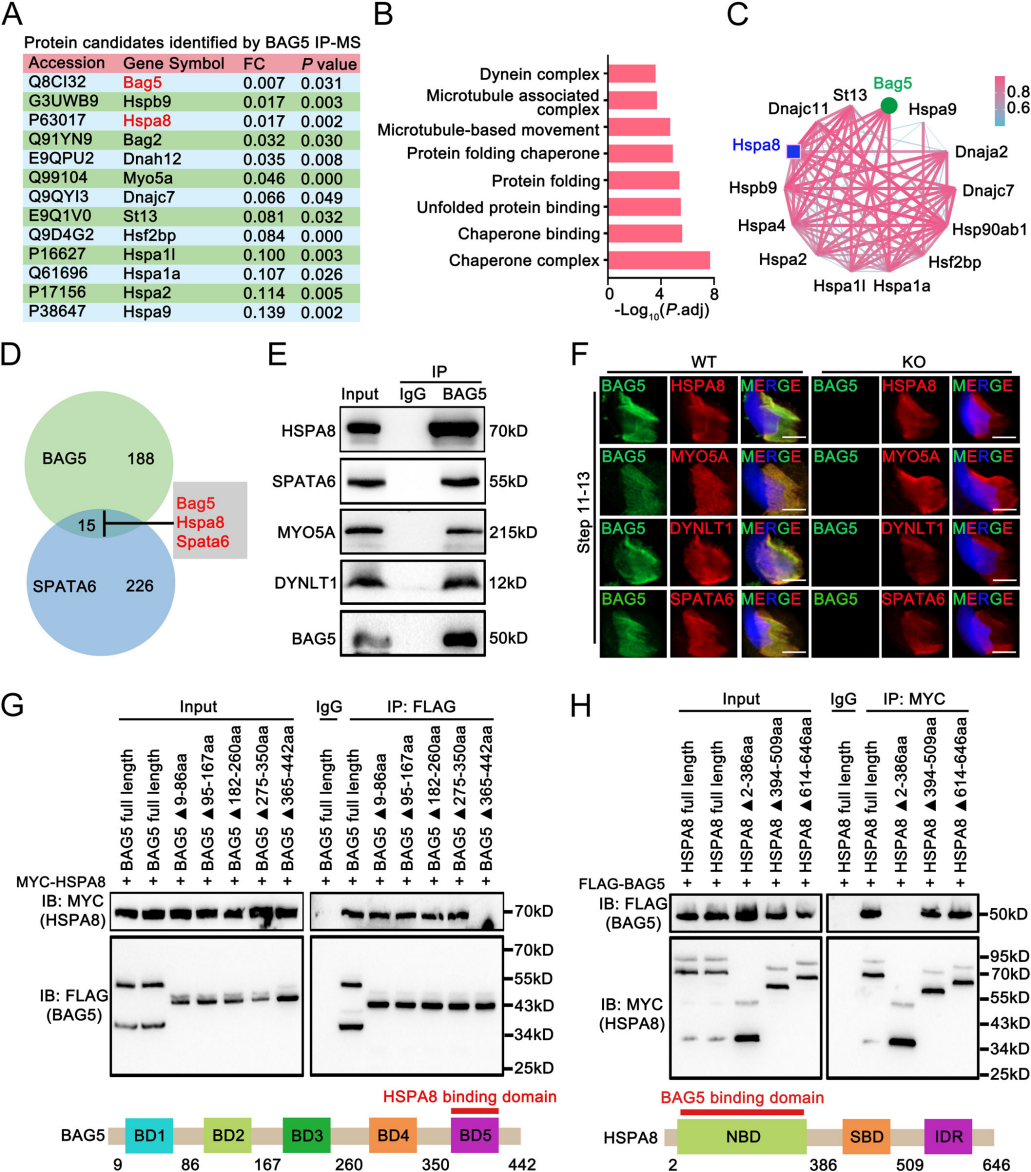
BAG5 interacts with chaperone protein HSPA8 through its NBD domain. (**A**) A list of representative BAG5-interacting partners identified from adult WT mouse testes is shown. FC fold change (KO/WT). *P* values (Student *t*-test, two-sided). (**B**) GO analyses of BAG5-interacting proteins identified from IP-MS. *P* values (Hypergeometric distribution test). (**C**) The String 11.5 program (http://string-db.org) analyses the protein interaction networks among 14 candidate proteins associated with heat shock proteins. (**D**) A comparison of BAG5 and SPATA6-interacting protein networks. (**E**) Co-immunoprecipitation (Co-IP) assays validated the interactions between BAG5 and four putative BAG5-interacting proteins (HSPA8, SPATA6, MYO5A, and DYNLT1) in mouse testes. IgG serves as a negative control. (**F**) Immunofluorescence stainings show the localization of HSPA8, MYO5A, DYNLT1, and SPATA6 in step 11–13 spermatids from WT and KO mice. Scale bars = 5 μm. (**G**, **H**) Reciprocal Co-IP assays of interaction domains between BAG5 and its binding partner HSPA8 are shown. HEK293T cells were co-transfected with MYC-HSPA8 and the indicated fragments of FLAG-BAG5 (**G**) or co-transfected with FLAG-BAG5 and the indicated fragments of MYC-HSPA8 (**H**). Data information: Data in (**E**–**H**) represent results from three independent biological replicate experiments. Source data are available online for this figure.

To investigate the protein interaction network of BAG5, we performed immunoprecipitation-mass spectrometry (IP-MS) using the BAG5 antibody to screen BAG5-interacting proteins in testes. The proteins that IP-MS identified in KO testes served as the negative control. The BAG5 antibody pulled down a total of 188 specific proteins, and interestingly, we found many BAG5-interacting candidates belonged to the heat shock protein family (Fig. 6A; Dataset EV3). Gene Ontology (GO) analyses revealed that those candidate proteins are enriched in chaperone complex, chaperone binding, and microtubule-based movement (Fig. 6B). In addition, the protein-protein interaction network analysis by the STRING database revealed that those BAG5-interacting candidates are indeed involved in the heat shock protein interaction network (Fig. 6C).

Considering that SPATA6 regulates the formation of HTCA and interacts with BAG5, we thus compared the interacting proteins of BAG5 and SPATA6. Interestingly, among the interacting proteins of SPATA6 and BAG5, 15 proteins were found to interact with both BAG5 and SPATA6 (Fig. 6D). Of note, HSPA8 (also named as heat shock cognate 70 kDa protein, HSC70) is a molecular chaperone protein that maintains protein homeostasis under physiological and stress conditions (Valek et al, 2019; Wang et al, 2021; Wang et al, 2022a), which was identified to have a high interaction with BAG5 (Fig. 6A). Since BAG5 is a known co-chaperone that acts as a nucleotide exchange factor for HSPA8, promoting adenosine diphosphate release and activating HSPA8-mediated protein refolding (Arakawa et al, 2010; Hakui et al, 2022; Takayama et al, 1999), we then chose HSPA8 to perform Co-IP (co-immunoprecipitation) and IF assays to validate the interaction between BAG5 and HSPA8. The results confirmed that BAG5 could interact and co-localize with HSPA8 in testes (Fig. 6E,F). To reveal which domain mediated the interaction between BAG5 and HSPA8, we co-expressed BAG5 and HSPA8 full-length proteins or truncated domain proteins in HEK293T cells. Reciprocal Co-IP assays demonstrated that the BD5 domain of the BAG5 protein and the NBD domain of the HSPA8 protein are essential for their interaction (Fig. 6G,H). In addition, Co-IP showed that BAG5 interacts with SPATA6, MYO5A, and DYNLT1(Fig. 6E), which are misfolded in the *Bag5* KO testes. Further, IF analyses revealed the co-localization of BAG5 with SPATA6, MYO5A, and DYNLT1 in elongating spermatids, supporting the interaction of BAG5 with SPATA6, MYO5A, and DYNLT1 in testes (Fig. 6F).

**Figure 7:**
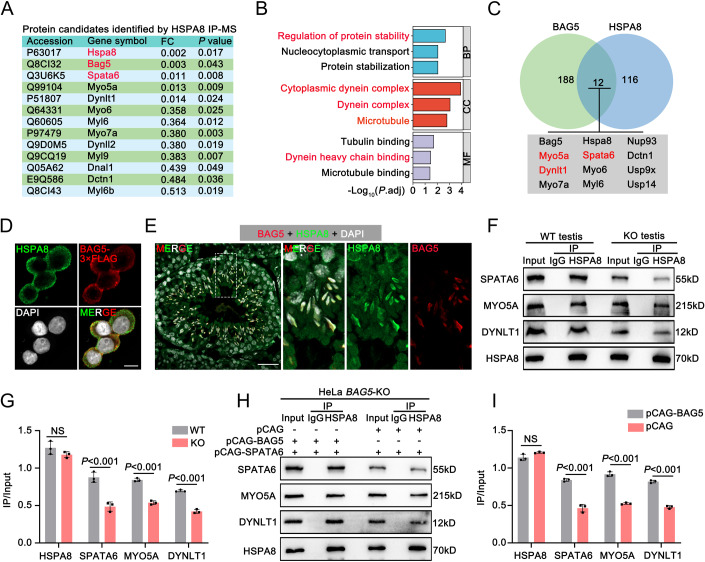
The interaction between HSPA8 and its protein substrates is disrupted in the absence of BAG5. (**A**) A list of representative HSPA8-interacting partners identified from adult WT mouse testes. FC fold change (KO/WT). *P* values (Student *t*-test, two-sided). (**B**) GO and KEGG enrichment analyses of HSPA8-interacting proteins identified from IP-MS. *P* values (Hypergeometric distribution test). (**C**) A comparison of BAG5 and HSPA8-interacting proteins. (**D**) Immunofluorescence stainings show the co-localization of HSPA8 (green) and BAG5 (red) in HEK293F cells. HEK293F cells were co-transfected with HSPA8 and BAG5-3x FLAG. Scale bars = 10 μm. (**E**) Co-immunofluorescence stainings show the co-localization of BAG5 (red) and HSPA8 (green) in adult WT mouse testicular sections (Stage XI). Scale bars = 50 μm. (**F**) Co-IP assays show the interactions between HSPA8 and its substrates (SPATA6, MYO5A, and DYNLT1) in WT and KO testes. The interactions were weakened in KO testes compared to WT testes. IgG serves as a negative control. (**G**) Histogram showing quantification of IP/Input ratio of HSPA8, SPATA6, MYO5A, and DYNLT1 proteins. Data are presented as mean ±  SD. NS not significant. *P* values (Student *t-*test, two-sided). (**H**) Co-IP assays show the interactions between HSPA8 and its substrates (SPATA6, MYO5A, and DYNLT1) in WT and KO HeLa cells after co-transfected with pCAG-BAG5 (or pCAG) and pCAG-SPATA6 plasmids. The interactions were weakened in KO compared to WT HeLa cells. IgG serves as a negative control. (**I**) Histogram showing quantification of IP/Input ratio of HSPA8, SPATA6, MYO5A, and DYNLT1 proteins in WT and KO HeLa cells. Data were presented as mean ± SD. NS not significant. *P* values (Student *t*-test, two-sided). Data information: Data in (**D**–**I**) represent results from three independent biological replicate experiments. Source data are available online for this figure.

To further investigate how BAG5 and HSPA8 cooperate physiologically, we performed proteomic analysis of HSPA8-binding proteins in mouse testes. From the pull-down products by the HSPA8 antibody, 116 specifically interacted proteins were identified to interact with HSPA8 (Fig. 7A; Dataset EV4). Strikingly, GO analyses revealed these proteins enriched in motor activity and dynein complexes (Fig. 7B), consistent with our observations of the misfolding proteins in *Bag5* KO testes. We then compared the interacting proteins between HSPA8 and BAG5 and identified 12 common interacting proteins, including SPATA6, MYO5A, and DYNLT1 (Fig. 7C). Further, in vitro experiments confirmed the co-localization of HSPA8 and BAG5 in the HEK293F cells (Fig. 7D), and more importantly, the examination for BAG5 and HSPA8 verified that BAG5 co-localized with HSPA8 along the manchette structure (Fig. 7E).

Interestingly, we found that in WT testes, HSPA8 protein strongly interacts with SPATA6, MYO5A, and DYNLT1 proteins, whereas these interactions weakened in *Bag5* KO testes, suggesting that BAG5 powers the stability of the HSPA8 protein complex and regulates HSPA8 target proteins (Fig. 7F,G). In support of these results, we utilized BAG5 KO HeLa cells in vitro to investigate whether BAG5 could regulate the stability of the HSPA8 interaction complex. Consistent with the in vivo data, *BAG5* KO HeLa cells also displayed weakened interactions of HSPA8 protein with SPATA6, MYO5A, and DYNLT1 (Fig. 7H,I). Taken together, these data suggest that BAG5 may act as a co-chaperone of HSPA8 to form a complex with HSPA8 to regulate HSPA8 for correctly SPATA6, MYO5A, and DYNLT1 protein folding.

### BAG5 regulates the folding of SPATA6 via the affinity of HSPA8 to its substrate

**Figure 8:**
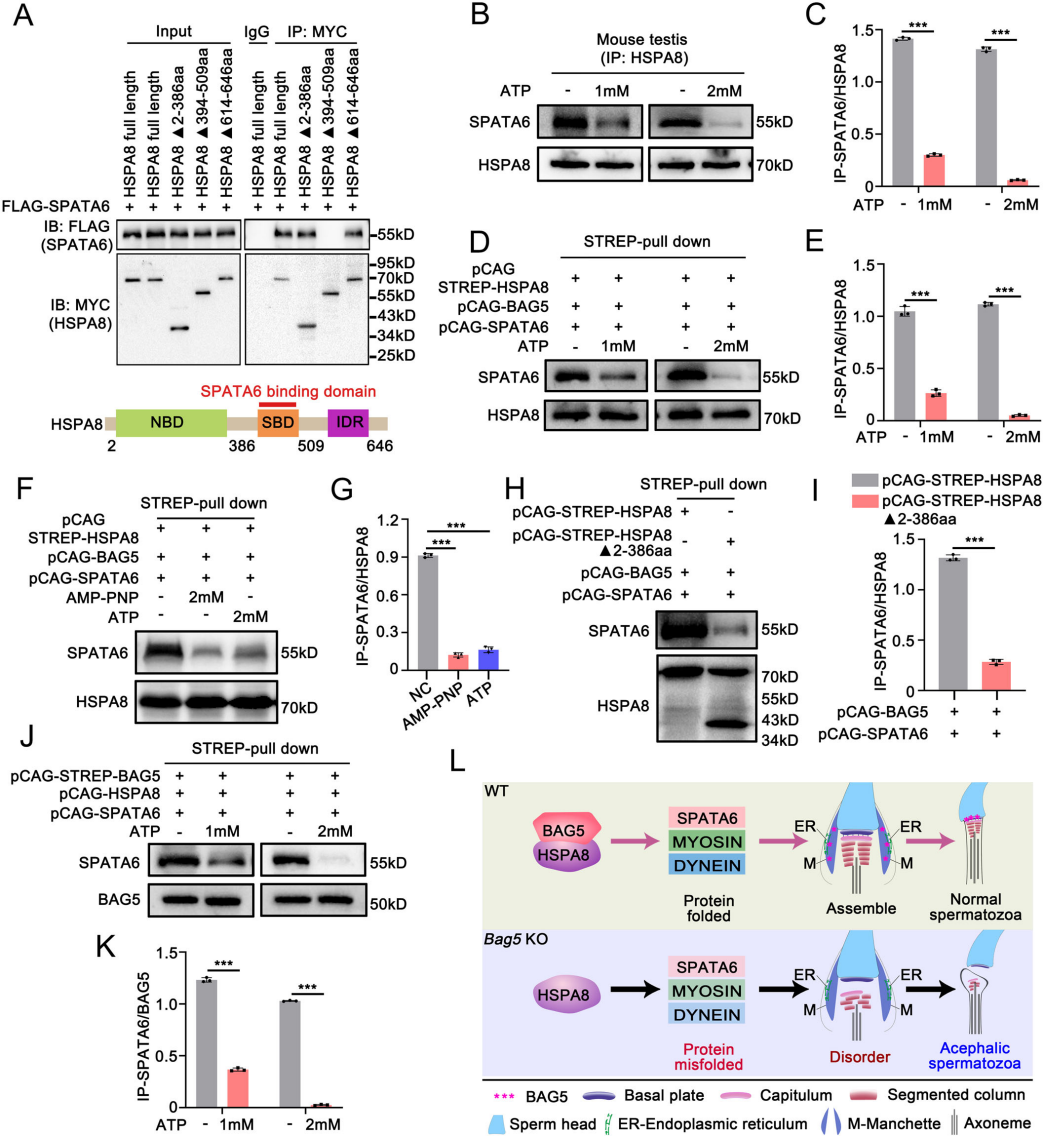
HSPA8 interacts with SPATA6 through its SBD domain and forms a complex with BAG5 and SPATA6. (**A**) Co-IP assays show the interaction domains of HSPA8 with SPATA6. HEK293T cells were co-transfected with FLAG-SPATA6 and the indicated fragments of MYC-HSPA8. (**B**) In vivo detection of the interactions between HSPA8 and SPATA6 in mouse testes after being treated with ATP. (**C**) Histogram showing quantification of IP ratio of SPATA6 / HSPA8 for (**B**). Data were presented as mean ± SD. *P* values (Student *t*-test, two-sided).***P < 0.001. (**D**) In vitro detection of the interactions between HSPA8 and SPATA6 in HEK293F cells after co-transfected with pCAG-STREP-HSPA8, pCAG-BAG5, and pCAG-SPATA6 plasmids. The protein lysis was being treated with ATP. (**E**) Histogram showing quantification of IP ratio of SPATA6 / HSPA8 for (**D**). Data were presented as mean ± SD. *P* values (Student *t*-test, two-sided).***P < 0.001. (**F**) In vitro detection of the interactions between HSPA8 and SPATA6 in HEK293F cells after co-transfected with pCAG-STREP-HSPA8, pCAG-BAG5, and pCAG-SPATA6 plasmids. The protein lysis was treated with ATP and AMP-PNP (Non-hydrolysable analog of ATP). (**G**) Histogram showing quantification of IP ratio of SPATA6/HSPA8 for (**F**). NC negative control, indicated no treatment with ATP or AMP/PNP. Data were presented as mean ± SD. *P* values (Student *t*-test, two-sided).***P < 0.001. (**H**) In vitro detection of the interactions between HSPA8 and SPATA6 after transfected pCAG-STREP-HSPA8 and nucleotide-binding domain mutant of pCAG-STREP-HSPA8 (deletion of 2–386 amino acids) into HEK293F cells, respectively, with pCAG-BAG5 and pCAG-SPATA6 plasmids. (**I**) Histogram showing quantification of IP ratio of SPATA6/HSPA8 for (**H**). Data were presented as mean ± SD. *P* values (Student *t*-test, two-sided).***P < 0.001. (**J**) In vitro detection of the BAG5-interacting partner (SPATA6) with or without ATP treatment after co-transfected with pCAG-STREP-BAG5, pCAG-HSPA8, and pCAG-SPATA6 plasmids. (**K**) Histogram showing quantification of IP ratio of SPATA6 / BAG5 for (**J**). Data were presented as mean ± SD. *P* values (Student *t*-test, two-sided).***P < 0.001. (**L**) Schematic working model of BAG5 in the assembly of HTCA. BAG5 binds key chaperone proteins HSPA8 to regulate the protein folding of SPATA6, MYOSIN, and DYNEIN proteins. MYOSIN and DYNEIN proteins are critical for transporting proteins during HTCA formation in elongating and elongated spermatids. Data information: Data in (**A**–**K**) represent results from three independent biological replicate experiments. See also Fig. EV5. Source data are available online for this figure.

**Figure EV5:**
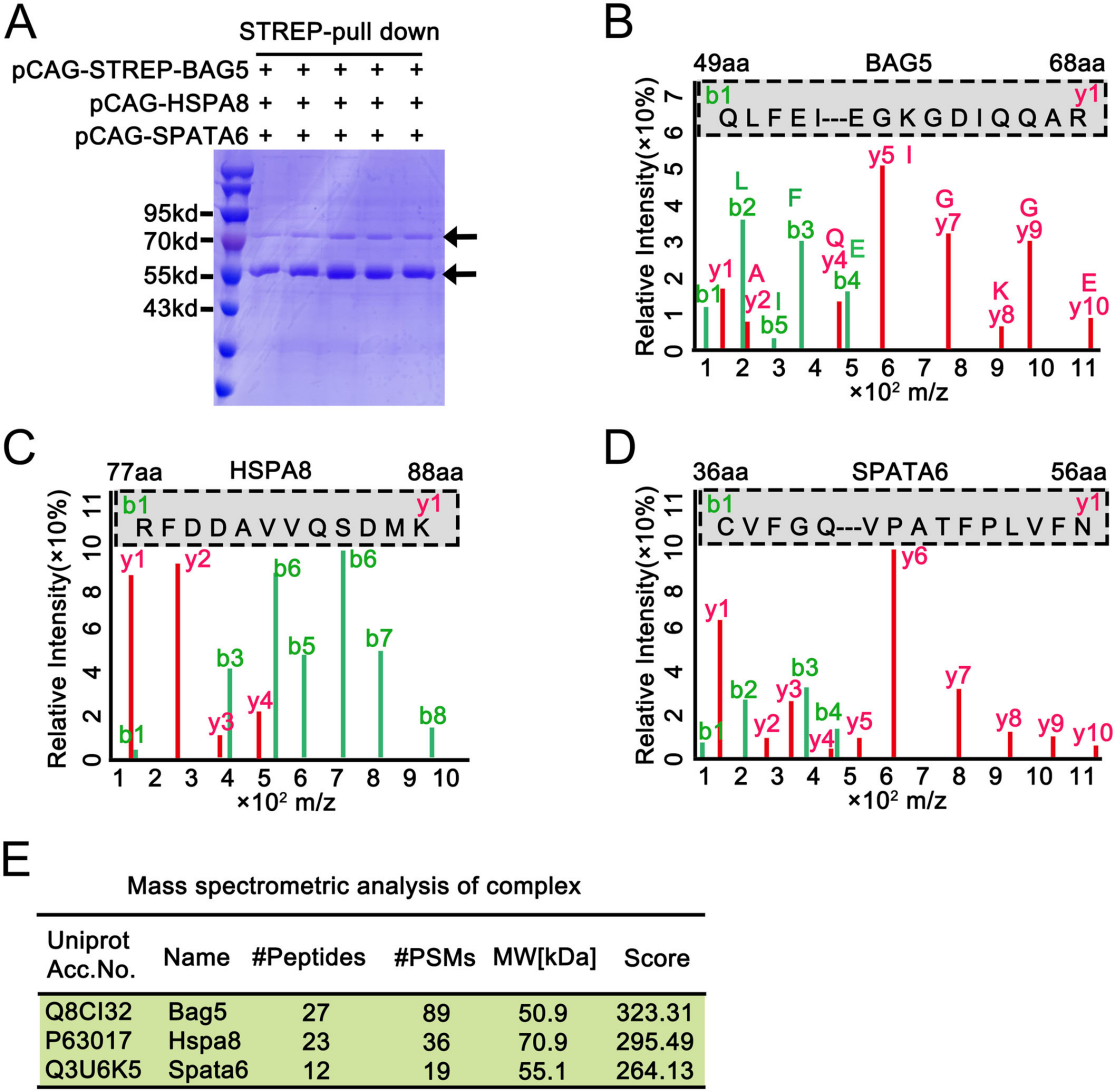
Analyses of the BAG5-HSPA8-SPATA6 protein complex. (**A**) The representative images of Coomassie brilliant blue staining from Strep-tag affinity chromatography purified protein complexes are shown after co-transfected with pCAG-STREP-BAG5, pCAG-HSPA8, and pCAG-SPATA6 plasmids. The arrows indicated the protein molecular weights of 70 kd and 55 kd. STREP-BAG5 indicated protein molecular weight 55 KD, STREP 5 KD plus BAG5 50 KD. (**B**–**D)** Representative MS spectra for specific peptides of BAG5 (**B**), HSPA8 (**C**), and SPATA6 (**D**) proteins. (**E**) The table shows the proteins detected by mass spectrometry of STREP-pull-down complexes. BAG5 protein molecular weight was 50 KD in the UniProt database. Data information: Data in (**A**) represent results from three independent biological replicates experiments. Source data are available online for this figure.

To unveil which domain of HSPA8 interacts with SPATA6, we co-expressed SPATA6 full-length proteins and HSPA8 truncated domain proteins in HEK293T cells. The co-IP and subsequent WB analysis revealed that SPATA6 could not interact with HSPA8 protein without the SBD domain, suggesting that HSPA8 interacts with SPATA6 via its substrate binding domain (SBD) (Fig. 8A). Because the substrate protein folding of HSPA8 is achieved through the binding and release of ATP (Buxbaum and Woodman, 1996), and it was shown that when ATP is bound to the nucleotide-binding domain (NBD) of HSPA8, the binding ability of the SBD domain to the substrate will be weakened (Arakawa et al, 2010; Bonam et al, 2019; Cyr and Ramos, 2023; Stricher et al, 2013), and when ATP is hydrolyzed to ADP, the affinity of the SBD domain for the substrate will be enhanced (Kityk et al, 2012). We, therefore, examined if the interaction ability between HSPA8 and SPATA6 was affected by adding ATP. The results showed that the binding capacity of HSPA8 to SPATA6 was reduced after treatment of the testis lysates with ATP, and the reduction was more pronounced at a high concentration of ATP (Fig. 8B,C), suggesting that SPATA6 is a substrate protein of HSPA8 for protein folding. In support of these findings, we performed in vitro experiments and found that both ATP and non-hydrolyzable ATP analog (AMP-PNP) could block HSPA8 binding to SPATA6 (Fig. 8D–G). Further experiments verified the disruption of NBD decreased the binding ability of SBD with SPATA6 after transfection of an NBD mutant (Fig. 8H,I). All the above results showed that the NBD domain of HSPA8 is required for regulating the affinity of the SBD domain with its substrates (e.g., SPATA6) (Fig. 8B–I). Interestingly, we found that the interaction of BAG5 with SPATA6 was also weakened after treatment with ATP in the samples (Fig. 8J,K). Since there is no evidence that the addition of ATP disrupts the binding ability of BAG5, combined with the above results, we speculated that the interaction between BAG5 and SPATA6 is indirect via HSPA8. To test this hypothesis, we constructed and transfected the pCAG-Strep-BAG5, pCAG-HSPA8, and pCAG-SPATA6 plasmids into HEK293F cells, and purified the protein complex through Strep-tag affinity chromatography. Coomassie brilliant blue staining of the purified protein shows two bands at 55 kDa (containing Strep-BAG5 and SPATA6) and 70 kDa (containing HSPA8) (Fig. EV5A). We then performed mass spectrometry analysis to confirm that the Strep-tag successfully pulled down BAG5, HSPA8, and SPATA6 (Fig. EV5B–E; Dataset EV5), and that BAG5, HSPA8, and SPATA6 could form a functional complex. Taken together, these findings indicate that BAG5 interacts with HSPA8 and regulates the affinity of HSPA8 to its substrates, e.g., SPATA6.

## Discussion

Previously, we reported that SPATA6 interacts with the myosin protein MYL6, a motor protein along microfilaments accounting for the protein transport in sperm, and loss of SPATA6 results in impaired development of the sperm head-to-tail coupling apparatus (HTCA) (Yuan et al, 2015). Here, we identified a molecular co-chaperone protein BAG5, which interacts with SPATA6 and expresses in steps 9–16 spermatids essential for sperm HTCA assembly. Inspiringly, the knockout of *Bag5* in mice results in the disorganization of HTCA and the disruption of the “9 + 2” microtubule, similar to the phenotype of SPATA6-deficient mice (Yuan et al, 2015). At the molecular level, we further showed that BAG5 might form a complex with HSPA8 to modulate the protein folding of SPATA6, MYO5A, and DYNLT1, thus affecting the assembly of sperm HTCA (Fig. 8L).

In the current study, we identified BAG5 as a co-chaperon protein that could interact with HSPA8 to regulate chaperone-mediated protein folding, and dysfunction of BAG5 would render the protein susceptible to misfolding during HTCA assembly, which expanded our understanding of sperm protein trafficking in HTCA assembly. Through proteomics analysis in WT and *Bag5* KO testes, we confirmed that insufficient protein folding activity reduces functional soluble protein in *Bag5* KO testes, especially under heat shock-induced stress conditions (Fig. 5D,E). In addition, the previous study has reported that *Bag5* can modulate GRP78 protein stability and reduce endoplasmic reticulum (ER) stress in cardiomyocytes (Gupta et al, 2016), further supporting our conclusion that *Bag5*-deficient sperm fail to properly fold proteins to cope with heat stress.

In 2018, Shang et al, reported that SUN5 is required for the integrity of the neck junction fragment, and that HSP40 may directly bind to SUN5 and may facilitate its protein folding (Shang et al, 2018). However, mutation of *HSP40* in humans does not lead to headless sperm but instead results in abnormal flagellar formation (El Khouri et al, 2016). Therefore, the relationship between HSP40 and SUN5 remains to be investigated. Existing studies have yielded little elucidation about the functional roles of protein folding in HTCA assembly. We have shown for the first time that BAG5 is involved in the heat shock protein network by IP-MS analysis and found that SPATA6, MYO5A, and DYNLT1 fail to bind to HSPA8 in the absence of BAG5.

During the protein folding process, co-chaperones work efficiently in coordination with molecular chaperones. In our study, BAG5 may act as a scaffold for coupling the HSPA8 chaperone complex to regulate the protein folding of SPATA6, MYO5A, and DYNLT1. These proteins play a critical role in spermatogenesis. For example, SPATA6 has been identified as an HTCA-associated protein. Myosin proteins [e.g., MYO5A (Hayasaka et al, 2008) and MYL6 (Yuan et al, 2015)] along microfilaments and dynein proteins [e.g., DNAL1 (Horváth et al, 2005), DYNLT1 (Indu et al, 2015), and DCTN1 (Zheng et al, 2011)] along microtubules acted as motor proteins to transport proteins in the sperm (Kierszenbaum et al, 2003; Teves et al, 2020). Therefore, the disassembly of the HTCA apparatus in *Bag5*-deficient mice may be due to the reduced protein-folding capacity of the HSPA8 chaperone complexes, and in turn, leading to reduced functional proteins and aggregation of unfolded proteins that are directly or indirectly essential for HTCA assembly.

Interestingly, we found that SPATA6 can interact with the SBD domain of HSPA8 (Fig. 8A), suggesting that SPATA6 is indeed a substrate protein of HSPA8 for protein folding. Notably, we found that the interaction between BAG5 and SPATA6 was attenuated by the addition of ATP and AMP-PNP both in vivo and in vitro (Fig. 8B–G). Since there was no evidence that ATP could disrupt the binding ability of BAG5, we concluded that BAG5 may interact with SPATA6 indirectly through HSPA8. In the future, the molecular links of protein structural specifications and complex formation among BAG5, HSPA8, and SPATA6 need to be investigated by cryoelectron microscopy analyses.

To date, several proteins have been reported to be required for the proper development or function of HTCA (Sha et al, 2018; Zhang et al, 2021). The deletion of either PMFBP1, SUN5, or CENTLEIN leads to the separation of sperm head from tail, resulting in ASS (Shang et al, 2017; Zhang et al, 2021; Zhu et al, 2018). Noteworthy, zhang et al pointed out that SUN5, PMFBP1, and CENTLEIN form a “sandwich” like structure at the sperm neck junction, connecting the basal plate and the fossa of the sperm (Zhang et al, 2021). In PMFBP1-deficient mouse sperm, the SUN5 signal remained in the sperm head (Zhu et al, 2018), which suggests that SUN5 may be localized in the basal fossa of the nuclear membrane, and the extended part connects to the basal plate. Interestingly, in our study, we found that SUN5 and PMFBP1 remain attached to the head, while SPATA6 localized in the detached tail after BAG5 deletion (Fig. EV3A–C). This observation is consistent with our transmission electron microscopy (TEM) results that Bp (basal plate) is attached to the head of *Bag5* KO sperm while Sc (segmented column) and Cp (capitulum) are detached from sperm (Fig. 4C). Moreover, in the absence of SUN5 and PMFBP1 protein, although the Bp fails to anchor to the basal fossa or nuclear membrane, the structure of HTCA was intact and well-ordered (Shang et al, 2017; Zhu et al, 2018). Interestingly, the assembly of HTCA was disrupted in *Centlein* KO mice (Zhang et al, 2021), similar to the disorganized HTCA of *Bag5* KO mice. These results provide an important reference for an in-depth understanding of the mechanism of sperm head-tail separation (such as the anchoring function and integrity of Sc and Cp).

In addition to the disorganization of HTCA, we observed the occurrence of abnormal cytoplasm invagination in *Bag5* KO mice. During sperm differentiation, cytoplasmic development and removal is a complex biological process that requires precise biological regulation (Shimada and Ikawa, 2023; Shimada et al, 2023). Therefore, it was very fascinating and worthy to analyze in detail the association between loss of BAG5 and cytoplasmic behavior (invagination and removal) and explore the biological mechanism causing the abnormal cytoplasm invagination in future studies. In *Bag5* KO mice, the headless sperm also manifest flagella defects, including disordered mitochondrial sheath arrangement and 9 + 2 microtubule (Fig. 3H), consistent with the phenotype of *Spata6*, *Sun5*, *Centlein*, and *Pmfbp1* KO sperm (Shang et al, 2017; Yuan et al, 2015; Zhang et al, 2021; Zhu et al, 2018). In mammals, the flagellum is attached to the implantation fossa of the head by the HTCA, which is essential for sperm motility and subsequent fertilization (Blanco et al, 2023; Kubo et al, 2021). In our study, sperm motility decreased significantly due to the defection of the flagellum in the absence of BAG5 (Fig. 3D). Interestingly, centrosome proteins regulated the assembly of HTCA and flagella (Hall et al, 2013; Zhang et al, 2021). Considering that ODF1 localized in the flagellum and that loss of the small heat shock protein ODF1/HSPB10 not only results in abnormal flagella but also affects HTCA assembly (Hetherington et al, 2016; Yang et al, 2014; Yang et al, 2012), we thus proposed that sperm HTCA assembly and flagellum development may be interrelated. In recent years, flagellar development, especially the “9 + 2” microtubule structure, has been well studied. However, there is a lack of research on HTCA assembly. Therefore, the structural analysis of HTCA in spermatozoa deserves further research in the future.

The assembly of HTCA is a complex process that requires precise protein transport (Yuan et al, 2015). Manchette occurs in the late stage of spermiogenesis and is indispensable for protein trafficking to the neck and flagella (Zhang et al, 2022a). SPATA6 (Yuan et al, 2015), CCDC42 (Tapia Contreras and Hoyer-Fender, 2019), CNTROB (Liska et al, 2009), FAM46C (Zheng et al, 2019), HOOK1 (Chen et al, 2018), and IFT88 (Kierszenbaum et al, 2011) proteins have been reported to be located in the manchette structure, and the absence of these proteins will lead to headless sperm in mice. Although these studies have reported the importance of these manchette-located proteins in the head-tail linkage, the direct molecular regulatory mechanisms involved in maintaining the integrity of the sperm head-tail junction remain unclear. Here, our study provides critical possible mechanisms for the role of the manchette structure in trafficking sperm HTCA proteins and stabilizing sperm head-tail attachment (Fig. 8L) and demonstrates that the manchette structure plays a vital role in regulating sperm HTCA assembly and maintaining its integrity. Of note, we observed that BAG5 translocated from the manchette in steps 9–14 spermatids to the basal plate in steps 15–16 in the current study, which is consistent with SPATA6 and CCDC42. In addition, BAG5 was localized in the basal plate and regulated protein transport to assemble the HTCA, such as the capitulum (Cp) and segmented column (Sc), which further supported that protein transport is indispensable for the assembly of HTCA.

In conclusion, we demonstrated that BAG5 served as a co-chaperone of HSPA8 to modulate the protein folding and proteostasis involved in the assembly of the HTCA (Fig. 8L). The protein regulatory network of sperm HTCA assembly identified in this study unveils a new genetic and molecular insight into the production of acephalic spermatozoa, which may provide a promising strategy for the future diagnosis and even treatment of male infertility. However, what we know about the potential mechanism of HTCA assembly is only the tip of the iceberg. Further studies are needed to unravel the mechanisms by which protein folding and cargo transport are involved in the assembly of sperm HTCA.

## Methods

### Ethics statement

All animal work was conducted under the guidelines of the Institutional Animal Care and Use Committee (IACUC) of Tongji Medical College, Huazhong University of Science and Technology, China. All mice were housed in the same pathogen-free animal facility of the Laboratory of Animal Center, Huazhong University of Science and Technology. All animal procedures were approved by the Ethics Committee for the Welfare of Experimental Animals of Huazhong University of Science and Technology (approval reference number: S2795).

### Generation of *Bag5* knockout mice

The *Bag5* knockout (KO) mice were generated by applying the CRISPR/Cas9-mediated genome editing technology. The T7 promoter and the guiding sequence, targeting exon 2 of the *Bag5* gene with two pairs of single-guide RNAs (sgRNAs), were used. The PCR amplification for sgRNAs using the following primers: BAG5-sgRNA1-Forward: 5′- TAGGTCCTGAAGCCTACTAATGGA-3′ and BAG5- sgRNA1-Reverse: 5′-AAACTCCATTAGTAGGCTTCAGGA-3′. BAG5-sgRNA2-Forward: 5′- TAGGTGACGCACGTGAAGACCGGA-3′ and BAG5- sgRNA2-Reverse: 5′- AAACTCCGGTCTTCACGTGCGTCA-3′. Cas9 mRNA (20 ng) and sgRNA (10 ng) were mixed and injected into C57BL/6 zygotes, then implanted into pseudo-pregnant C57BL/6 females. Genomic DNA was extracted from founder mice, followed by Sanger sequencing confirmation and PCR analysis. The founder heterozygous mice were backcrossed to wild-type (WT) C57BL/6 mice to obtain F1 heterozygous mice, and F2 generation mice were genotyped and intercrossed to generate *Bag5* KO mice for experiments. The primers used for genotyping are listed in Table EV1.

### Epididymal sperm analysis

The caudal epididymis was dissected from 8-week-old mice. To release sperm, mouse caudal epididymis was minced and incubated with pre-warmed PBS for 10–20 min at 37 °C. For sperm morphology, slides were stained with Hematoxylin-eosin staining (Servicebio, G1076) according to the manufacturer’s protocol. The percentages of tailless, headless, and intact sperm were quantified, and at least 200 spermatozoa were examined for each mouse.

### Histological analysis

The mouse testes, epididymides, and ovaries were dissected and fixed overnight in Bouin’s solution (Sigma, HT10132) at 4 °C. Following dehydration, the samples were embedded in paraffin and cut into 5-µm-thick paraffin sections. After deparaffinization and rehydration, the tissue sections were stained with hematoxylin and eosin (H&E) or periodic acid-Schiff (PAS) using manufacturers’ protocols and photographed with a light microscope (Axio Scope.A1, Zeiss, Germany).

### Electron microscopy

For scanning electron microscopy (SEM), fresh sperm samples were collected and resuspended in an electron microscopy fixative (Servicebio, G1102), fixed for 2 h at room temperature (RT), and transferred to 4 °C for 8 h. Sperm samples were blocked with 1% OsO4 in 0.1 M PBS (pH = 7.4) for 1.5 h at RT. After being dehydrated in a series of ethanol dilutions at increasing concentrations and in isoamyl acetate (Sinopharm, 10003128) for 15 min, the samples were dried with a critical point dryer (Quorum, K850). Then the samples were coated with gold particles and visualized with an S-3400N scanning electron microscope (Hitachi, Tokyo, Japan). For transmission electron microscopy (TEM), the adult mouse testes were cut into small pieces and fixed in 0.1 M cacodylate buffer (pH = 7.4) containing 2.5% glutaraldehyde and 3% paraformaldehyde overnight. After washing in 0.1 M cacodylate buffer three times, samples were immersed in 1% OsO4 for 1 h at 4 °C. Ultrathin sections (60–70 nm thickness) were counterstained with uranyl acetate and lead citrate and examined with a JEM-1400 transmission electron microscope (JEOL). For immunogold-based electron microscopy (Ig-EM), the mouse testis smear samples were fixed in 0.1 M cacodylate buffer (pH = 7.4) containing 2.5% glutaraldehyde and 3% paraformaldehyde for 8 h at 4 °C, washed overnight in 0.1 M PBS, dehydrated in graded ethanol, then embedded in acrylic resin, and polymerized at 53 °C for 24 h. Ultrathin sections (60–70 nm thickness) were immunostained overnight at 4 °C with the rabbit anti-BAG5 antibody (Sigma, dilution of 1:100), followed by incubation for 1 h with 10 nm colloidal gold-conjugated goat anti-rabbit IgG. Immunostained sections were photographed on a JEM-1400 transmission electron microscope (JEOL).

### Immunofluorescence

Freshly isolated testes were fixed with 4% paraformaldehyde (PFA) solution (Sigma, P6148) for 4–6 h at 4 °C. After treatment with a sucrose gradient, the testes were embedded in OCT compound (Sakura Finetek, 4583) and sectioned at 5-μm thickness. For immunofluorescence staining, the cryosections were microwaved in 0.01 M sodium citrate buffer (pH = 6.0) for 15 min to retrieve the antigen. The slides were then permeabilized with 0.3% Triton X-100 for 30 min and blocked in 5% bovine serum albumin (BSA, AP0027, Amresco) in PBS at RT for 1 h. The cryosections were incubated with primary and secondary antibodies, mounted with ProLong Gold (P36931, Invitrogen) containing DAPI, and photographed using a laser scan confocal microscope (LSM900, Zeiss, Germany). Details of primary and secondary antibodies used in this study are shown in Table EV2.

### Quantitative real-time PCR (qRT-PCR)

Total RNA was extracted from testes and cells using TRIzol reagent (Invitrogen, 15596-025) according to the manufacturer’s procedure. Then the total RNA was used to perform qRT-PCR after being mixed with corresponding primers and SYBR green master mix in the Step One Plus machine (Applied Biosystems, 4309155). The relative mRNA expression was calculated using the 2^−ΔΔCt^ method with GAPDH as an internal control. The primers used for PCR are listed in Table EV1.

### Immunoprecipitation (IP)

The testes or cells were lysed in ice-cold IP lysis buffer (20 mM HEPES, 150 mM NaCl, 0.5% NP-40, 1 mM DTT, pH = 7.3) supplemented with protease inhibitor cocktail (Beyotime, P1008). The lysates were clarified by centrifugation at 12,000 × *g* for 20 min at 4 °C, and the supernatants were incubated with relevant antibodies overnight on a shaker. Then, the supernatants were incubated with protein A/G-coated magnetic beads at 4 °C for 4 h. The beads were washed with lysis buffer and collected for subsequent Western blot or liquid chromatography-tandem mass spectrometry (LC-MS).

### Immunoblotting

The testis tissues or cells were rinsed with PBS and lysed in cold RIPA buffer (CWBIO, CW2333S). The extraction of endoplasmic reticulum (ER) proteins in the testis using the Kit (HR0249, bjbalb). The tissue lysates were centrifuged at 12,000 × *g* for 20 min at 4 °C, and the protein extracts were denatured with 5 × SDS loading buffer (Beyotime, P0015L) at 100 °C for 5–10 min followed by incubation on ice and separated by SDS-PAGE gel, and then transferred onto polyvinylidene difluoride membranes (IPVH00010, Millipore). The membranes were then blocked with TBST buffer (10 mM Tris–HCl pH = 7.4, 150 mM NaCl, and 0.1% Tween-20) containing 5% skimmed milk at RT for 2 h and stained with the primary antibody overnight at 4 °C and secondary antibodies for 1 h at RT. After the final washes with TBST buffer, the membranes were photographed using enhanced chemiluminescence detection (US Everbright Inc, IS0527). The antibodies used in this study are listed in Table EV2.

### Proteomics analysis

The protein of testis samples was extracted and digested with trypsin, followed by desalted using C18 Cartridge (Phenomenex) and vacuum-dried. Then, the protein peptides were reconstituted in 1% trifluoroacetic acid (TFA) and processed for tandem mass tag (TMT) kit/isobaric tags for relative and absolute quantitation (iTRAQ) kit. After TMT/iTRAQ labeling, the peptides were fractionated into fractions by high pH reverse-phase high-performance liquid chromatography (HPLC) with Thermo Betasil C18 column (5 μm particles, 10 mm ID, 250 mm length) and then analyzed by LC-tandem mass spectrometry (LC/MS). The LC/MS raw data were processed using Proteome Discoverer software (v.2.4, Thermo Scientific).

### Cell culture and plasmids construction generation

HEK293T and HeLa cells were obtained from the Stem Cell Bank of Chinese Academic Science and cultured in DMEM. HEK293F cells were purchased from the ATCC and cultured in the UNION-293 medium. All cultured mediums were supplemented with 10% (v/v) fetal bovine serum (FBS) and 1% penicillin–streptomycin at 37 °C with 5% CO_2_. Full-length mouse *Bag5, Hspa8, Sun5, Myo5a, Dynlt1, Pmfbp1, Centlein*, and *Spata6* cDNA were obtained from mouse testis cDNA. For Fig. 6G,H, full-length and truncated mutants of *Bag5* were cloned into the vectors pRK-FLAG, and full-length and truncated mutants of *Hspa8* were cloned into the vectors pCMV-MYC. For Figs. 5E and 7H, *Bag5* and *Spata6* were cloned into pCAG vector. For Fig. 8A, full-length and truncated mutants of mouse *Hspa8* cDNA were cloned into the vectors pCMV-MYC, and full-length *Spata6* cDNA were cloned into the vector pRK-FLAG. For Fig. 8D–K, full-length and truncated mutants of mouse *Bag5* and *Hspa8* cDNA were cloned into the vector pCAG-2 × STREP-FLAG, and *Spata6* cDNA was cloned into the vector pCAG. For Fig. EV3D–G, full-length *Sun5, Pmfbp1, Centlein*, and *Spata6* were cloned into the pCMV-MYC vector and *Bag5* was cloned into the pRK-FLAG vector. All construction of plasmids was performed using Clon Express Ultra One Step Cloning Kit (C115, Vazyme).

### Generation of *BAG5* knockout HeLa cell lines

CRISPR/Cas9-mediated gene knockout (KO) strategy was applied to generate the *BAG5* KO HeLa cells. Briefly, two specific sgRNAs of targeting the *BAG5* gene were cloned into the lenti-CRISPR vector. Then the lenti-CRISPR-sgRNA plasmids and package plasmids were co-transfected into HEK293T with Lipofectamine 2000 (Invitrogen) and added into the HeLa cell culture medium to infect the cells. Single-cell clones were picked up after selection with puromycin for 2 weeks and expanded for subsequent experiments. The primers of sgRNA are listed in Table EV1.

### Intracytoplasmic sperm injection (ICSI)

The ICSI was performed as described in our previous report (Yuan et al, 2015). Briefly, the intact sperm and sperm heads were collected from WT and *Bag5* KO mouse epididymis, respectively. For WT intact sperm, the spermatozoa were sonicated to dissociate sperm heads and tails. The single sperm head was picked up and injected into oocytes using a micromanipulator with a Piezo-electric actuating pipette. Injected oocytes were transferred to KSOM medium (MR-107-D, Millipore) under mineral oil and cultured at 37 °C with 5% CO_2_. The injected oocytes were assessed and photographed 5–8 h after ICSI. Two-cell embryos were transferred into oviducts of pseudo-pregnant female mice, and offspring were examined 20 days after embryo transfer.

### Statistical analysis

The data were shown as the mean ± SD/SEM. The statistical significance of the differences between the groups was measured by the two-sided Student’s *t*-test and two-tailed Mann–Whitney *U*-test using GraphPad Prism 8.0. Data were considered statistically significant when *P < 0.05, **P < 0.01, and ***P < 0.001.

## Supplementary information

Appendix

Table EV1

Table EV2

Dataset EV1

Dataset EV2

Dataset EV3

Dataset EV4

Dataset EV5

Movie EV1

Movie EV2

Appendix and EV Figures Source Data

Source Data Fig. 1

Source Data Fig. 2

Source Data Fig. 3

Source Data Fig. 4

Source Data Fig. 5

Source Data Fig. 6

Source Data Fig. 7

Source Data Fig. 8

Peer Review File

Expanded View Figures

## Data Availability

All the raw data of proteomics are available via ProteomeXchange (https://www.ebi.ac.uk/pride/archive) with the identifier PXD042965. Confocal microscopy images are available in the BioStudies database under accession number S-BSST1229.
