# Supplementary material for: BAG5 regulates HSPA8-mediated protein folding required for sperm head-tail coupling apparatus assembly
Source: EMBO Rep. 2024 Mar 7;25(4):23. doi: 10.1038/s44319-024-00112-x (PMC11015022; doi:10.1038/s44319-024-00112-x)
Supplement: Supplementary file 1 — Appendix [file 44319_2024_112_MOESM1_ESM.pdf]

Appendix File Table of Contents

Appendix Figure S1 Immunogold labeling followed by transmission electron microscopy (IG-TEM) on mouse testicular cells in KO mice.

Figure……………………………………………………………………………………2

Figure legend………………………………………………………………...……………2

Appendix Figure S2 Comparative analysis of homology-based on the amino acid sequence.

Figure……………………………………………………………………………………3

Figure legend…………………………………………………………………...…………3

Appendix Figure S3 Examination of sperm flagellum orientation in WT and KO mice testis.

Figure……………………………………………………………………………………4

Figure legend…………………………………………………………………...…………4

Appendix Figure S4 The occurrence of abnormal cytoplasm invagination after deletion of BAG5.

Figure……………………………………………………………………………………5

Figure legend………………………………………………………………...……………5

Appendix Figure S5 Loss of BAG5 results in protein misfolding.

Figure……………………………………………………………………………………6

Figure legend…………………………………………………………………...…………6

Appendix Figure S6 Loss of BAG5 does not affect the transcription of some myosin and dynein proteins.

Figure……………………………………………………………………………………7

Figure legend………………………………………………………………...……………7

Appendix Figure S1. Complement to Figure 1.

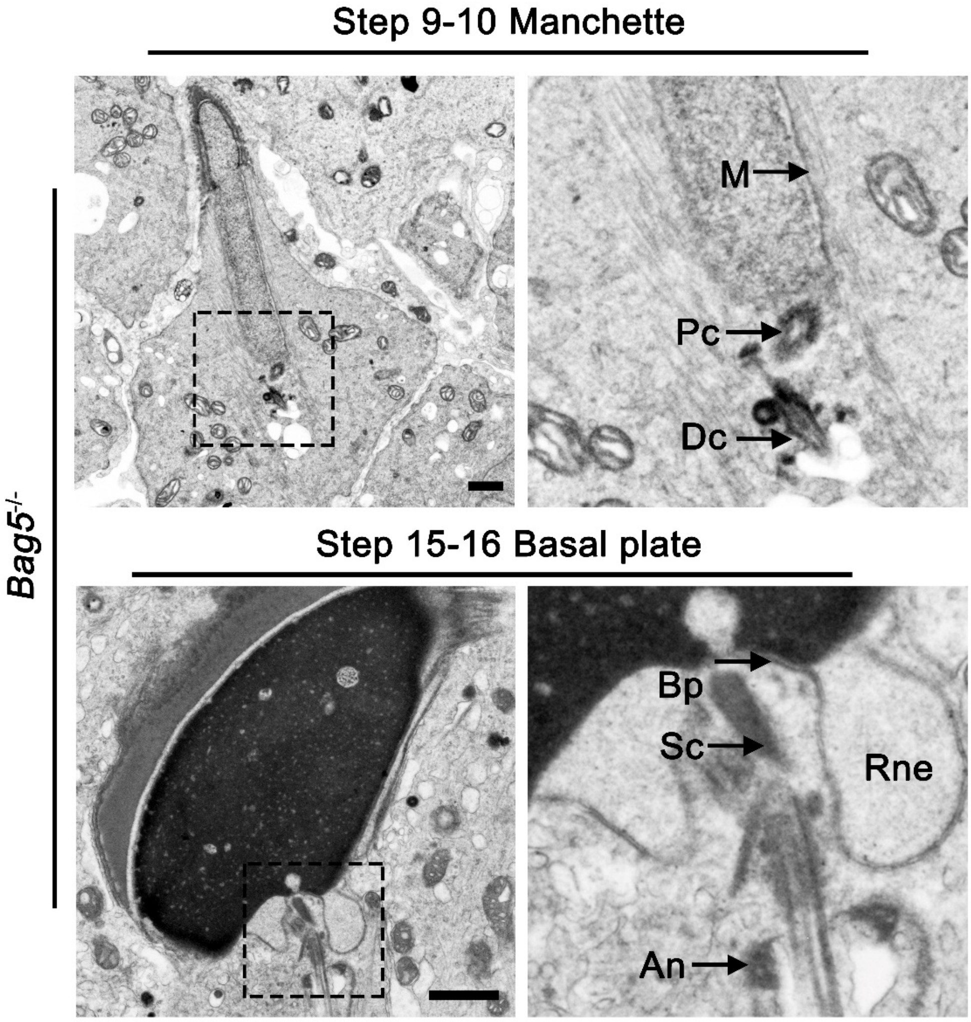

Appendix Figure S1 Immunogold labeling followed by transmission electron microscopy (IG-TEM) on mouse testicular cells in KO mice. No gold particles were detected in *Bag5*$^{-/-}$ sperm. In addition, the connecting piece was disrupted in *Bag5*$^{-/-}$ sperm. M: manchette; Bp: basal plate; Sc: segmented column; Pc: proximal centriole; Dc: distal centriole; Rne: redundant nuclear envelope; An: annulus. Scale bars = 1 μm.

Data information: Data represent results from three independent biological replicates experiments.

Source data are available online for this figure.

Appendix Figure S2. Complement to Figure 1.

	Cattle	Chimp-anzee	Dog	Horse	Mouse	Human	Rat	Pig	Monkey
Cattle		94.0%	94.6%	93.7%	90.8%	93.7%	90.2%	88.8%	94.0%
Chimpanzee	94.0%		96.4%	96.2%	91.7%	99.6%	91.3%	87.9%	99.3%
Dog	94.6%	96.4%		96.4%	92.8%	96.4%	92.2%	89.5%	96.0%
Horse	93.7%	96.2%	96.4%		91.3%	96.2%	89.9%	87.9%	95.5%
Mouse	90.8%	91.7%	92.8%	91.3%		91.3%	94.2%	85.2%	91.9%
Human	93.7%	99.6%	96.4%	96.2%	91.3%		90.8%	87.7%	98.9%
Rat	90.2%	91.3%	92.2%	89.9%	94.2%	90.8%		85.5%	91.5%
Pig	88.8%	87.9%	89.5%	87.9%	85.2%	87.7%	85.5%		88.1%
Monkey	94.0%	99.3%	96.0%	95.5%	91.9%	98.9%	91.5%	88.1%	

Appendix Figure S2 Comparative analysis of homology-based on the amino acid sequence. Multi-alignment analyses of BAG5 orthologs in nine vertebrate species.

Appendix Figure S3. Complement to Figure 4.

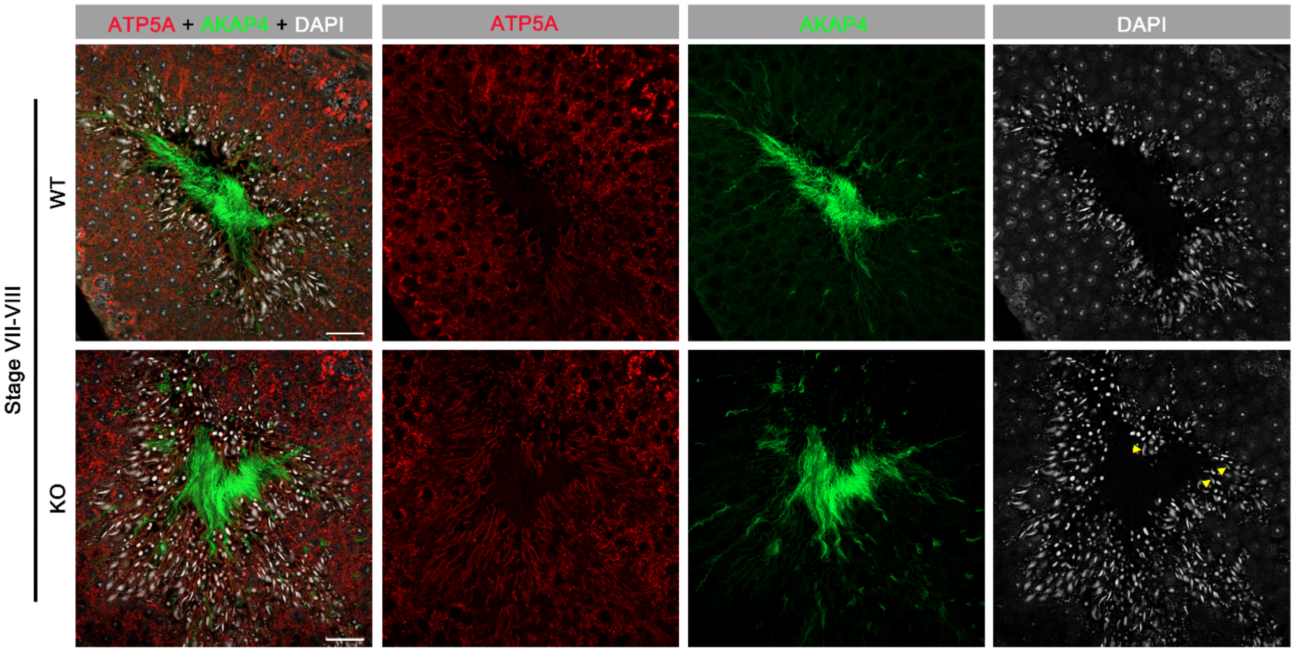

Appendix Figure S3 Examination of sperm flagellum orientation in WT and KO mice testis. Representative images of ATP5A (red) and AKAP4 (green) immunostaining on WT and KO testis sections are shown. The sperm flagellum orientation was arranged relatively normally in *Bag5* KO mice. ATP5A marks the mitochondria located in the midpiece. AKAP4 is located in the principal piece. The yellow arrowheads indicated the abnormal orientation of sperm head. Scale bars = 25 μm.

Data information: Data represent results from three independent biological replicates experiments.

Source data are available online for this figure.

Appendix Figure S4. Complement to Figure 4

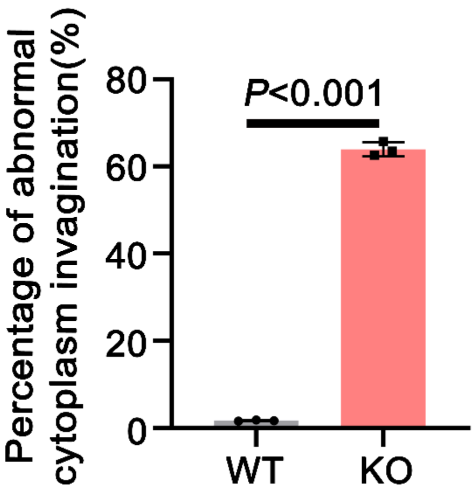

Appendix Figure S4 Complement to Figure 4. The occurrence of abnormal cytoplasm invagination after deletion of BAG5. The quantification indicated ~63% of abnormal cytoplasm invagination in *Bag5* KO. Data are presented as mean ± SD. *P* values (Student *t*-test, two-sided).

Data information: Data represent results from counting 30 spermatids per mouse and three independent biological replicates experiments.

Source data are available online for this figure.

Appendix Figure S5. Complement to Figure 5.

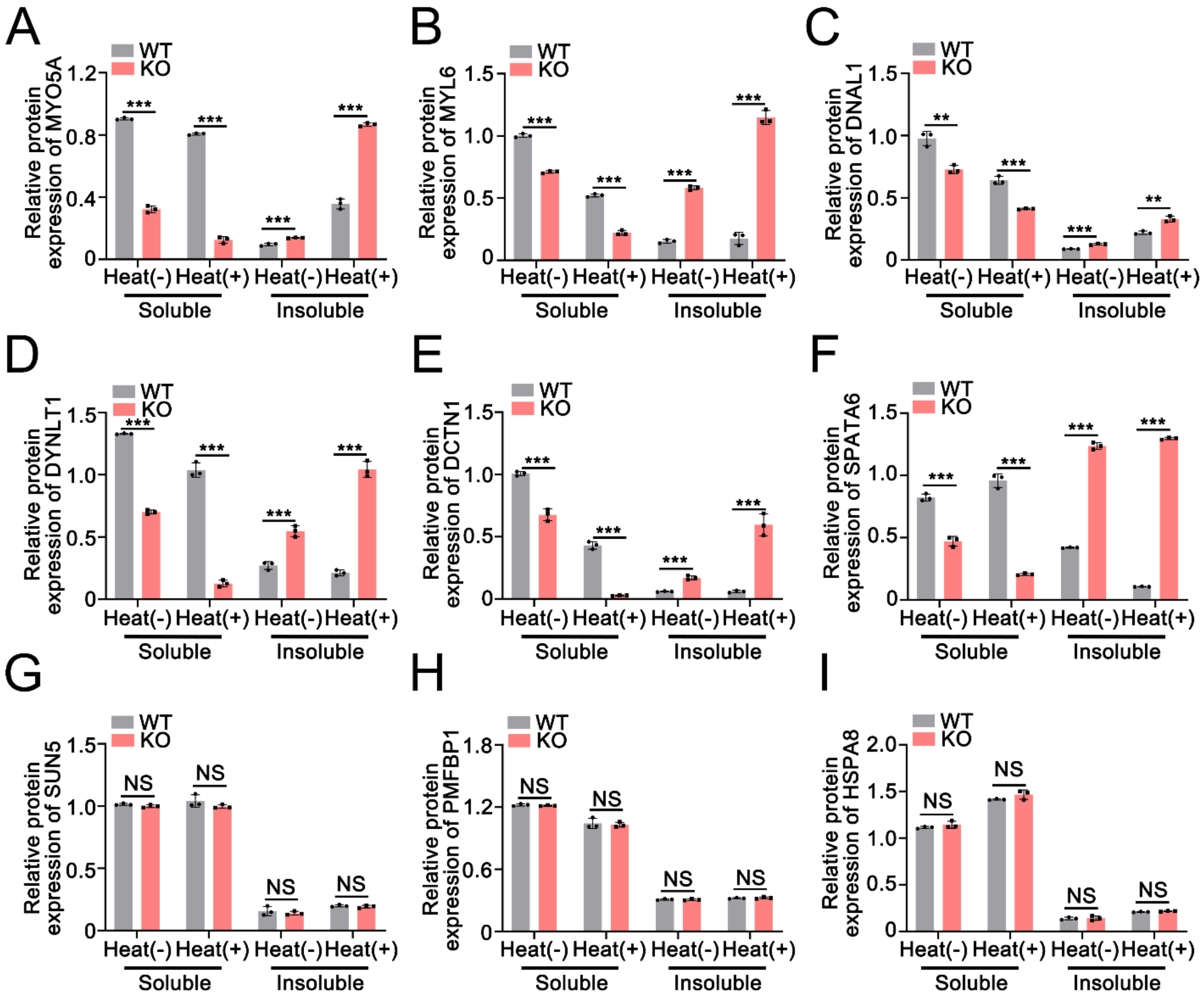

Appendix Figure S5 Complement to Figure 5. Loss of BAG5 results in protein misfolding.

A-I Quantifications of the protein expression levels in **Fig. 5D** are shown. Data are presented as mean ± SD. **P < 0.01, ***P < 0.001. NS, not significant. P values (Student t-test, two-sided).

Data information: Data in A-I represent results from three independent biological replicate experiments.

Source data are available online for this figure.

Appendix Figure S6. Complement to Figure 5

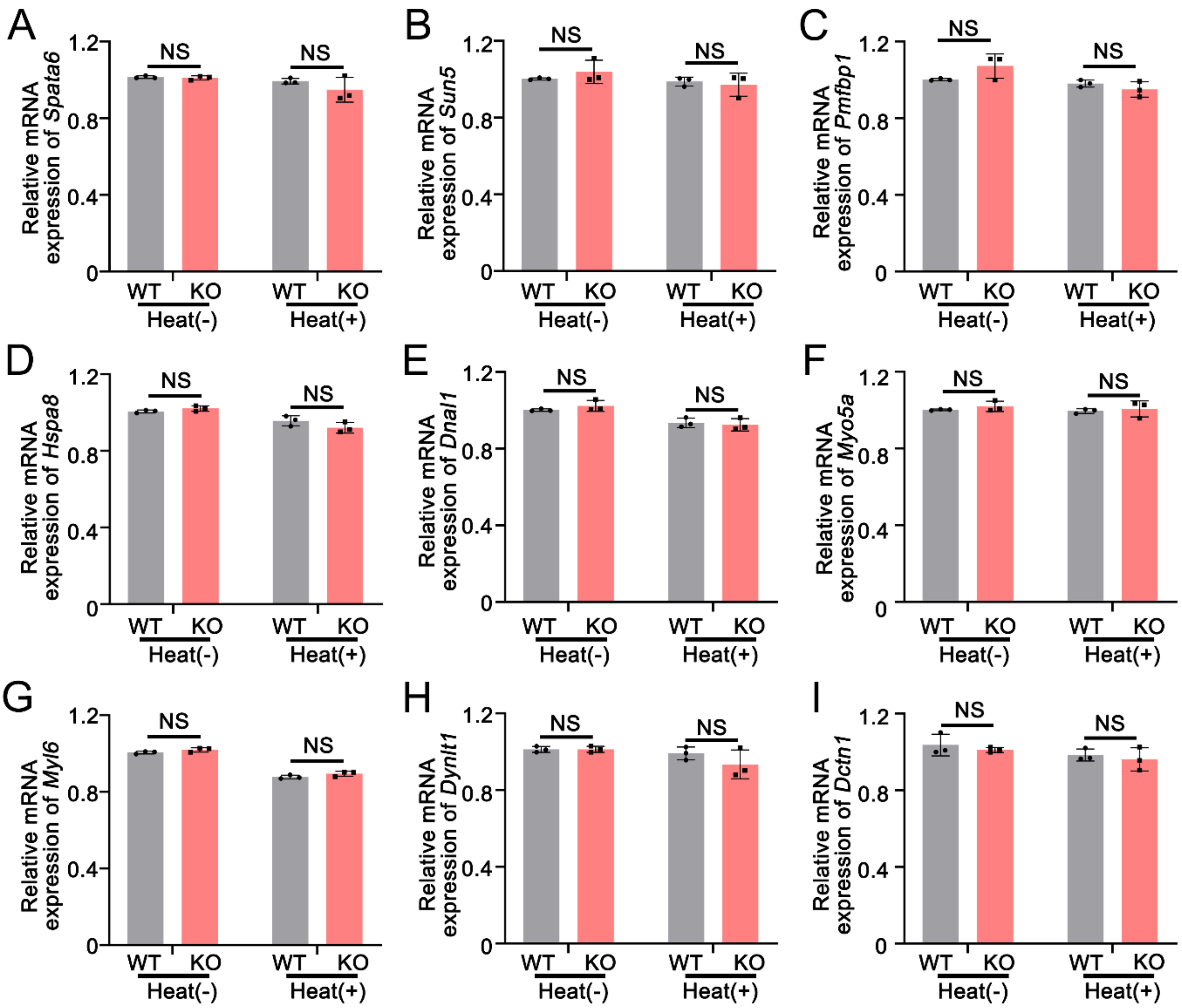

Appendix Figure S6 Complement to Figure 5. Loss of BAG5 does not affect the transcription of some myosin and dynein proteins.

A-I Histograms showing the mRNA expression level of *Spata6* (A), *Sun5* (B), *Pmfbp1* (C), *Hspa8* (D), *Dnal1* (E), *Myo5a* (F), *Myl6* (G), *Dynlt1* (H), *and Dctn1* (I) in **Fig. 5D**. Data are presented as mean ± SD. NS, not significant. *P* values (Student *t*-test, two-sided).

Data information: Data in A-I represent results from three independent biological replicate experiments.

Source data are available online for this figure.
