# Supplementary figures and images for: BAG5 regulates HSPA8-mediated protein folding required for sperm head-tail coupling apparatus assembly (part 1 of 2)
Source: EMBO Rep. 2024 Mar 7;25(4):23. doi: 10.1038/s44319-024-00112-x (PMC11015022; doi:10.1038/s44319-024-00112-x)

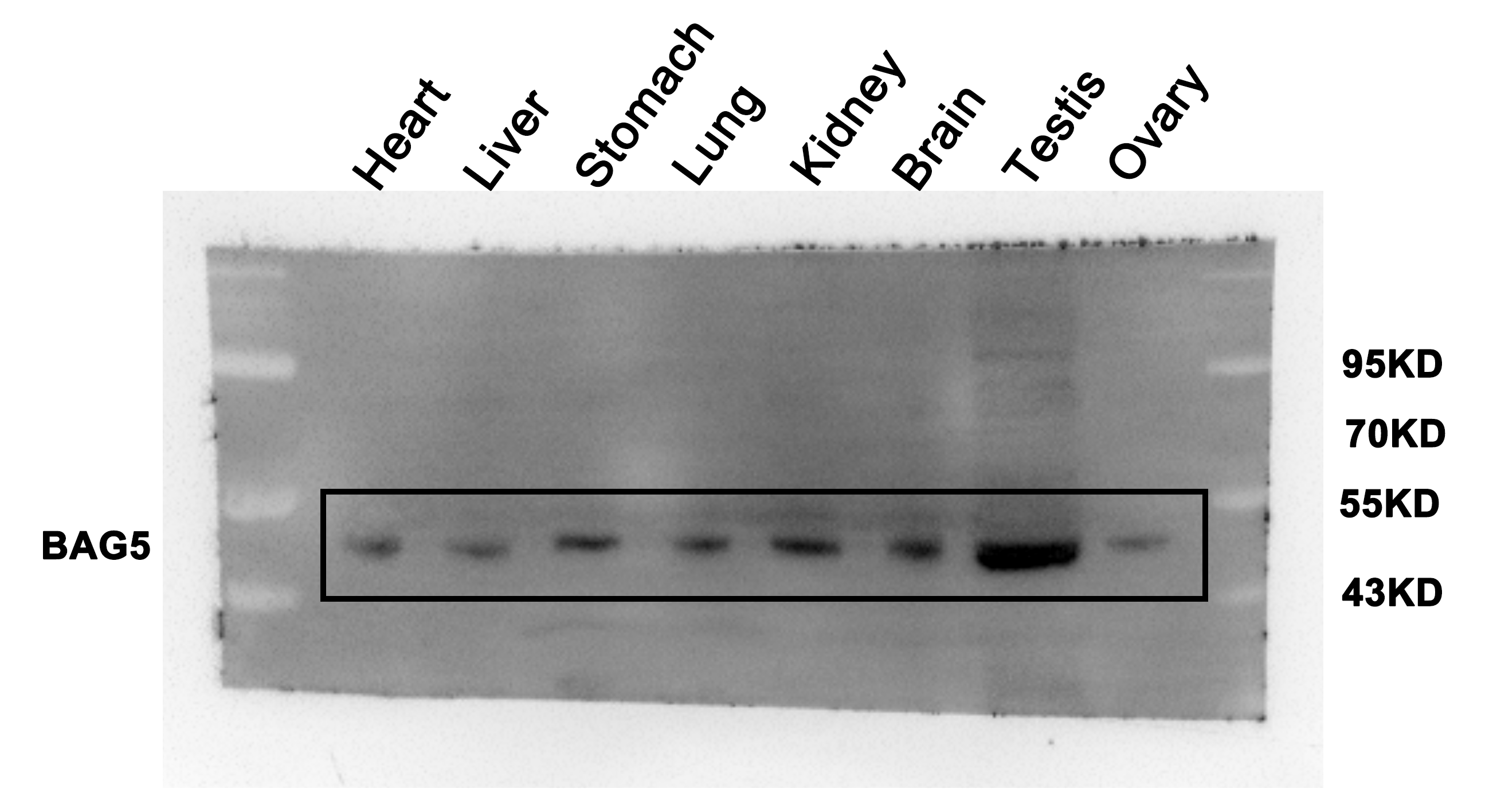

Supplement: Supplementary file 11 — Appendix and EV Figures Source Data [file 44319_2024_112_MOESM11_ESM.zip › Figure EV1-EV5/Figure EV1/1C/WB BAG5.tif]

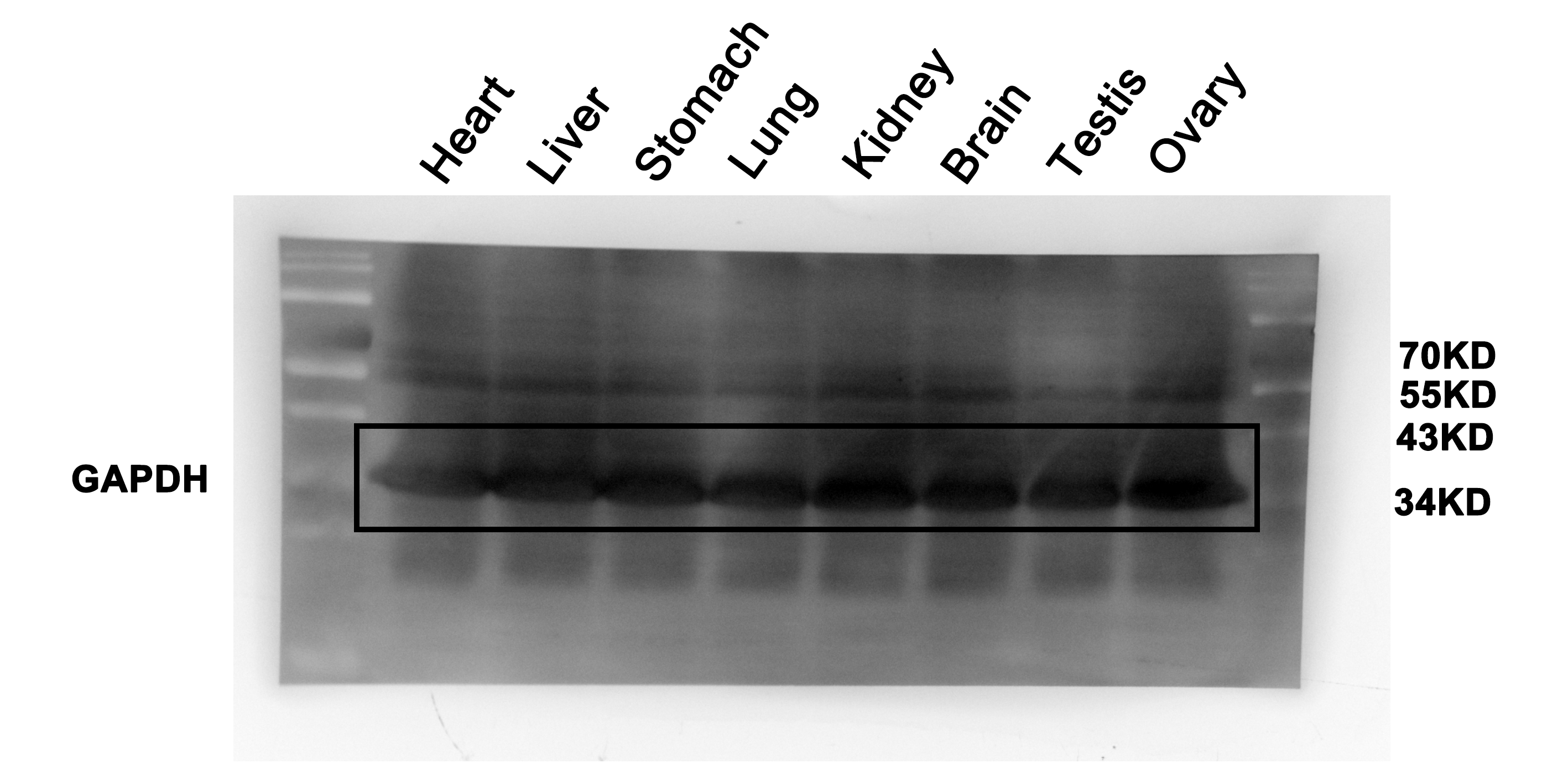

Supplement: Supplementary file 11 — Appendix and EV Figures Source Data [file 44319_2024_112_MOESM11_ESM.zip › Figure EV1-EV5/Figure EV1/1C/WB GAPDH.tif]

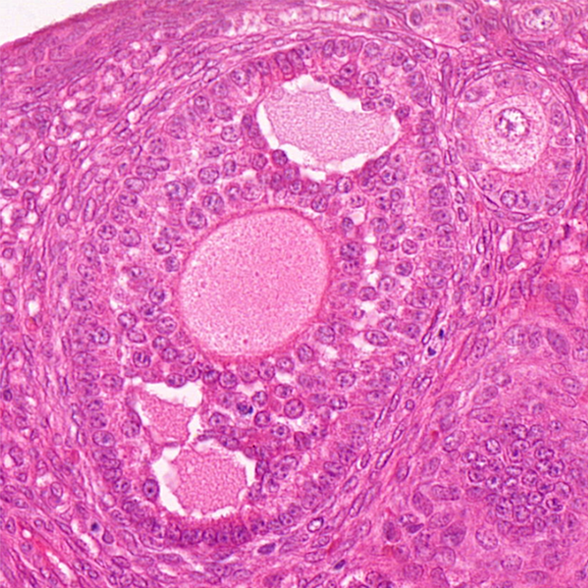

Supplement: Supplementary file 11 — Appendix and EV Figures Source Data [file 44319_2024_112_MOESM11_ESM.zip › Figure EV1-EV5/Figure EV2/2A/KO/ANTRAL.tif]

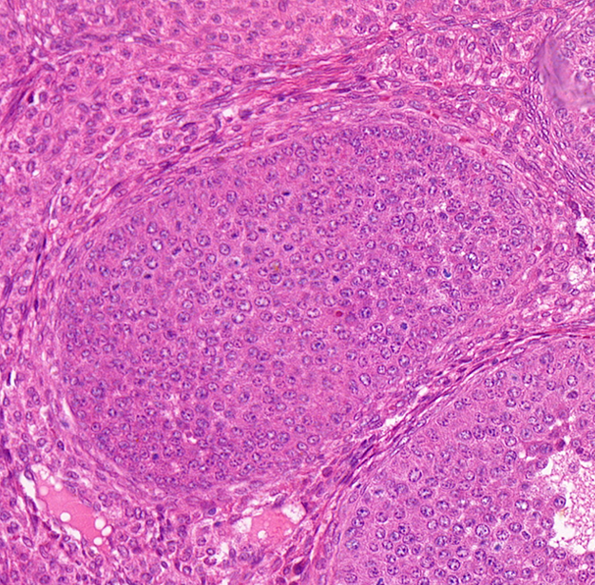

Supplement: Supplementary file 11 — Appendix and EV Figures Source Data [file 44319_2024_112_MOESM11_ESM.zip › Figure EV1-EV5/Figure EV2/2A/KO/CORPUS LUTEUM.tif]

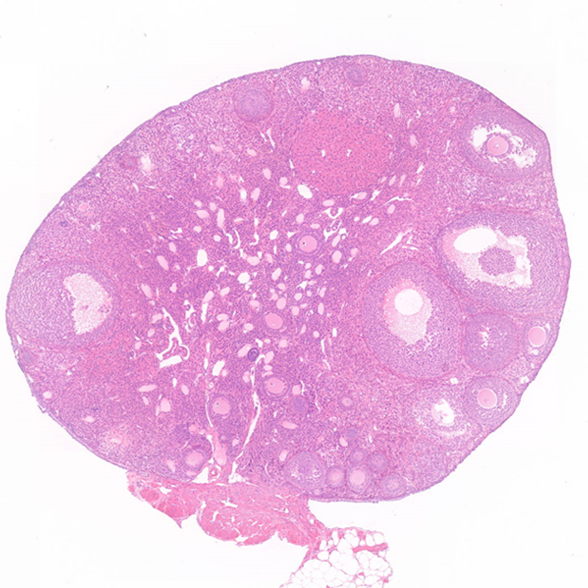

Supplement: Supplementary file 11 — Appendix and EV Figures Source Data [file 44319_2024_112_MOESM11_ESM.zip › Figure EV1-EV5/Figure EV2/2A/KO/OVARY.tif]

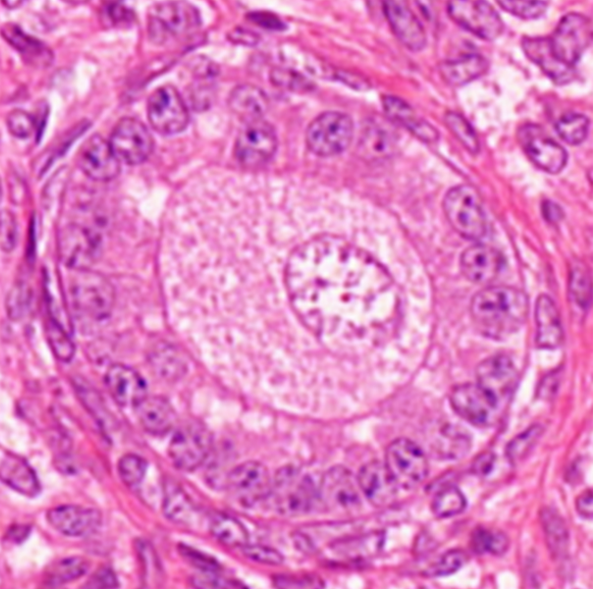

Supplement: Supplementary file 11 — Appendix and EV Figures Source Data [file 44319_2024_112_MOESM11_ESM.zip › Figure EV1-EV5/Figure EV2/2A/KO/PRIMARY.tif]

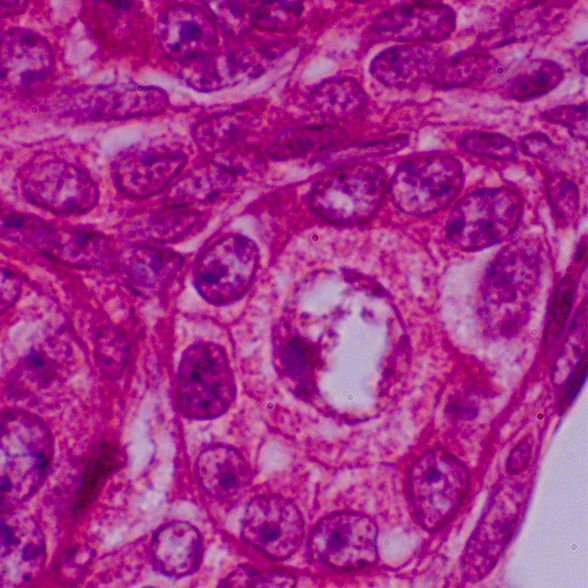

Supplement: Supplementary file 11 — Appendix and EV Figures Source Data [file 44319_2024_112_MOESM11_ESM.zip › Figure EV1-EV5/Figure EV2/2A/KO/PRIMORDIAL.tif]

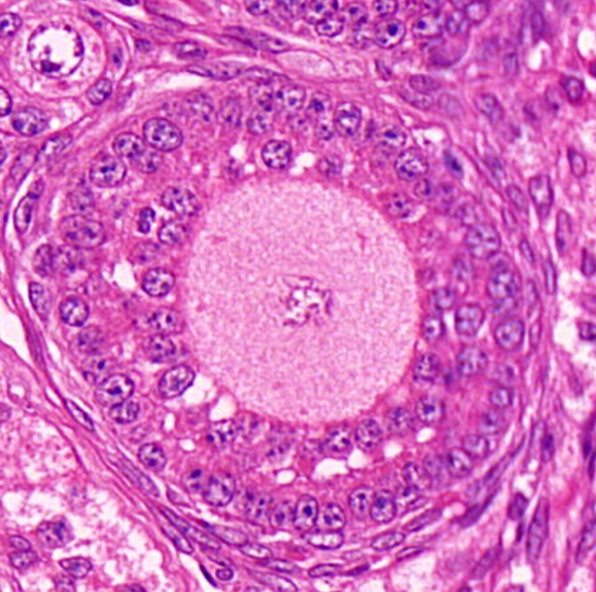

Supplement: Supplementary file 11 — Appendix and EV Figures Source Data [file 44319_2024_112_MOESM11_ESM.zip › Figure EV1-EV5/Figure EV2/2A/KO/SECONDARY.tif]

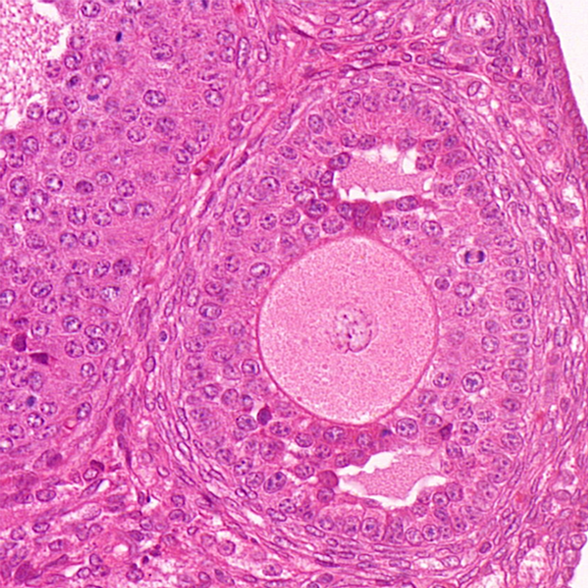

Supplement: Supplementary file 11 — Appendix and EV Figures Source Data [file 44319_2024_112_MOESM11_ESM.zip › Figure EV1-EV5/Figure EV2/2A/WT/ANTRAL.tif]

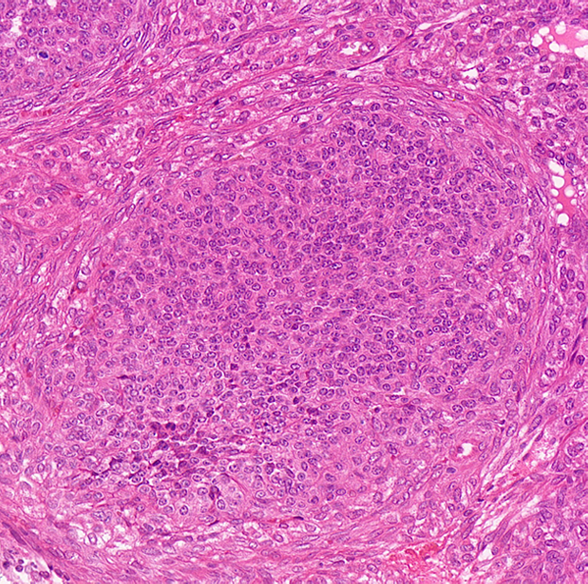

Supplement: Supplementary file 11 — Appendix and EV Figures Source Data [file 44319_2024_112_MOESM11_ESM.zip › Figure EV1-EV5/Figure EV2/2A/WT/CORPUS LUTEUM.tif]

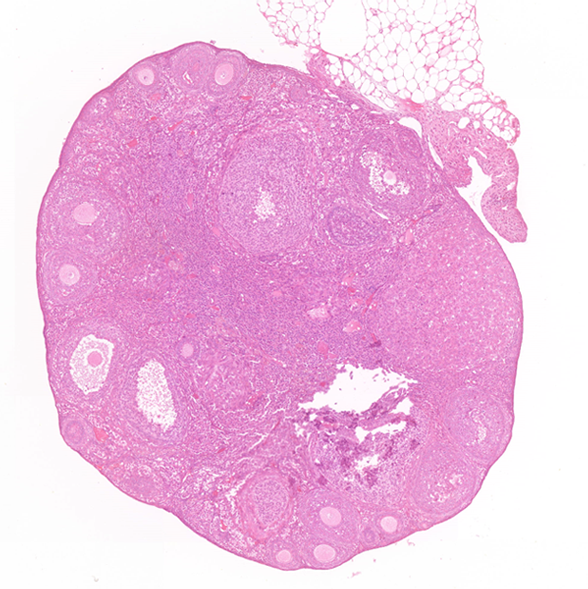

Supplement: Supplementary file 11 — Appendix and EV Figures Source Data [file 44319_2024_112_MOESM11_ESM.zip › Figure EV1-EV5/Figure EV2/2A/WT/OVARY.tif]

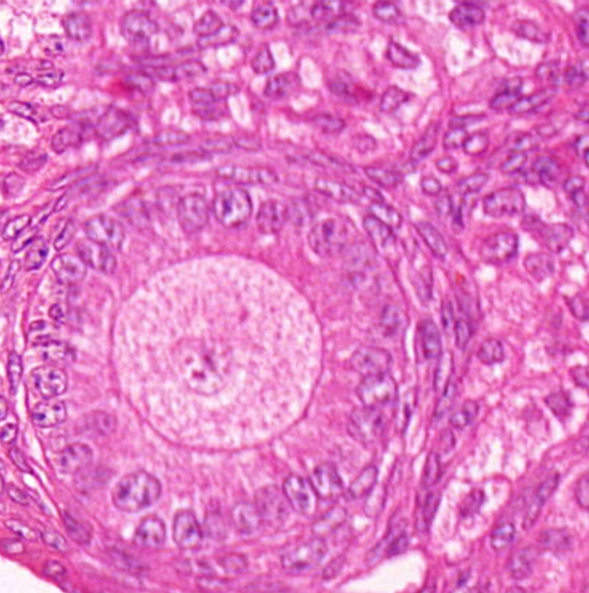

Supplement: Supplementary file 11 — Appendix and EV Figures Source Data [file 44319_2024_112_MOESM11_ESM.zip › Figure EV1-EV5/Figure EV2/2A/WT/PRIMARY.tif]

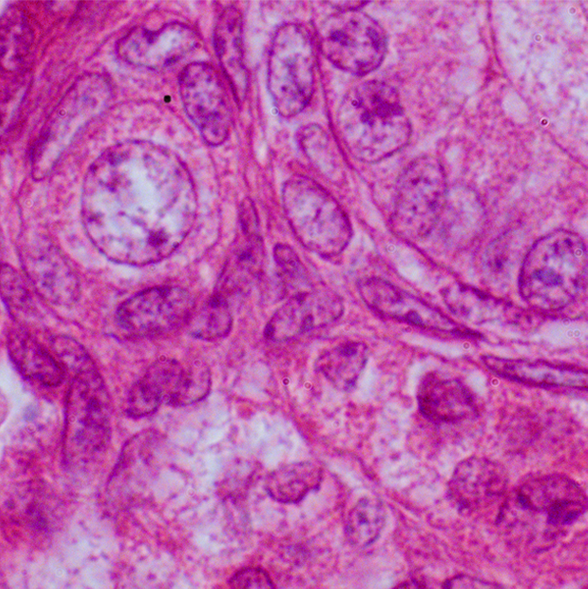

Supplement: Supplementary file 11 — Appendix and EV Figures Source Data [file 44319_2024_112_MOESM11_ESM.zip › Figure EV1-EV5/Figure EV2/2A/WT/PRIMORDIAL.tif]

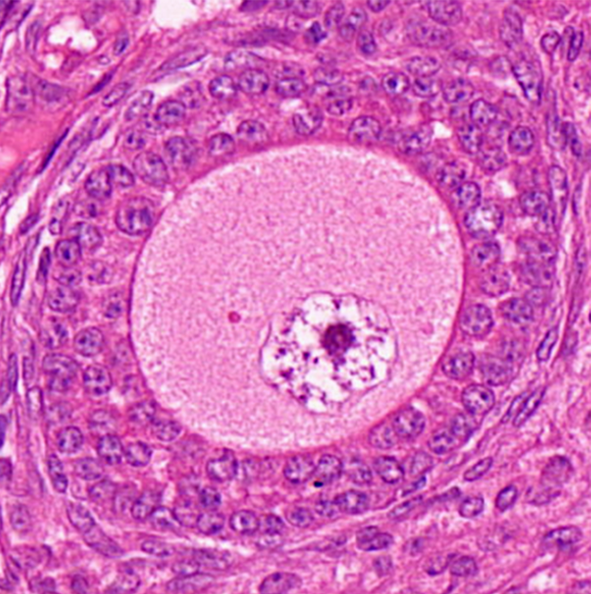

Supplement: Supplementary file 11 — Appendix and EV Figures Source Data [file 44319_2024_112_MOESM11_ESM.zip › Figure EV1-EV5/Figure EV2/2A/WT/SECONDARY.tif]

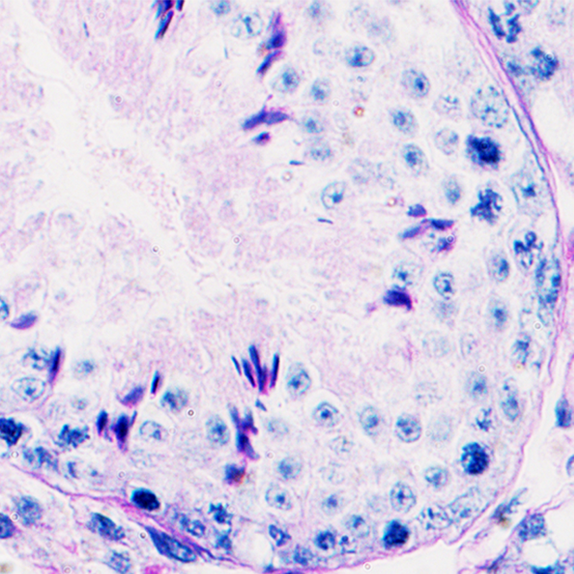

Supplement: Supplementary file 11 — Appendix and EV Figures Source Data [file 44319_2024_112_MOESM11_ESM.zip › Figure EV1-EV5/Figure EV2/2B/KO/I.tif]

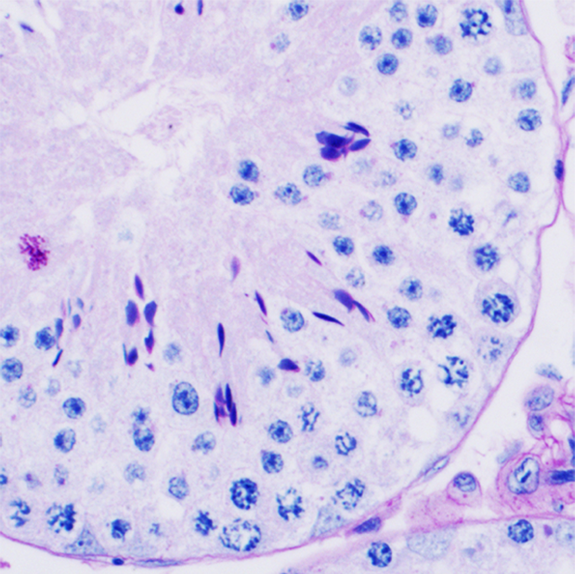

Supplement: Supplementary file 11 — Appendix and EV Figures Source Data [file 44319_2024_112_MOESM11_ESM.zip › Figure EV1-EV5/Figure EV2/2B/KO/II.tif]

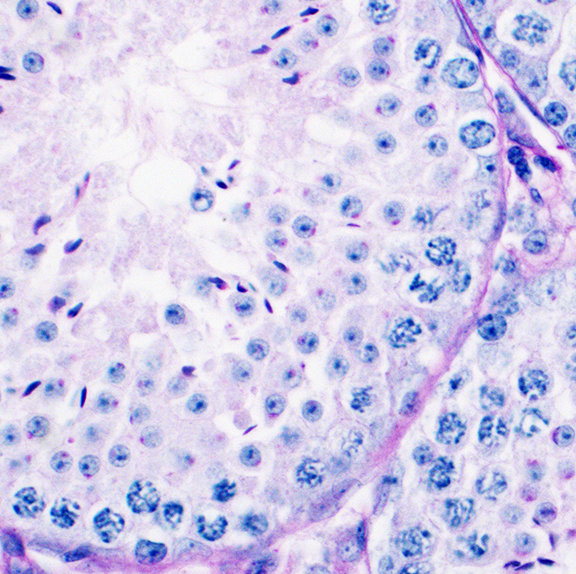

Supplement: Supplementary file 11 — Appendix and EV Figures Source Data [file 44319_2024_112_MOESM11_ESM.zip › Figure EV1-EV5/Figure EV2/2B/KO/III.tif]

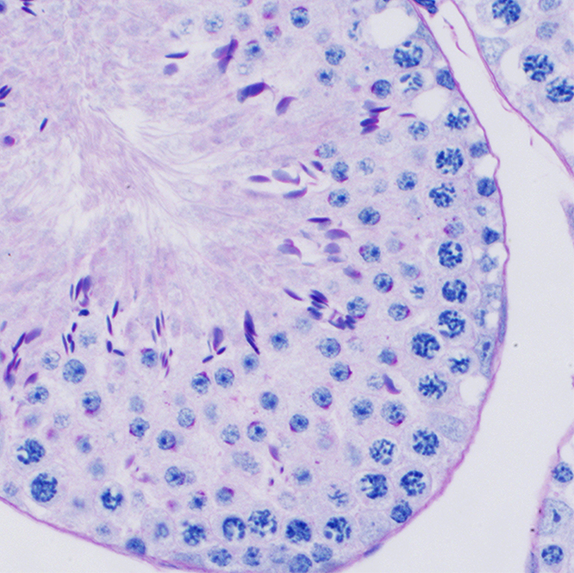

Supplement: Supplementary file 11 — Appendix and EV Figures Source Data [file 44319_2024_112_MOESM11_ESM.zip › Figure EV1-EV5/Figure EV2/2B/KO/IV.tif]

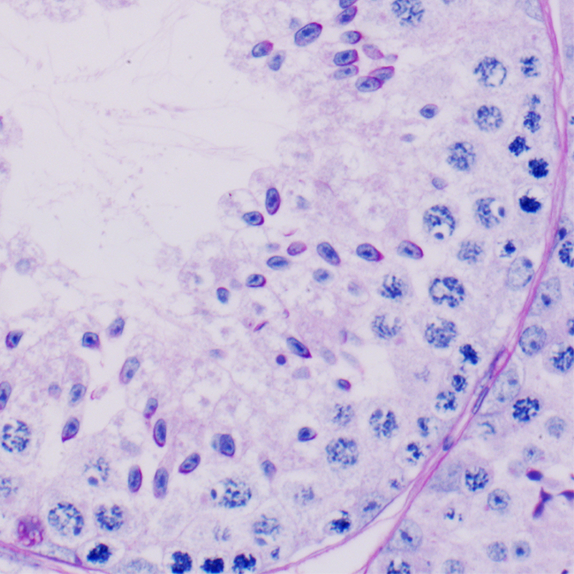

Supplement: Supplementary file 11 — Appendix and EV Figures Source Data [file 44319_2024_112_MOESM11_ESM.zip › Figure EV1-EV5/Figure EV2/2B/KO/IX.tif]

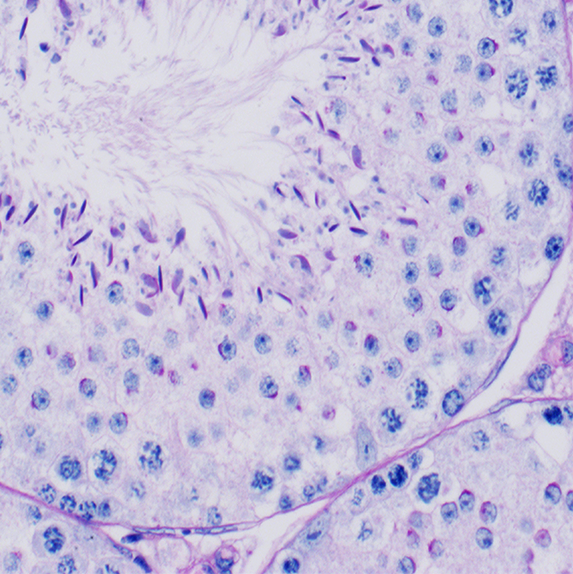

Supplement: Supplementary file 11 — Appendix and EV Figures Source Data [file 44319_2024_112_MOESM11_ESM.zip › Figure EV1-EV5/Figure EV2/2B/KO/V.tif]

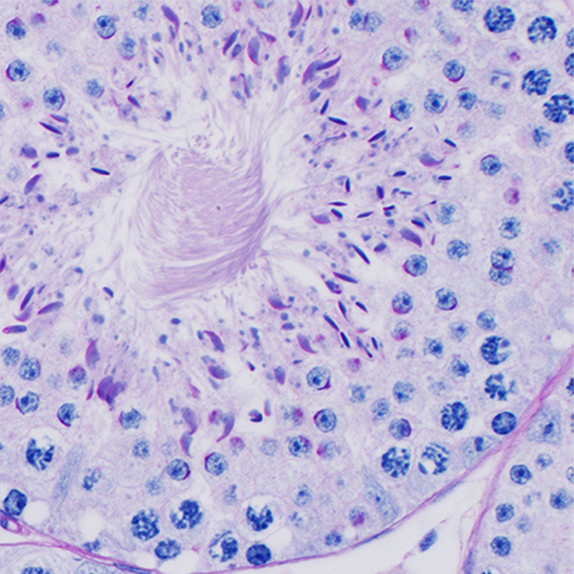

Supplement: Supplementary file 11 — Appendix and EV Figures Source Data [file 44319_2024_112_MOESM11_ESM.zip › Figure EV1-EV5/Figure EV2/2B/KO/VI.tif]

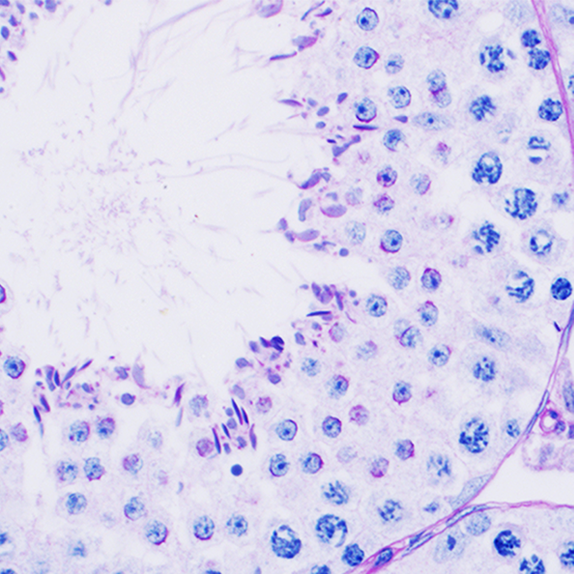

Supplement: Supplementary file 11 — Appendix and EV Figures Source Data [file 44319_2024_112_MOESM11_ESM.zip › Figure EV1-EV5/Figure EV2/2B/KO/VII.tif]

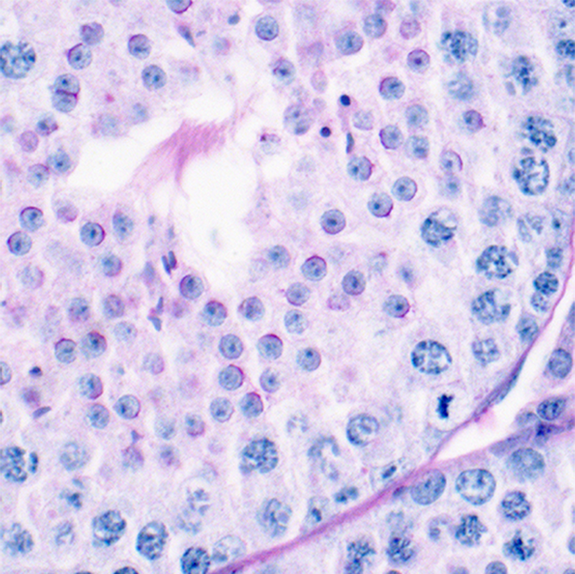

Supplement: Supplementary file 11 — Appendix and EV Figures Source Data [file 44319_2024_112_MOESM11_ESM.zip › Figure EV1-EV5/Figure EV2/2B/KO/VIII.tif]

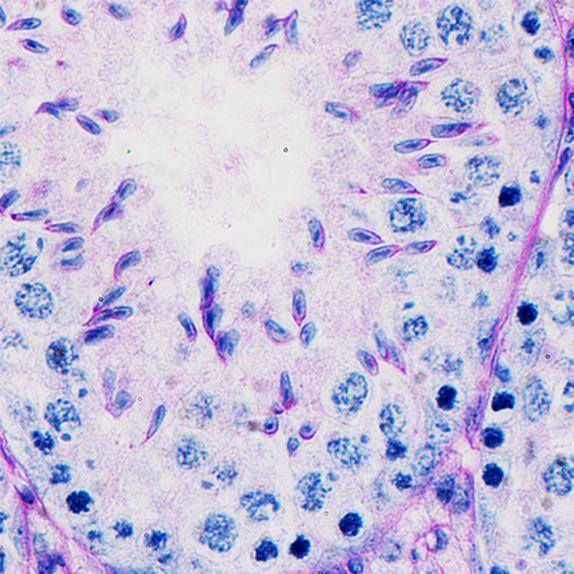

Supplement: Supplementary file 11 — Appendix and EV Figures Source Data [file 44319_2024_112_MOESM11_ESM.zip › Figure EV1-EV5/Figure EV2/2B/KO/X.tif]

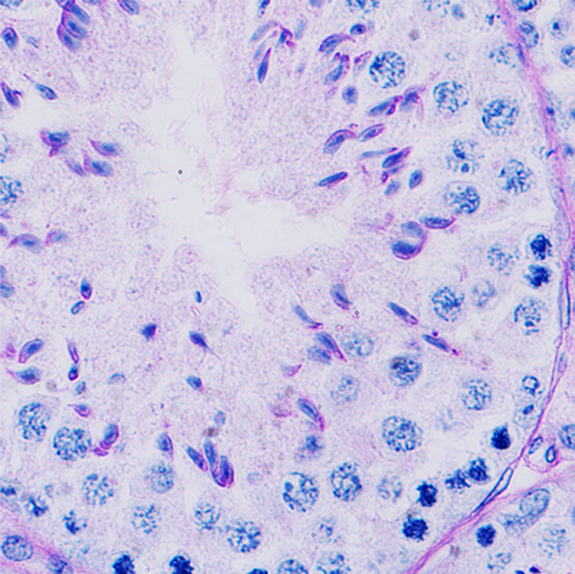

Supplement: Supplementary file 11 — Appendix and EV Figures Source Data [file 44319_2024_112_MOESM11_ESM.zip › Figure EV1-EV5/Figure EV2/2B/KO/XI.tif]

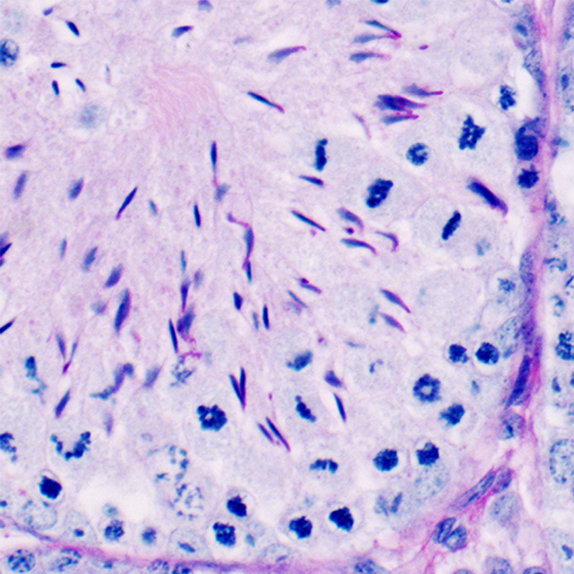

Supplement: Supplementary file 11 — Appendix and EV Figures Source Data [file 44319_2024_112_MOESM11_ESM.zip › Figure EV1-EV5/Figure EV2/2B/KO/XII.tif]

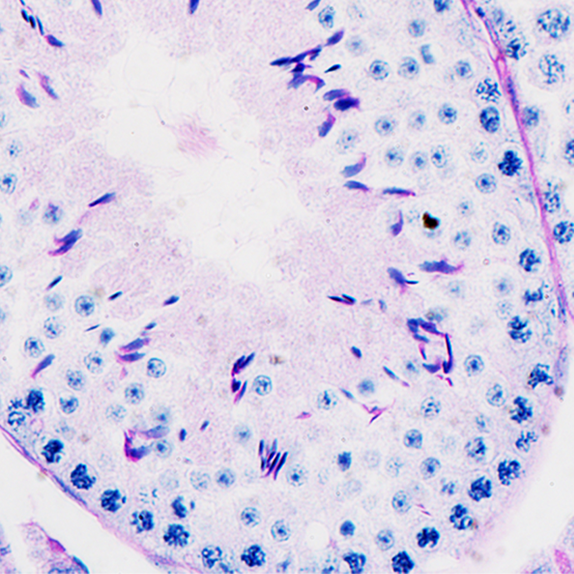

Supplement: Supplementary file 11 — Appendix and EV Figures Source Data [file 44319_2024_112_MOESM11_ESM.zip › Figure EV1-EV5/Figure EV2/2B/WT/I.tif]

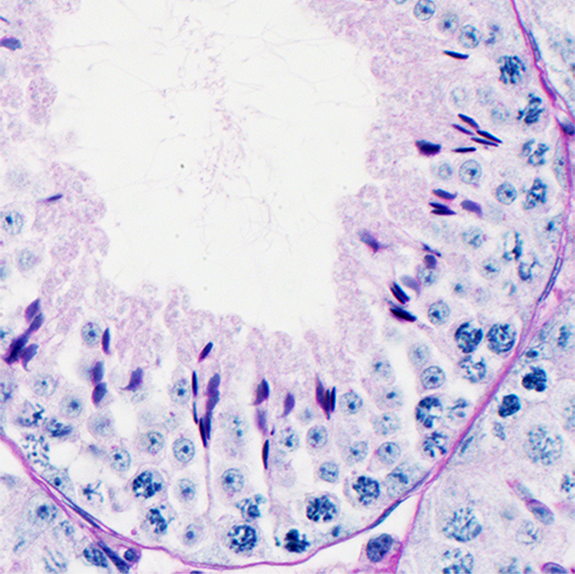

Supplement: Supplementary file 11 — Appendix and EV Figures Source Data [file 44319_2024_112_MOESM11_ESM.zip › Figure EV1-EV5/Figure EV2/2B/WT/II.tif]

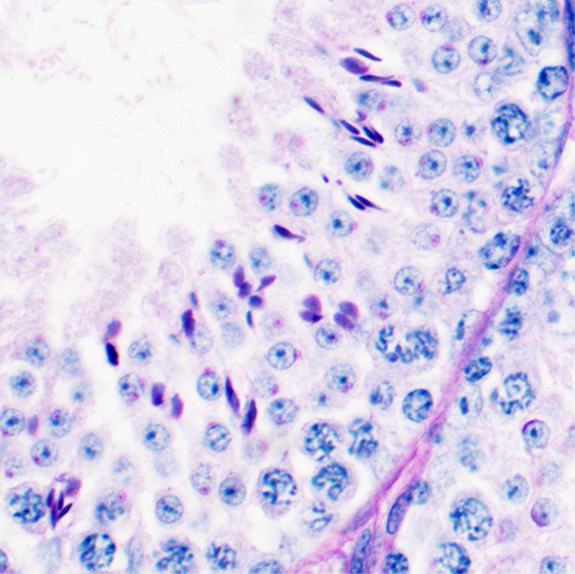

Supplement: Supplementary file 11 — Appendix and EV Figures Source Data [file 44319_2024_112_MOESM11_ESM.zip › Figure EV1-EV5/Figure EV2/2B/WT/III.tif]

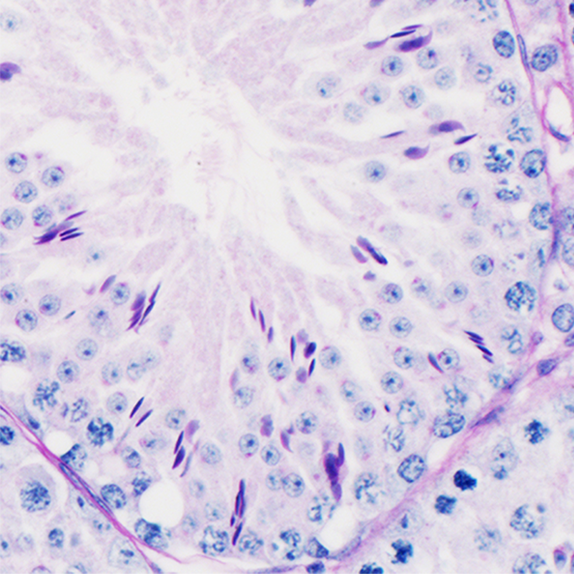

Supplement: Supplementary file 11 — Appendix and EV Figures Source Data [file 44319_2024_112_MOESM11_ESM.zip › Figure EV1-EV5/Figure EV2/2B/WT/IV.tif]

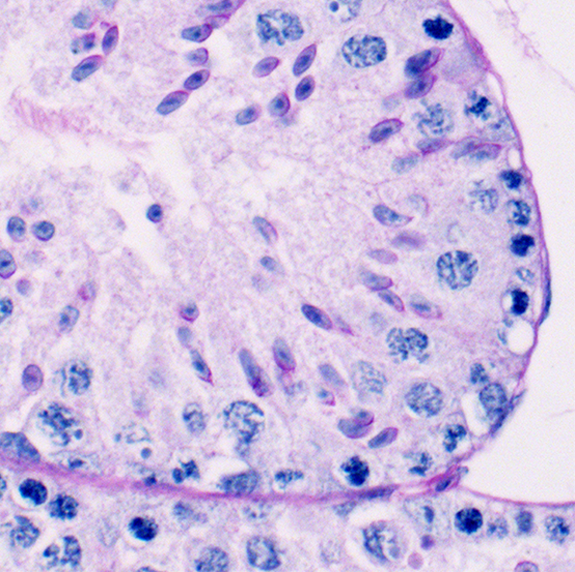

Supplement: Supplementary file 11 — Appendix and EV Figures Source Data [file 44319_2024_112_MOESM11_ESM.zip › Figure EV1-EV5/Figure EV2/2B/WT/IX.tif]

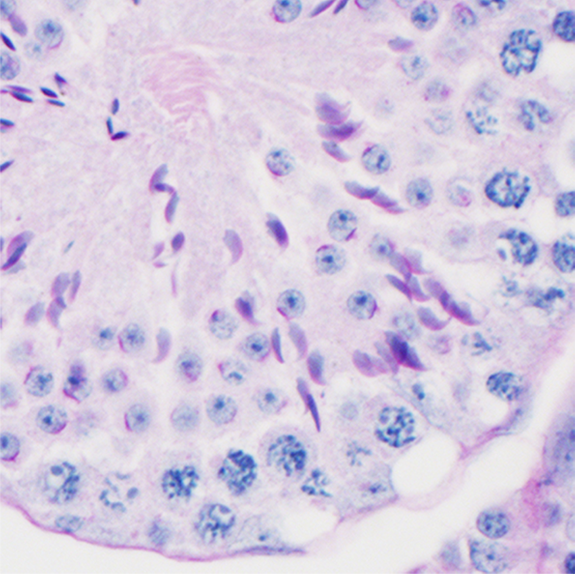

Supplement: Supplementary file 11 — Appendix and EV Figures Source Data [file 44319_2024_112_MOESM11_ESM.zip › Figure EV1-EV5/Figure EV2/2B/WT/V.tif]

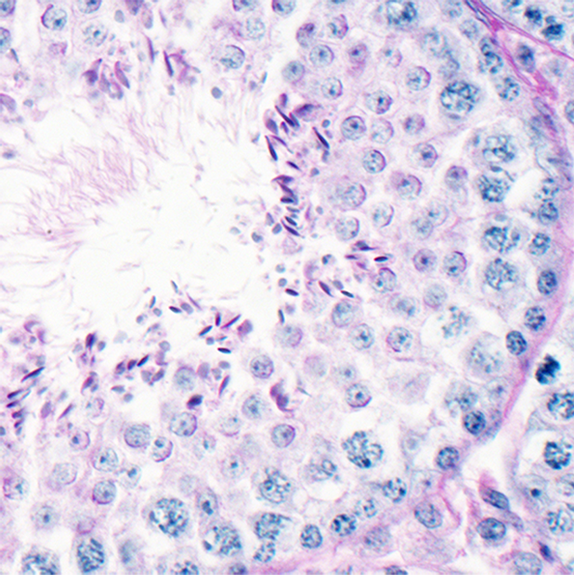

Supplement: Supplementary file 11 — Appendix and EV Figures Source Data [file 44319_2024_112_MOESM11_ESM.zip › Figure EV1-EV5/Figure EV2/2B/WT/VI.tif]

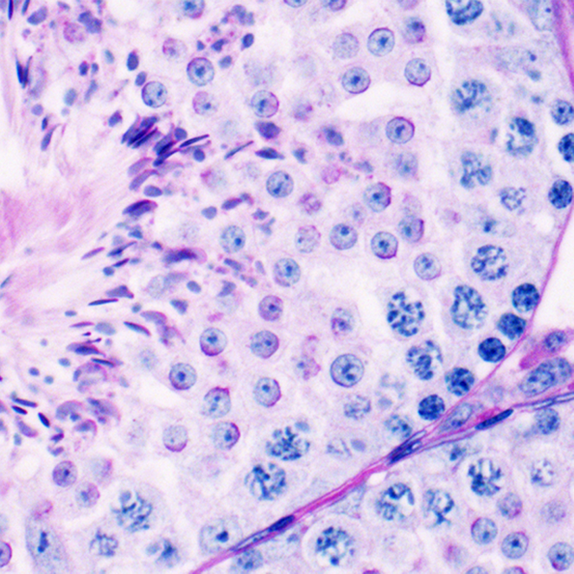

Supplement: Supplementary file 11 — Appendix and EV Figures Source Data [file 44319_2024_112_MOESM11_ESM.zip › Figure EV1-EV5/Figure EV2/2B/WT/VII.tif]

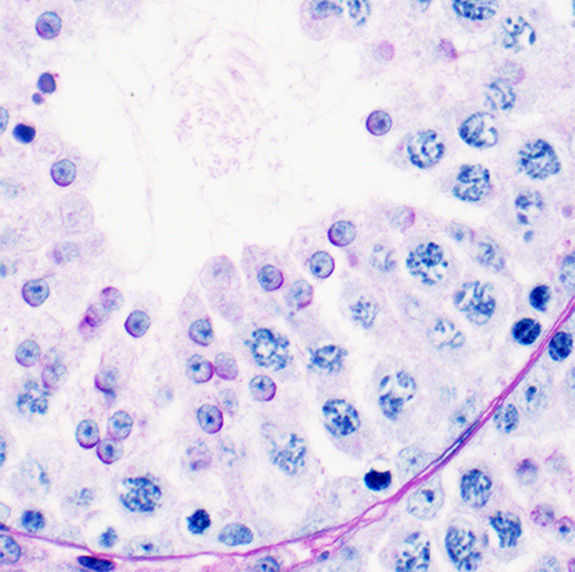

Supplement: Supplementary file 11 — Appendix and EV Figures Source Data [file 44319_2024_112_MOESM11_ESM.zip › Figure EV1-EV5/Figure EV2/2B/WT/VIII.tif]

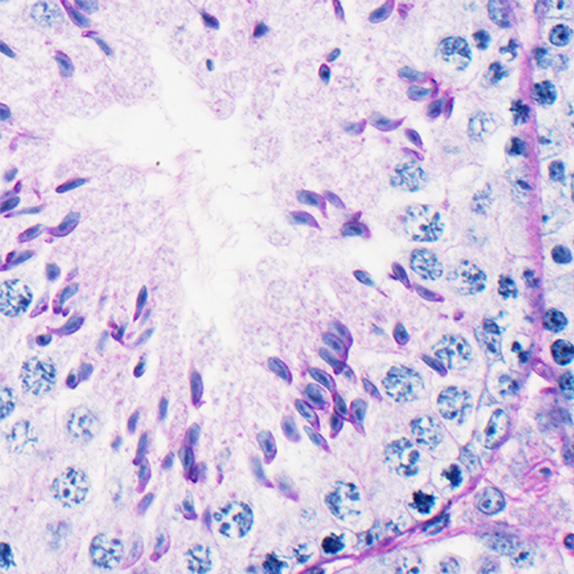

Supplement: Supplementary file 11 — Appendix and EV Figures Source Data [file 44319_2024_112_MOESM11_ESM.zip › Figure EV1-EV5/Figure EV2/2B/WT/X.tif]

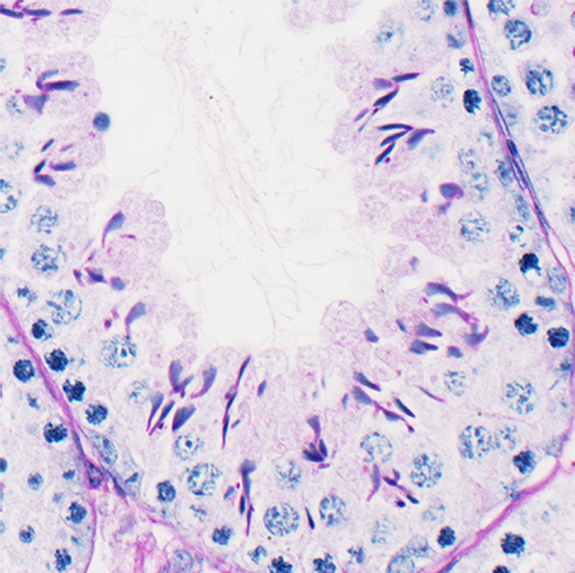

Supplement: Supplementary file 11 — Appendix and EV Figures Source Data [file 44319_2024_112_MOESM11_ESM.zip › Figure EV1-EV5/Figure EV2/2B/WT/XI.tif]

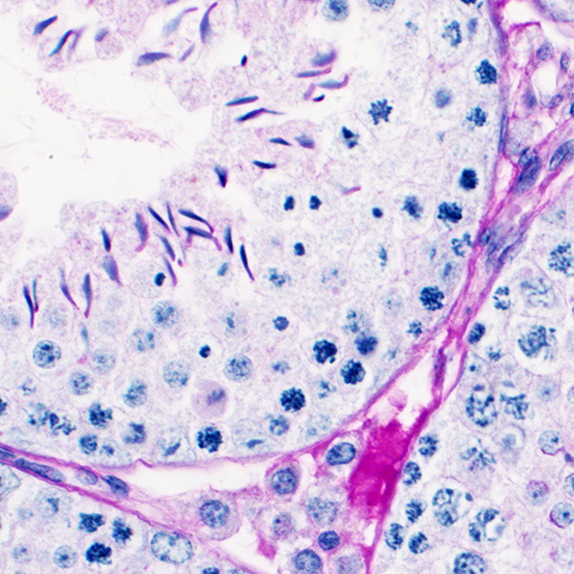

Supplement: Supplementary file 11 — Appendix and EV Figures Source Data [file 44319_2024_112_MOESM11_ESM.zip › Figure EV1-EV5/Figure EV2/2B/WT/XII.tif]

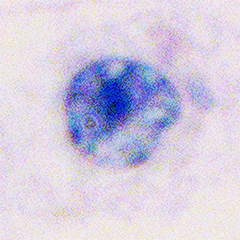

Supplement: Supplementary file 11 — Appendix and EV Figures Source Data [file 44319_2024_112_MOESM11_ESM.zip › Figure EV1-EV5/Figure EV2/2E/KO/STEP 1.tif]

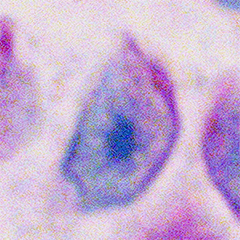

Supplement: Supplementary file 11 — Appendix and EV Figures Source Data [file 44319_2024_112_MOESM11_ESM.zip › Figure EV1-EV5/Figure EV2/2E/KO/STEP 10.tif]

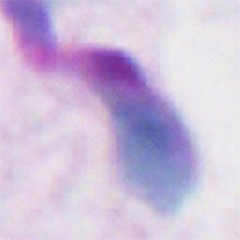

Supplement: Supplementary file 11 — Appendix and EV Figures Source Data [file 44319_2024_112_MOESM11_ESM.zip › Figure EV1-EV5/Figure EV2/2E/KO/STEP 11.tif]

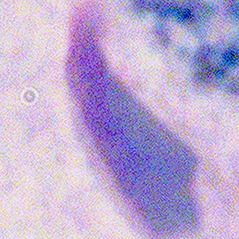

Supplement: Supplementary file 11 — Appendix and EV Figures Source Data [file 44319_2024_112_MOESM11_ESM.zip › Figure EV1-EV5/Figure EV2/2E/KO/STEP 12.tif]

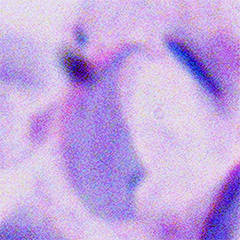

Supplement: Supplementary file 11 — Appendix and EV Figures Source Data [file 44319_2024_112_MOESM11_ESM.zip › Figure EV1-EV5/Figure EV2/2E/KO/STEP 13.tif]

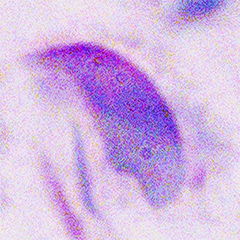

Supplement: Supplementary file 11 — Appendix and EV Figures Source Data [file 44319_2024_112_MOESM11_ESM.zip › Figure EV1-EV5/Figure EV2/2E/KO/STEP 14.tif]

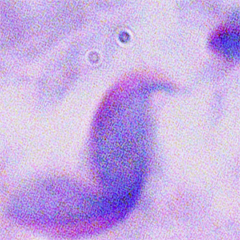

Supplement: Supplementary file 11 — Appendix and EV Figures Source Data [file 44319_2024_112_MOESM11_ESM.zip › Figure EV1-EV5/Figure EV2/2E/KO/STEP 15.tif]

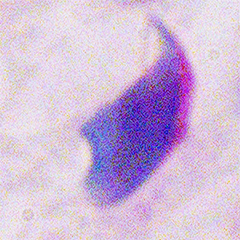

Supplement: Supplementary file 11 — Appendix and EV Figures Source Data [file 44319_2024_112_MOESM11_ESM.zip › Figure EV1-EV5/Figure EV2/2E/KO/STEP 16.tif]

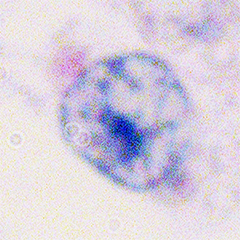

Supplement: Supplementary file 11 — Appendix and EV Figures Source Data [file 44319_2024_112_MOESM11_ESM.zip › Figure EV1-EV5/Figure EV2/2E/KO/STEP 2.tif]

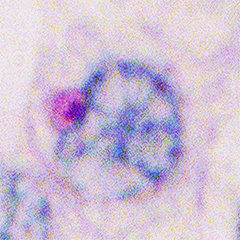

Supplement: Supplementary file 11 — Appendix and EV Figures Source Data [file 44319_2024_112_MOESM11_ESM.zip › Figure EV1-EV5/Figure EV2/2E/KO/STEP 3.tif]

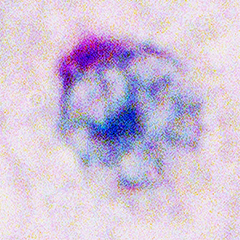

Supplement: Supplementary file 11 — Appendix and EV Figures Source Data [file 44319_2024_112_MOESM11_ESM.zip › Figure EV1-EV5/Figure EV2/2E/KO/STEP 4.tif]

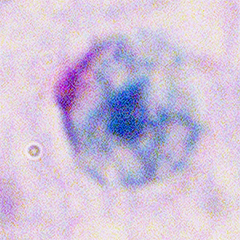

Supplement: Supplementary file 11 — Appendix and EV Figures Source Data [file 44319_2024_112_MOESM11_ESM.zip › Figure EV1-EV5/Figure EV2/2E/KO/STEP 5.tif]

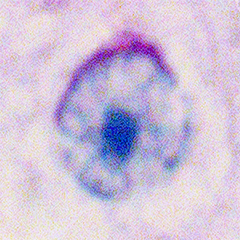

Supplement: Supplementary file 11 — Appendix and EV Figures Source Data [file 44319_2024_112_MOESM11_ESM.zip › Figure EV1-EV5/Figure EV2/2E/KO/STEP 6.tif]

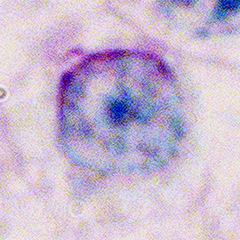

Supplement: Supplementary file 11 — Appendix and EV Figures Source Data [file 44319_2024_112_MOESM11_ESM.zip › Figure EV1-EV5/Figure EV2/2E/KO/STEP 7.tif]

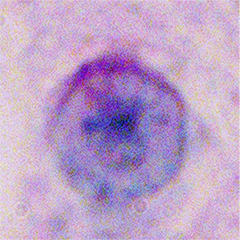

Supplement: Supplementary file 11 — Appendix and EV Figures Source Data [file 44319_2024_112_MOESM11_ESM.zip › Figure EV1-EV5/Figure EV2/2E/KO/STEP 8.tif]

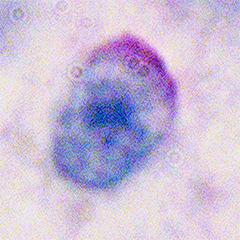

Supplement: Supplementary file 11 — Appendix and EV Figures Source Data [file 44319_2024_112_MOESM11_ESM.zip › Figure EV1-EV5/Figure EV2/2E/KO/STEP 9.tif]

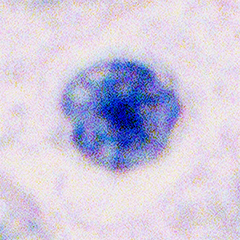

Supplement: Supplementary file 11 — Appendix and EV Figures Source Data [file 44319_2024_112_MOESM11_ESM.zip › Figure EV1-EV5/Figure EV2/2E/WT/STEP 1.tif]

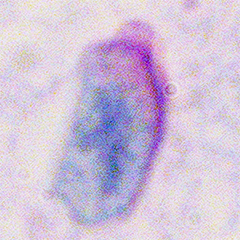

Supplement: Supplementary file 11 — Appendix and EV Figures Source Data [file 44319_2024_112_MOESM11_ESM.zip › Figure EV1-EV5/Figure EV2/2E/WT/STEP 10.tif]

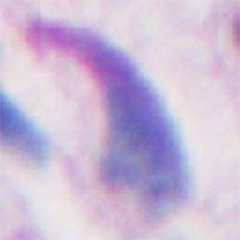

Supplement: Supplementary file 11 — Appendix and EV Figures Source Data [file 44319_2024_112_MOESM11_ESM.zip › Figure EV1-EV5/Figure EV2/2E/WT/STEP 11.tif]

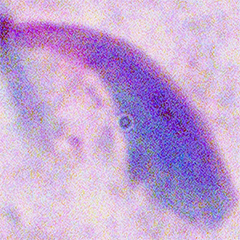

Supplement: Supplementary file 11 — Appendix and EV Figures Source Data [file 44319_2024_112_MOESM11_ESM.zip › Figure EV1-EV5/Figure EV2/2E/WT/STEP 12.tif]

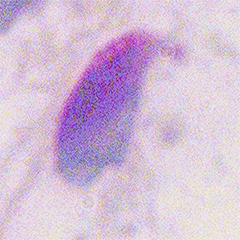

Supplement: Supplementary file 11 — Appendix and EV Figures Source Data [file 44319_2024_112_MOESM11_ESM.zip › Figure EV1-EV5/Figure EV2/2E/WT/STEP 13.tif]

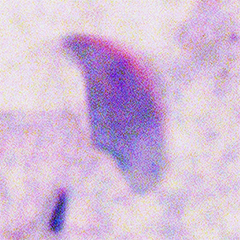

Supplement: Supplementary file 11 — Appendix and EV Figures Source Data [file 44319_2024_112_MOESM11_ESM.zip › Figure EV1-EV5/Figure EV2/2E/WT/STEP 14.tif]

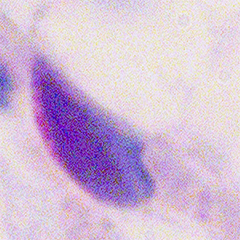

Supplement: Supplementary file 11 — Appendix and EV Figures Source Data [file 44319_2024_112_MOESM11_ESM.zip › Figure EV1-EV5/Figure EV2/2E/WT/STEP 15.tif]

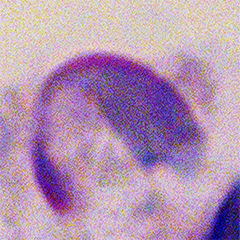

Supplement: Supplementary file 11 — Appendix and EV Figures Source Data [file 44319_2024_112_MOESM11_ESM.zip › Figure EV1-EV5/Figure EV2/2E/WT/STEP 16.tif]

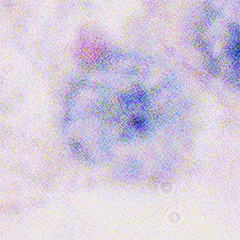

Supplement: Supplementary file 11 — Appendix and EV Figures Source Data [file 44319_2024_112_MOESM11_ESM.zip › Figure EV1-EV5/Figure EV2/2E/WT/STEP 2.tif]

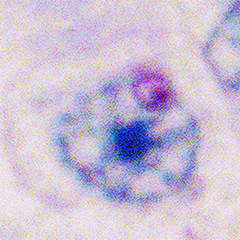

Supplement: Supplementary file 11 — Appendix and EV Figures Source Data [file 44319_2024_112_MOESM11_ESM.zip › Figure EV1-EV5/Figure EV2/2E/WT/STEP 3.tif]

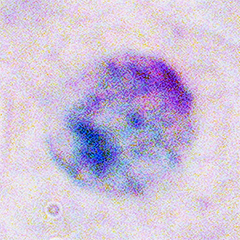

Supplement: Supplementary file 11 — Appendix and EV Figures Source Data [file 44319_2024_112_MOESM11_ESM.zip › Figure EV1-EV5/Figure EV2/2E/WT/STEP 4.tif]

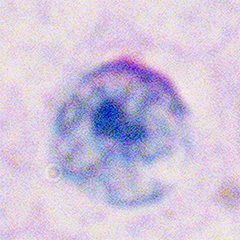

Supplement: Supplementary file 11 — Appendix and EV Figures Source Data [file 44319_2024_112_MOESM11_ESM.zip › Figure EV1-EV5/Figure EV2/2E/WT/STEP 5.tif]

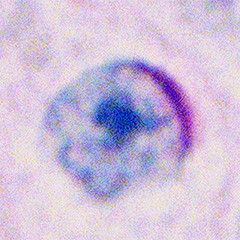

Supplement: Supplementary file 11 — Appendix and EV Figures Source Data [file 44319_2024_112_MOESM11_ESM.zip › Figure EV1-EV5/Figure EV2/2E/WT/STEP 6.tif]

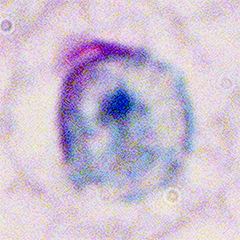

Supplement: Supplementary file 11 — Appendix and EV Figures Source Data [file 44319_2024_112_MOESM11_ESM.zip › Figure EV1-EV5/Figure EV2/2E/WT/STEP 7.tif]

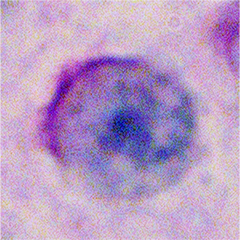

Supplement: Supplementary file 11 — Appendix and EV Figures Source Data [file 44319_2024_112_MOESM11_ESM.zip › Figure EV1-EV5/Figure EV2/2E/WT/STEP 8.tif]

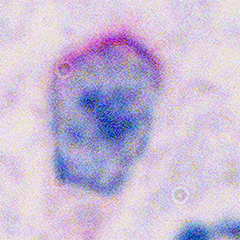

Supplement: Supplementary file 11 — Appendix and EV Figures Source Data [file 44319_2024_112_MOESM11_ESM.zip › Figure EV1-EV5/Figure EV2/2E/WT/STEP 9.tif]

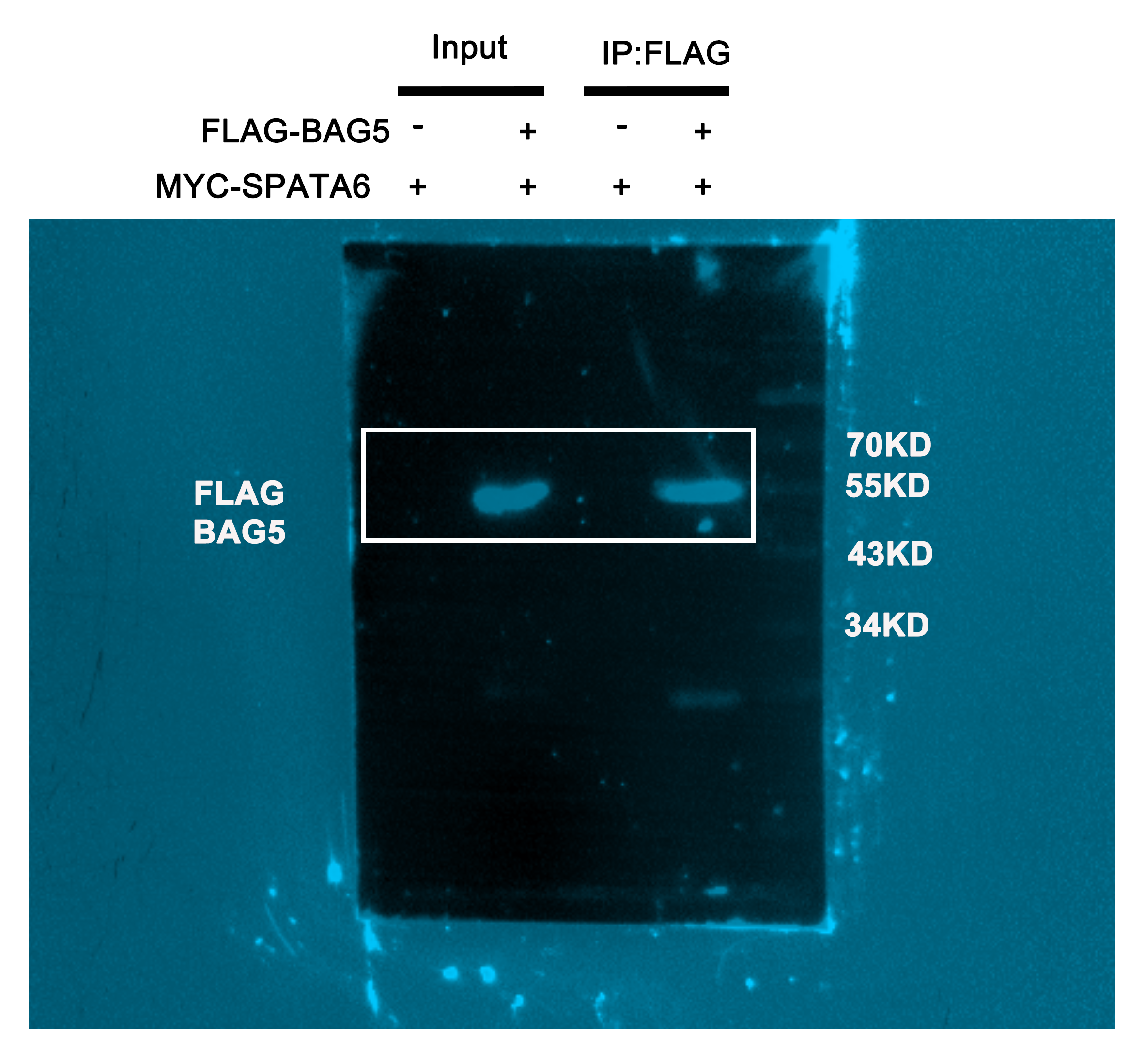

Supplement: Supplementary file 11 — Appendix and EV Figures Source Data [file 44319_2024_112_MOESM11_ESM.zip › Figure EV1-EV5/Figure EV3/3D/WB FLAG.tif]

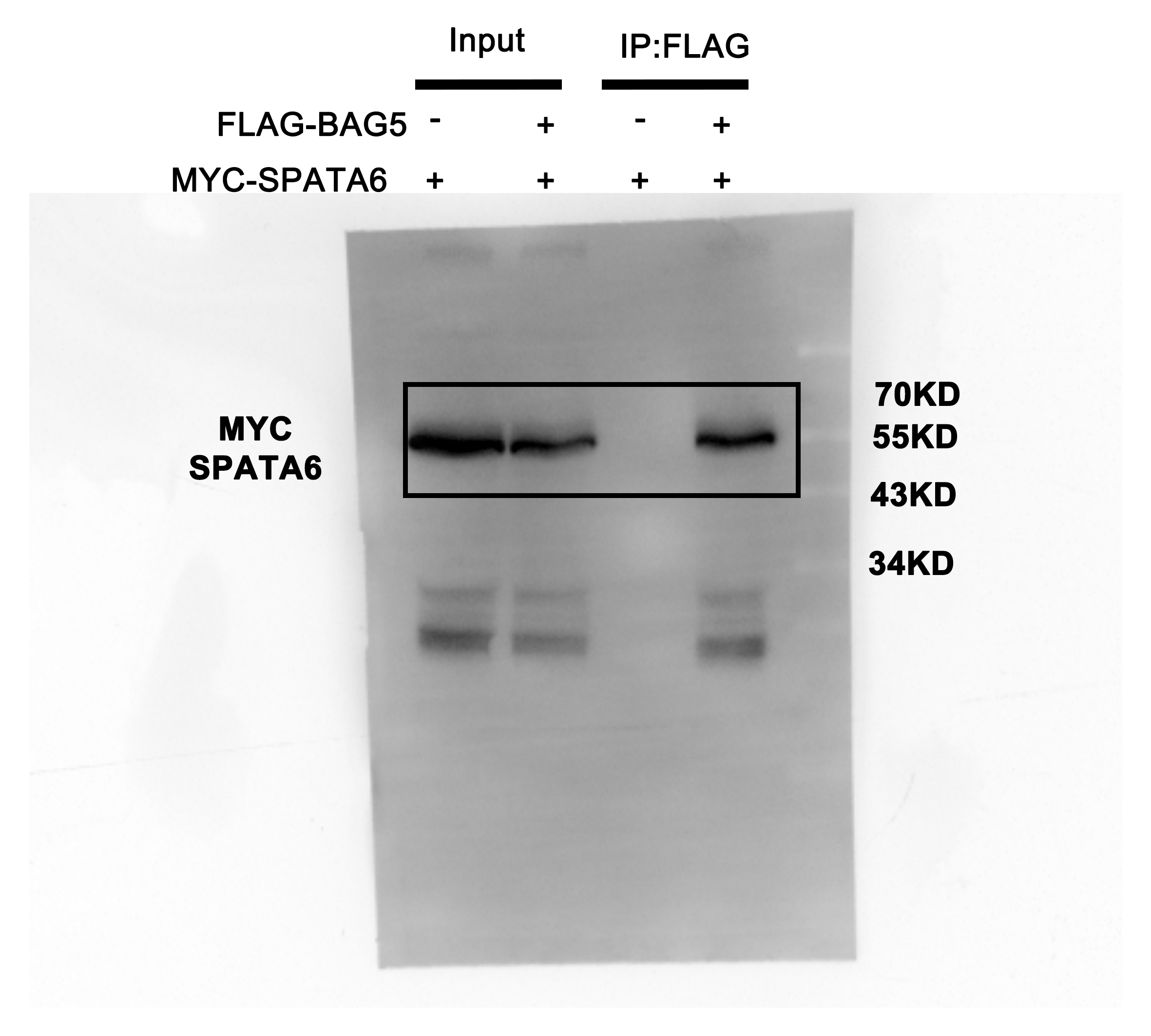

Supplement: Supplementary file 11 — Appendix and EV Figures Source Data [file 44319_2024_112_MOESM11_ESM.zip › Figure EV1-EV5/Figure EV3/3D/WB MYC.tif]

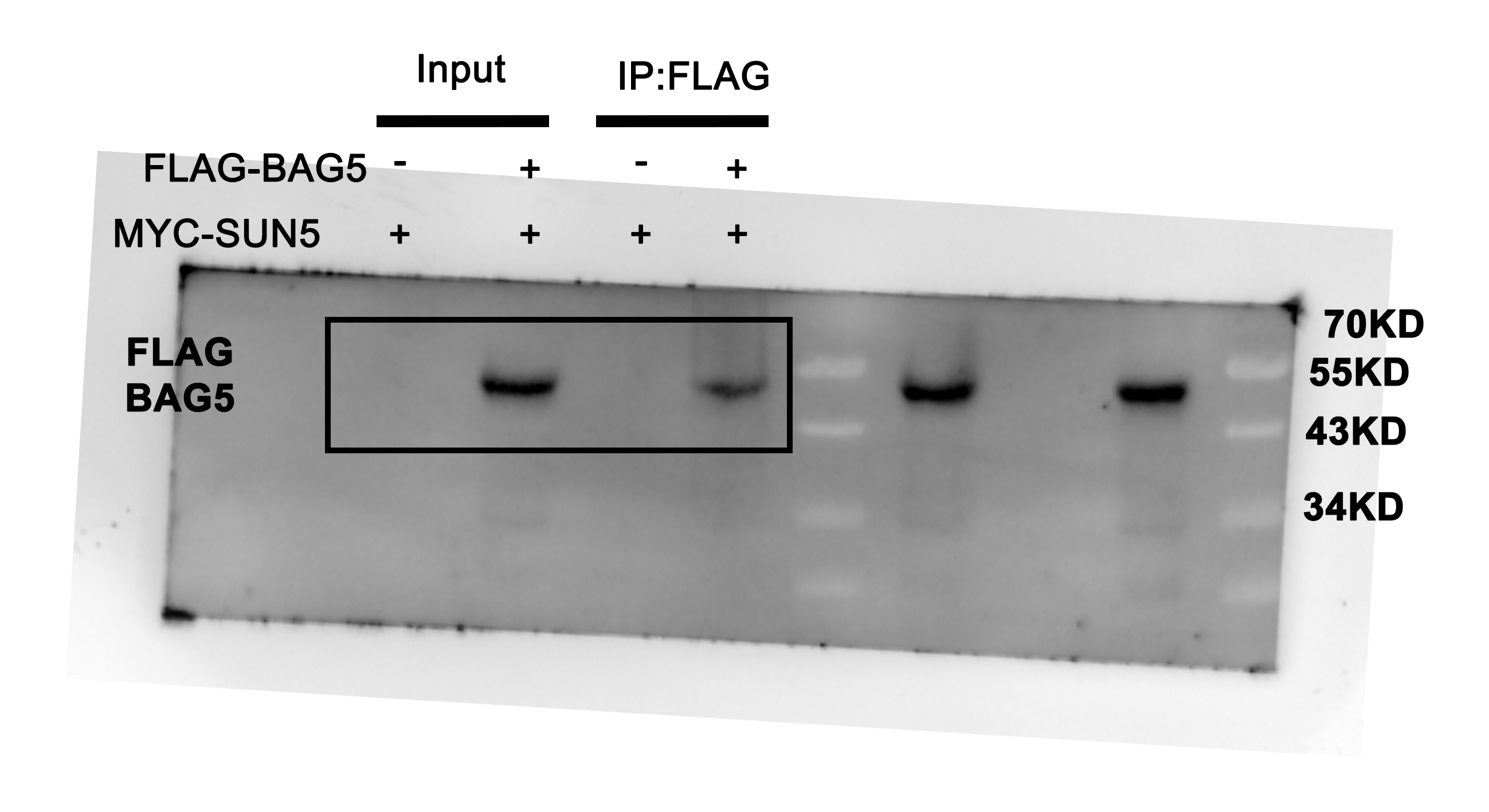

Supplement: Supplementary file 11 — Appendix and EV Figures Source Data [file 44319_2024_112_MOESM11_ESM.zip › Figure EV1-EV5/Figure EV3/3E/WB BAG5.tif]

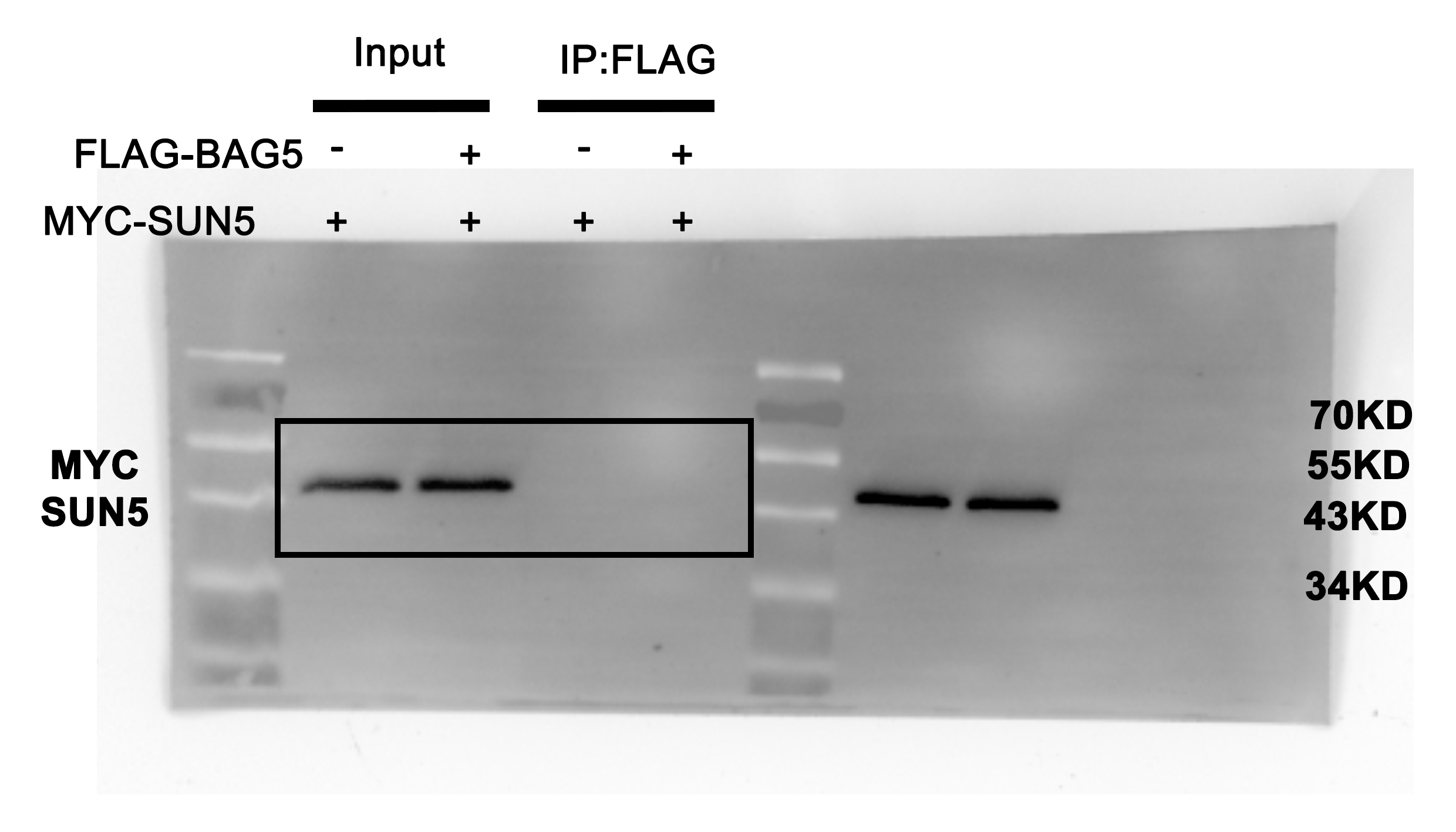

Supplement: Supplementary file 11 — Appendix and EV Figures Source Data [file 44319_2024_112_MOESM11_ESM.zip › Figure EV1-EV5/Figure EV3/3E/WB SUN5.tif]

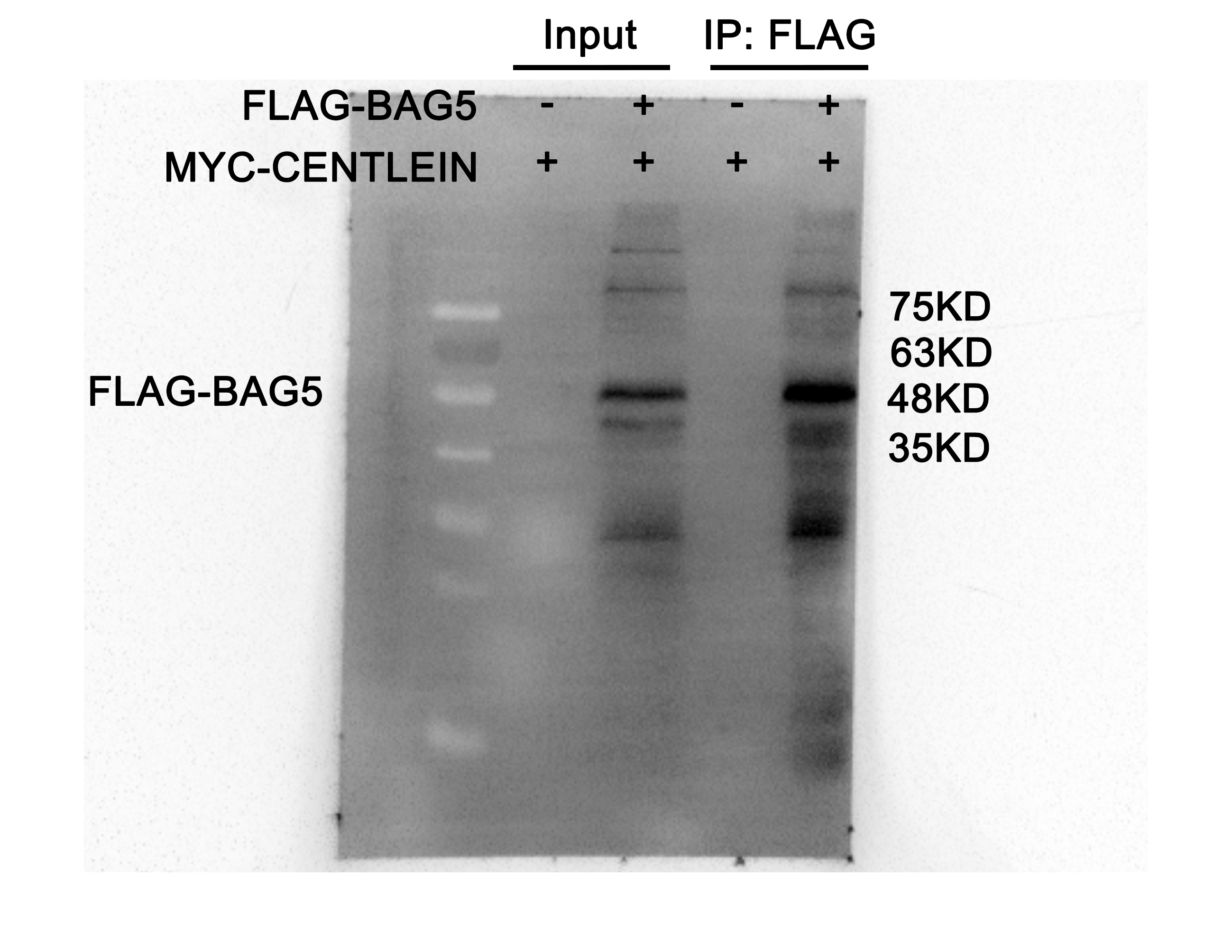

Supplement: Supplementary file 11 — Appendix and EV Figures Source Data [file 44319_2024_112_MOESM11_ESM.zip › Figure EV1-EV5/Figure EV3/3F/WB BAG5.tif]

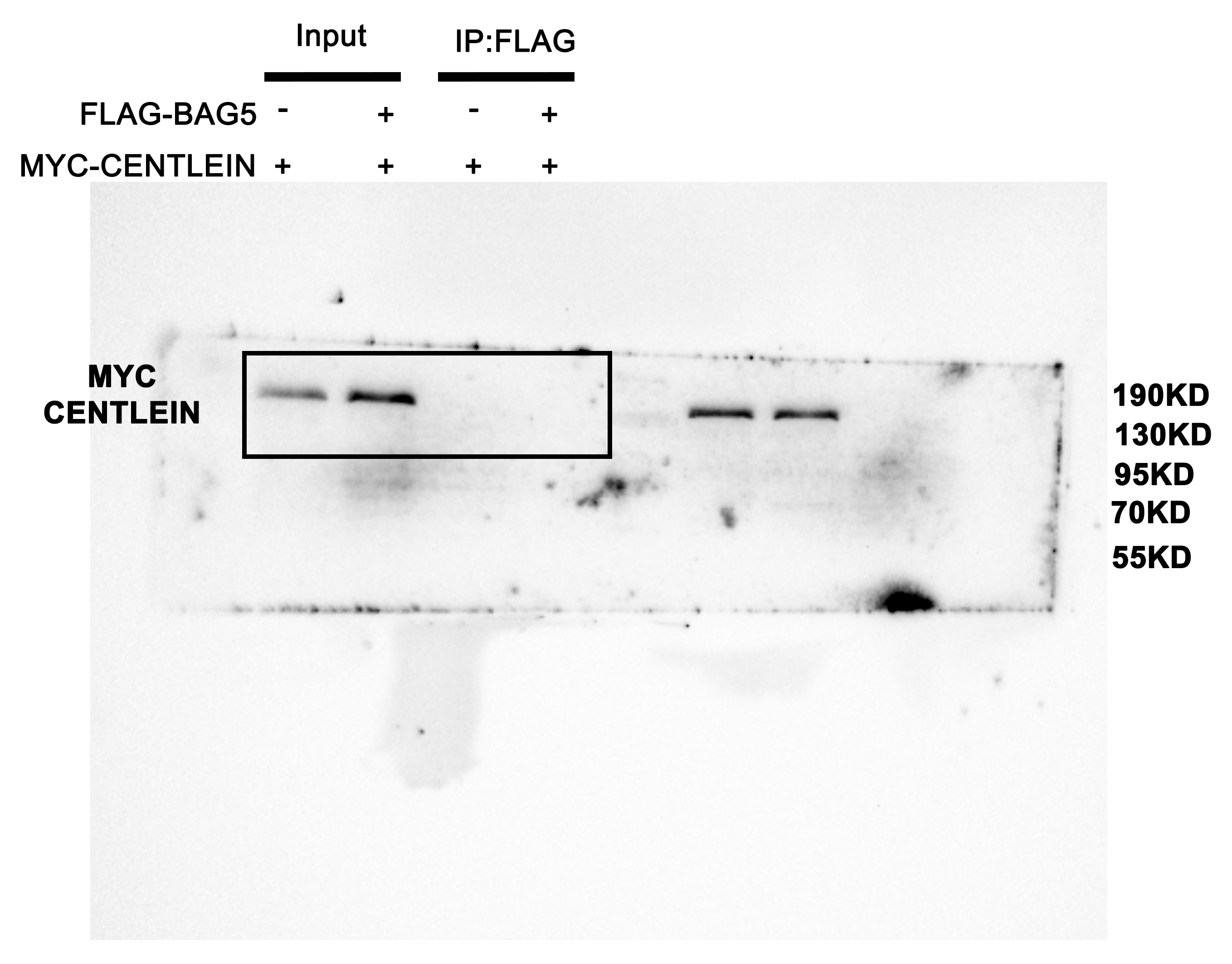

Supplement: Supplementary file 11 — Appendix and EV Figures Source Data [file 44319_2024_112_MOESM11_ESM.zip › Figure EV1-EV5/Figure EV3/3F/WB CENTLEIN.tif]

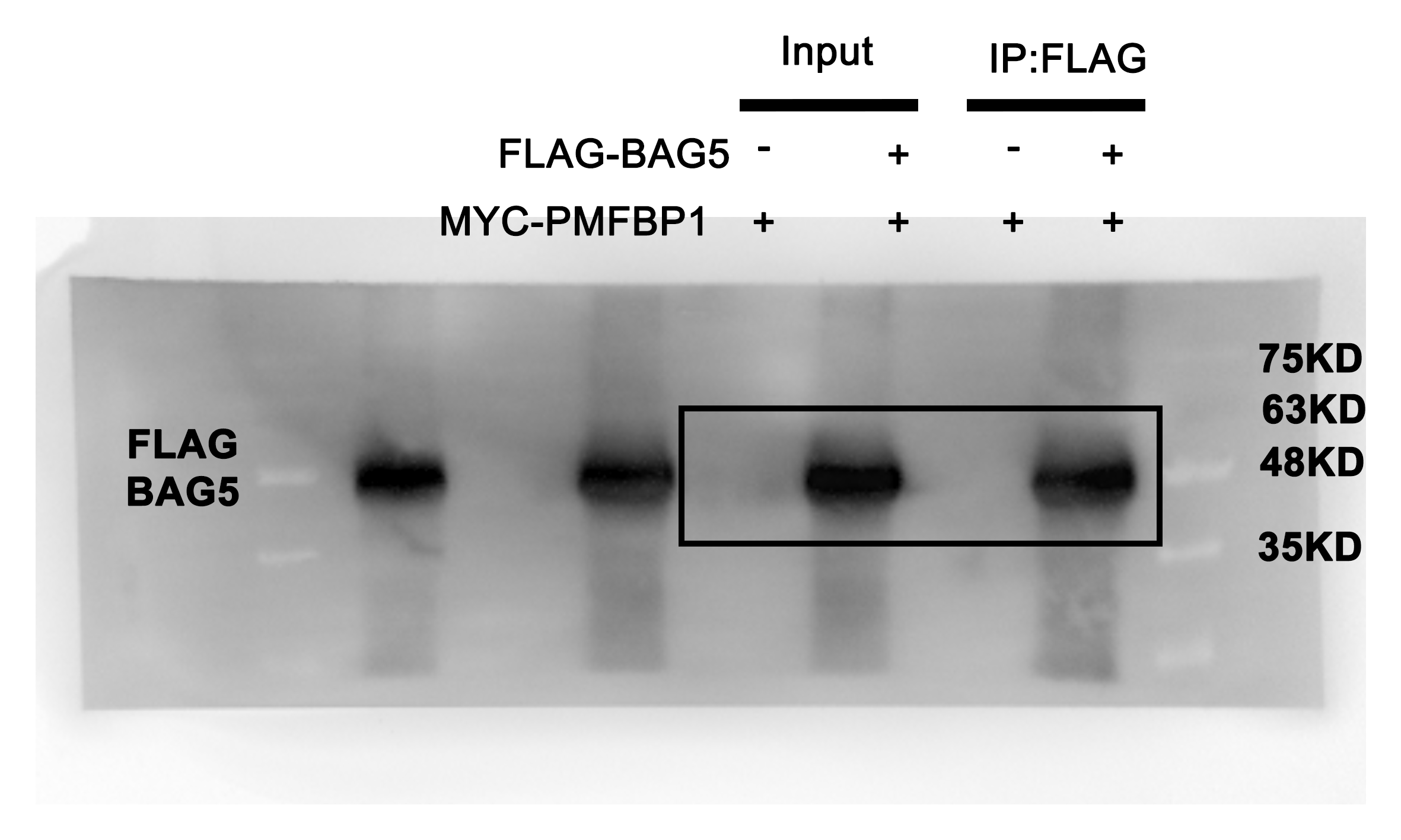

Supplement: Supplementary file 11 — Appendix and EV Figures Source Data [file 44319_2024_112_MOESM11_ESM.zip › Figure EV1-EV5/Figure EV3/3G/WB BAG5.tif]

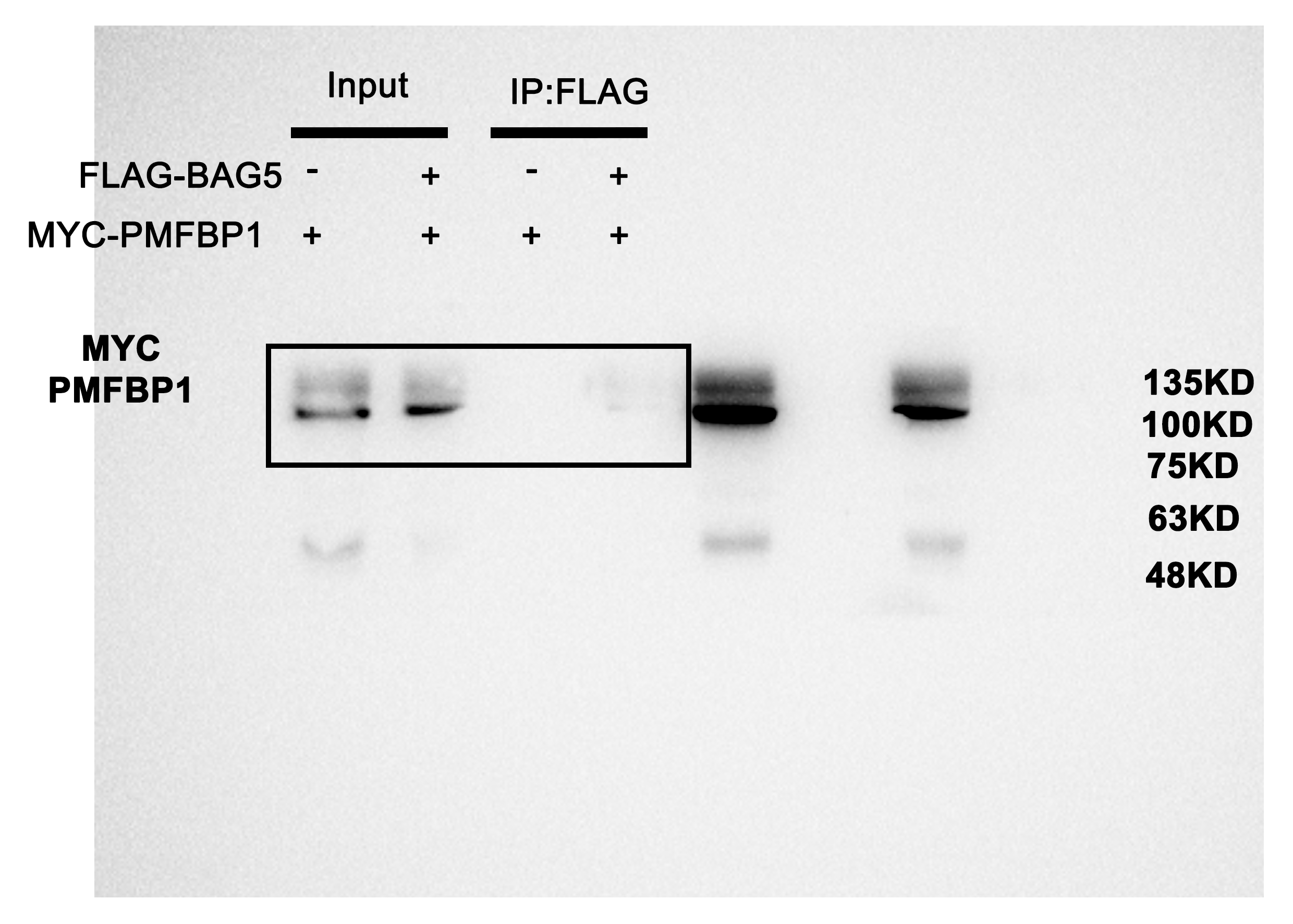

Supplement: Supplementary file 11 — Appendix and EV Figures Source Data [file 44319_2024_112_MOESM11_ESM.zip › Figure EV1-EV5/Figure EV3/3G/WB PMFBP1.tif]

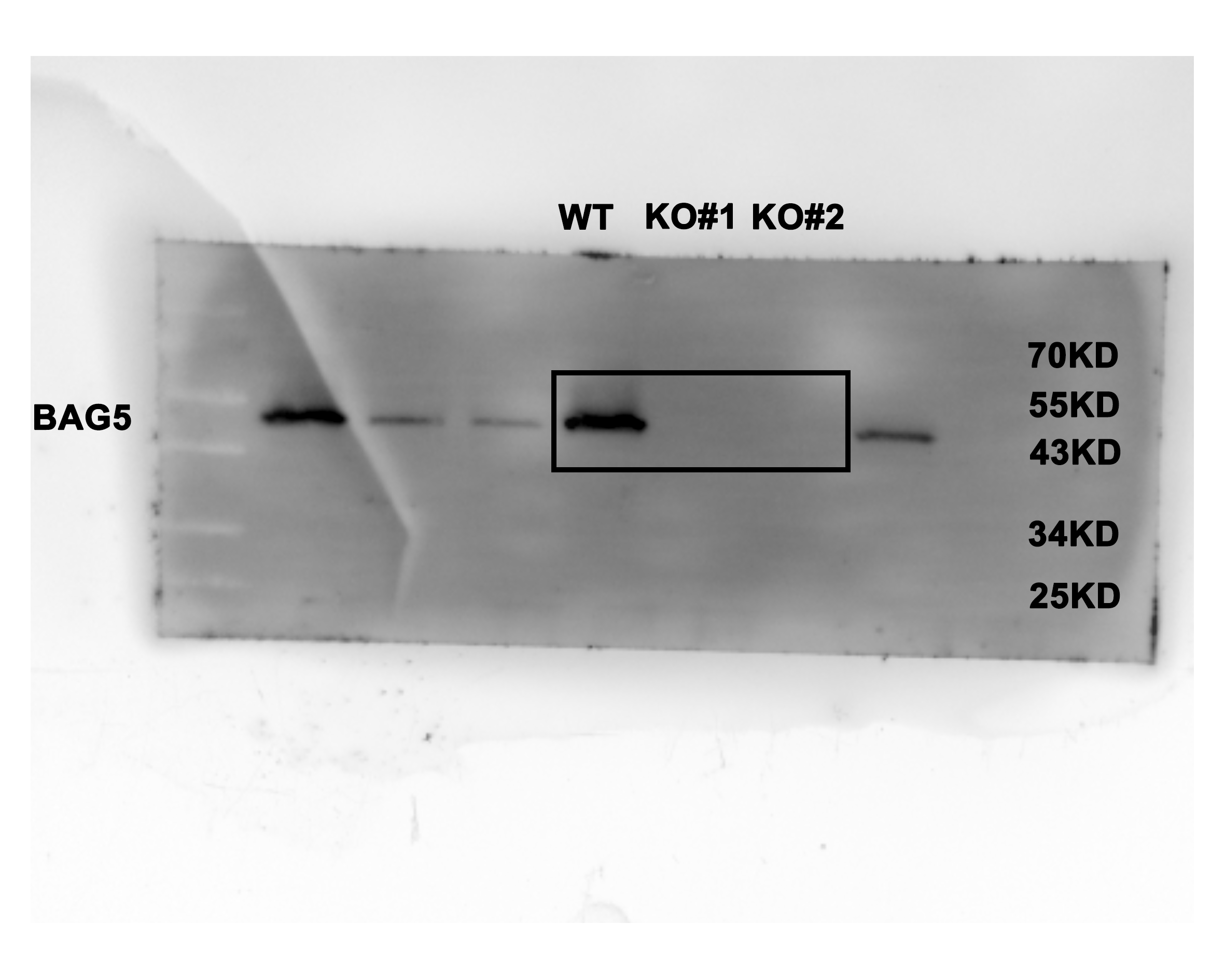

Supplement: Supplementary file 11 — Appendix and EV Figures Source Data [file 44319_2024_112_MOESM11_ESM.zip › Figure EV1-EV5/Figure EV4/4B/WB BAG5.tif]

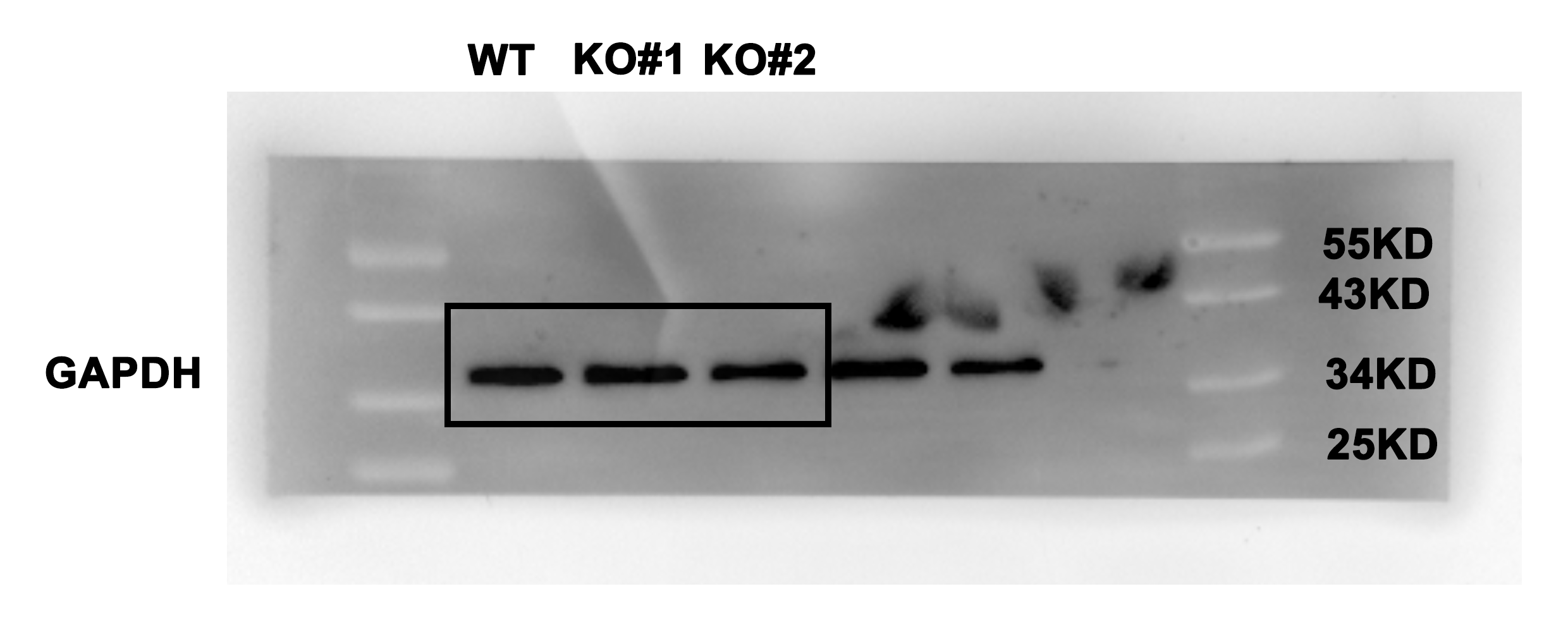

Supplement: Supplementary file 11 — Appendix and EV Figures Source Data [file 44319_2024_112_MOESM11_ESM.zip › Figure EV1-EV5/Figure EV4/4B/WB GAPDH.tif]

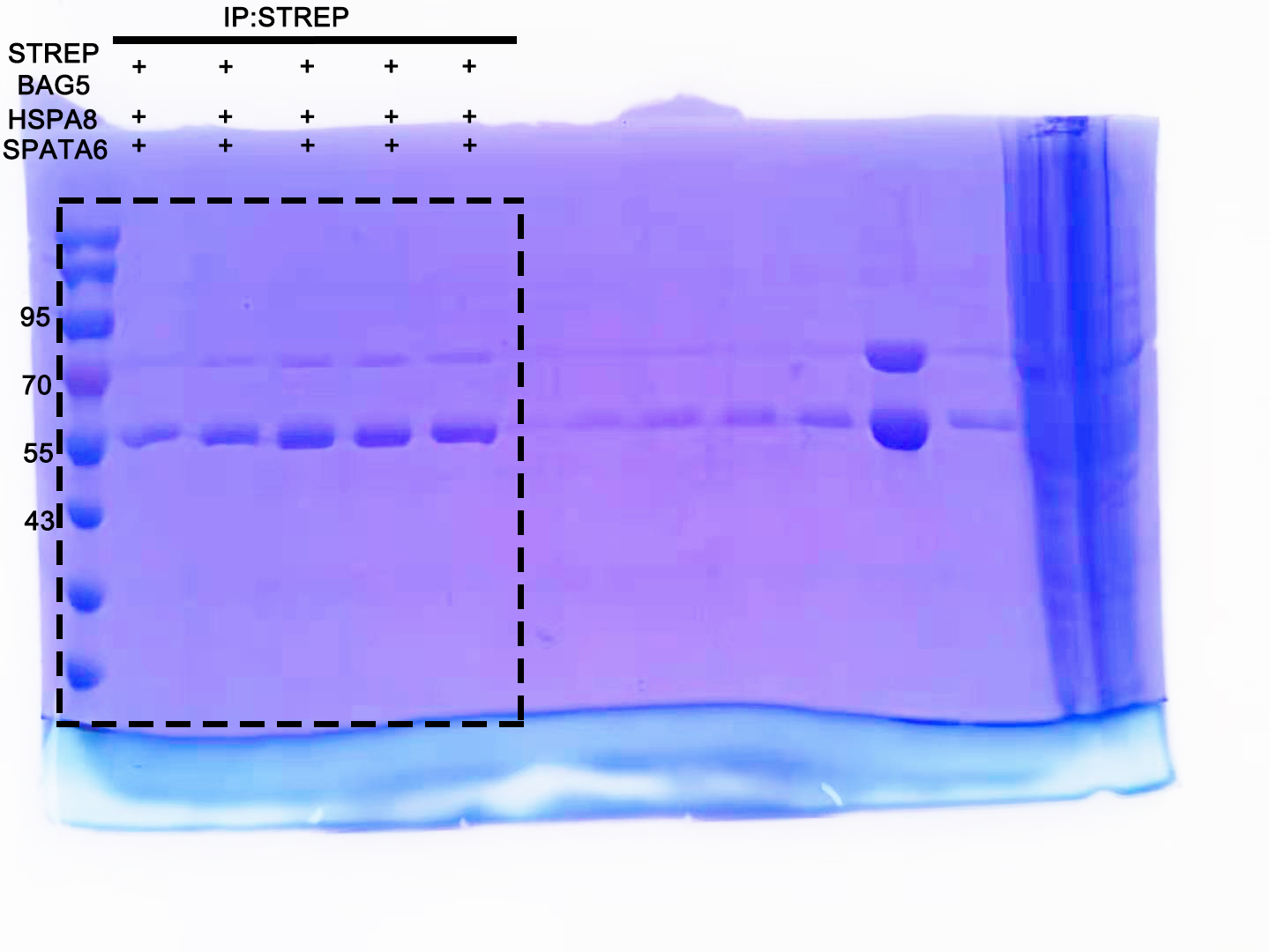

Supplement: Supplementary file 11 — Appendix and EV Figures Source Data [file 44319_2024_112_MOESM11_ESM.zip › Figure EV1-EV5/Figure EV5/5A/Coomassie brilliant blue.jpg]

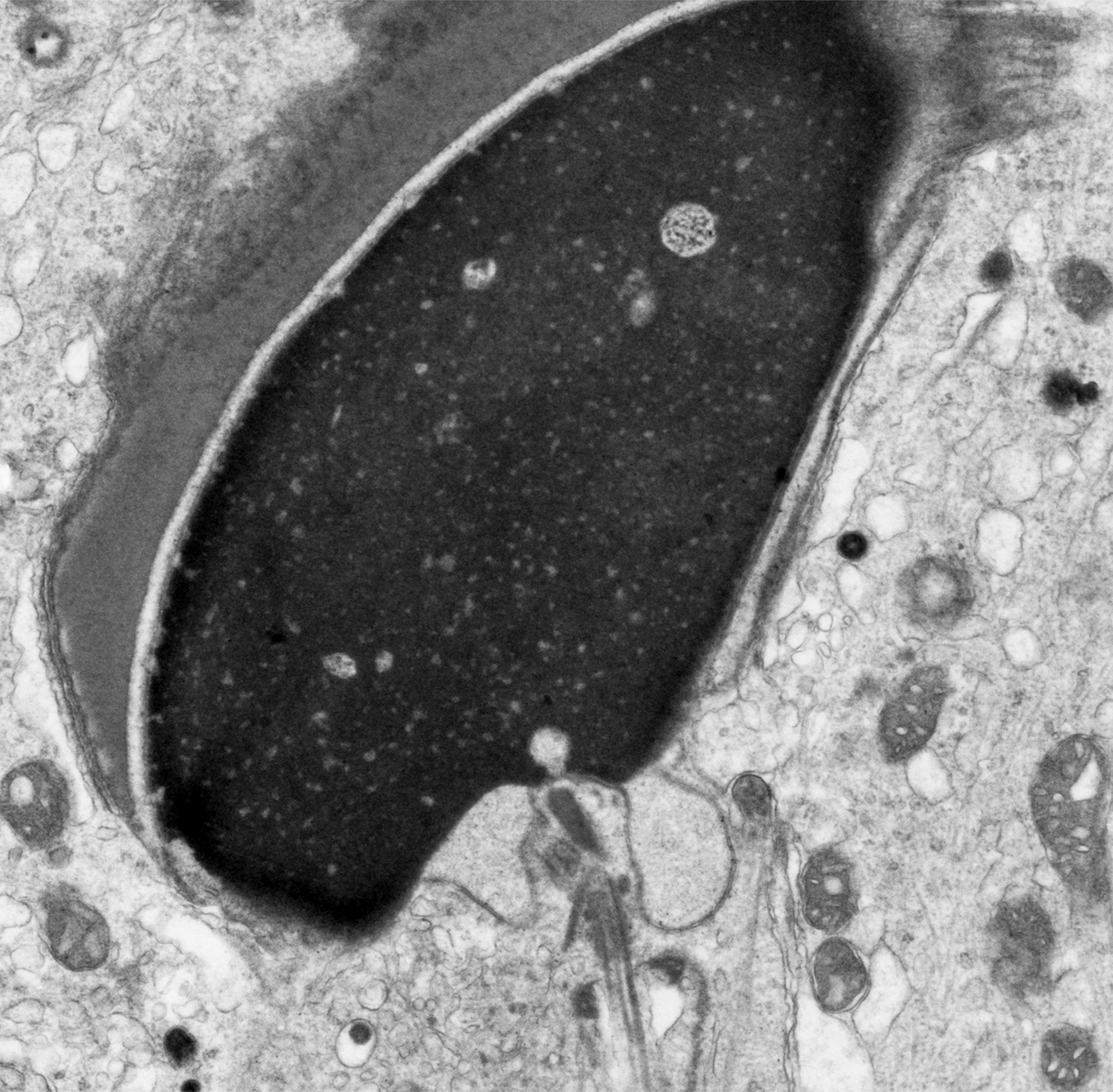

Supplement: Supplementary file 11 — Appendix and EV Figures Source Data [file 44319_2024_112_MOESM11_ESM.zip › Appendix Figure S1,S4,S5,S6/Appendix Figure S1/Appendix Figure S1/Basal plate.tif]

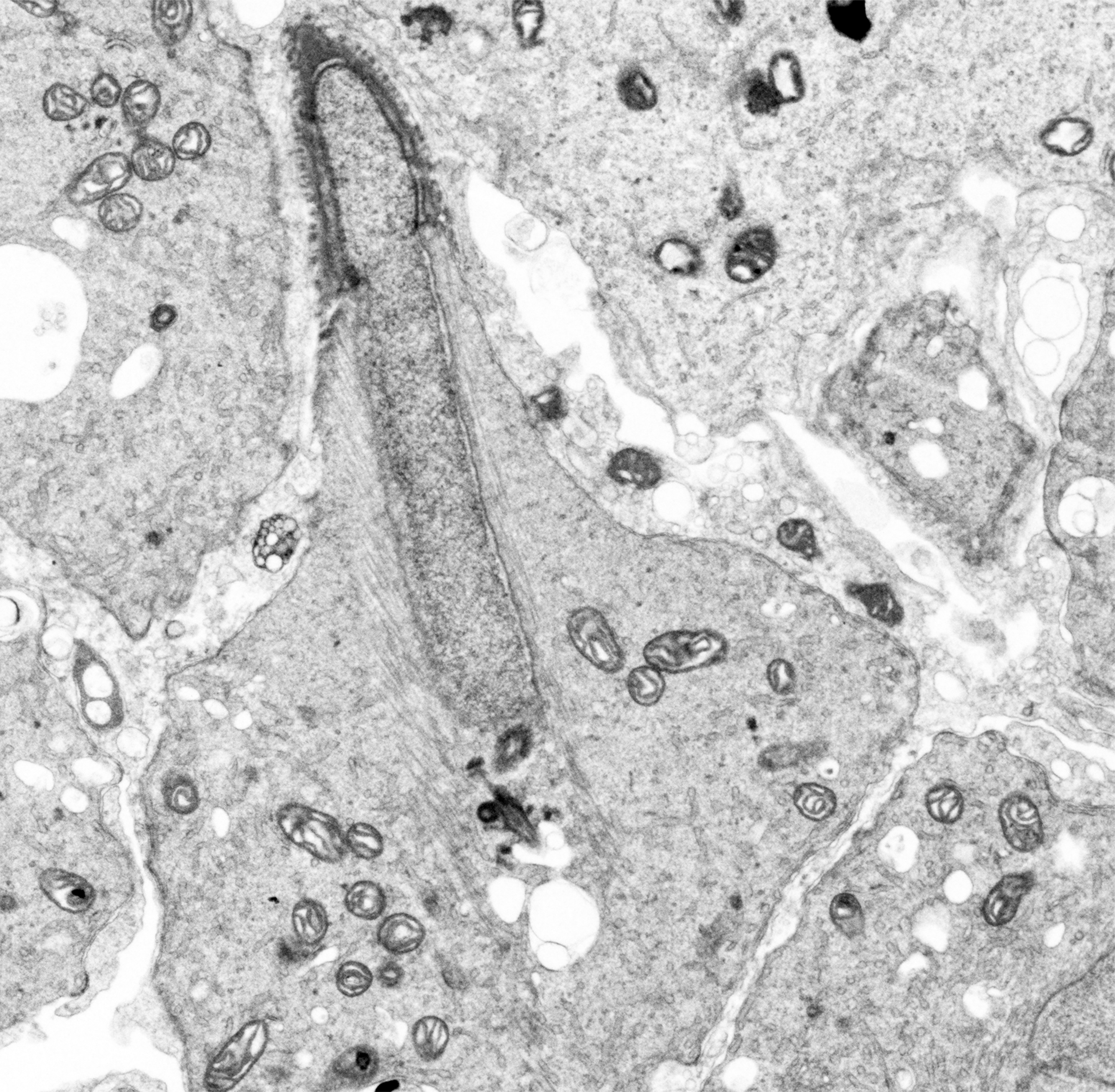

Supplement: Supplementary file 11 — Appendix and EV Figures Source Data [file 44319_2024_112_MOESM11_ESM.zip › Appendix Figure S1,S4,S5,S6/Appendix Figure S1/Appendix Figure S1/Manchette.tif]

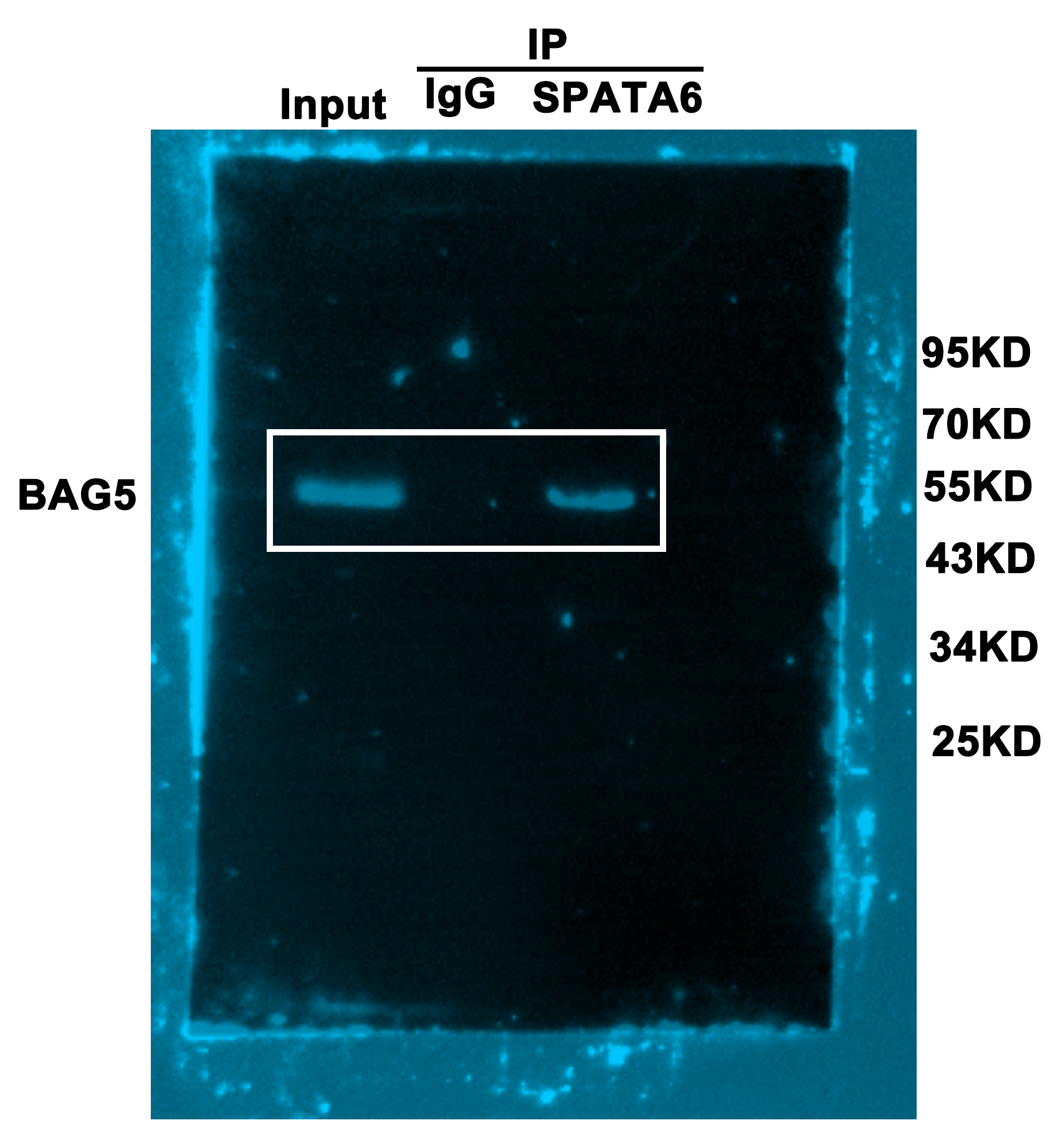

Supplement: Supplementary file 12 — Source Data Fig. 1 [file 44319_2024_112_MOESM12_ESM.zip › Figure 1/Figure 1/1A/WB BAG5.tif]

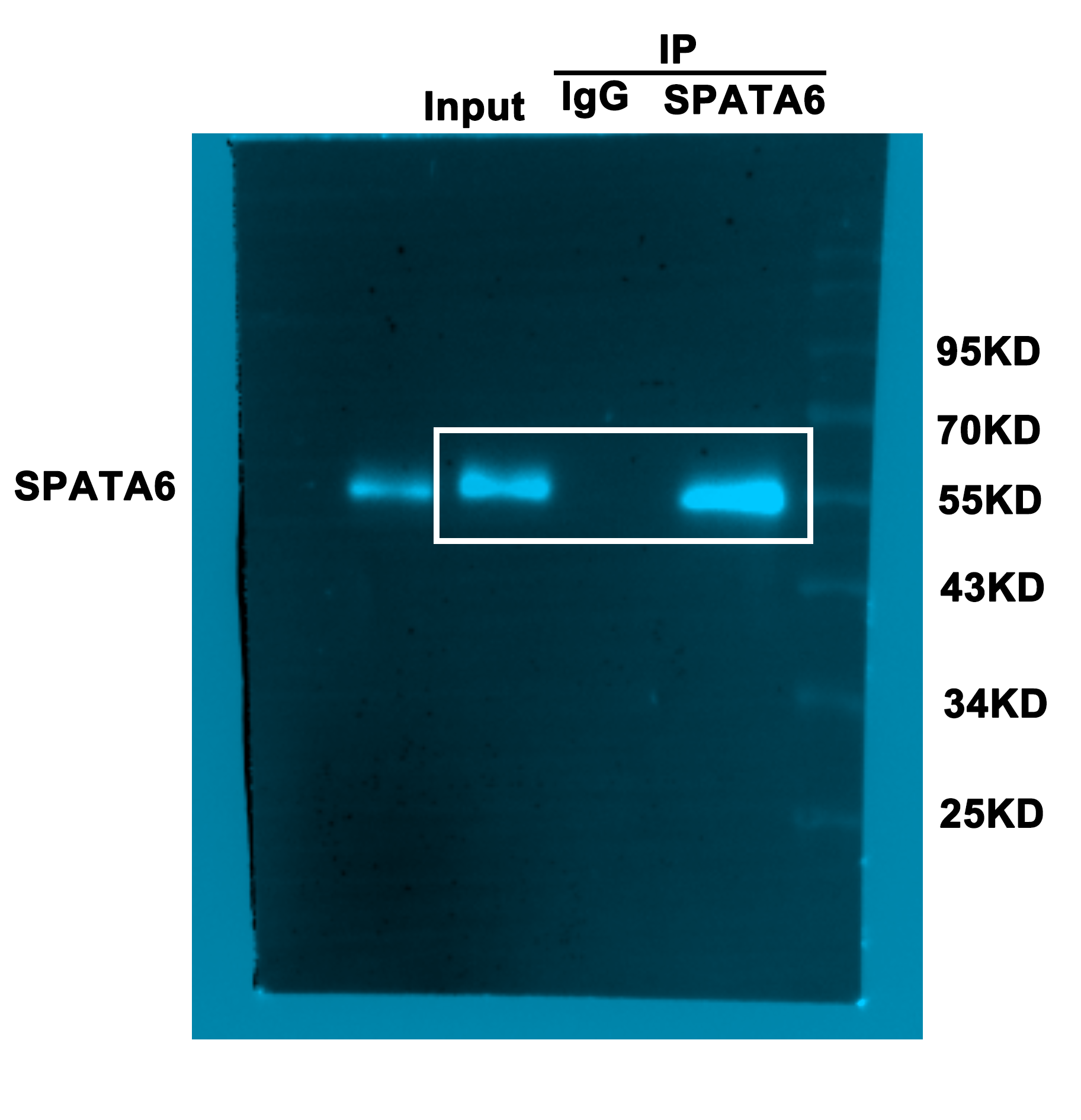

Supplement: Supplementary file 12 — Source Data Fig. 1 [file 44319_2024_112_MOESM12_ESM.zip › Figure 1/Figure 1/1A/WB SPATA6.tif]

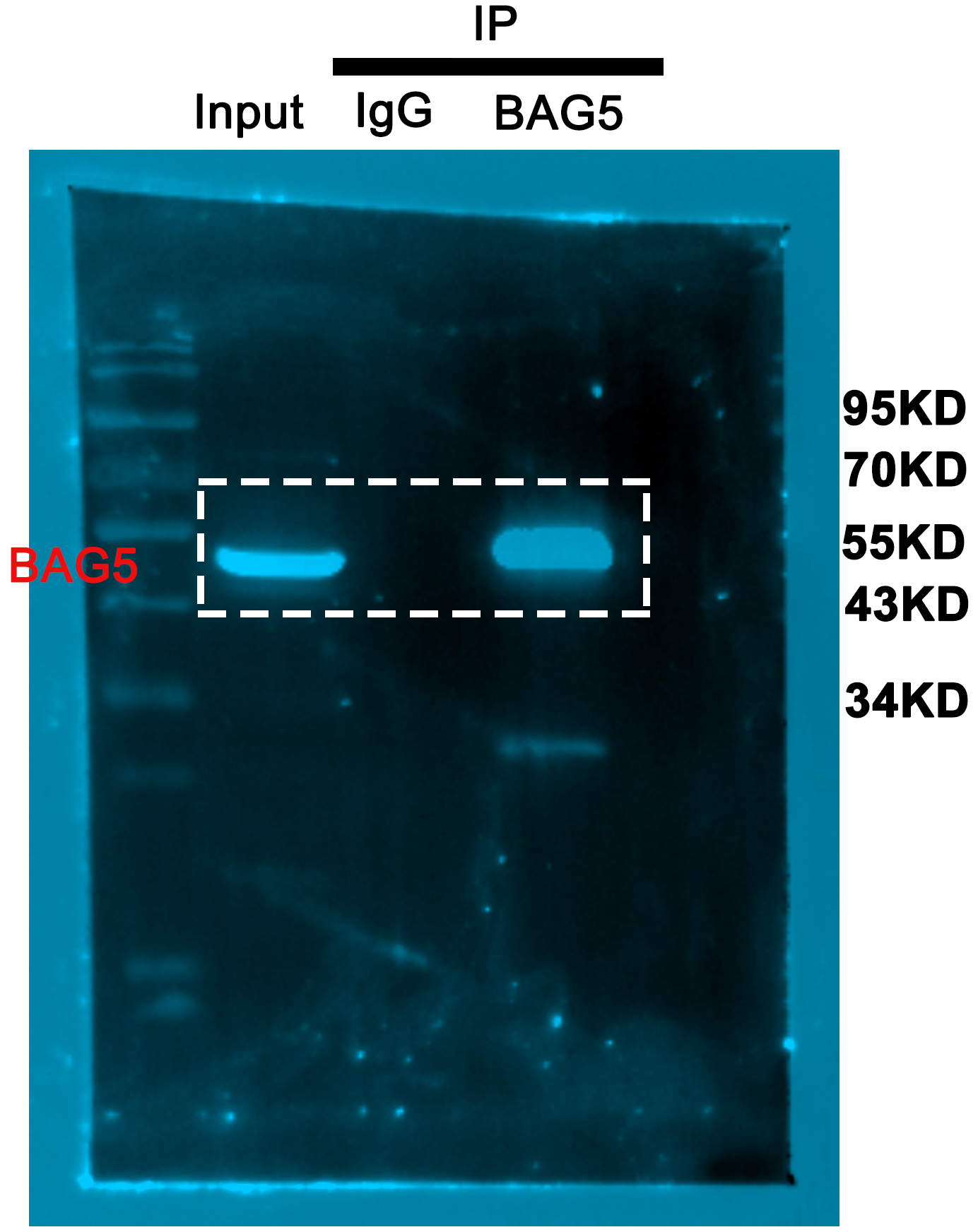

Supplement: Supplementary file 12 — Source Data Fig. 1 [file 44319_2024_112_MOESM12_ESM.zip › Figure 1/Figure 1/1B/WB BAG5.tif]

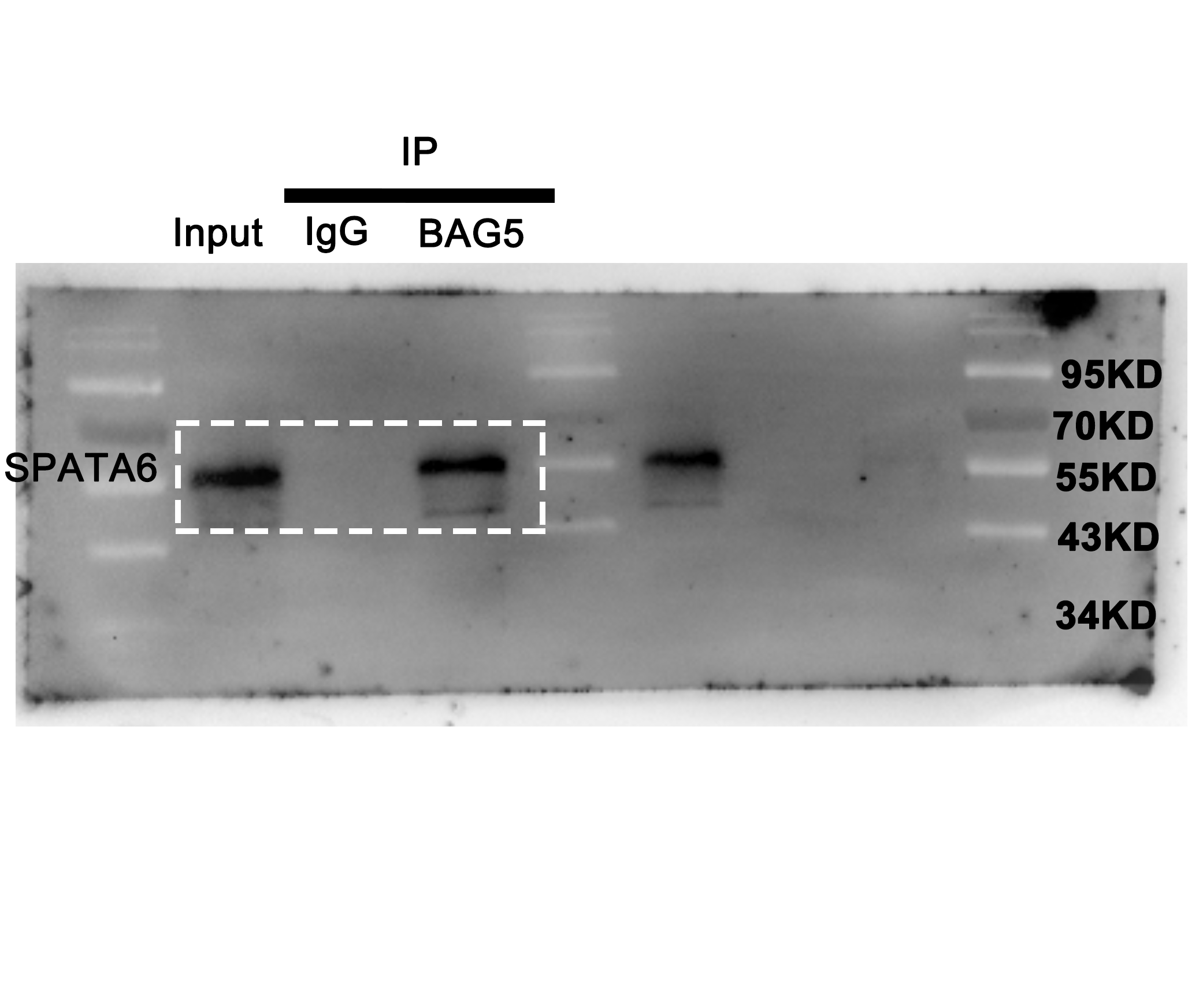

Supplement: Supplementary file 12 — Source Data Fig. 1 [file 44319_2024_112_MOESM12_ESM.zip › Figure 1/Figure 1/1B/WB SPATA6.tif]

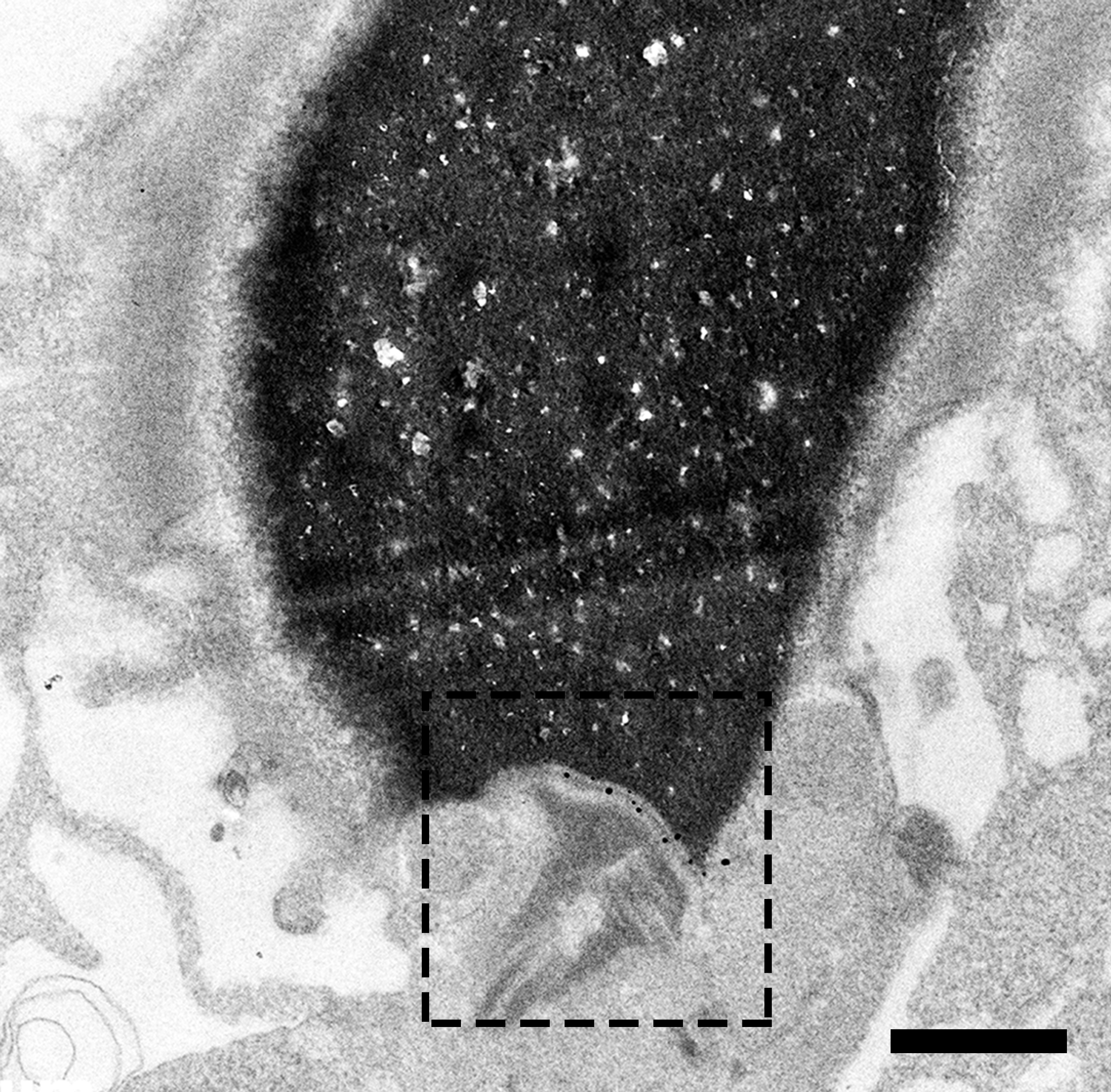

Supplement: Supplementary file 12 — Source Data Fig. 1 [file 44319_2024_112_MOESM12_ESM.zip › Figure 1/Figure 1/1E/Basal plate.tif]

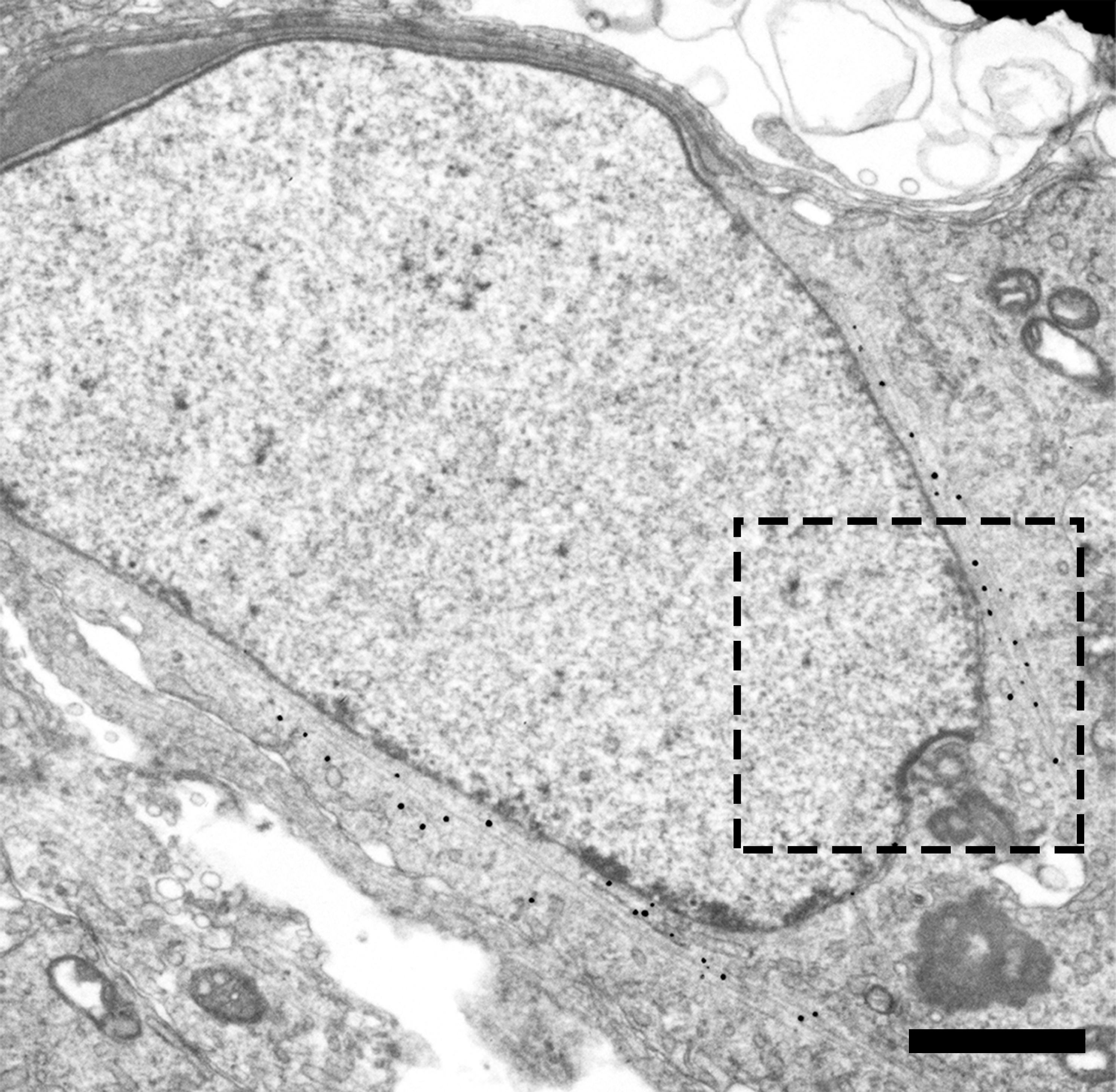

Supplement: Supplementary file 12 — Source Data Fig. 1 [file 44319_2024_112_MOESM12_ESM.zip › Figure 1/Figure 1/1E/Manchette.tif]

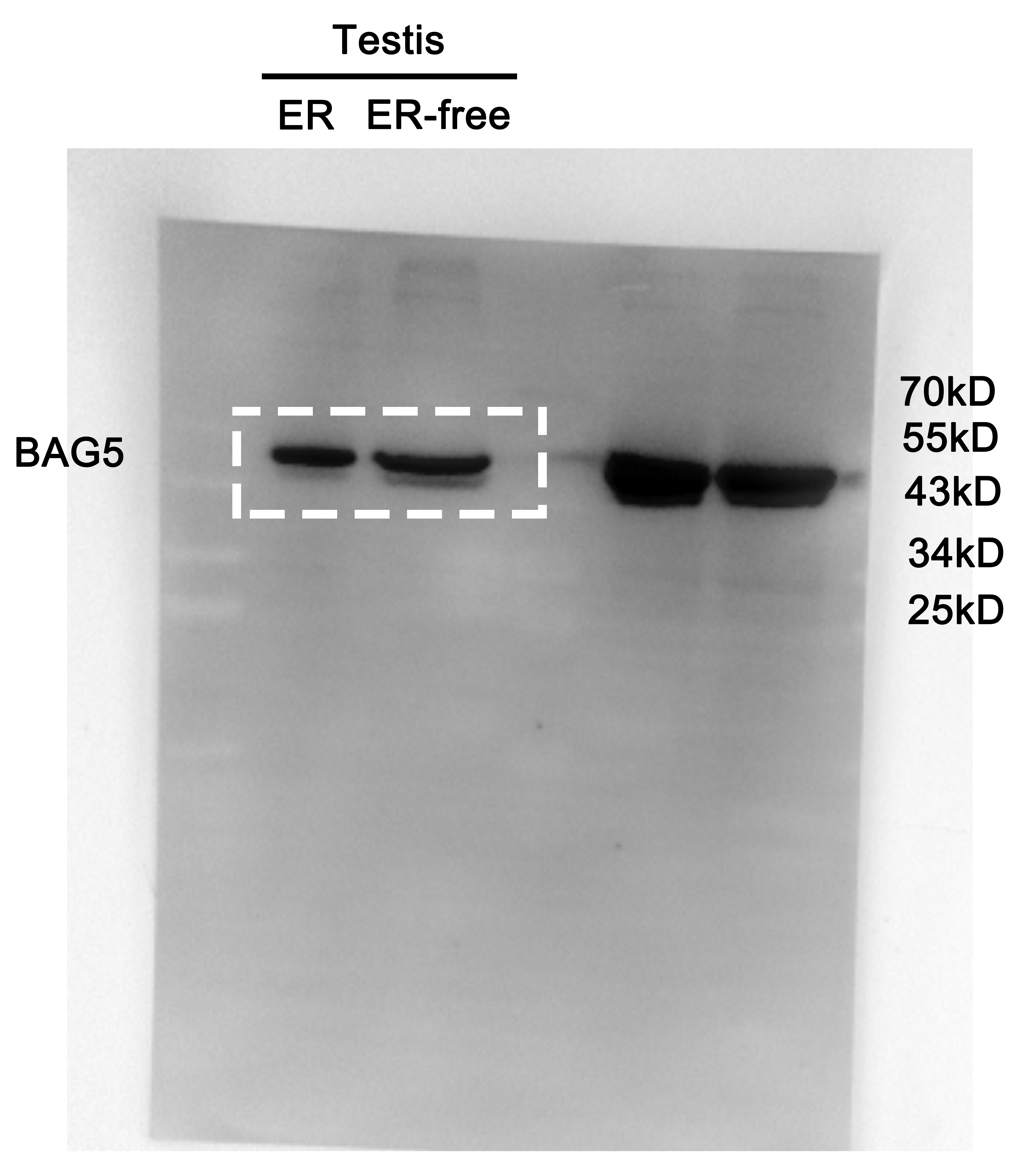

Supplement: Supplementary file 12 — Source Data Fig. 1 [file 44319_2024_112_MOESM12_ESM.zip › Figure 1/Figure 1/1I/WB BAG5.tif]

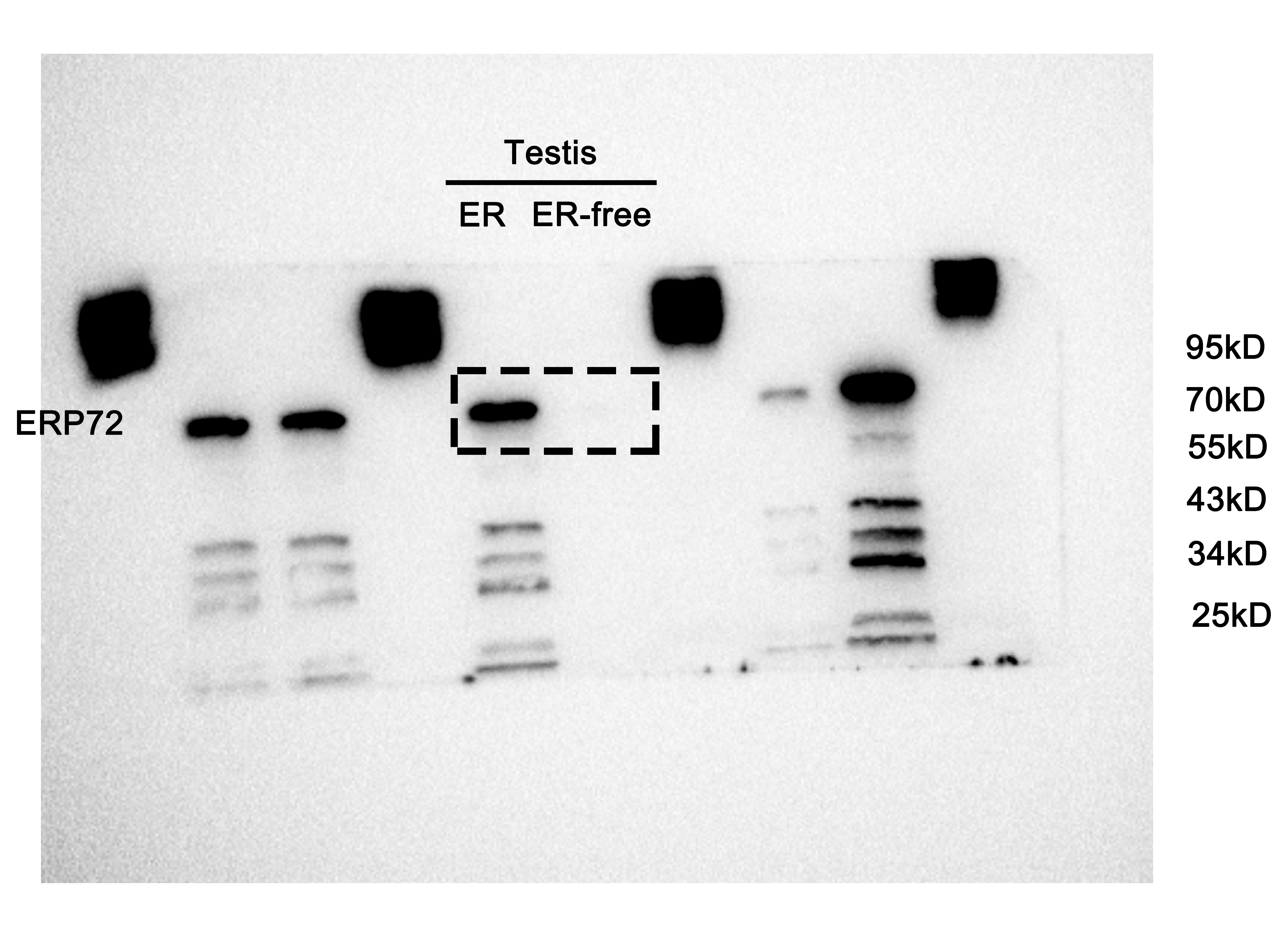

Supplement: Supplementary file 12 — Source Data Fig. 1 [file 44319_2024_112_MOESM12_ESM.zip › Figure 1/Figure 1/1I/WB ERP72.tif]

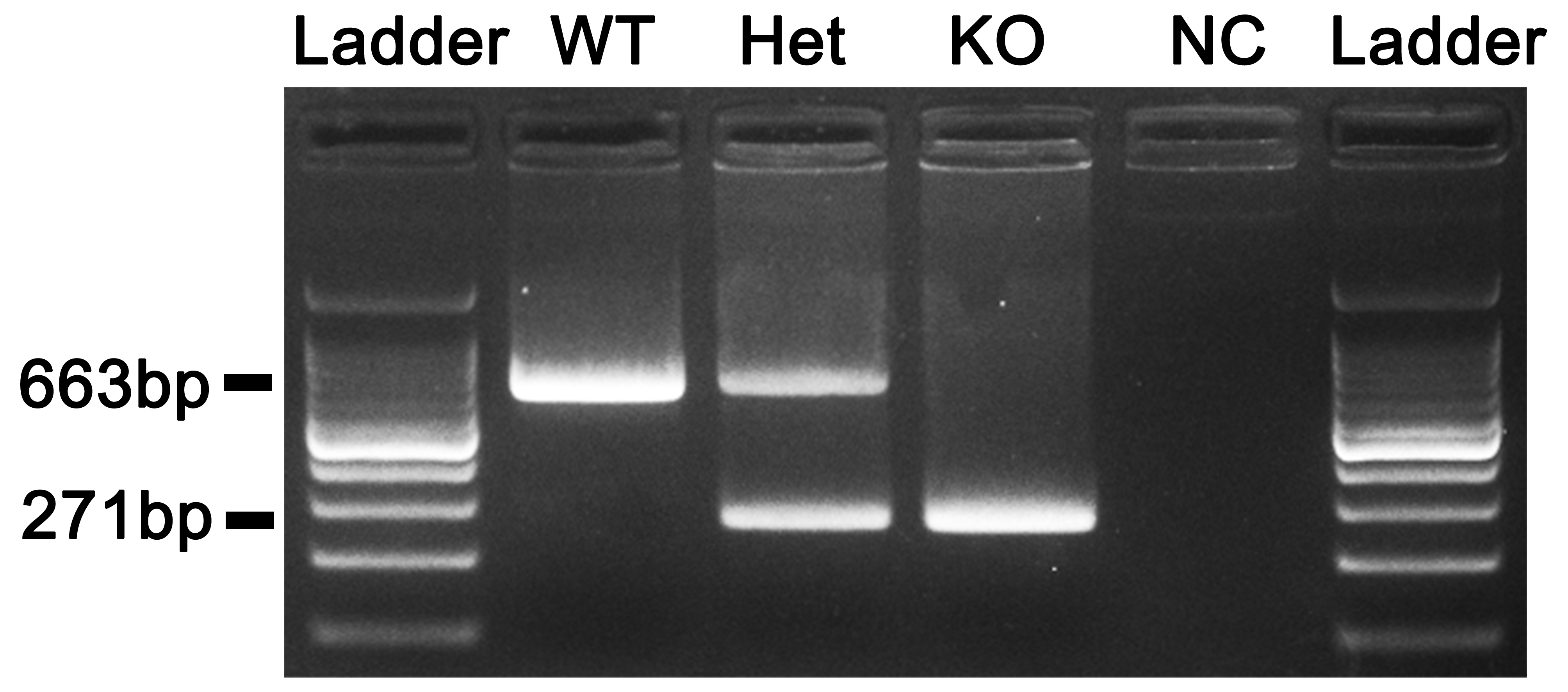

Supplement: Supplementary file 13 — Source Data Fig. 2 [file 44319_2024_112_MOESM13_ESM.zip › Figure 2/Figure 2/2B/Image gel.tif]

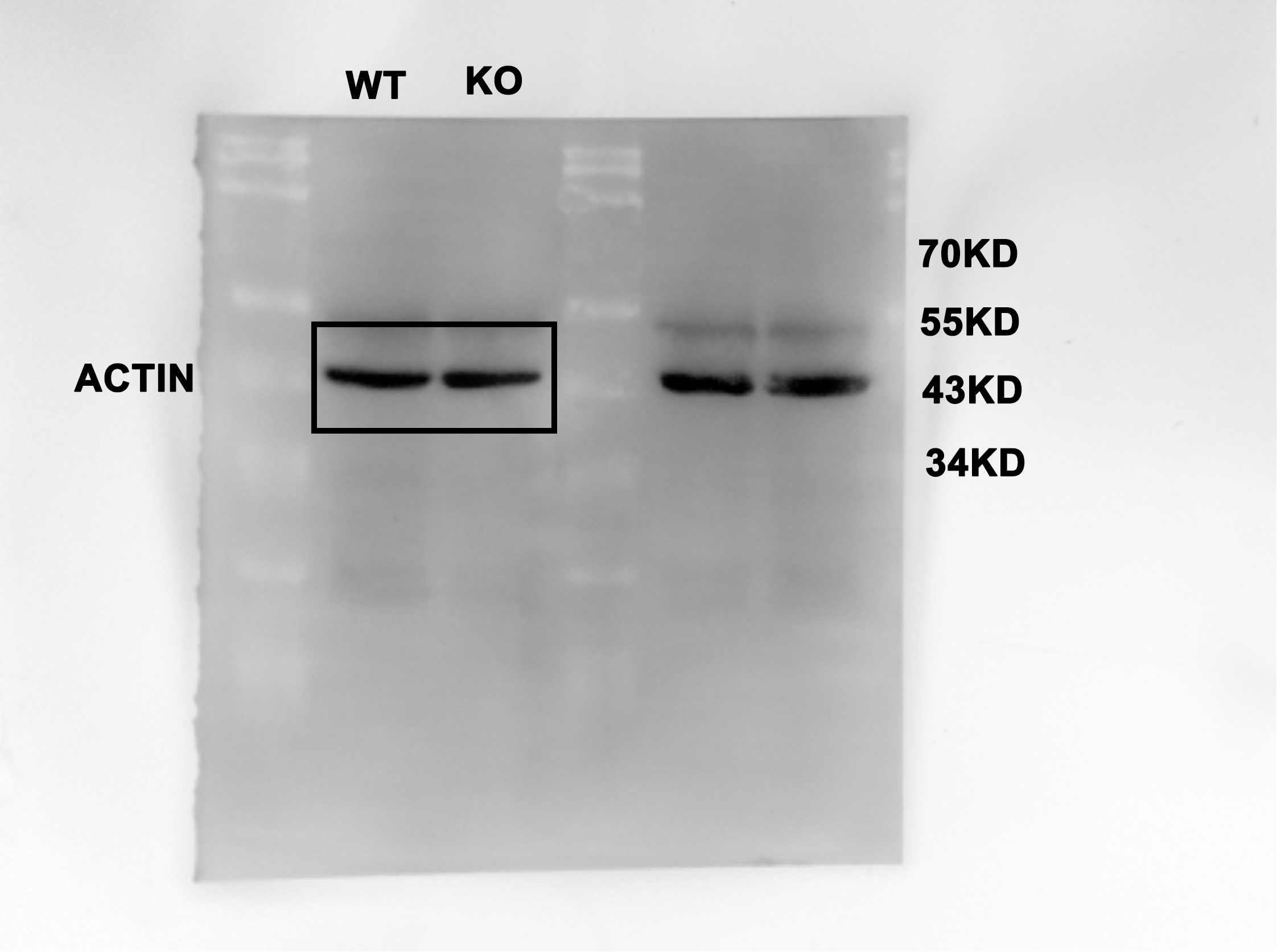

Supplement: Supplementary file 13 — Source Data Fig. 2 [file 44319_2024_112_MOESM13_ESM.zip › Figure 2/Figure 2/2D/WB ACTIN.tif]

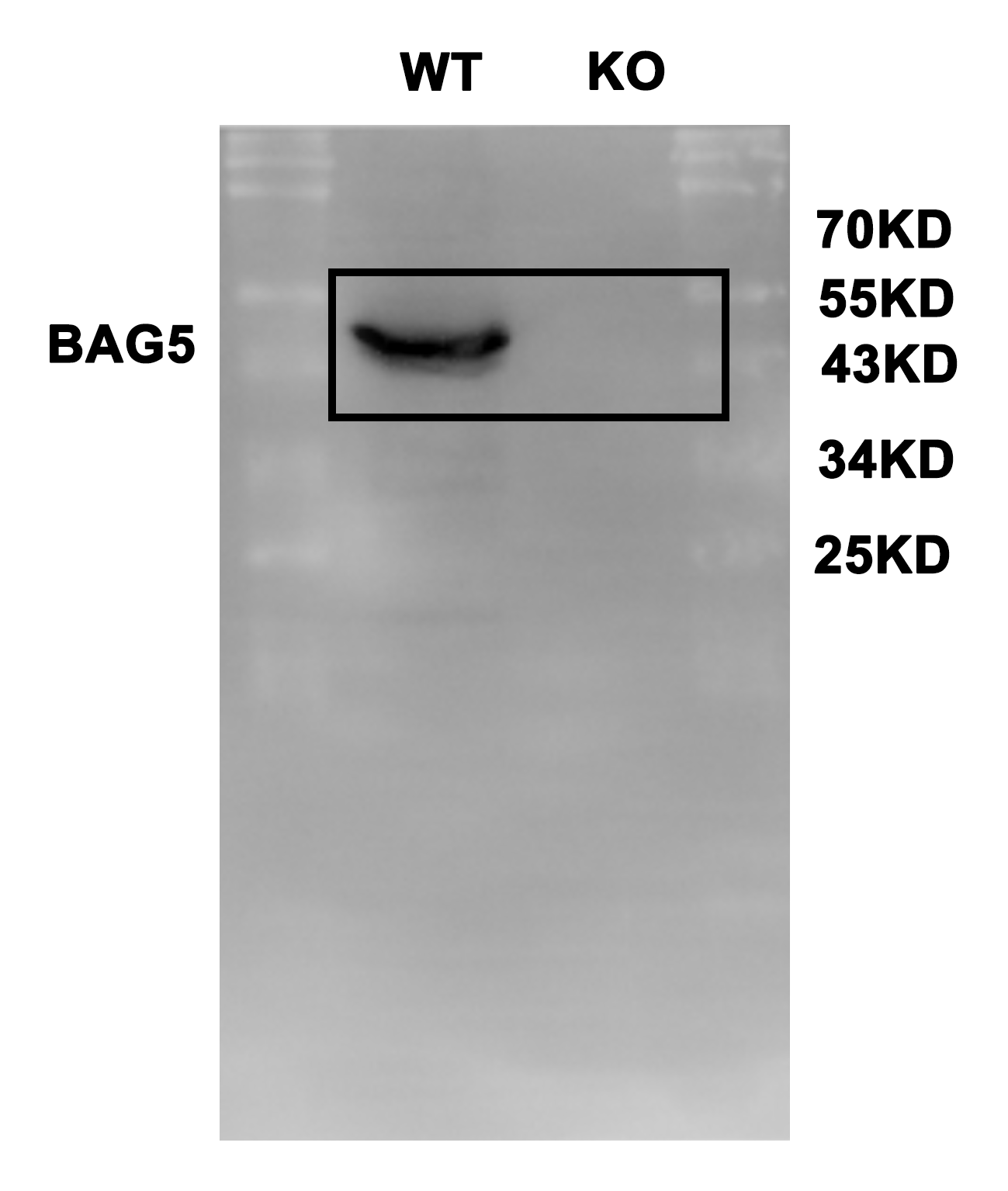

Supplement: Supplementary file 13 — Source Data Fig. 2 [file 44319_2024_112_MOESM13_ESM.zip › Figure 2/Figure 2/2D/WB BAG5.tif]

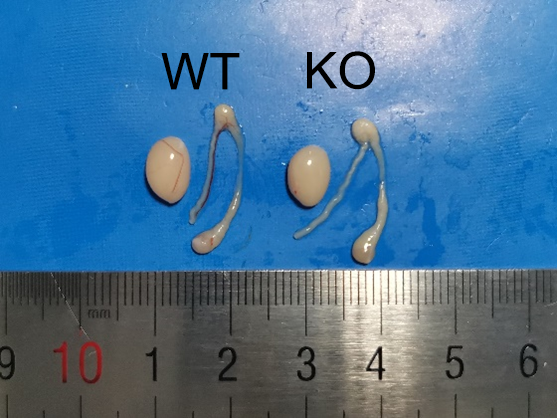

Supplement: Supplementary file 13 — Source Data Fig. 2 [file 44319_2024_112_MOESM13_ESM.zip › Figure 2/Figure 2/2G/Image.tif]

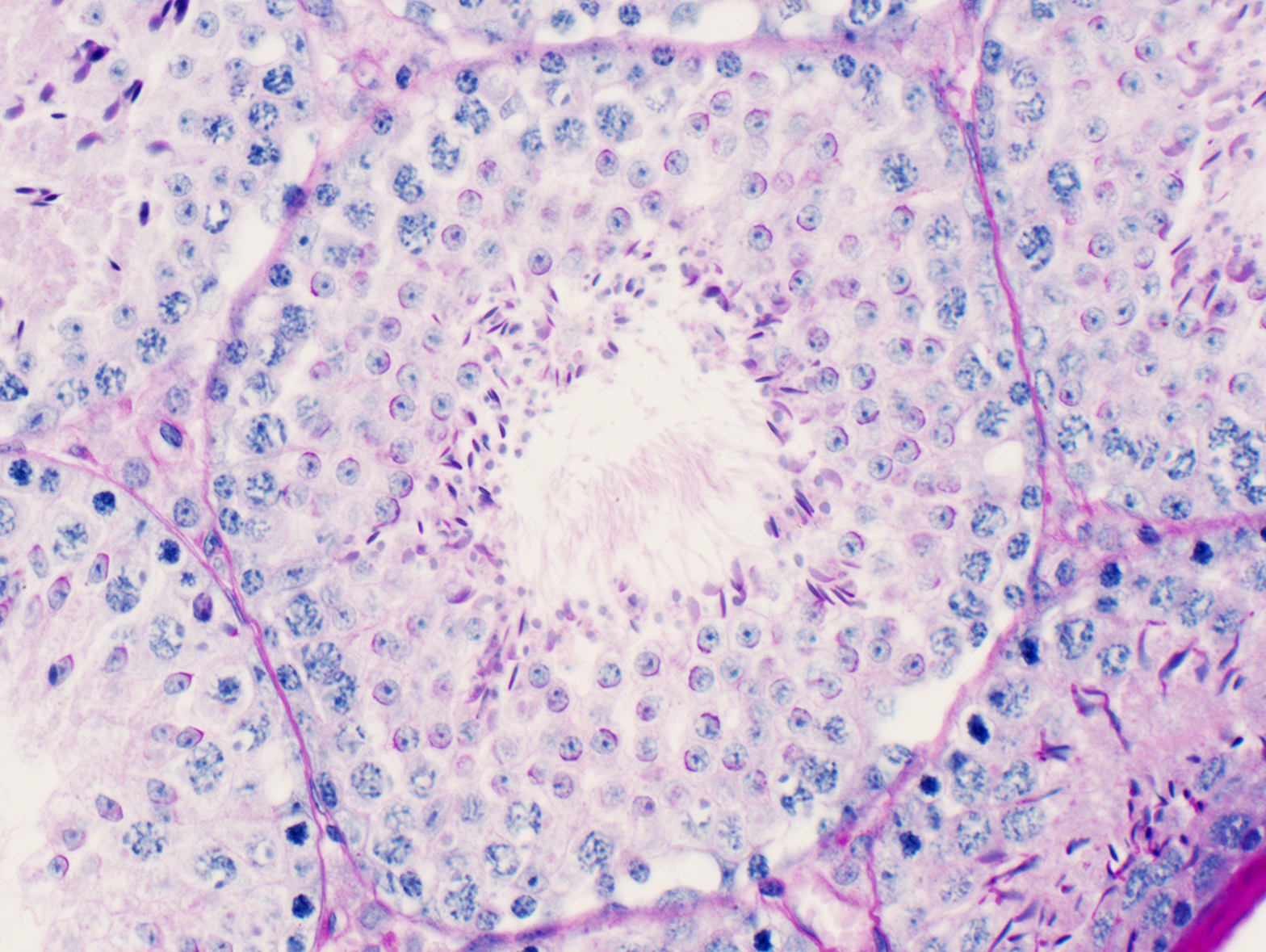

Supplement: Supplementary file 13 — Source Data Fig. 2 [file 44319_2024_112_MOESM13_ESM.zip › Figure 2/Figure 2/2J/KO.tif]

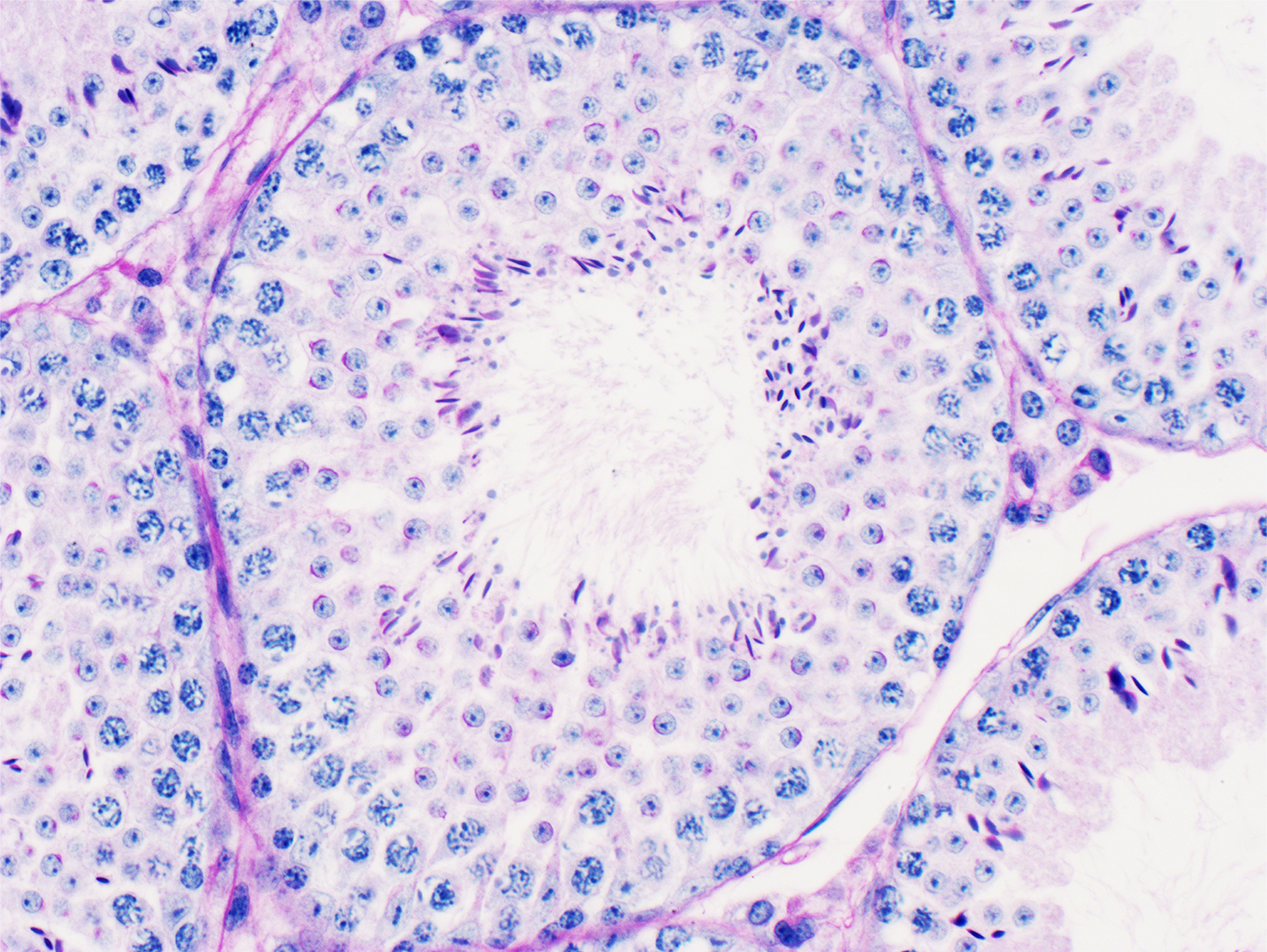

Supplement: Supplementary file 13 — Source Data Fig. 2 [file 44319_2024_112_MOESM13_ESM.zip › Figure 2/Figure 2/2J/WT.tif]

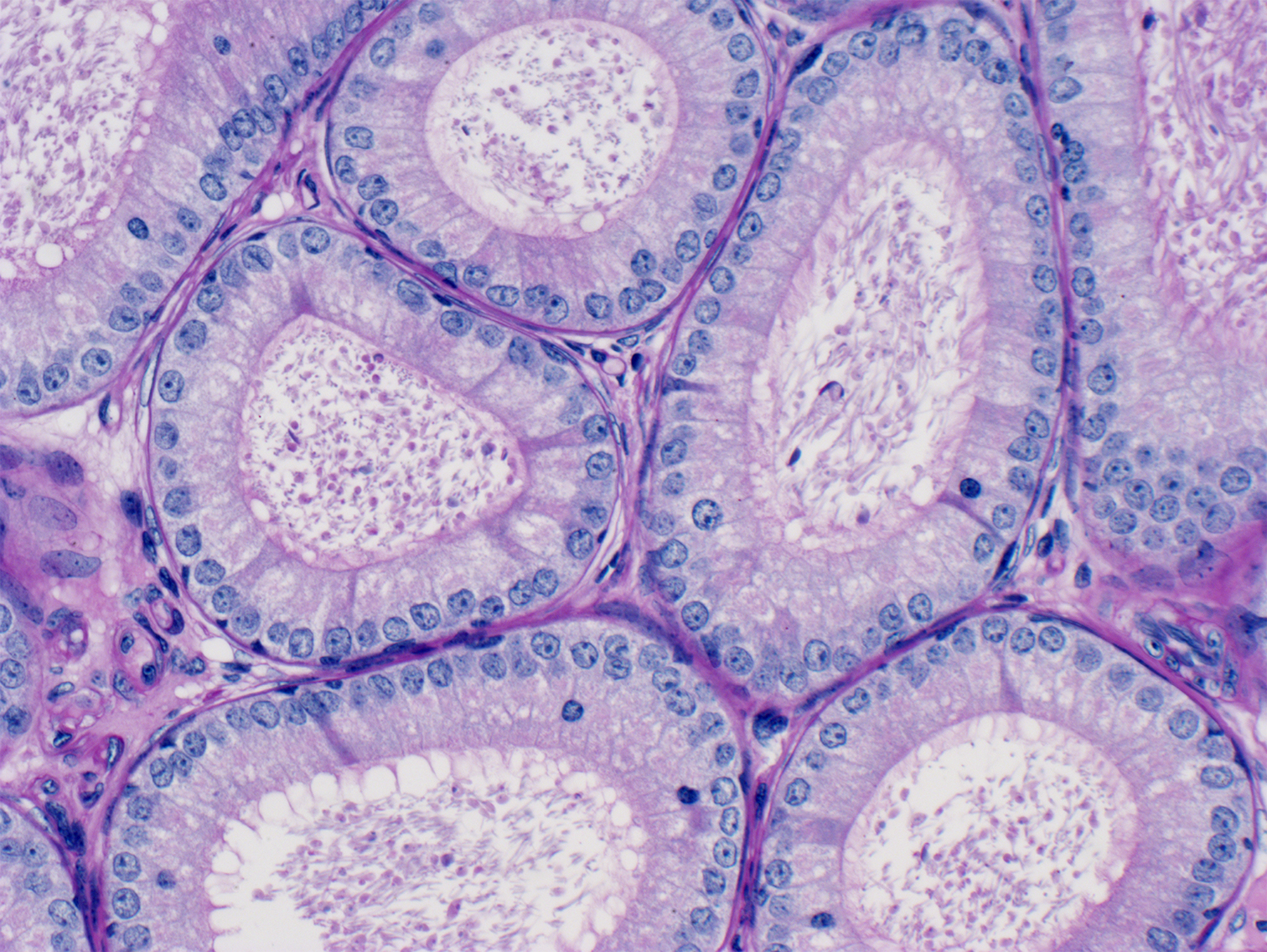

Supplement: Supplementary file 14 — Source Data Fig. 3 [file 44319_2024_112_MOESM14_ESM.zip › Figure 3/Figure 3/3A/KO Caput.tif]

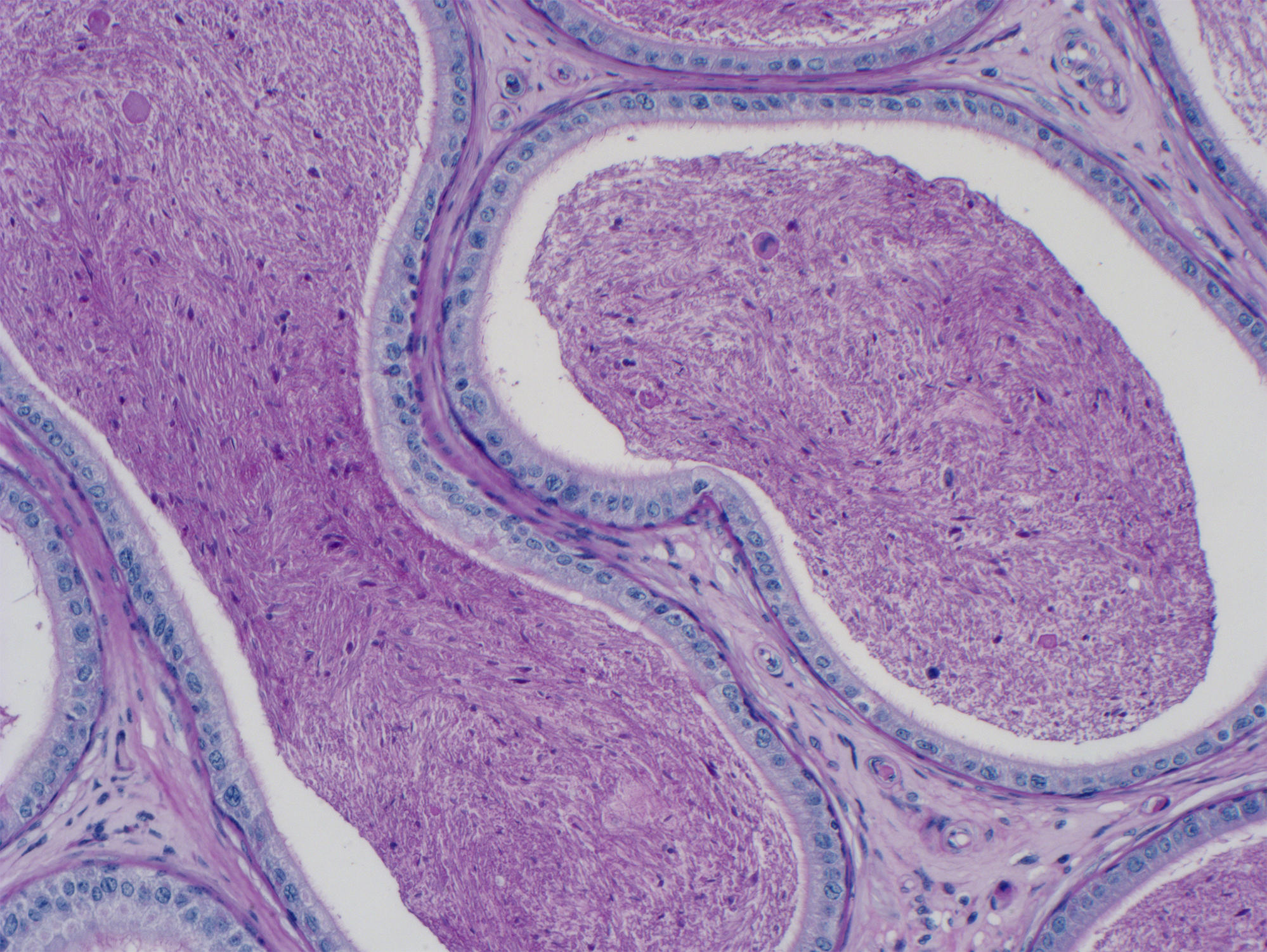

Supplement: Supplementary file 14 — Source Data Fig. 3 [file 44319_2024_112_MOESM14_ESM.zip › Figure 3/Figure 3/3A/KO Cauda.tif]

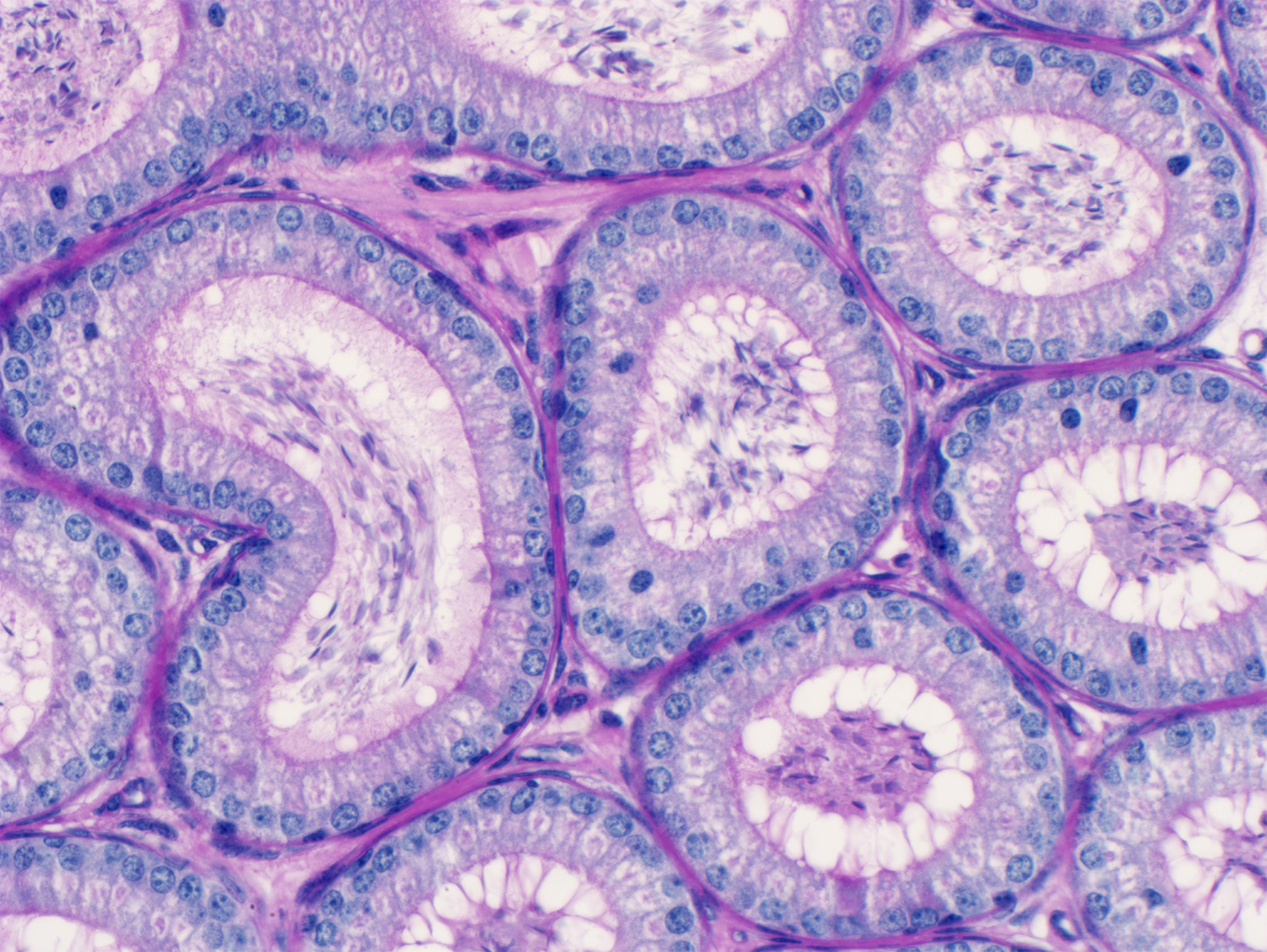

Supplement: Supplementary file 14 — Source Data Fig. 3 [file 44319_2024_112_MOESM14_ESM.zip › Figure 3/Figure 3/3A/WT Caput.tif]
