# Supplementary figures and images for: BAG5 regulates HSPA8-mediated protein folding required for sperm head-tail coupling apparatus assembly (part 2 of 2)
Source: EMBO Rep. 2024 Mar 7;25(4):23. doi: 10.1038/s44319-024-00112-x (PMC11015022; doi:10.1038/s44319-024-00112-x)

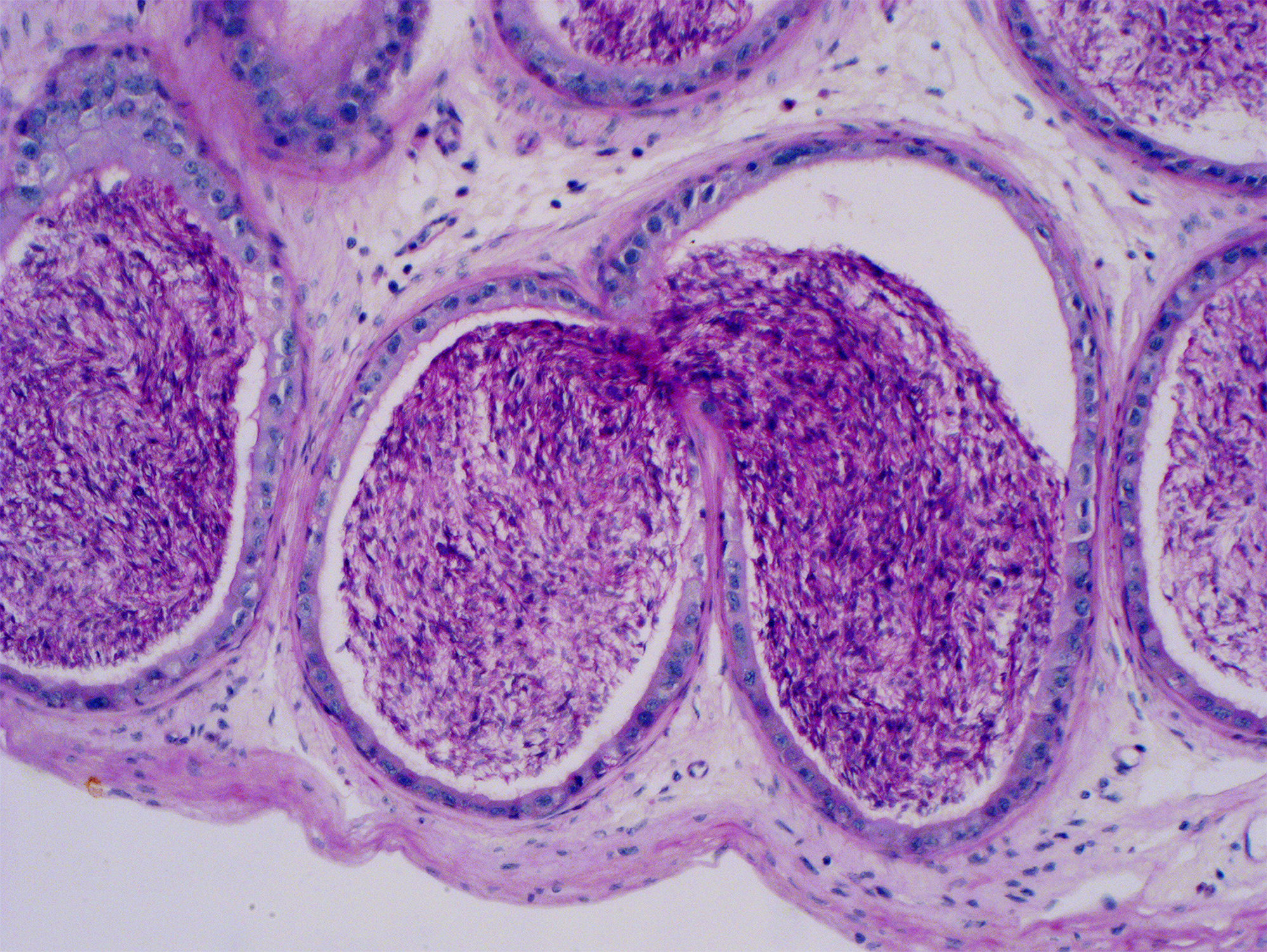

Supplement: Supplementary file 14 — Source Data Fig. 3 [file 44319_2024_112_MOESM14_ESM.zip › Figure 3/Figure 3/3A/WT Cauda.tif]

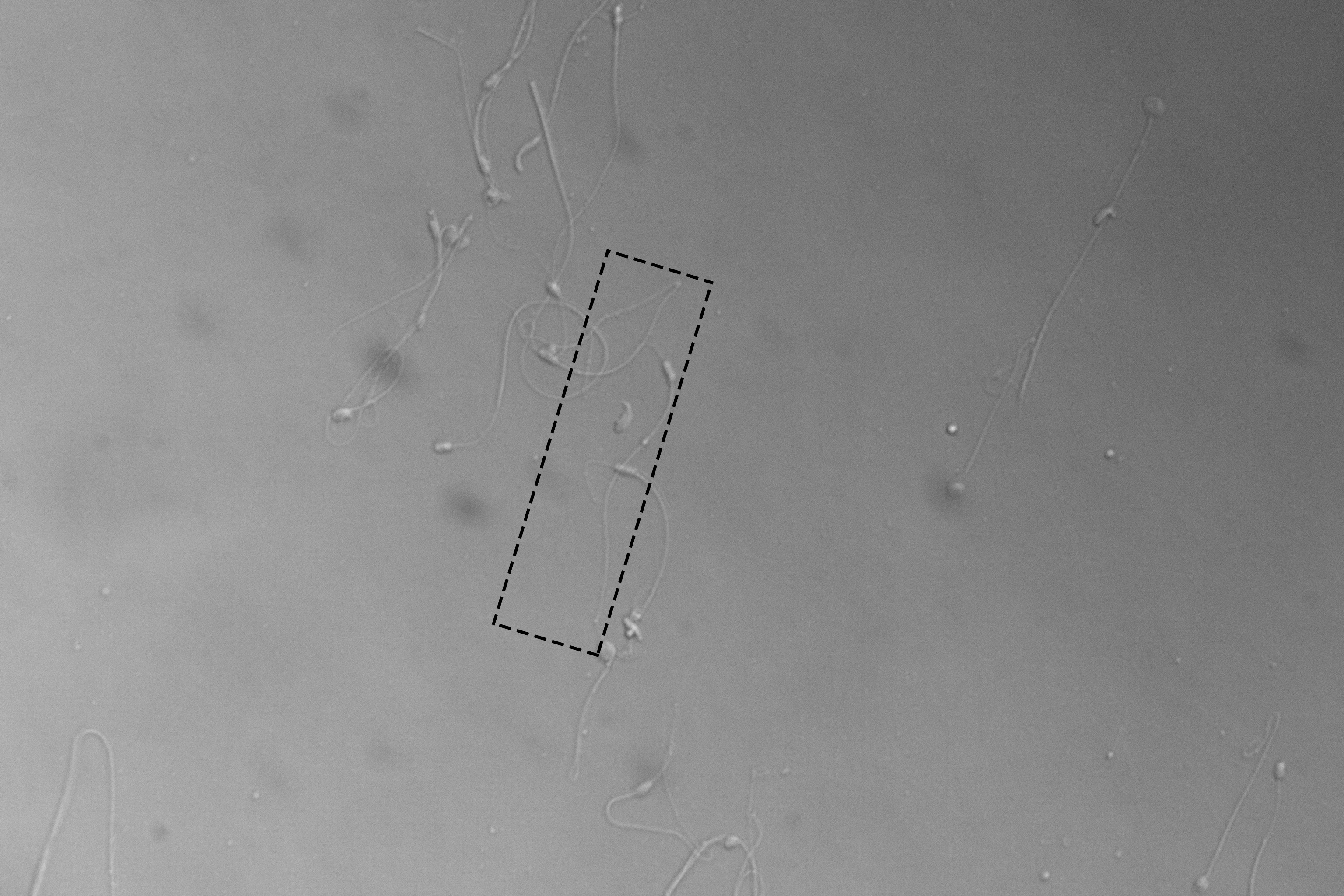

Supplement: Supplementary file 14 — Source Data Fig. 3 [file 44319_2024_112_MOESM14_ESM.zip › Figure 3/Figure 3/3B/KO-DIC.tif]

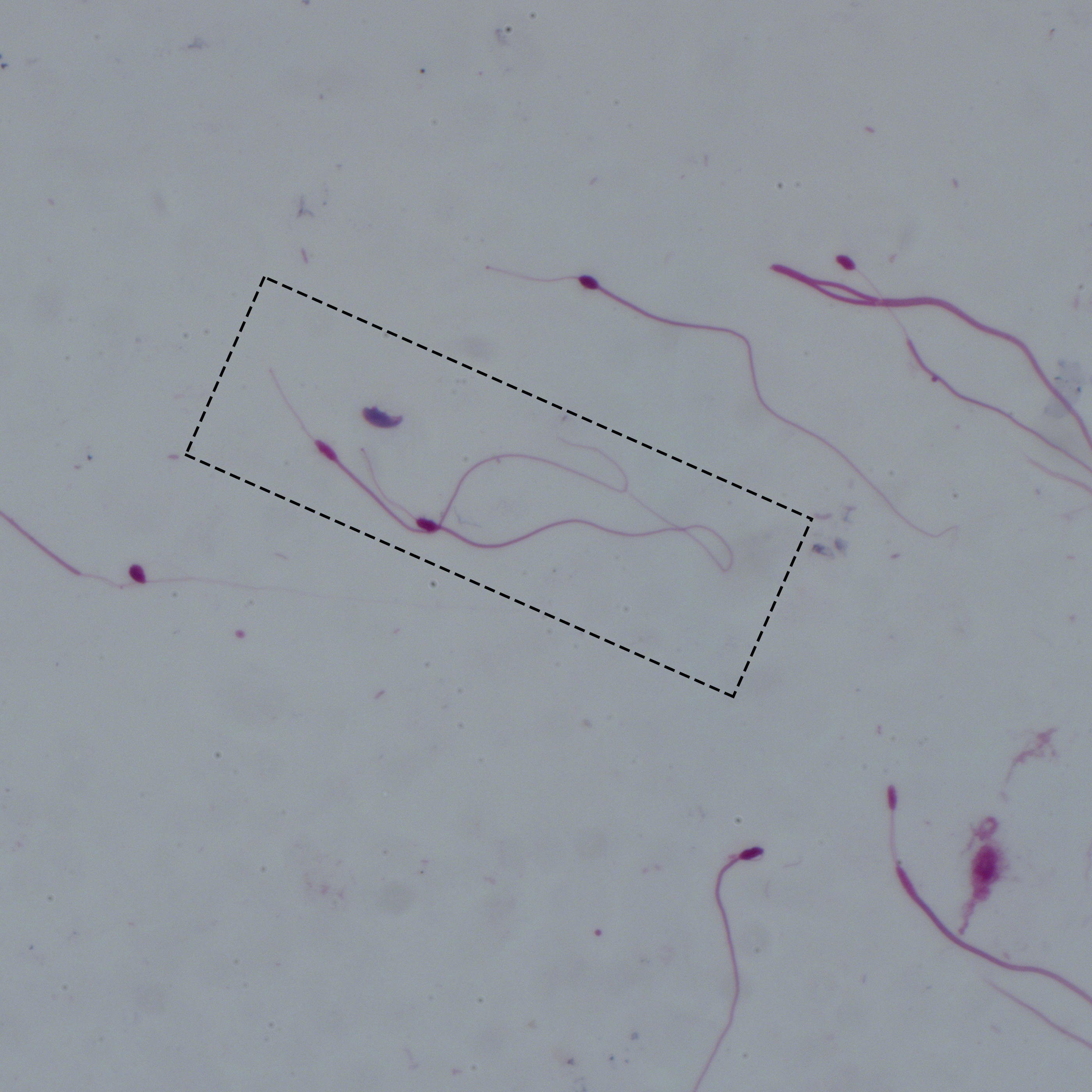

Supplement: Supplementary file 14 — Source Data Fig. 3 [file 44319_2024_112_MOESM14_ESM.zip › Figure 3/Figure 3/3B/KO-HE.tif]

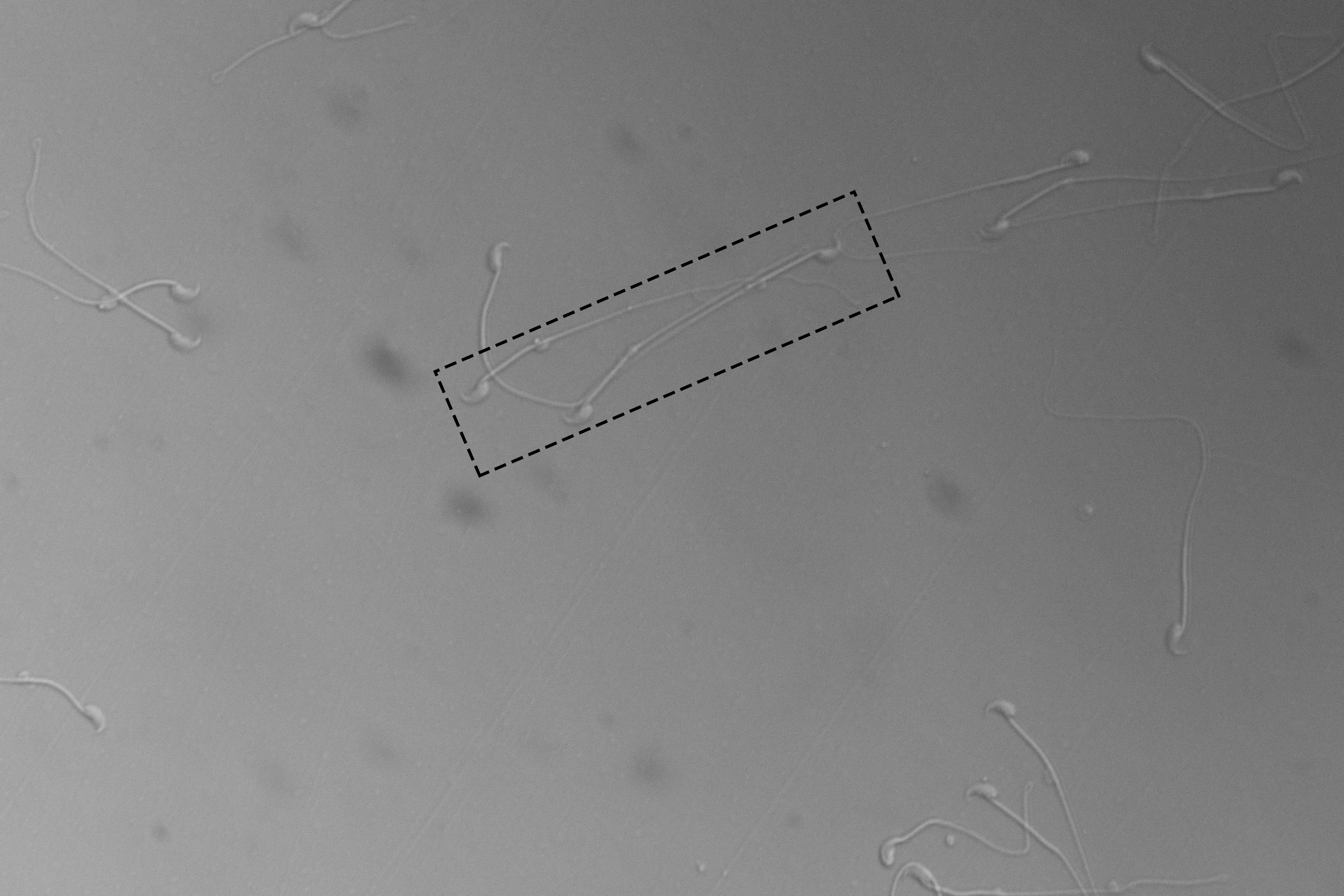

Supplement: Supplementary file 14 — Source Data Fig. 3 [file 44319_2024_112_MOESM14_ESM.zip › Figure 3/Figure 3/3B/WT-DIC.tif]

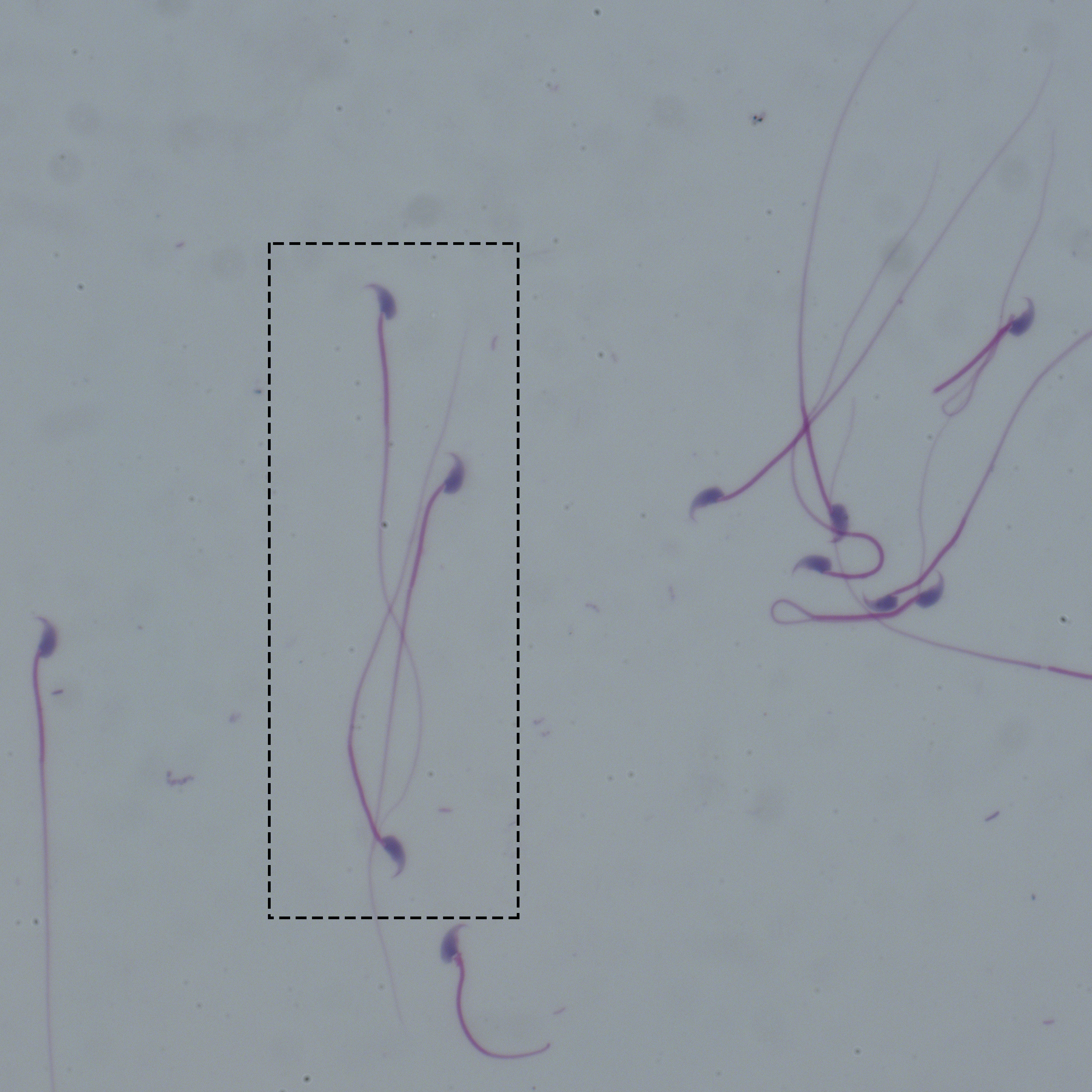

Supplement: Supplementary file 14 — Source Data Fig. 3 [file 44319_2024_112_MOESM14_ESM.zip › Figure 3/Figure 3/3B/WT-HE.tif]

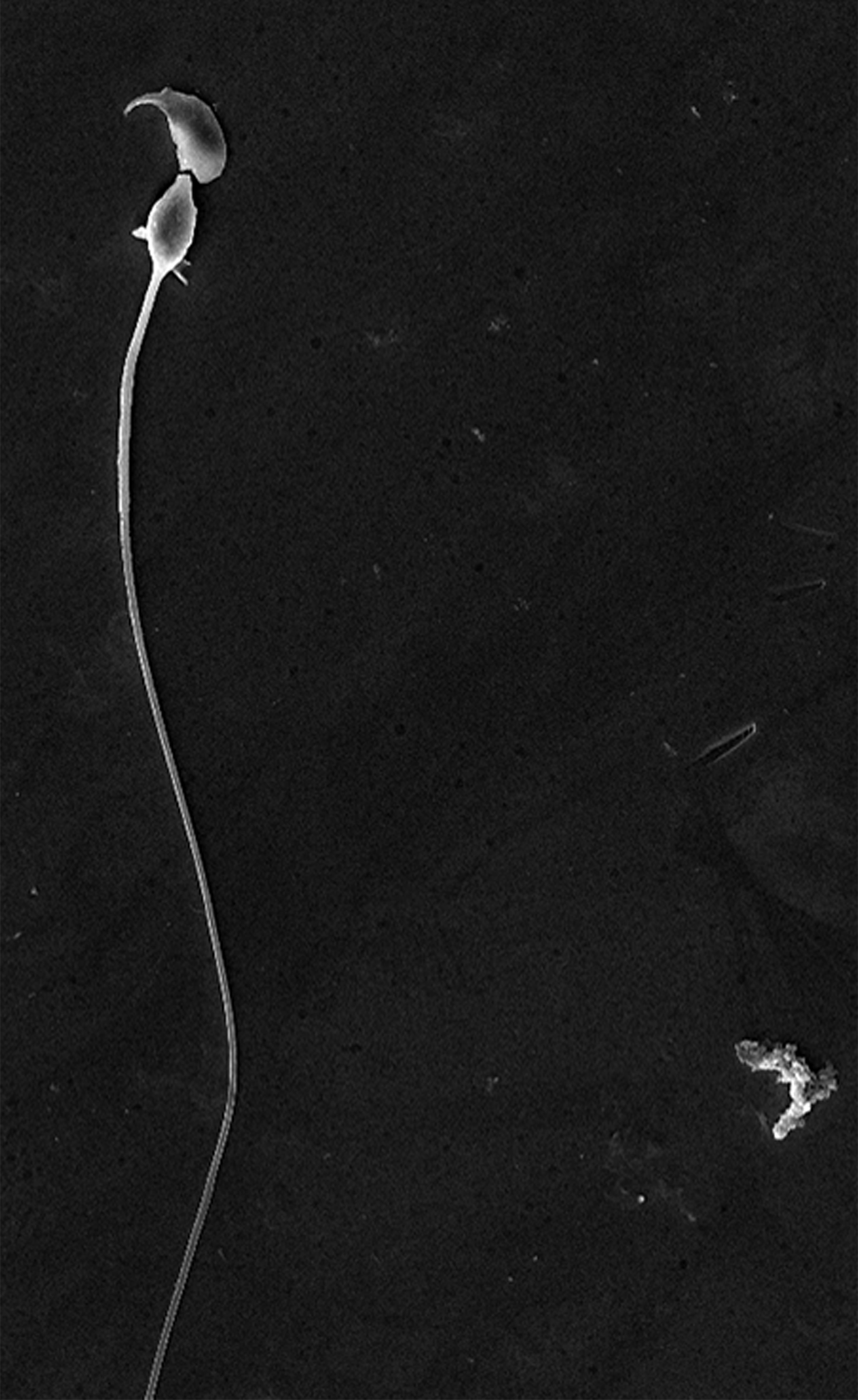

Supplement: Supplementary file 14 — Source Data Fig. 3 [file 44319_2024_112_MOESM14_ESM.zip › Figure 3/Figure 3/3E/KO#1.tif]

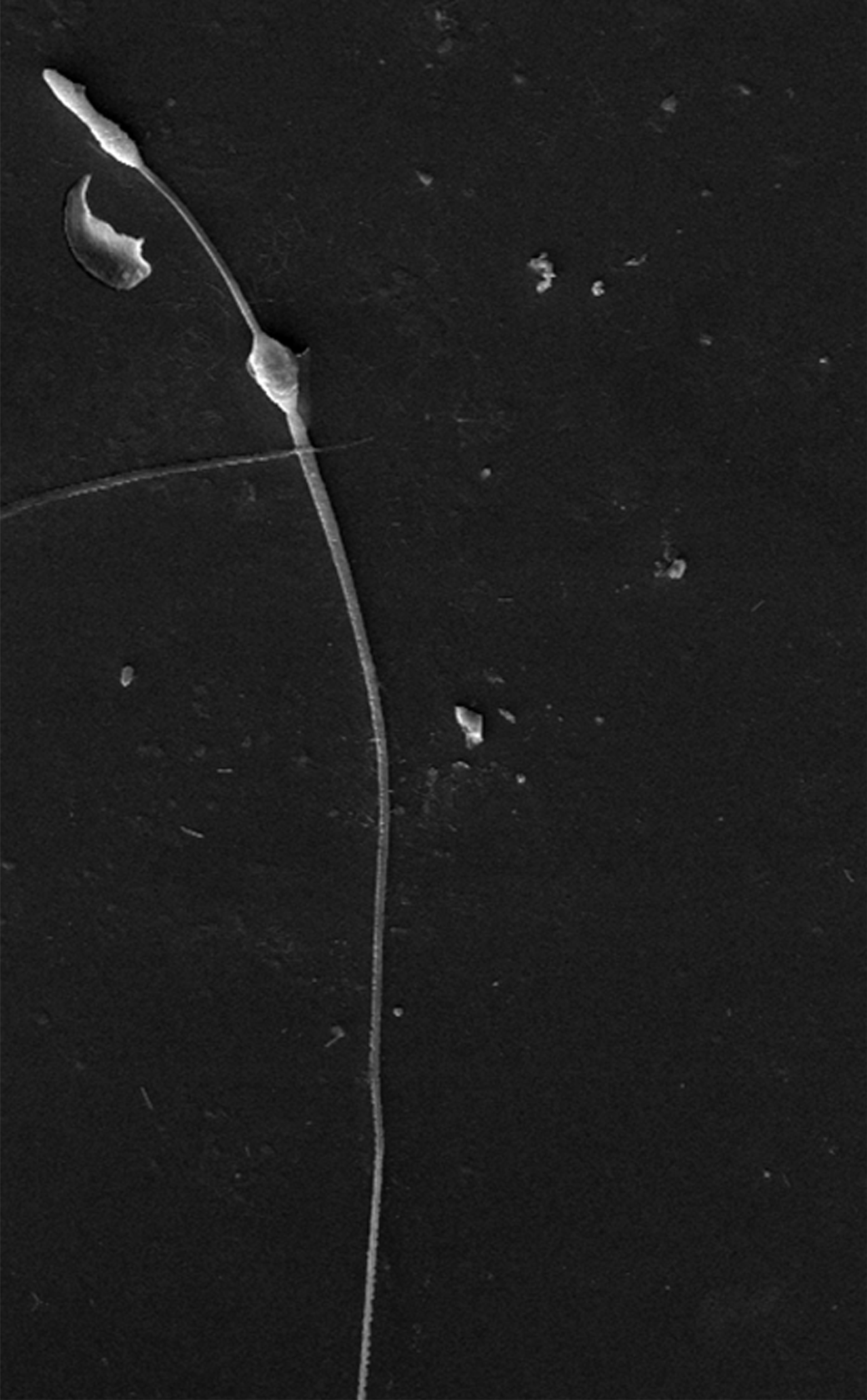

Supplement: Supplementary file 14 — Source Data Fig. 3 [file 44319_2024_112_MOESM14_ESM.zip › Figure 3/Figure 3/3E/KO#2.tif]

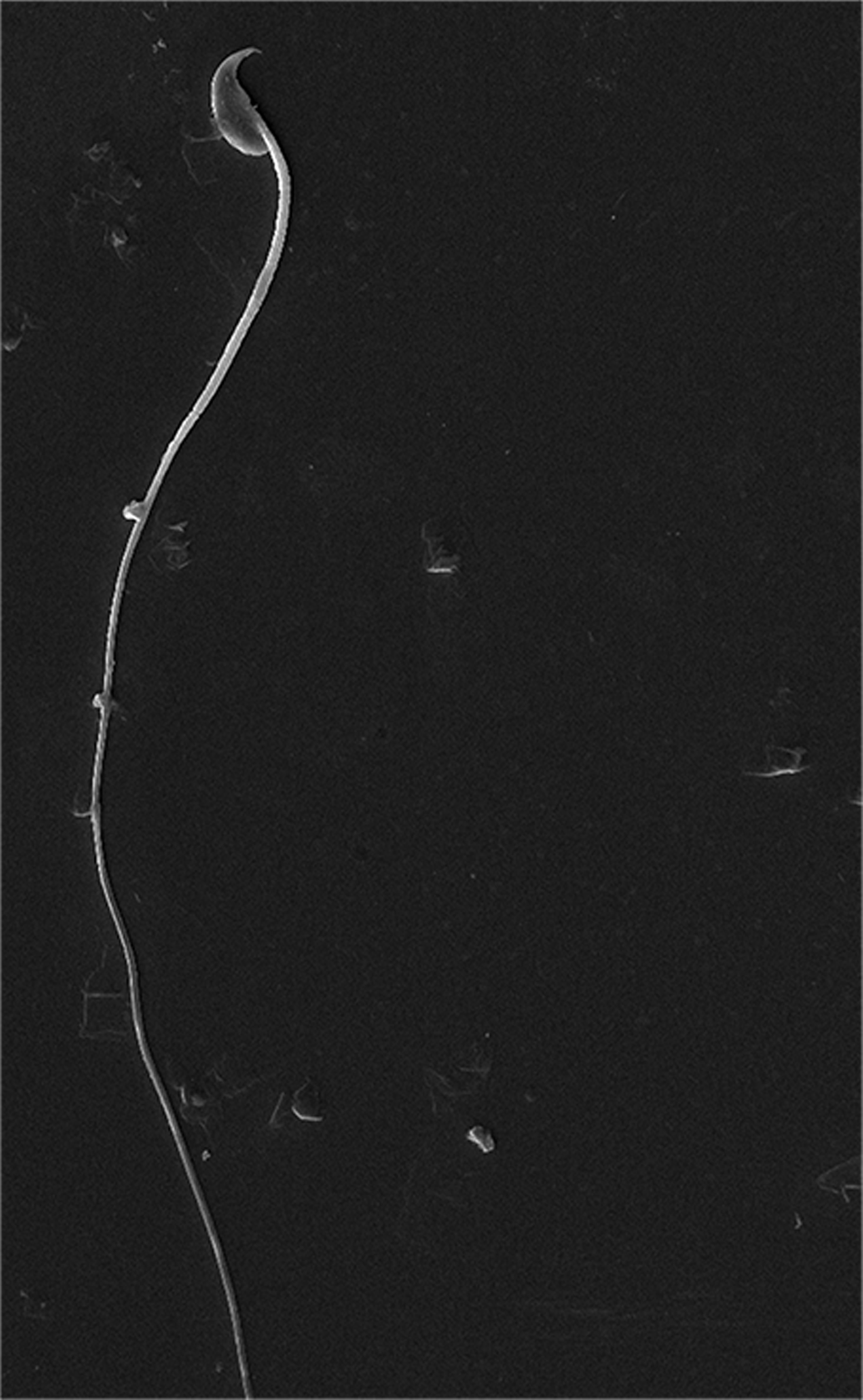

Supplement: Supplementary file 14 — Source Data Fig. 3 [file 44319_2024_112_MOESM14_ESM.zip › Figure 3/Figure 3/3E/WT.tif]

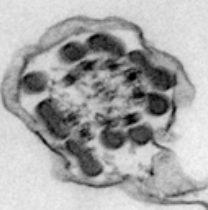

Supplement: Supplementary file 14 — Source Data Fig. 3 [file 44319_2024_112_MOESM14_ESM.zip › Figure 3/Figure 3/3H/KO MIDPIECE.png]

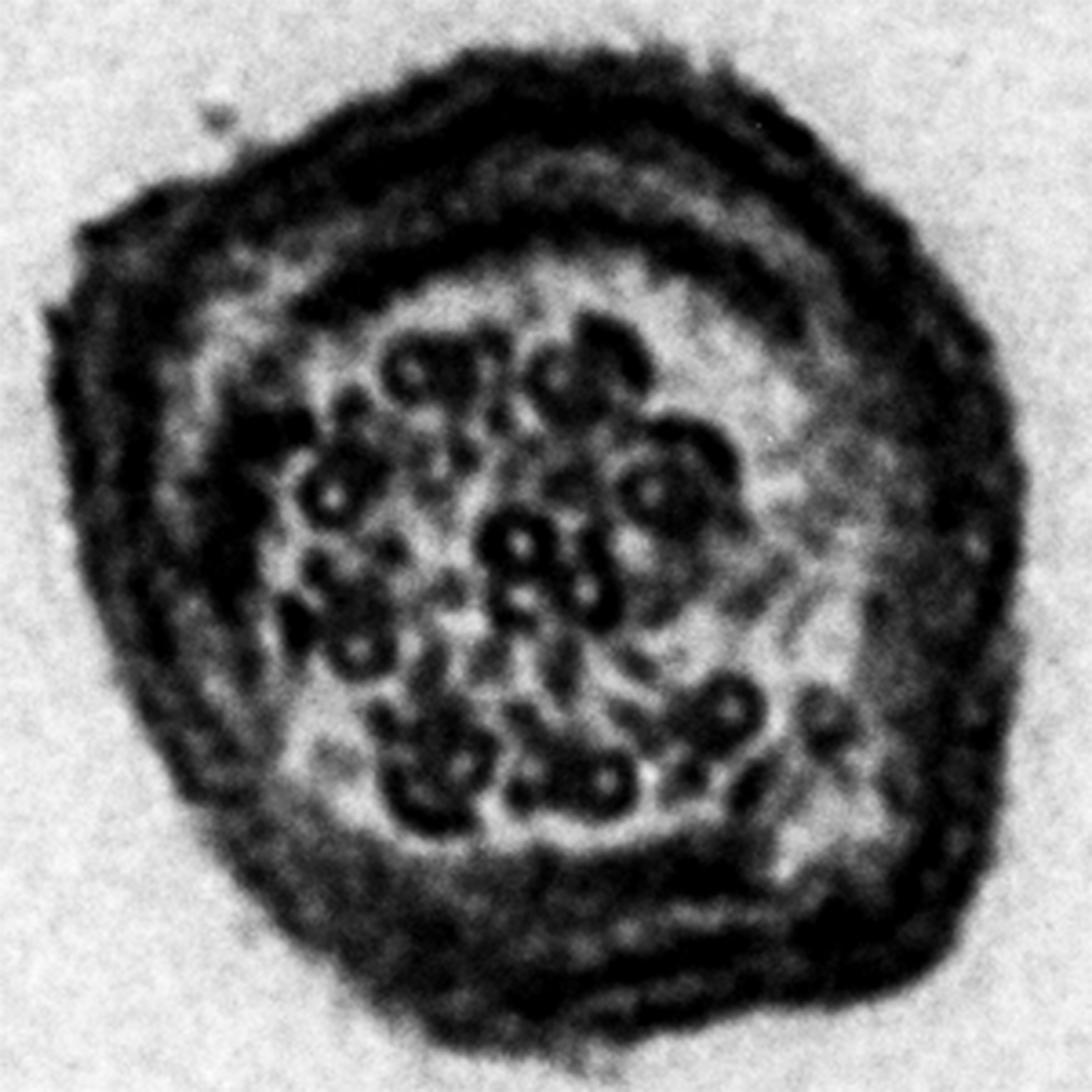

Supplement: Supplementary file 14 — Source Data Fig. 3 [file 44319_2024_112_MOESM14_ESM.zip › Figure 3/Figure 3/3H/KO PRINCIPAL PIECE.tif]

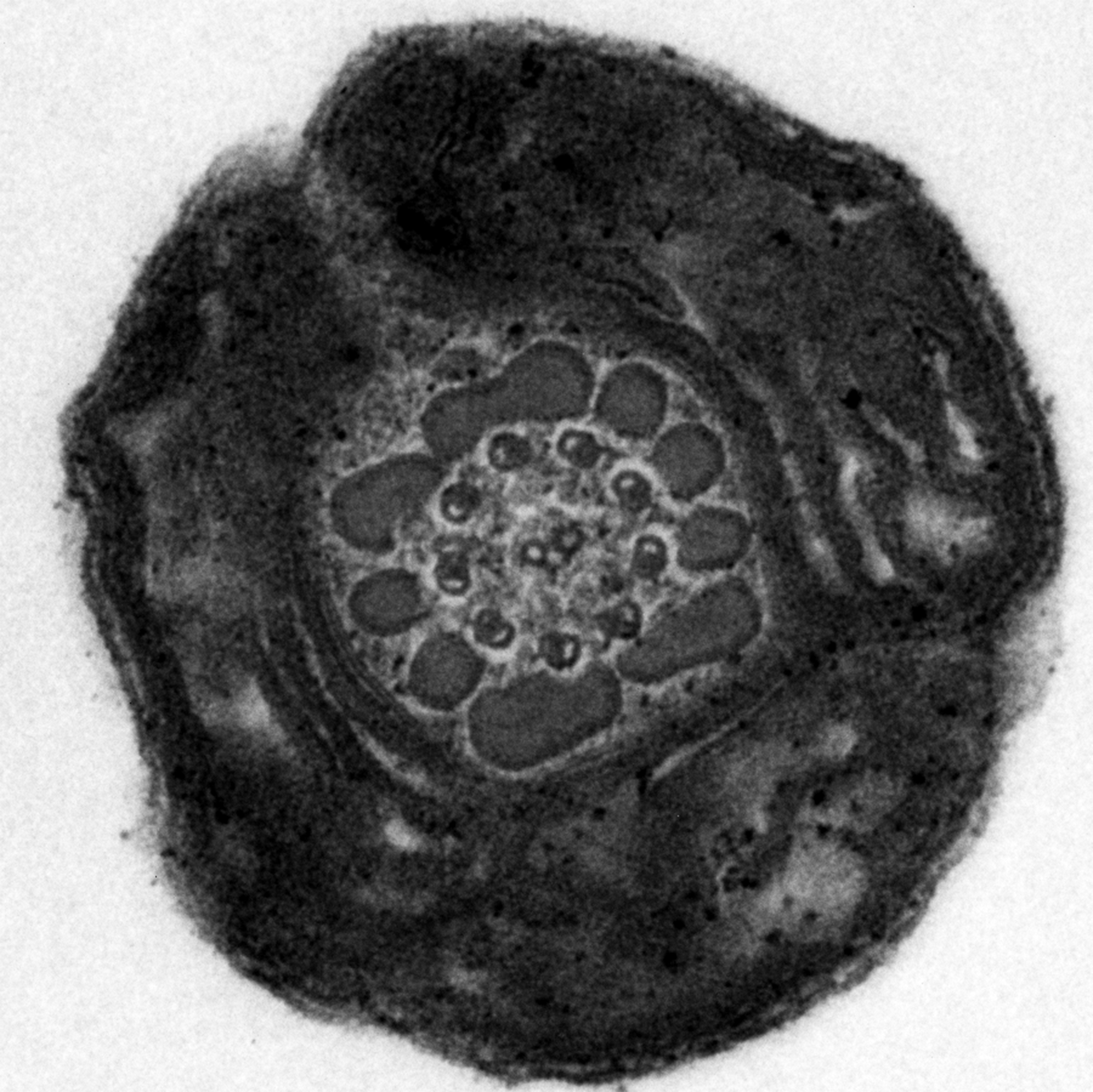

Supplement: Supplementary file 14 — Source Data Fig. 3 [file 44319_2024_112_MOESM14_ESM.zip › Figure 3/Figure 3/3H/WT MIDPIECE.tif]

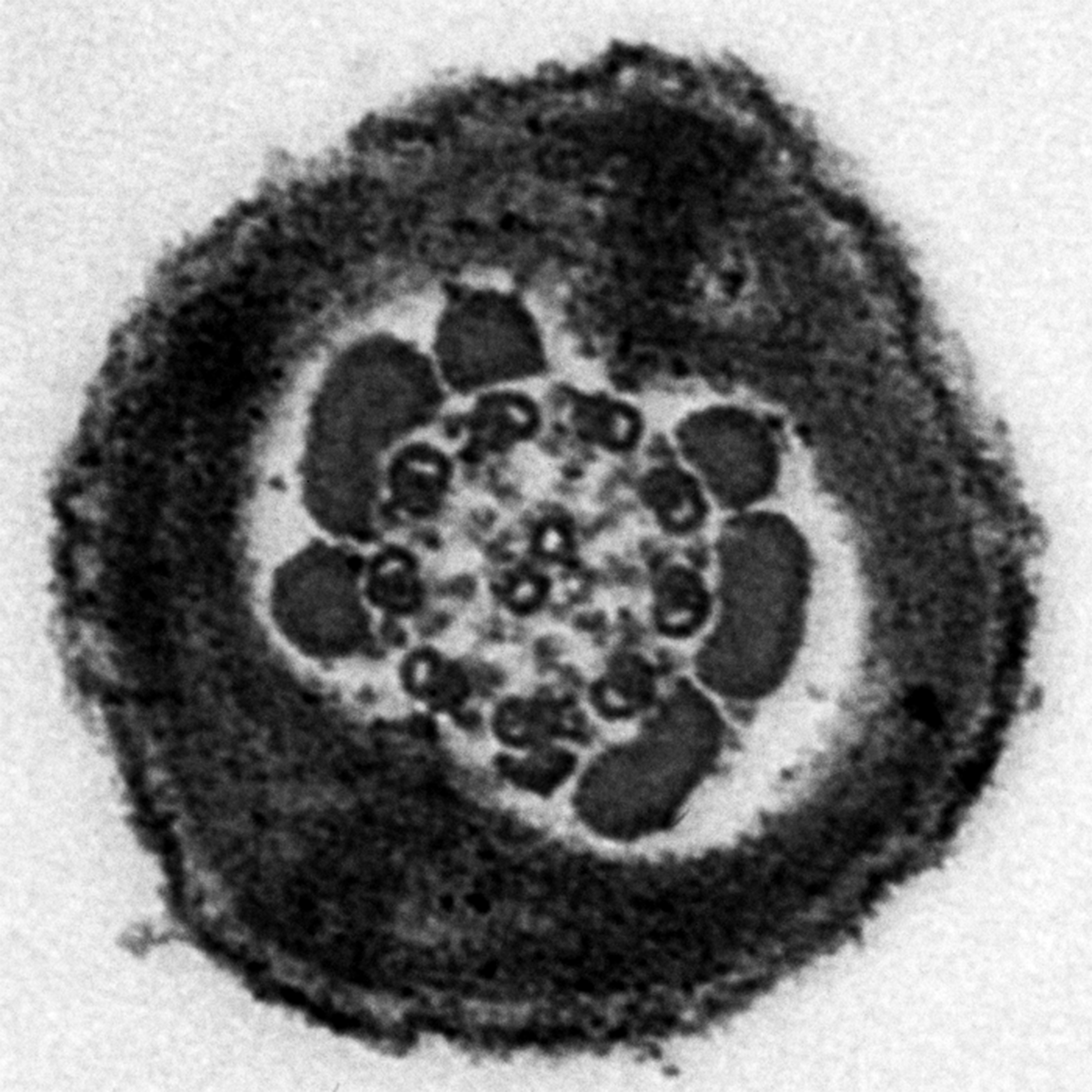

Supplement: Supplementary file 14 — Source Data Fig. 3 [file 44319_2024_112_MOESM14_ESM.zip › Figure 3/Figure 3/3H/WT PRINCIPAL PIECE.tif]

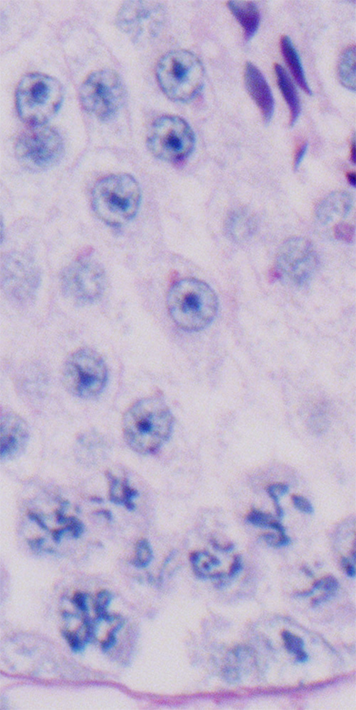

Supplement: Supplementary file 15 — Source Data Fig. 4 [file 44319_2024_112_MOESM15_ESM.zip › Figure 4/Figure 4/4A/PAS/KO/II-III.tif]

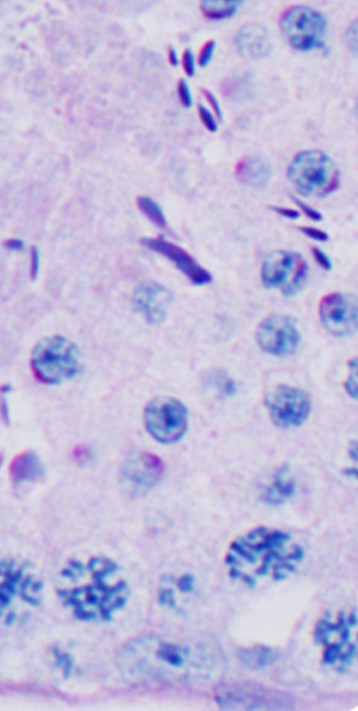

Supplement: Supplementary file 15 — Source Data Fig. 4 [file 44319_2024_112_MOESM15_ESM.zip › Figure 4/Figure 4/4A/PAS/KO/IV.tif]

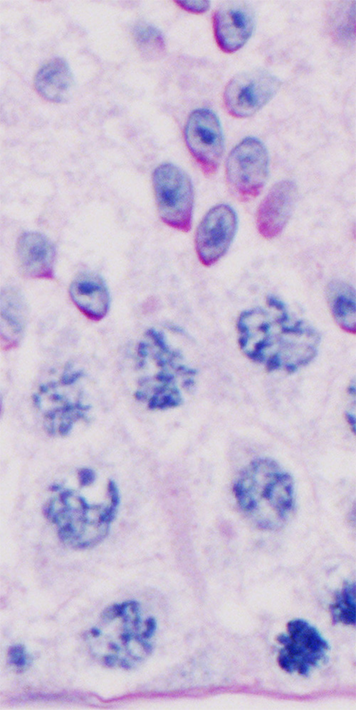

Supplement: Supplementary file 15 — Source Data Fig. 4 [file 44319_2024_112_MOESM15_ESM.zip › Figure 4/Figure 4/4A/PAS/KO/IX-XI.tif]

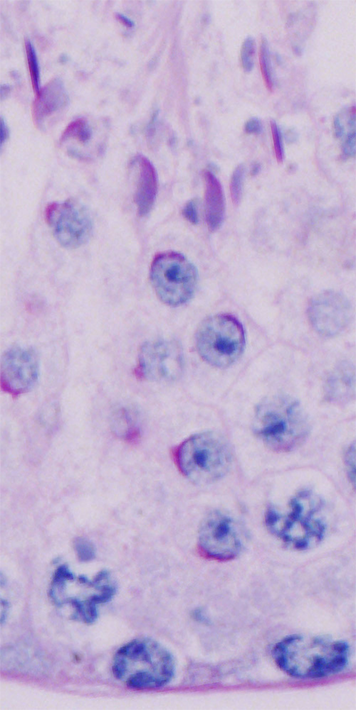

Supplement: Supplementary file 15 — Source Data Fig. 4 [file 44319_2024_112_MOESM15_ESM.zip › Figure 4/Figure 4/4A/PAS/KO/V-VII.tif]

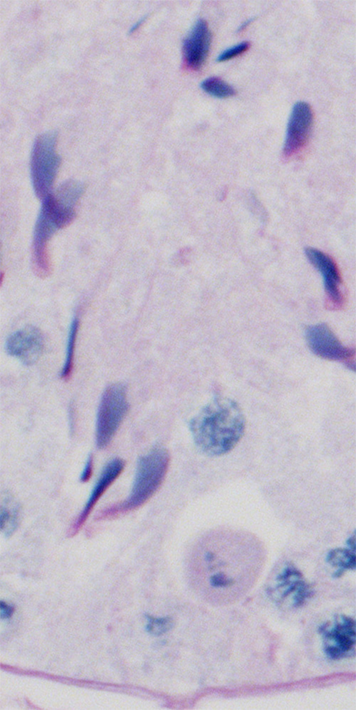

Supplement: Supplementary file 15 — Source Data Fig. 4 [file 44319_2024_112_MOESM15_ESM.zip › Figure 4/Figure 4/4A/PAS/KO/XII.tif]

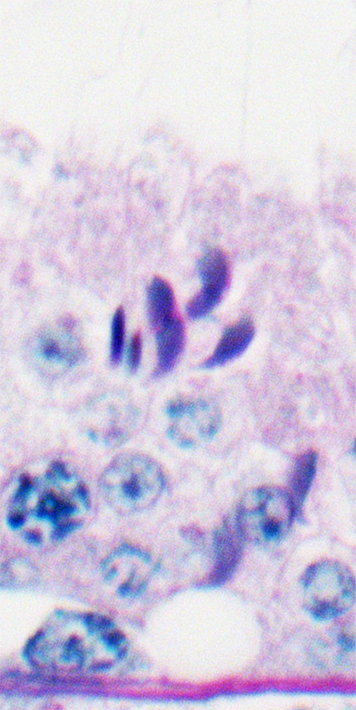

Supplement: Supplementary file 15 — Source Data Fig. 4 [file 44319_2024_112_MOESM15_ESM.zip › Figure 4/Figure 4/4A/PAS/WT/II-III.tif]

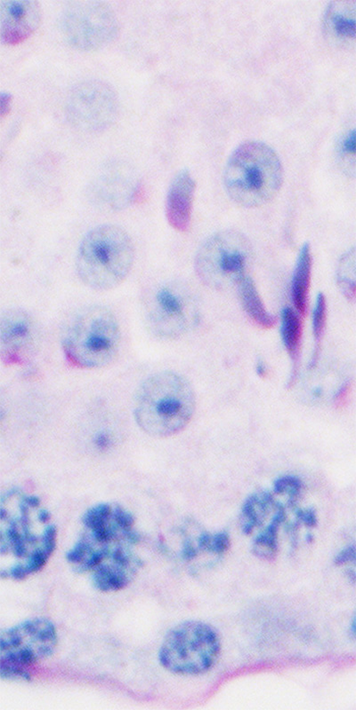

Supplement: Supplementary file 15 — Source Data Fig. 4 [file 44319_2024_112_MOESM15_ESM.zip › Figure 4/Figure 4/4A/PAS/WT/IV.tif]

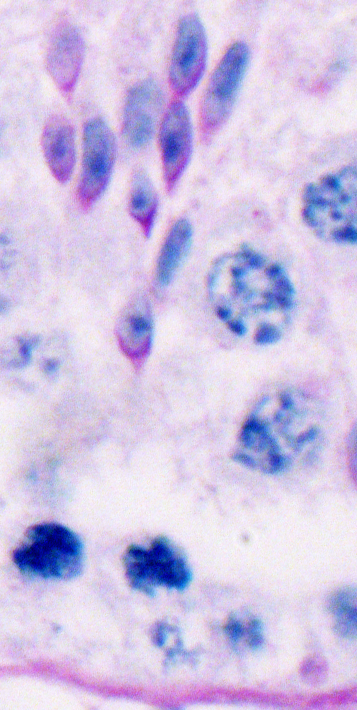

Supplement: Supplementary file 15 — Source Data Fig. 4 [file 44319_2024_112_MOESM15_ESM.zip › Figure 4/Figure 4/4A/PAS/WT/IX-XI.tif]

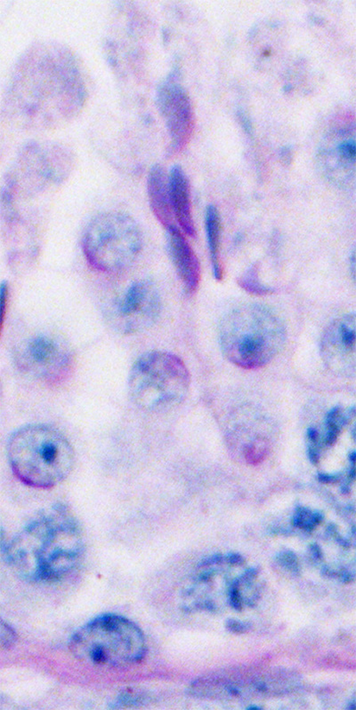

Supplement: Supplementary file 15 — Source Data Fig. 4 [file 44319_2024_112_MOESM15_ESM.zip › Figure 4/Figure 4/4A/PAS/WT/V-VII.tif]

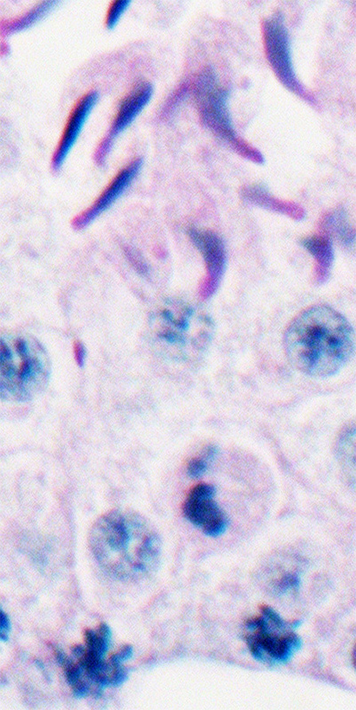

Supplement: Supplementary file 15 — Source Data Fig. 4 [file 44319_2024_112_MOESM15_ESM.zip › Figure 4/Figure 4/4A/PAS/WT/XII.tif]

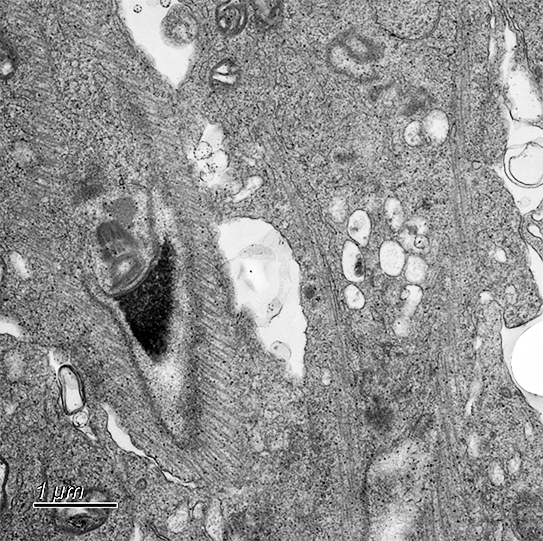

Supplement: Supplementary file 15 — Source Data Fig. 4 [file 44319_2024_112_MOESM15_ESM.zip › Figure 4/Figure 4/4C/KO/STEP 11-12.tif]

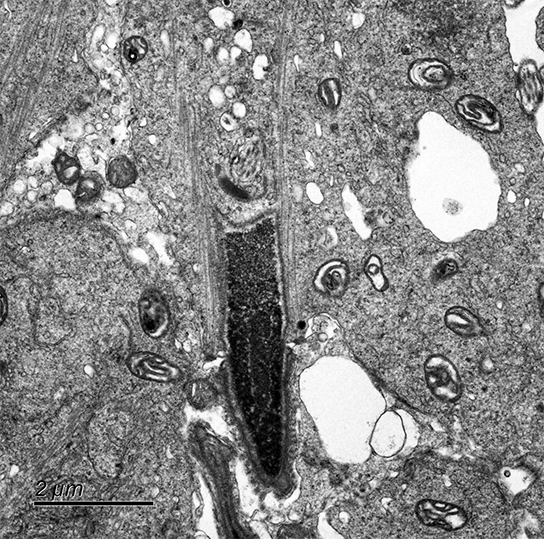

Supplement: Supplementary file 15 — Source Data Fig. 4 [file 44319_2024_112_MOESM15_ESM.zip › Figure 4/Figure 4/4C/KO/STEP 13-14.tif]

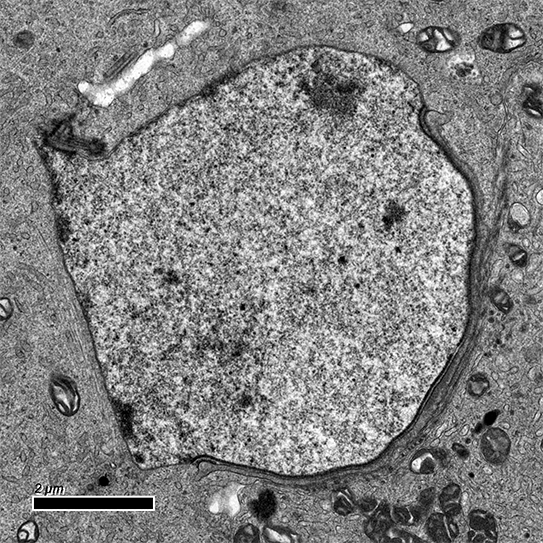

Supplement: Supplementary file 15 — Source Data Fig. 4 [file 44319_2024_112_MOESM15_ESM.zip › Figure 4/Figure 4/4C/KO/STEP 6-8.tif]

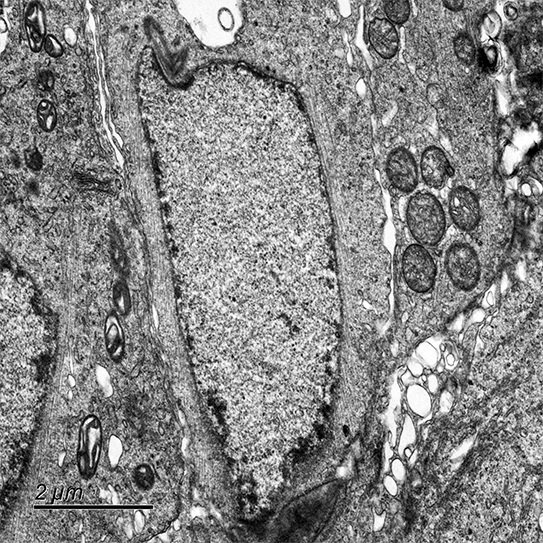

Supplement: Supplementary file 15 — Source Data Fig. 4 [file 44319_2024_112_MOESM15_ESM.zip › Figure 4/Figure 4/4C/KO/STEP 9-10.tif]

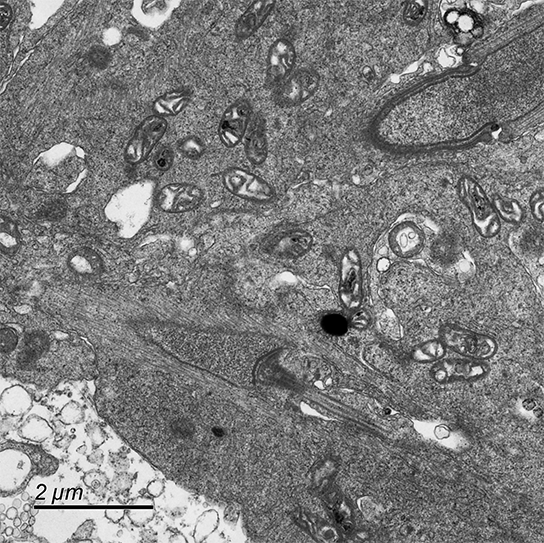

Supplement: Supplementary file 15 — Source Data Fig. 4 [file 44319_2024_112_MOESM15_ESM.zip › Figure 4/Figure 4/4C/WT/STEP 11-12.tif]

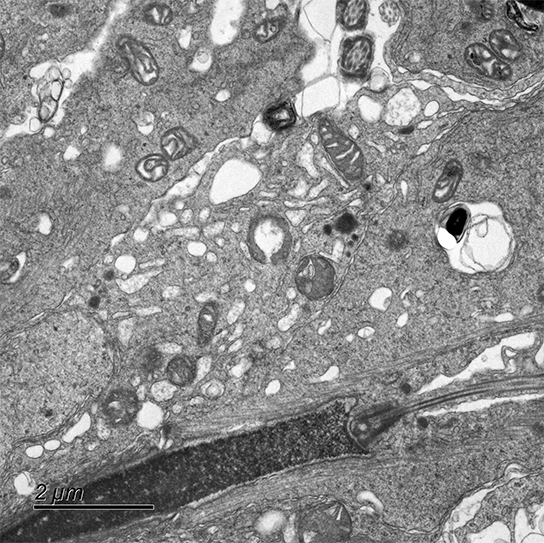

Supplement: Supplementary file 15 — Source Data Fig. 4 [file 44319_2024_112_MOESM15_ESM.zip › Figure 4/Figure 4/4C/WT/STEP 13-14.tif]

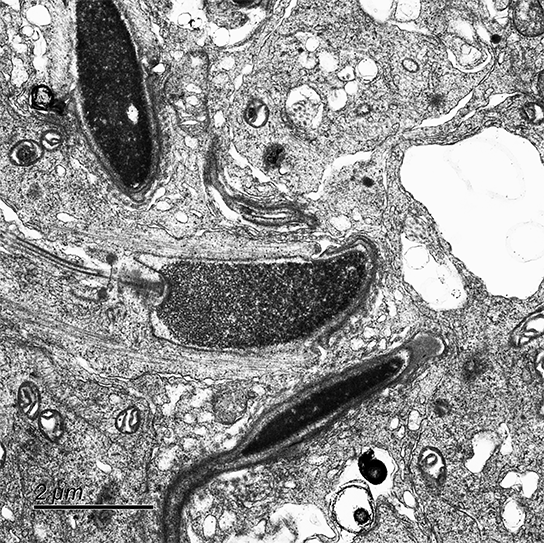

Supplement: Supplementary file 15 — Source Data Fig. 4 [file 44319_2024_112_MOESM15_ESM.zip › Figure 4/Figure 4/4C/WT/STEP 15-16.tif]

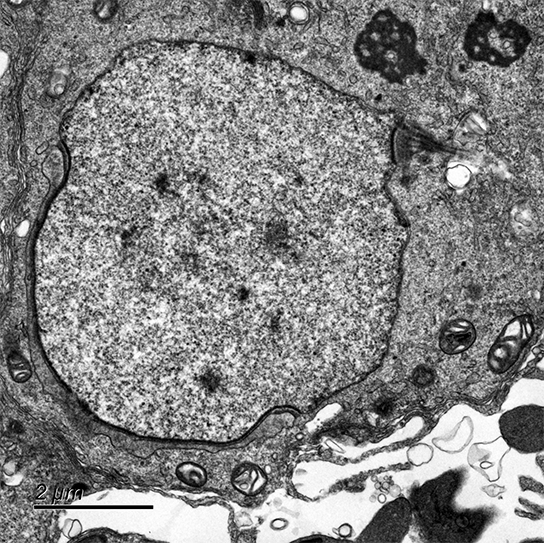

Supplement: Supplementary file 15 — Source Data Fig. 4 [file 44319_2024_112_MOESM15_ESM.zip › Figure 4/Figure 4/4C/WT/STEP 6-8.tif]

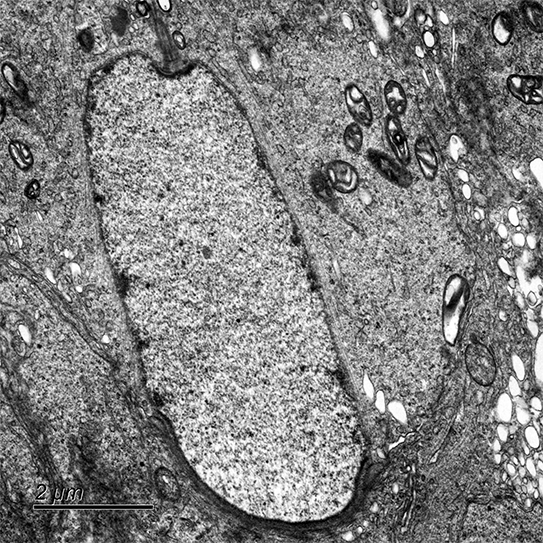

Supplement: Supplementary file 15 — Source Data Fig. 4 [file 44319_2024_112_MOESM15_ESM.zip › Figure 4/Figure 4/4C/WT/STEP 9-10.tif]

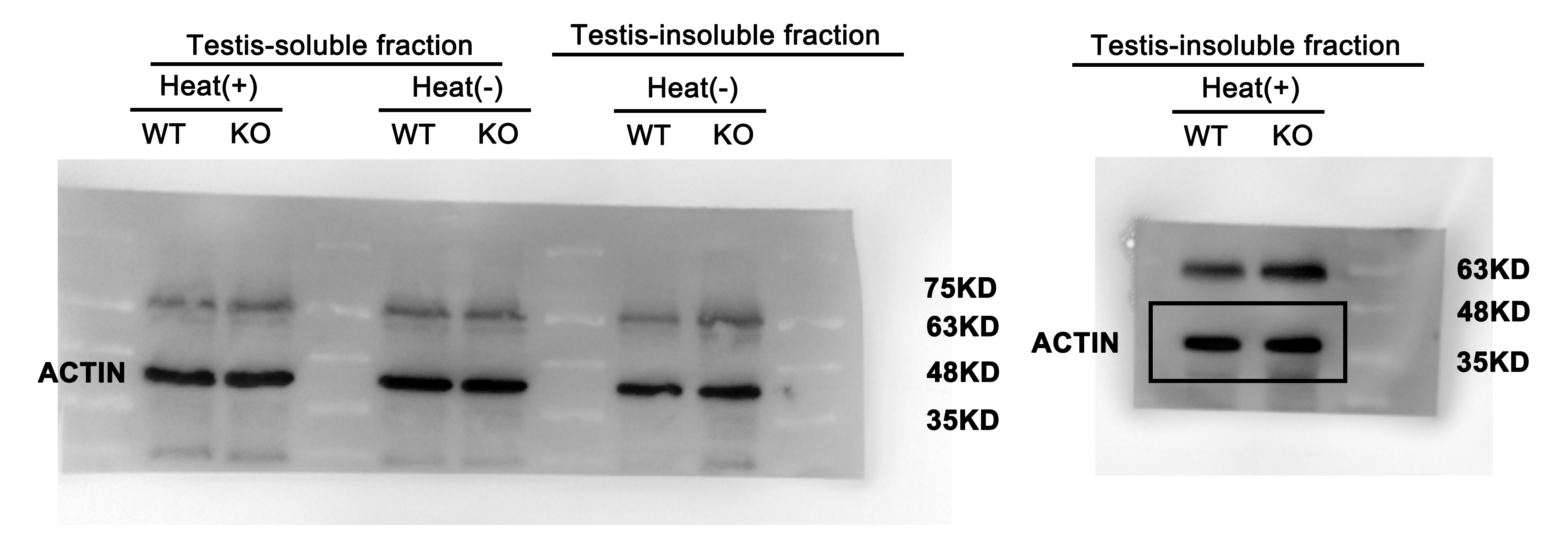

Supplement: Supplementary file 16 — Source Data Fig. 5 [file 44319_2024_112_MOESM16_ESM.zip › Figure 5/Figure 5/5D/WB ACTIN.tif]

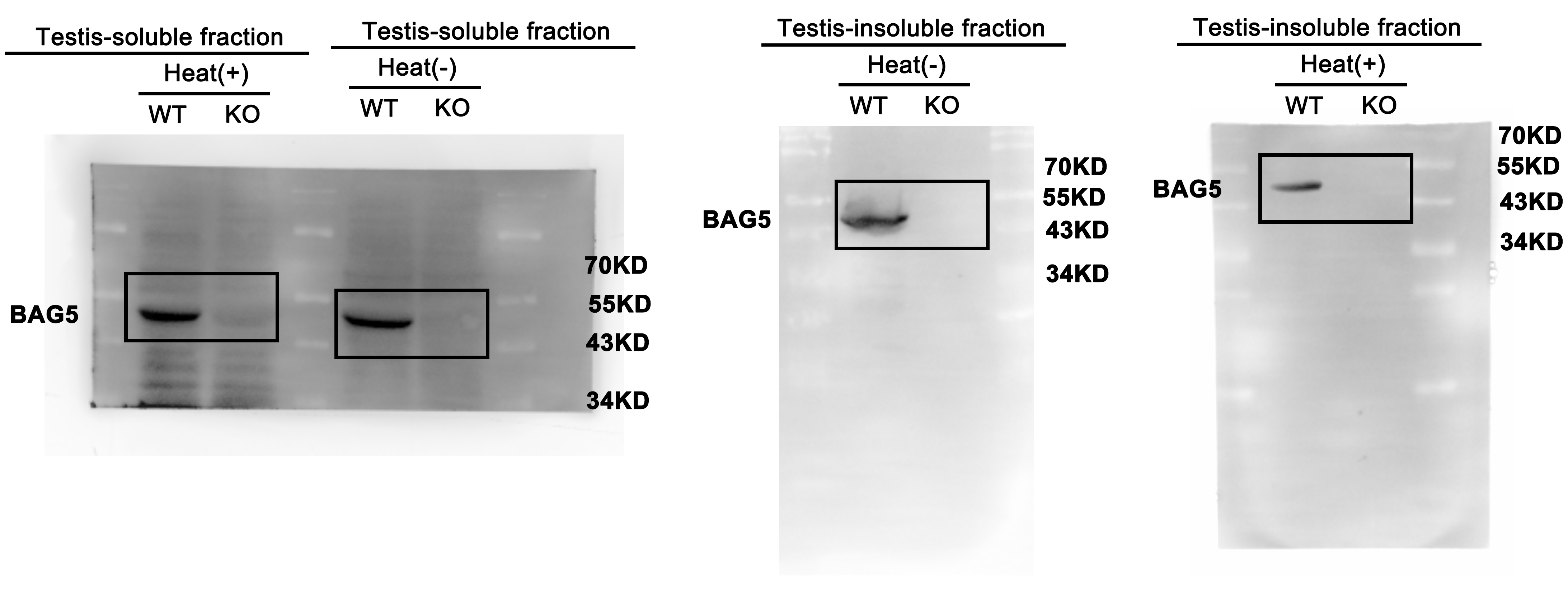

Supplement: Supplementary file 16 — Source Data Fig. 5 [file 44319_2024_112_MOESM16_ESM.zip › Figure 5/Figure 5/5D/WB BAG5.tif]

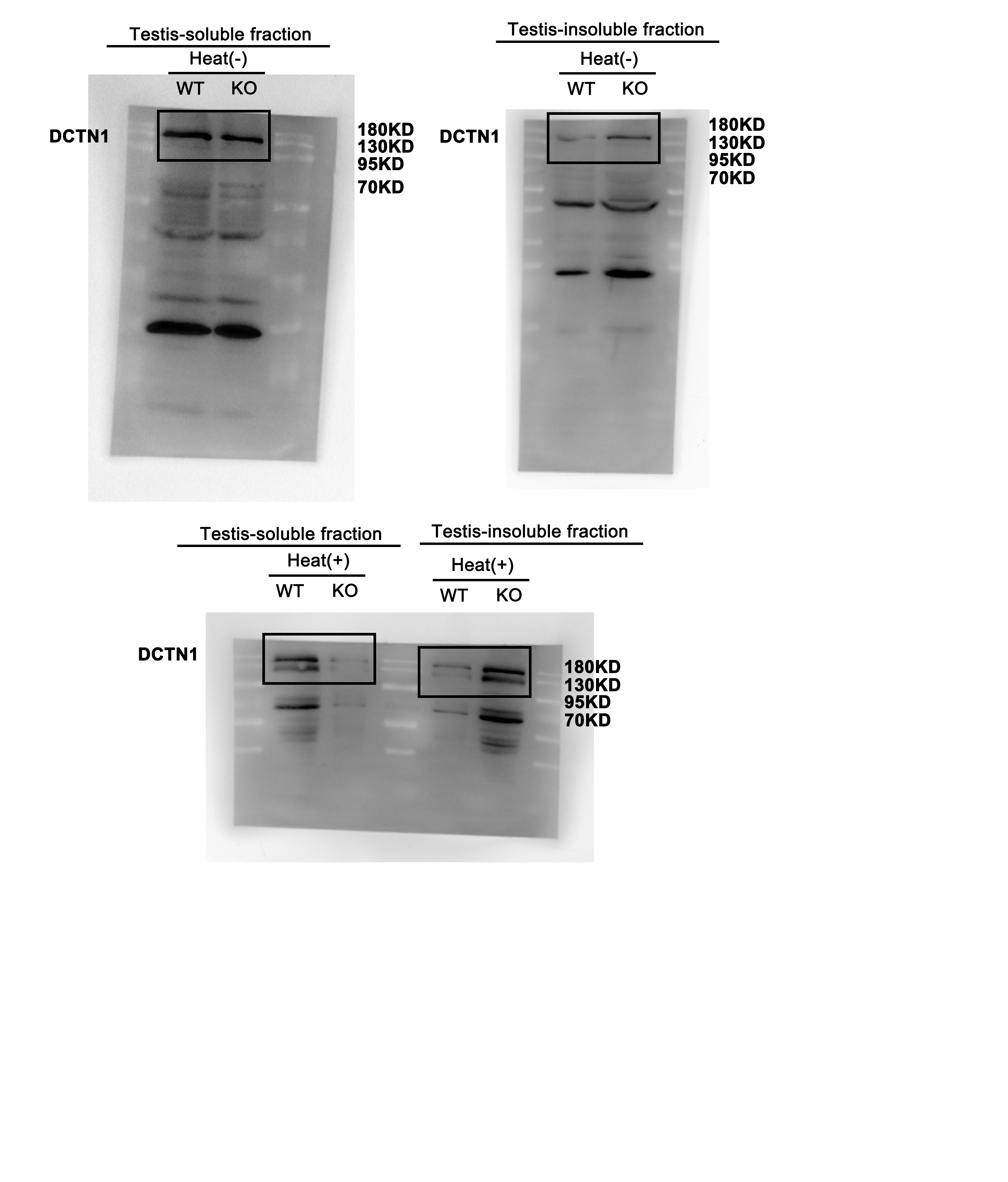

Supplement: Supplementary file 16 — Source Data Fig. 5 [file 44319_2024_112_MOESM16_ESM.zip › Figure 5/Figure 5/5D/WB DCTN1.tif]

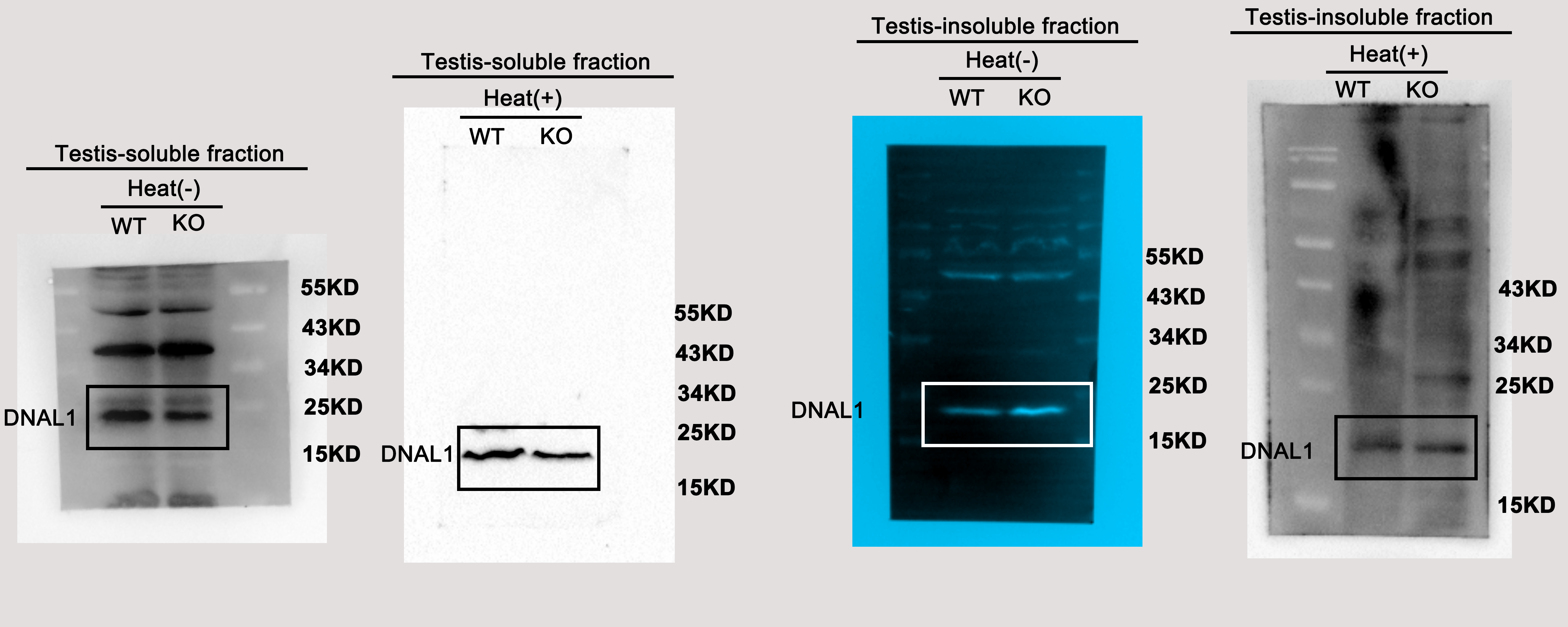

Supplement: Supplementary file 16 — Source Data Fig. 5 [file 44319_2024_112_MOESM16_ESM.zip › Figure 5/Figure 5/5D/WB DNAL1.tif]

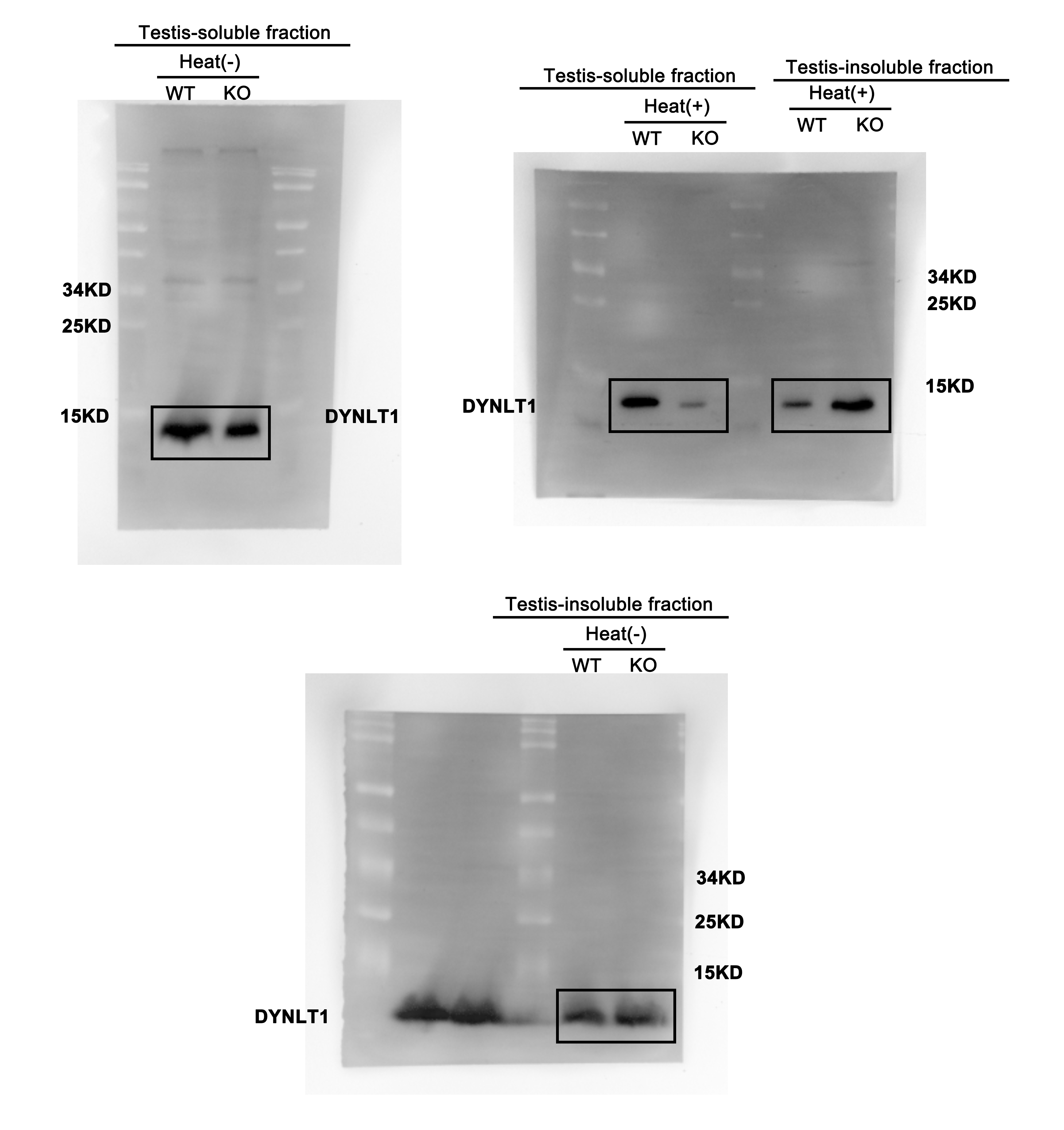

Supplement: Supplementary file 16 — Source Data Fig. 5 [file 44319_2024_112_MOESM16_ESM.zip › Figure 5/Figure 5/5D/WB DYNLT1.tif]

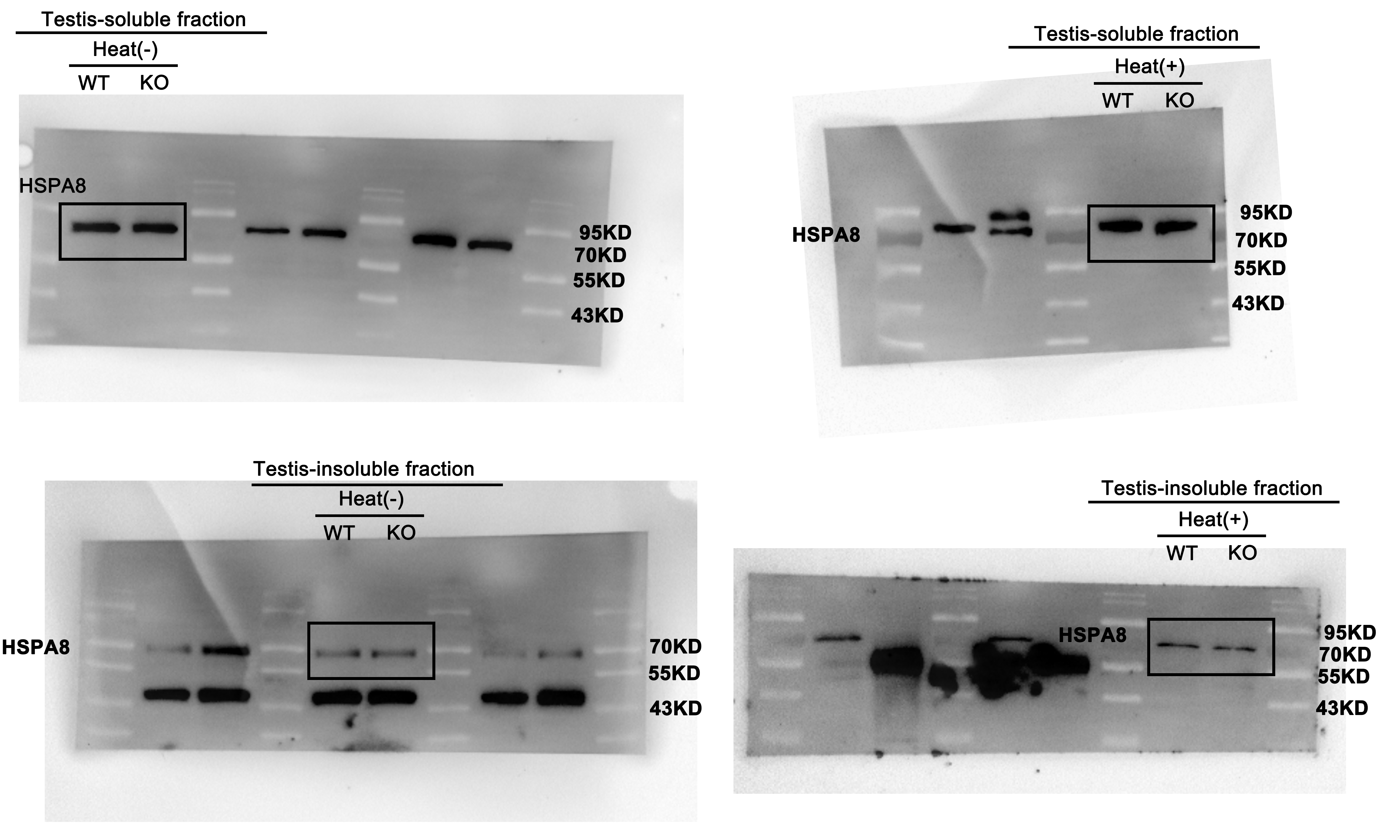

Supplement: Supplementary file 16 — Source Data Fig. 5 [file 44319_2024_112_MOESM16_ESM.zip › Figure 5/Figure 5/5D/WB HSPA8.tif]

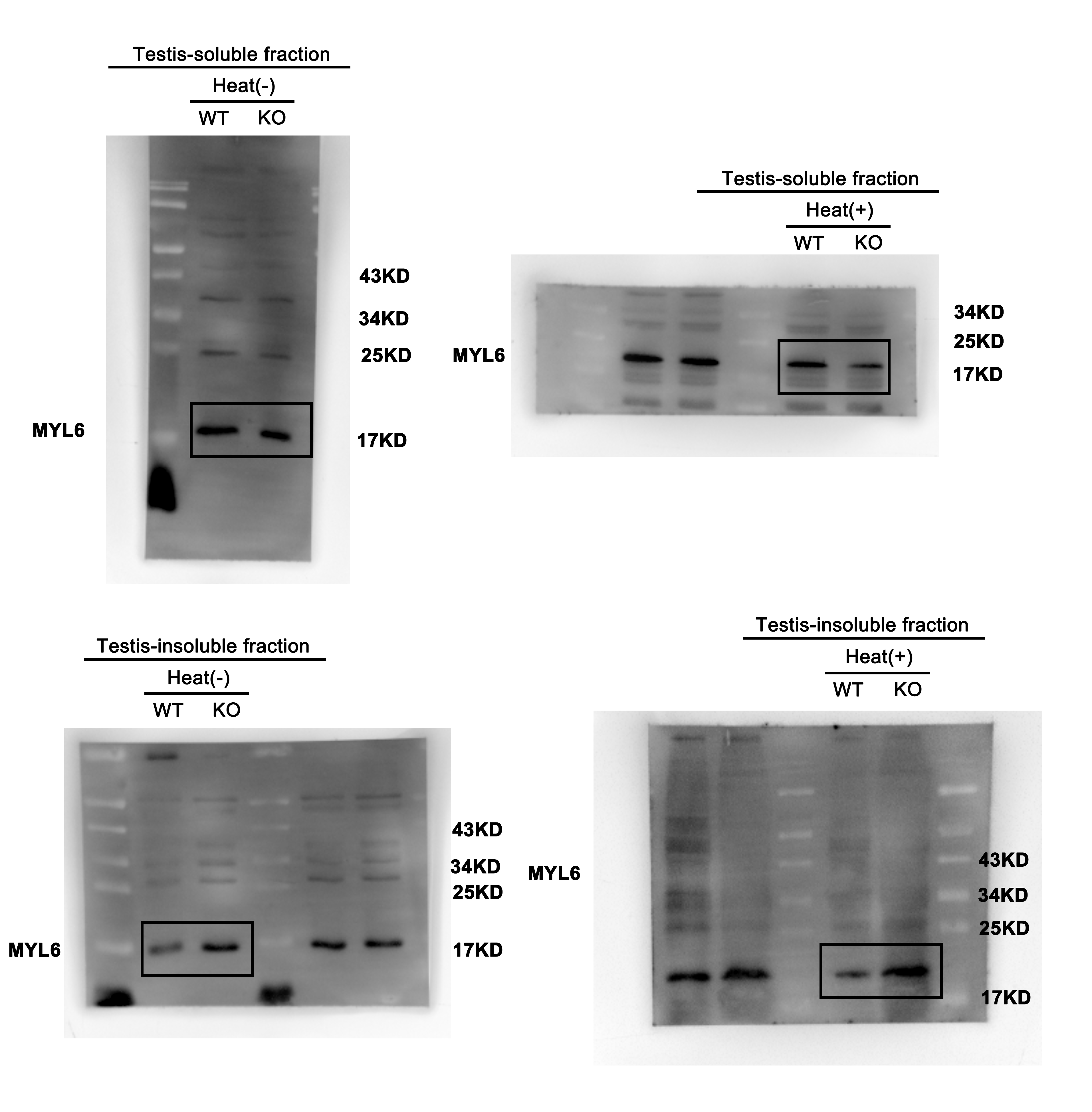

Supplement: Supplementary file 16 — Source Data Fig. 5 [file 44319_2024_112_MOESM16_ESM.zip › Figure 5/Figure 5/5D/WB MYL6.tif]

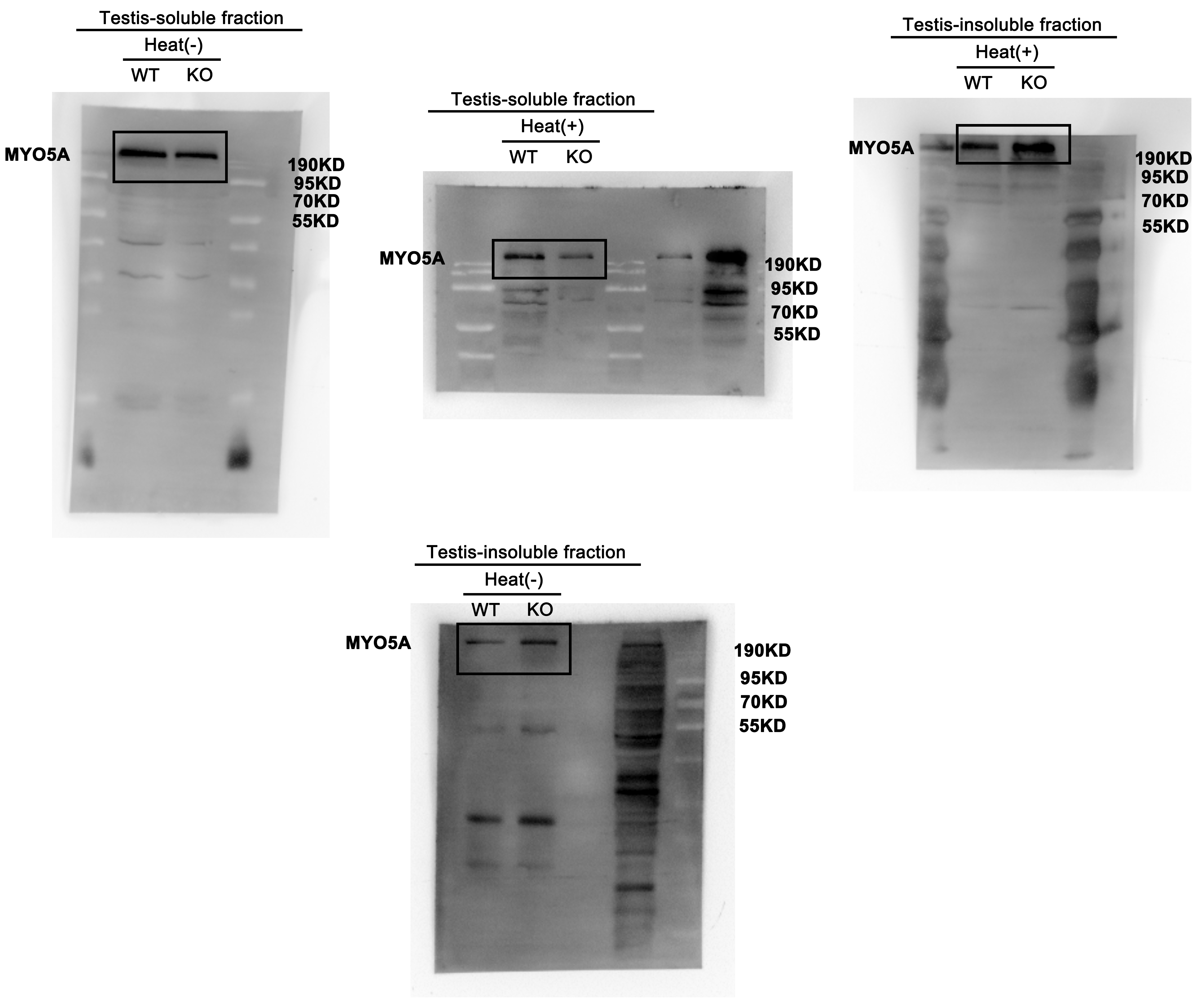

Supplement: Supplementary file 16 — Source Data Fig. 5 [file 44319_2024_112_MOESM16_ESM.zip › Figure 5/Figure 5/5D/WB MYO5A.tif]

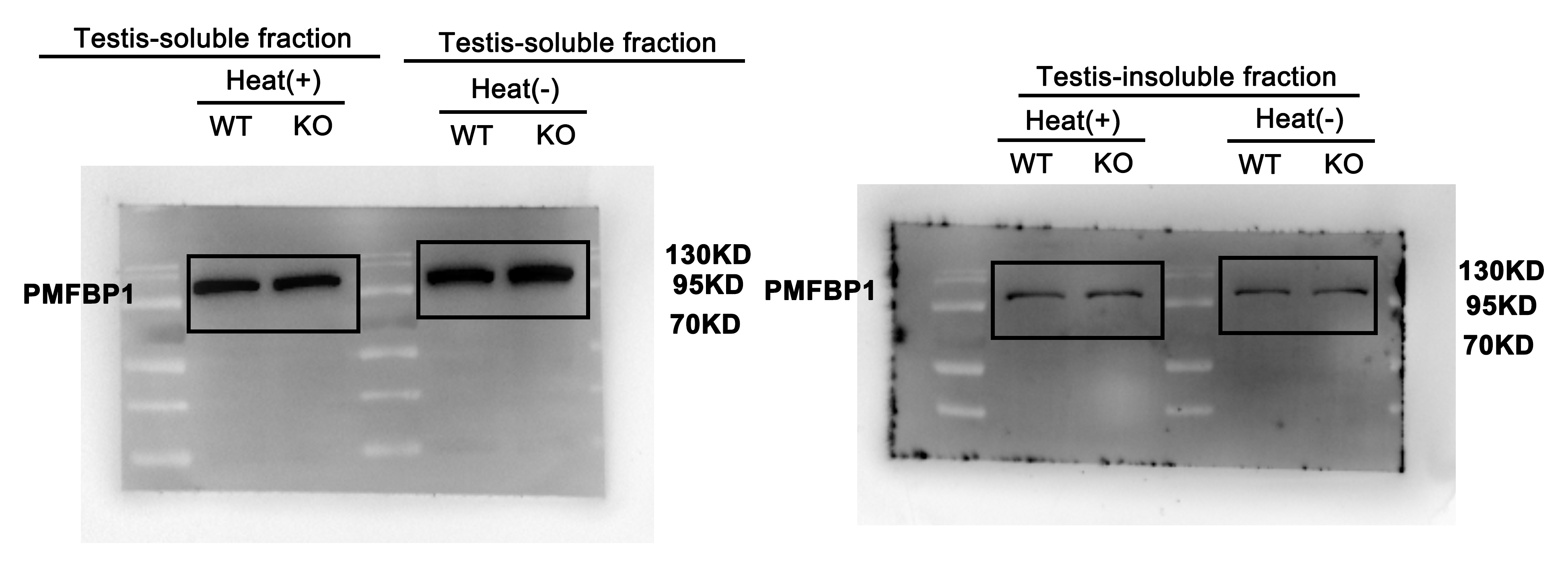

Supplement: Supplementary file 16 — Source Data Fig. 5 [file 44319_2024_112_MOESM16_ESM.zip › Figure 5/Figure 5/5D/WB PMFBP1.tif]

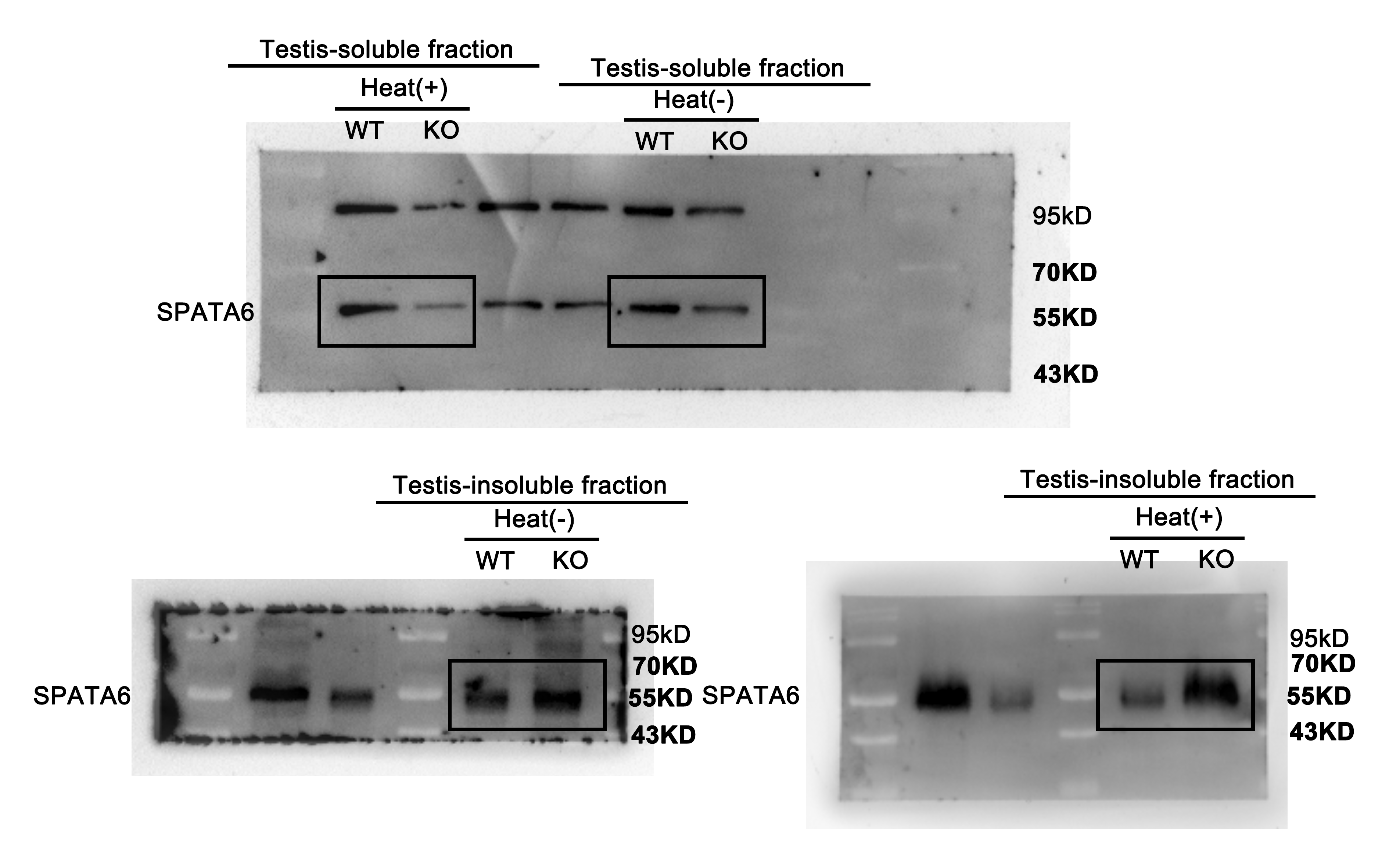

Supplement: Supplementary file 16 — Source Data Fig. 5 [file 44319_2024_112_MOESM16_ESM.zip › Figure 5/Figure 5/5D/WB SPATA6.tif]

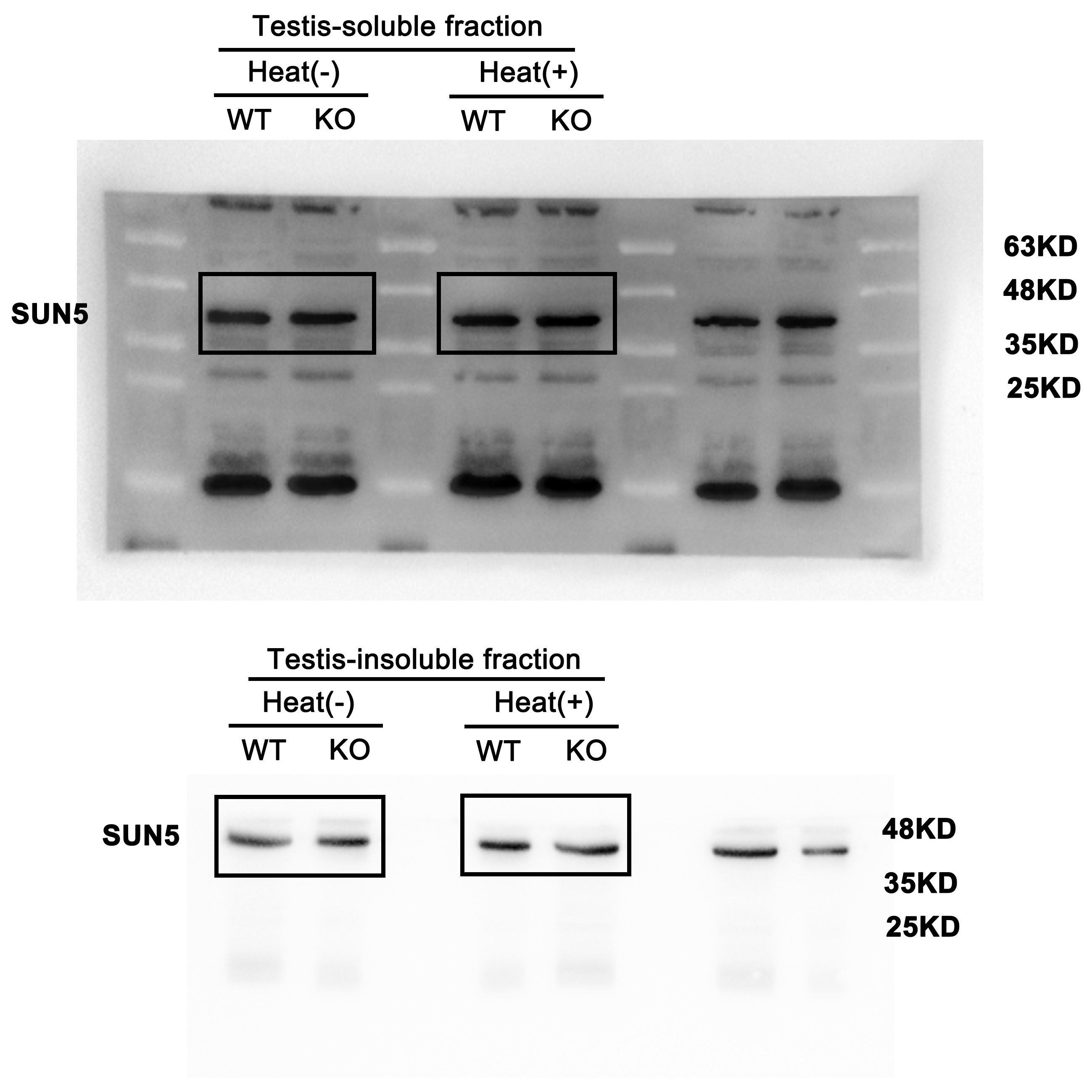

Supplement: Supplementary file 16 — Source Data Fig. 5 [file 44319_2024_112_MOESM16_ESM.zip › Figure 5/Figure 5/5D/WB SUN5.tif]

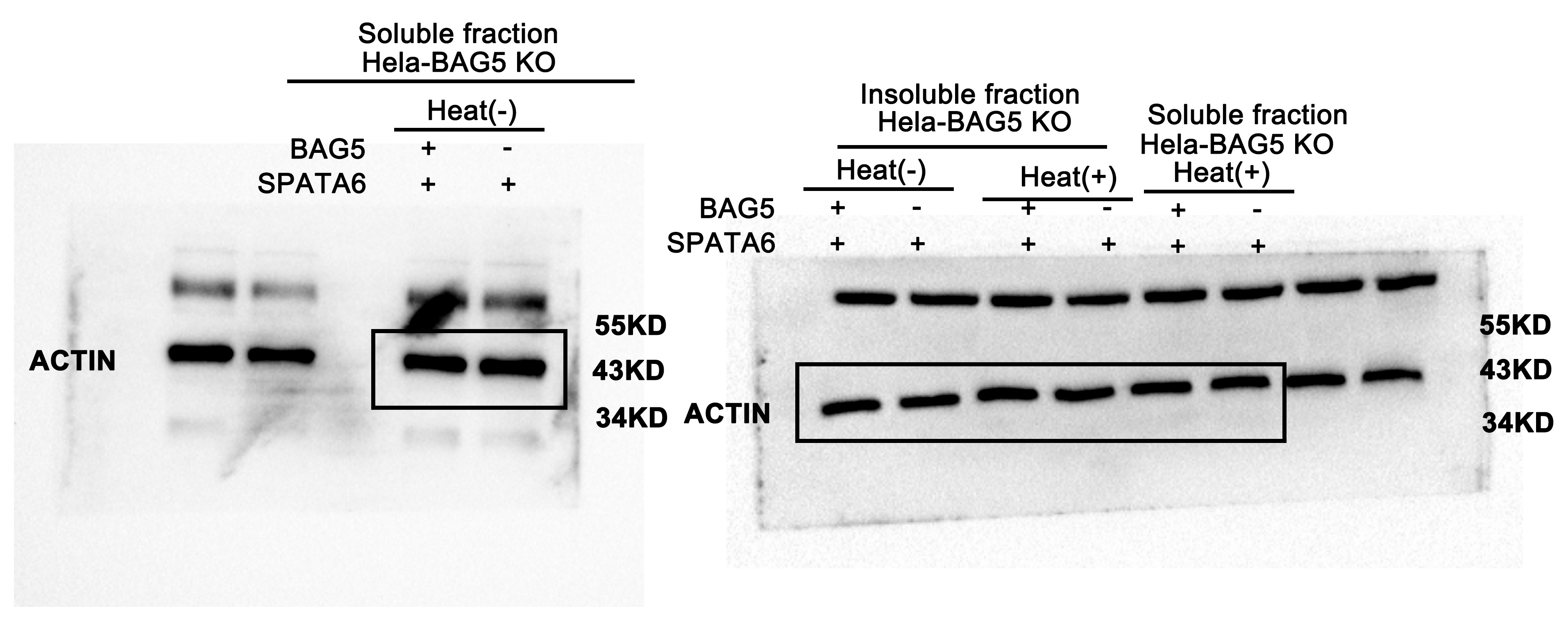

Supplement: Supplementary file 16 — Source Data Fig. 5 [file 44319_2024_112_MOESM16_ESM.zip › Figure 5/Figure 5/5E/WB ACTIN.tif]

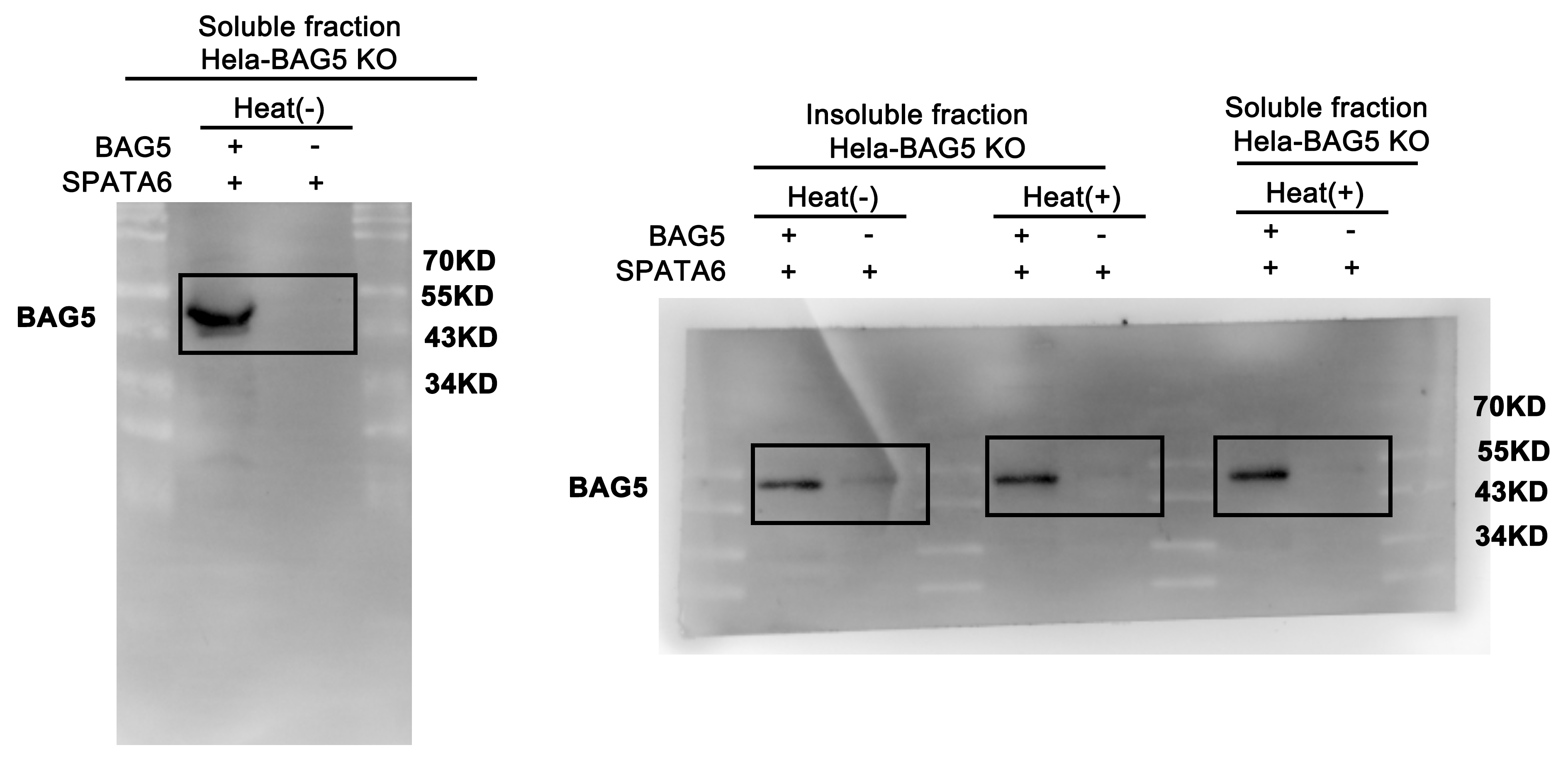

Supplement: Supplementary file 16 — Source Data Fig. 5 [file 44319_2024_112_MOESM16_ESM.zip › Figure 5/Figure 5/5E/WB BAG5.tif]

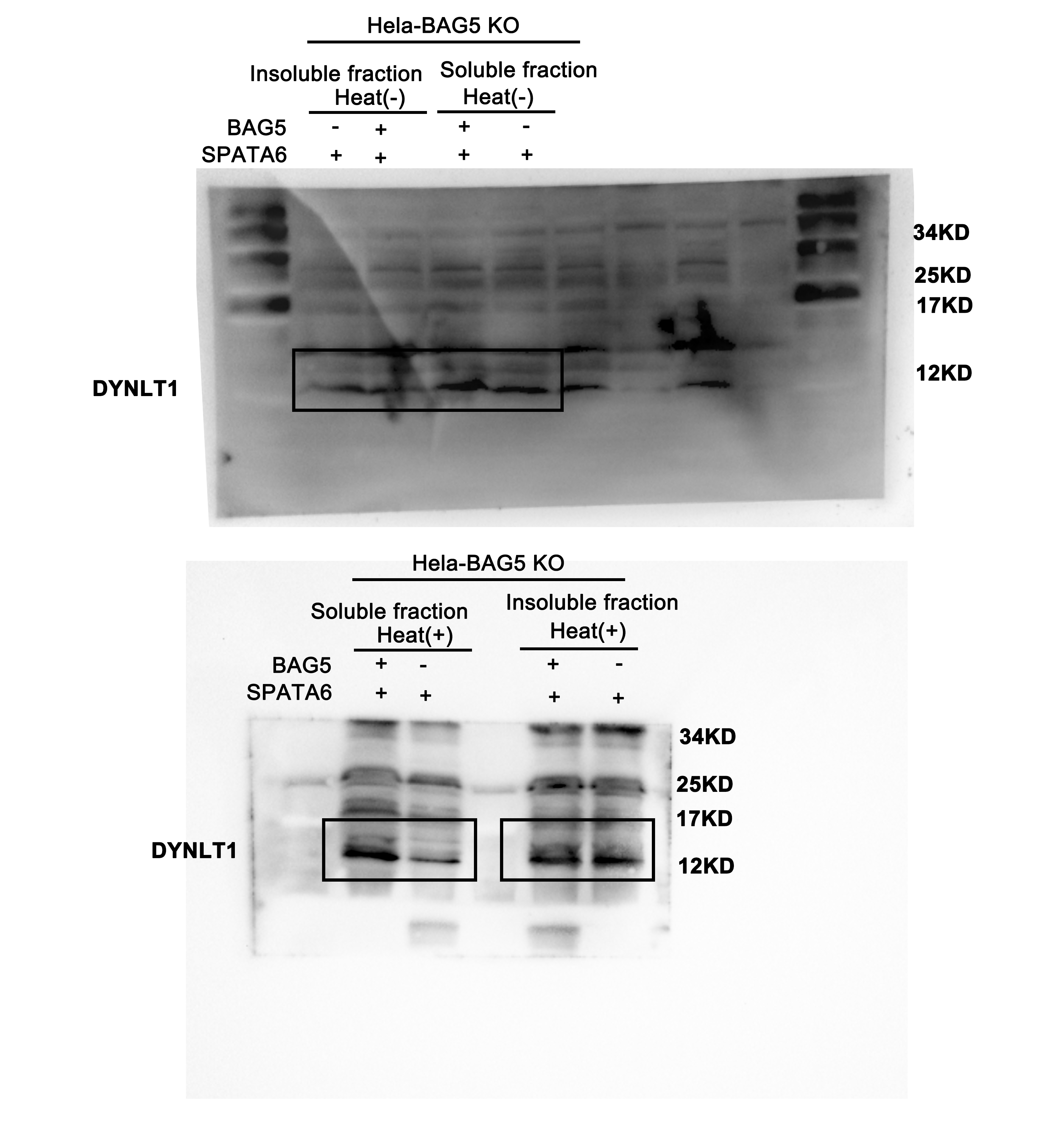

Supplement: Supplementary file 16 — Source Data Fig. 5 [file 44319_2024_112_MOESM16_ESM.zip › Figure 5/Figure 5/5E/WB DYNLT1.tif]

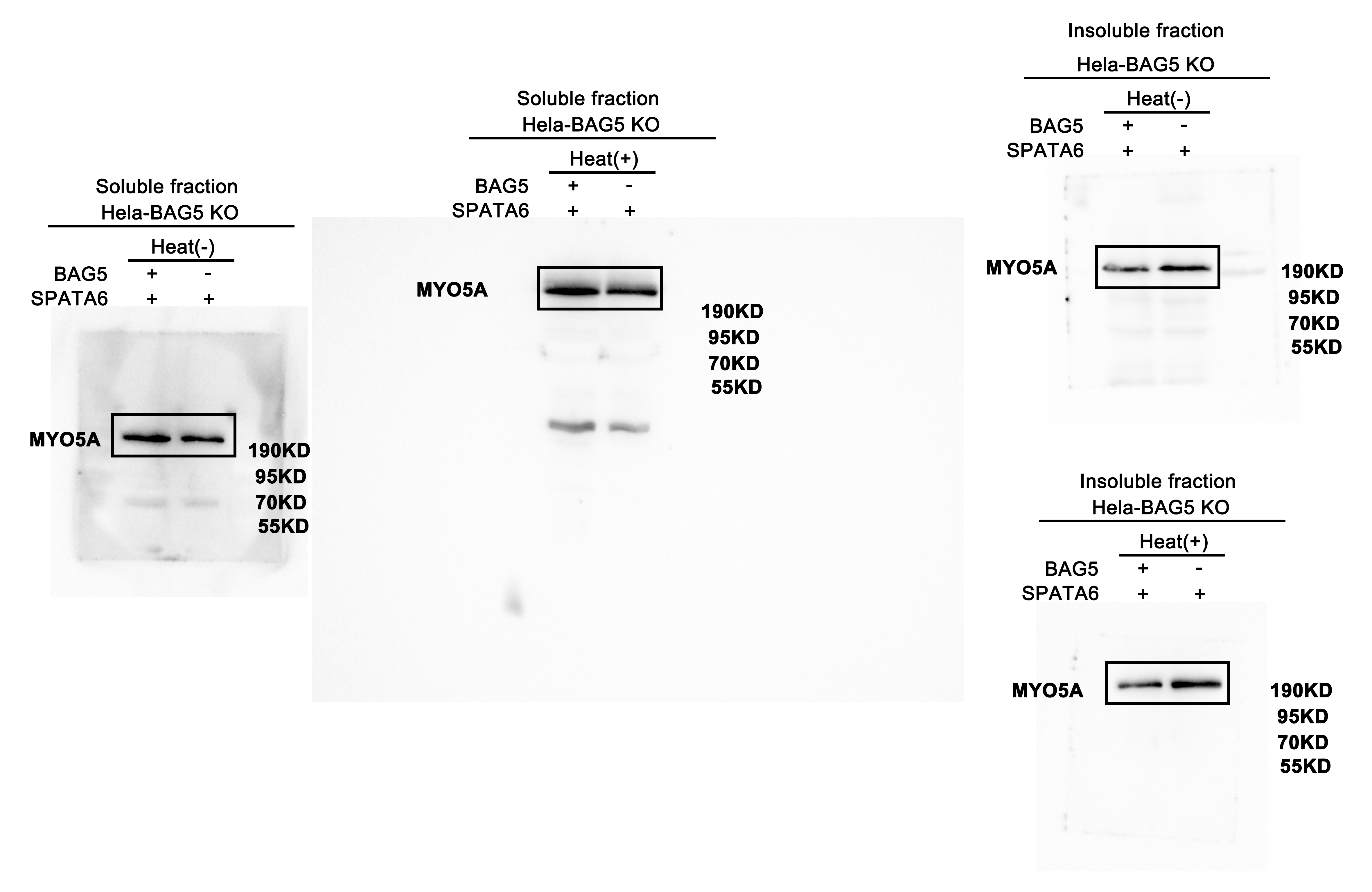

Supplement: Supplementary file 16 — Source Data Fig. 5 [file 44319_2024_112_MOESM16_ESM.zip › Figure 5/Figure 5/5E/WB MYO5A.tif]

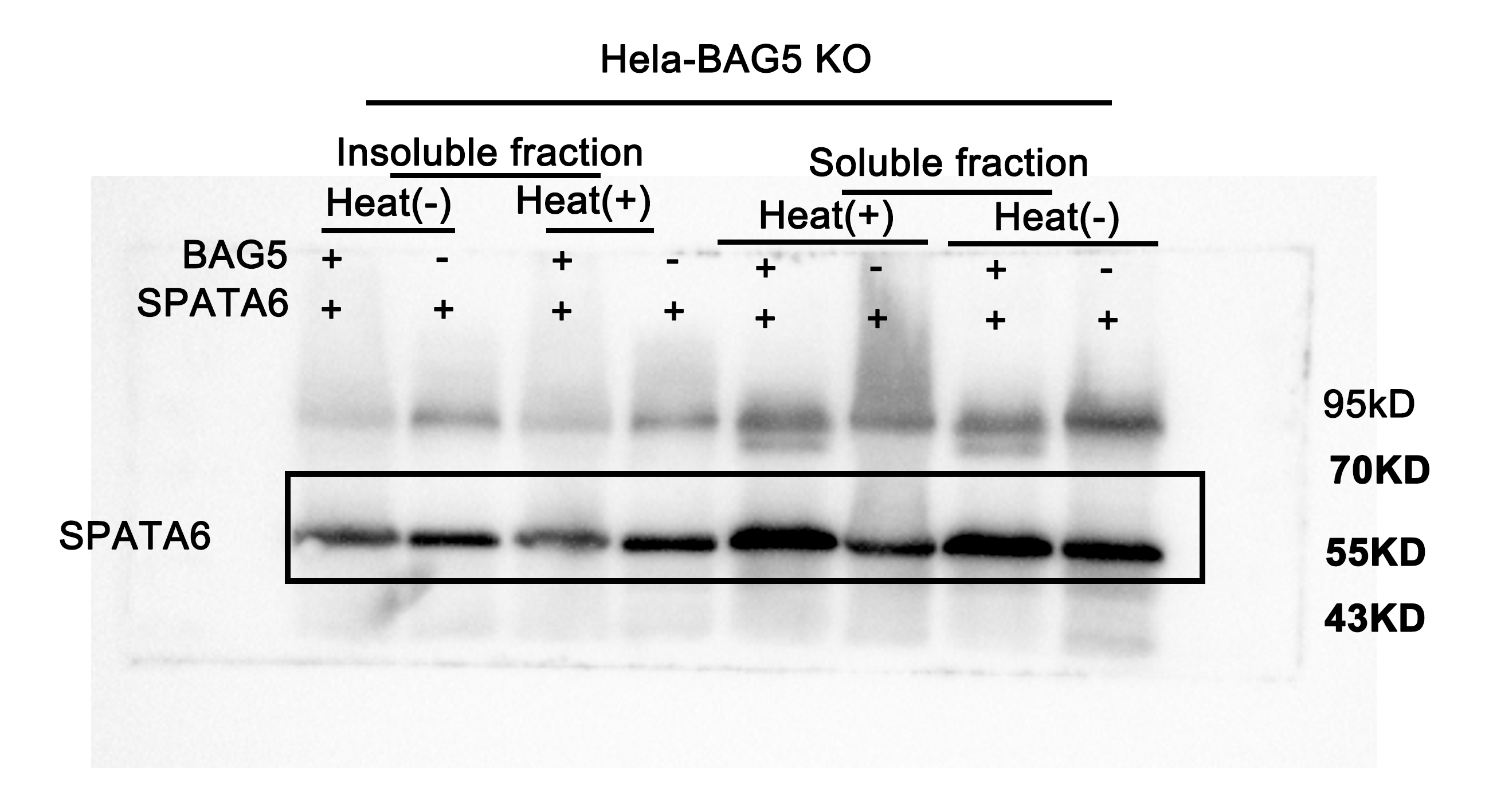

Supplement: Supplementary file 16 — Source Data Fig. 5 [file 44319_2024_112_MOESM16_ESM.zip › Figure 5/Figure 5/5E/WB SPATA6.tif]

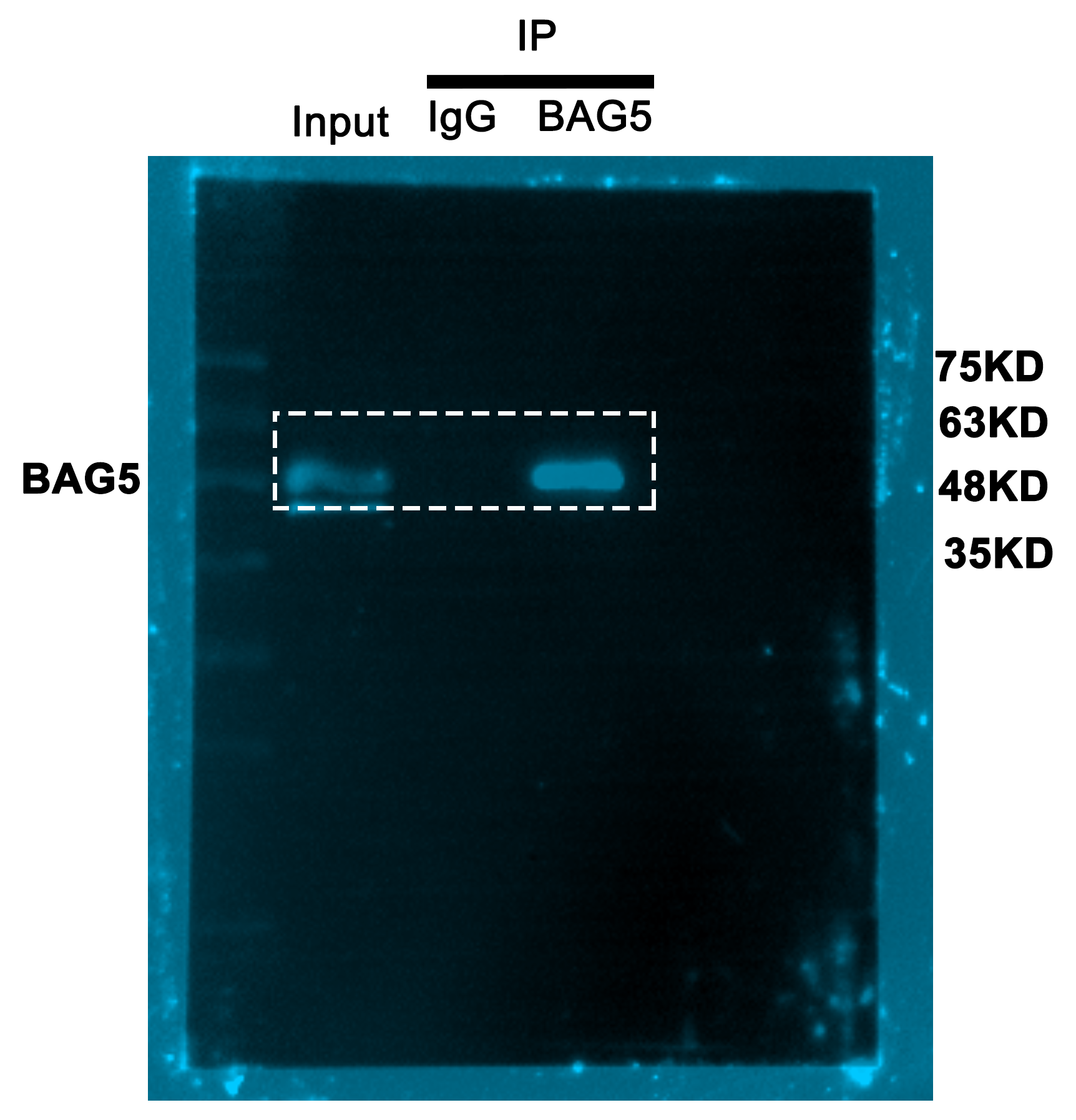

Supplement: Supplementary file 17 — Source Data Fig. 6 [file 44319_2024_112_MOESM17_ESM.zip › Figure 6/Figure 6/6E/WB BAG5.tif]

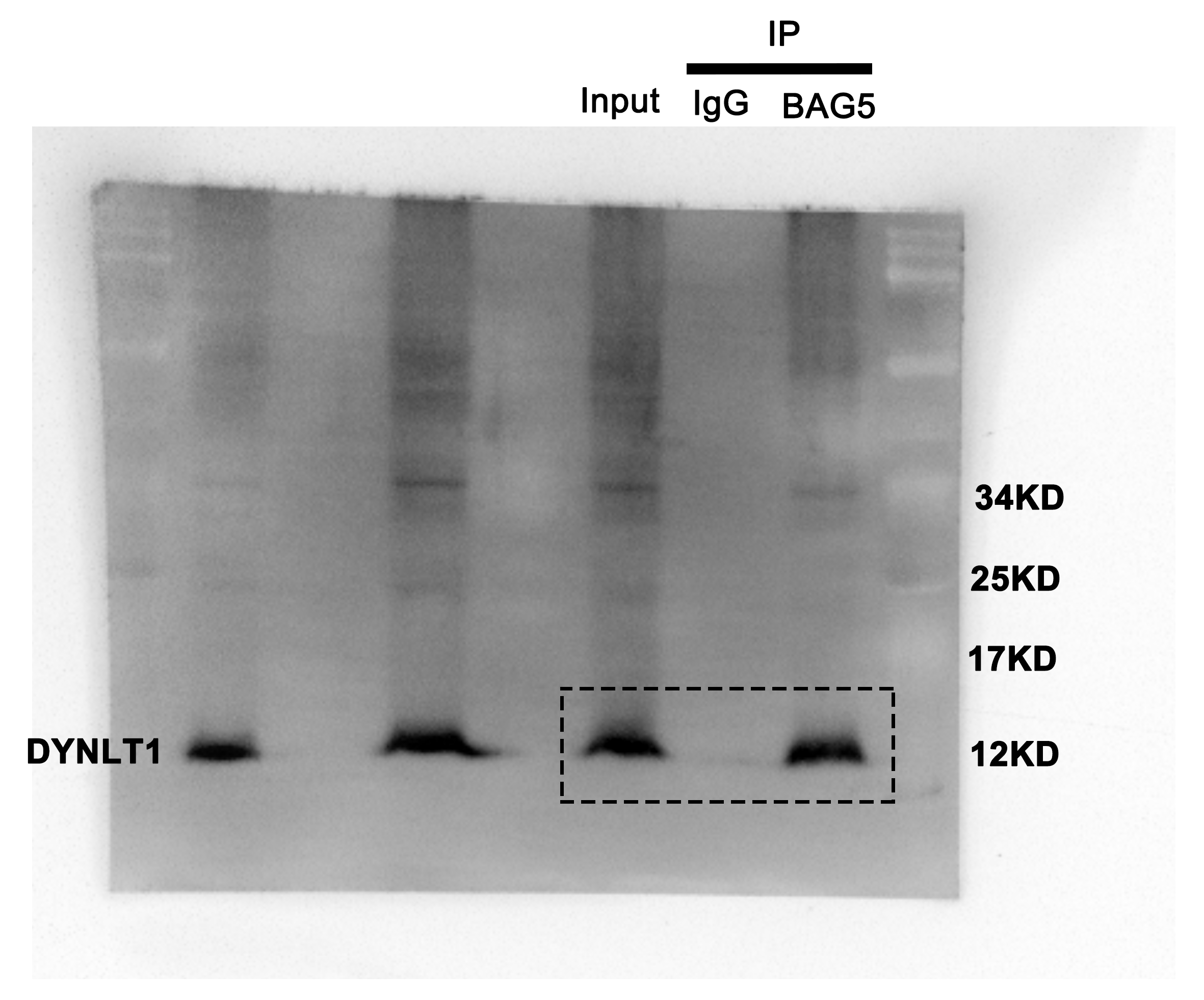

Supplement: Supplementary file 17 — Source Data Fig. 6 [file 44319_2024_112_MOESM17_ESM.zip › Figure 6/Figure 6/6E/WB DYNLT1.tif]

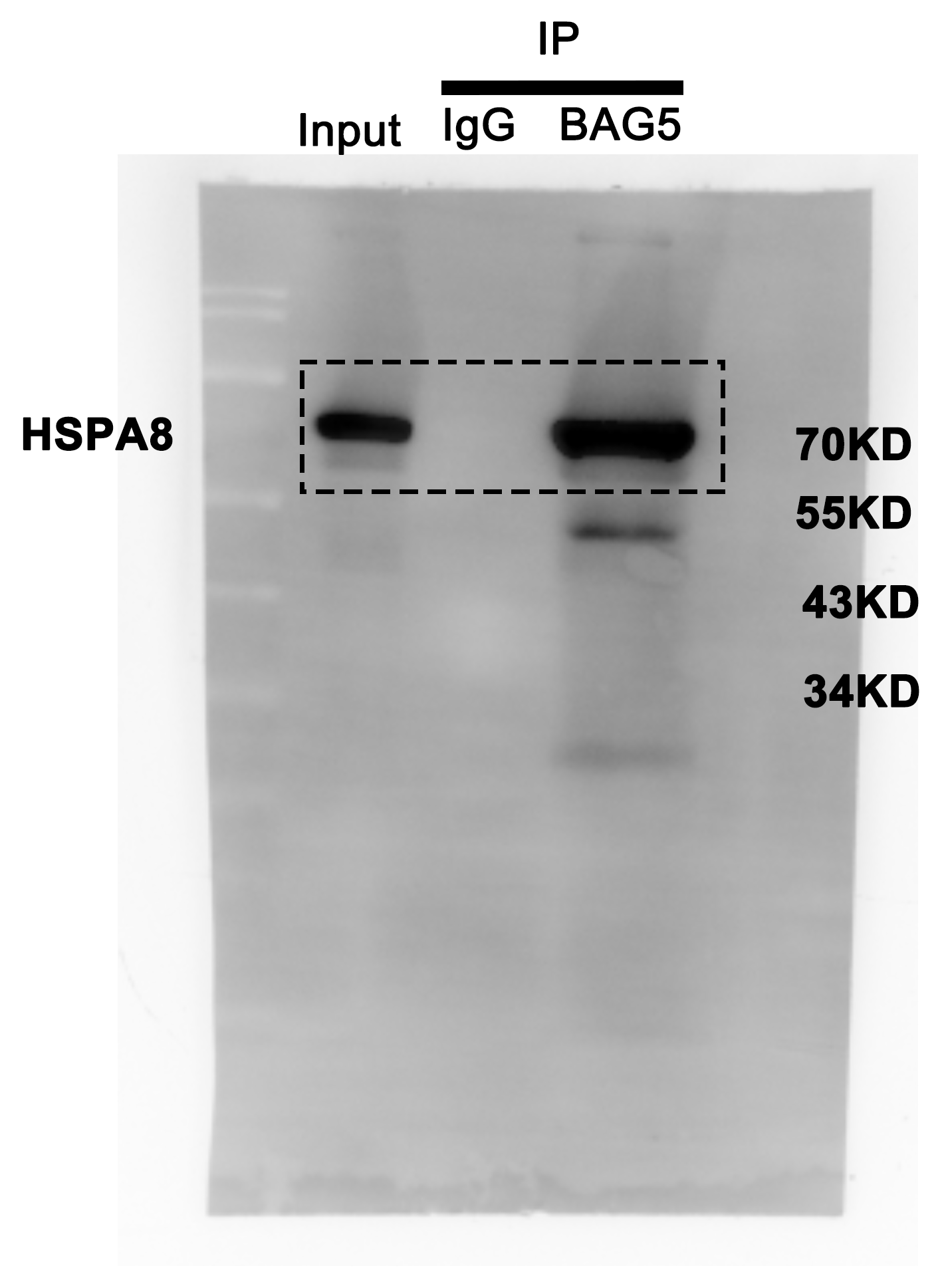

Supplement: Supplementary file 17 — Source Data Fig. 6 [file 44319_2024_112_MOESM17_ESM.zip › Figure 6/Figure 6/6E/WB HSPA8.tif]

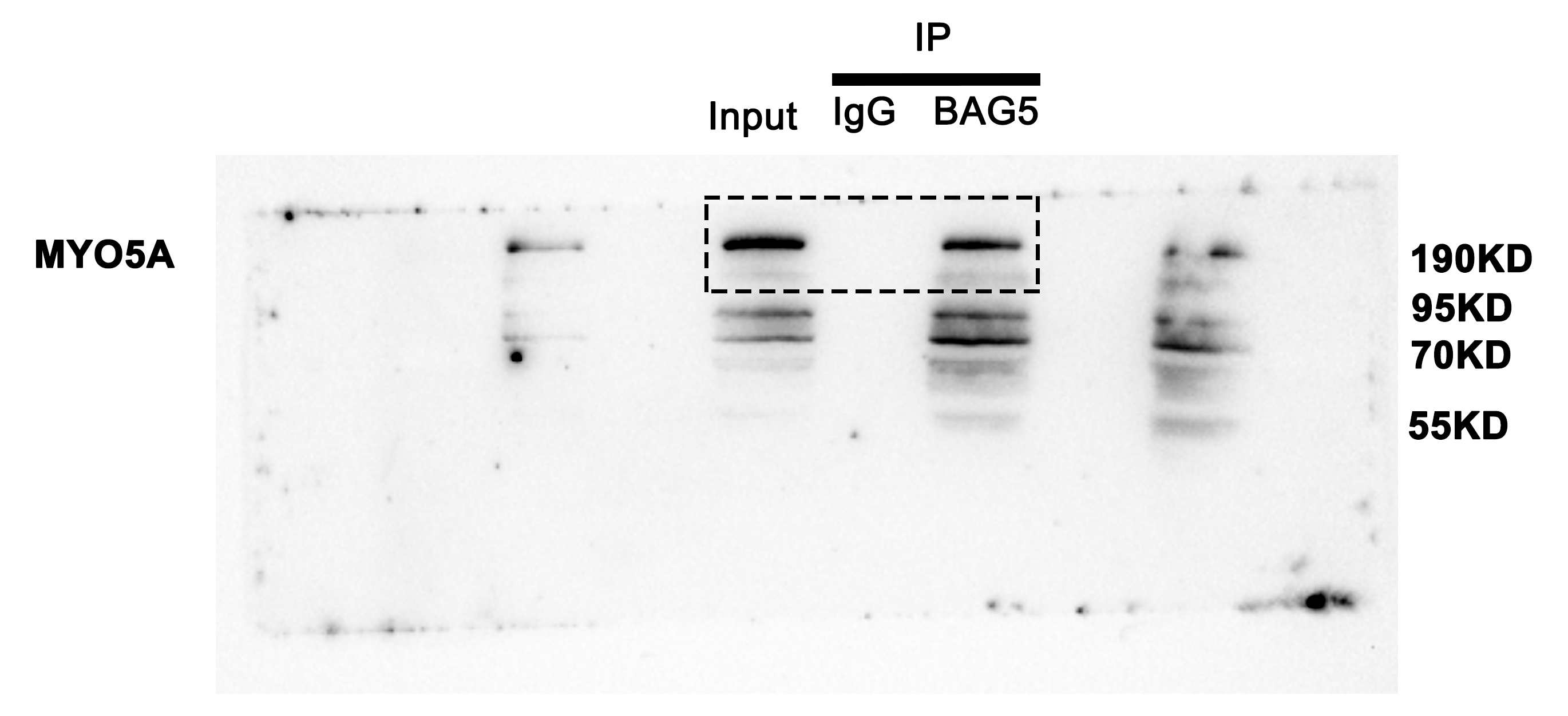

Supplement: Supplementary file 17 — Source Data Fig. 6 [file 44319_2024_112_MOESM17_ESM.zip › Figure 6/Figure 6/6E/WB MYO5A.tif]

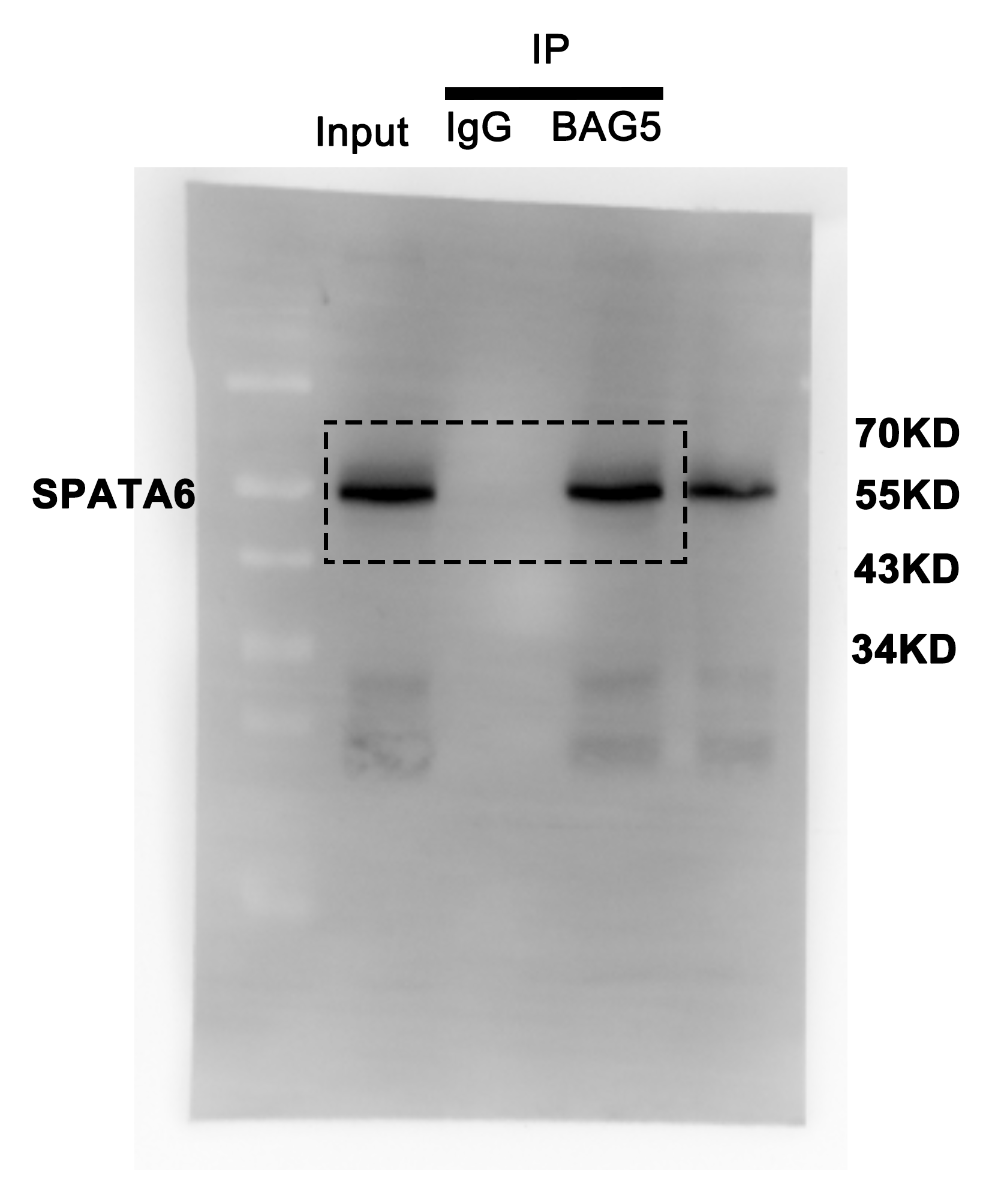

Supplement: Supplementary file 17 — Source Data Fig. 6 [file 44319_2024_112_MOESM17_ESM.zip › Figure 6/Figure 6/6E/WB SPATA6.tif]

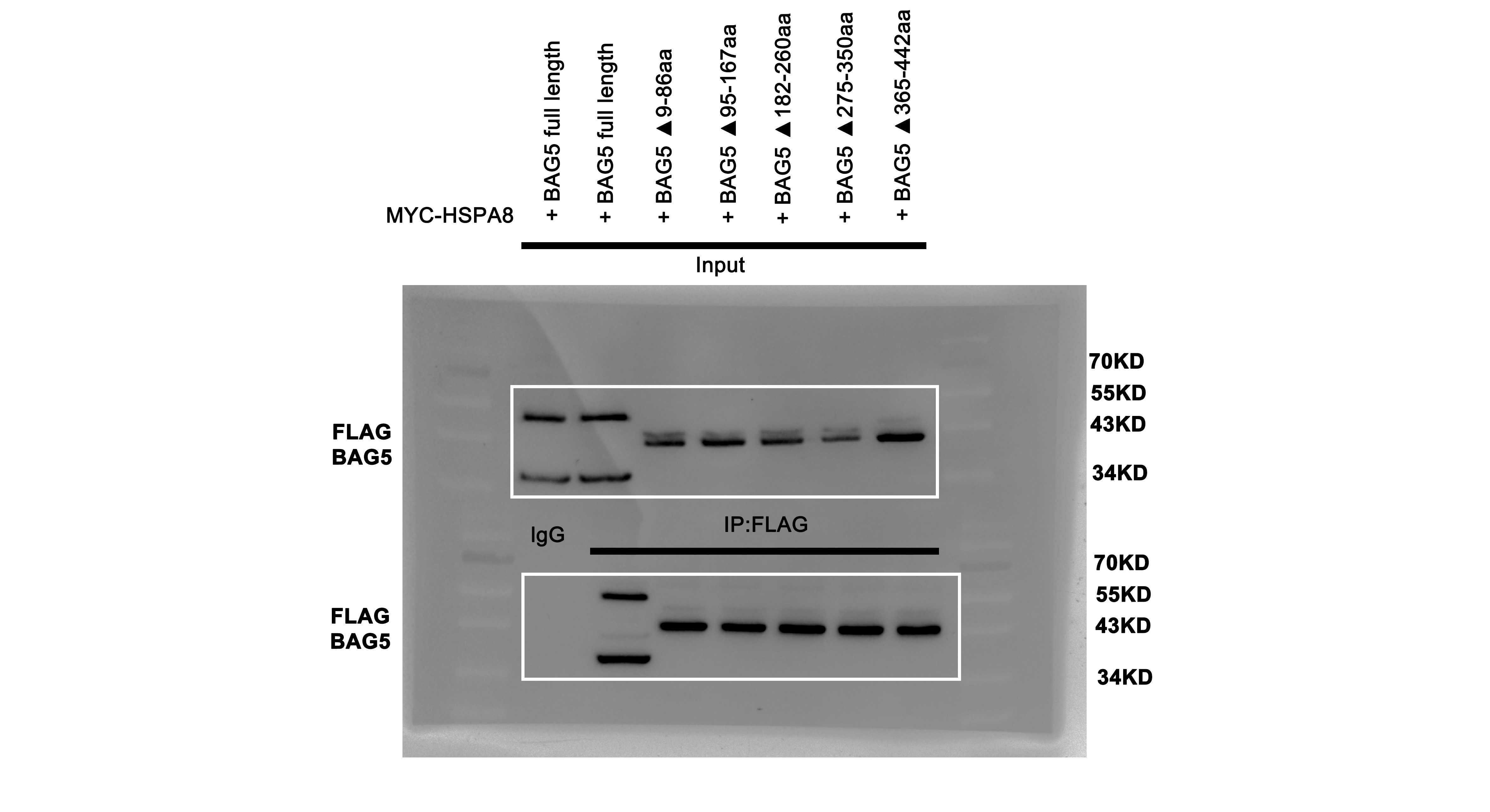

Supplement: Supplementary file 17 — Source Data Fig. 6 [file 44319_2024_112_MOESM17_ESM.zip › Figure 6/Figure 6/6G/WB BAG5.tif]

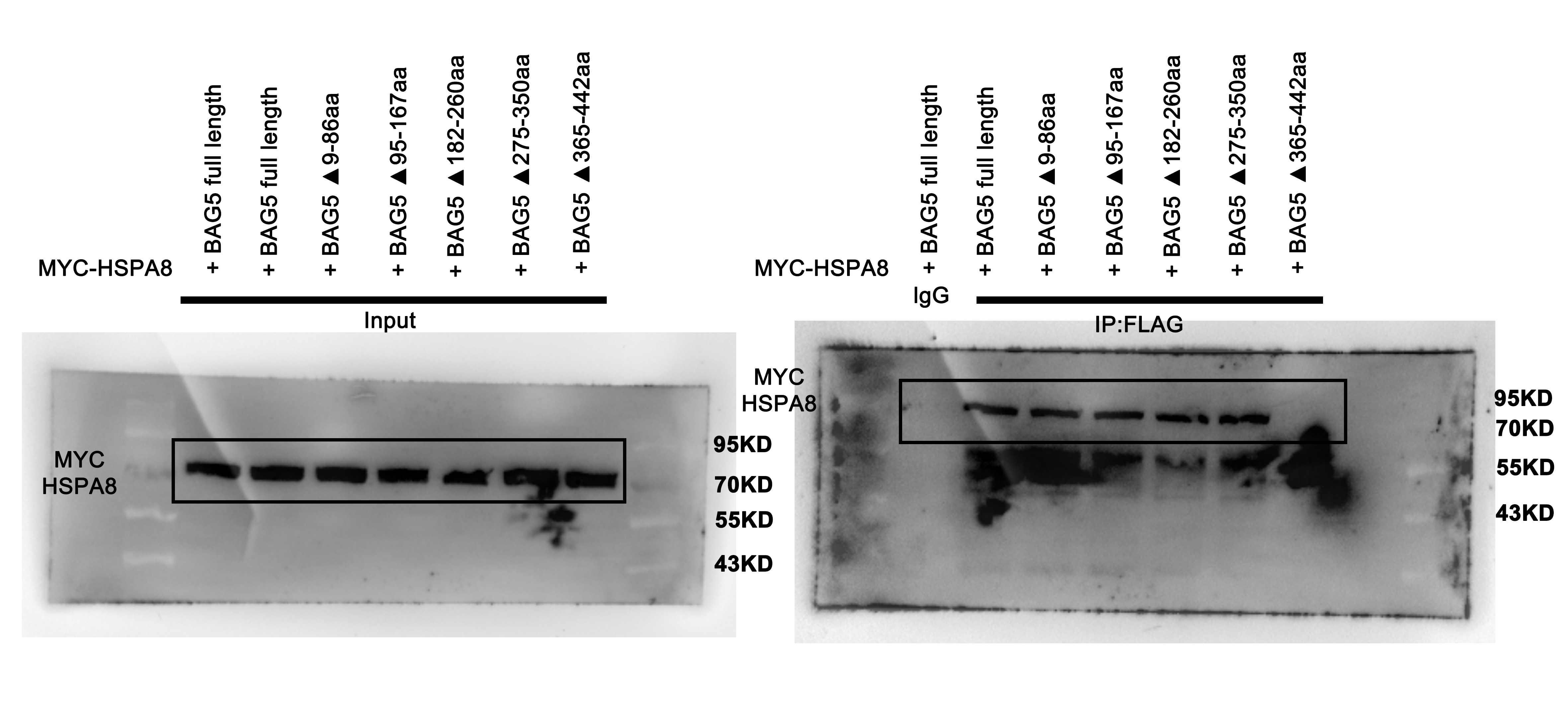

Supplement: Supplementary file 17 — Source Data Fig. 6 [file 44319_2024_112_MOESM17_ESM.zip › Figure 6/Figure 6/6G/WB HSPA8.tif]

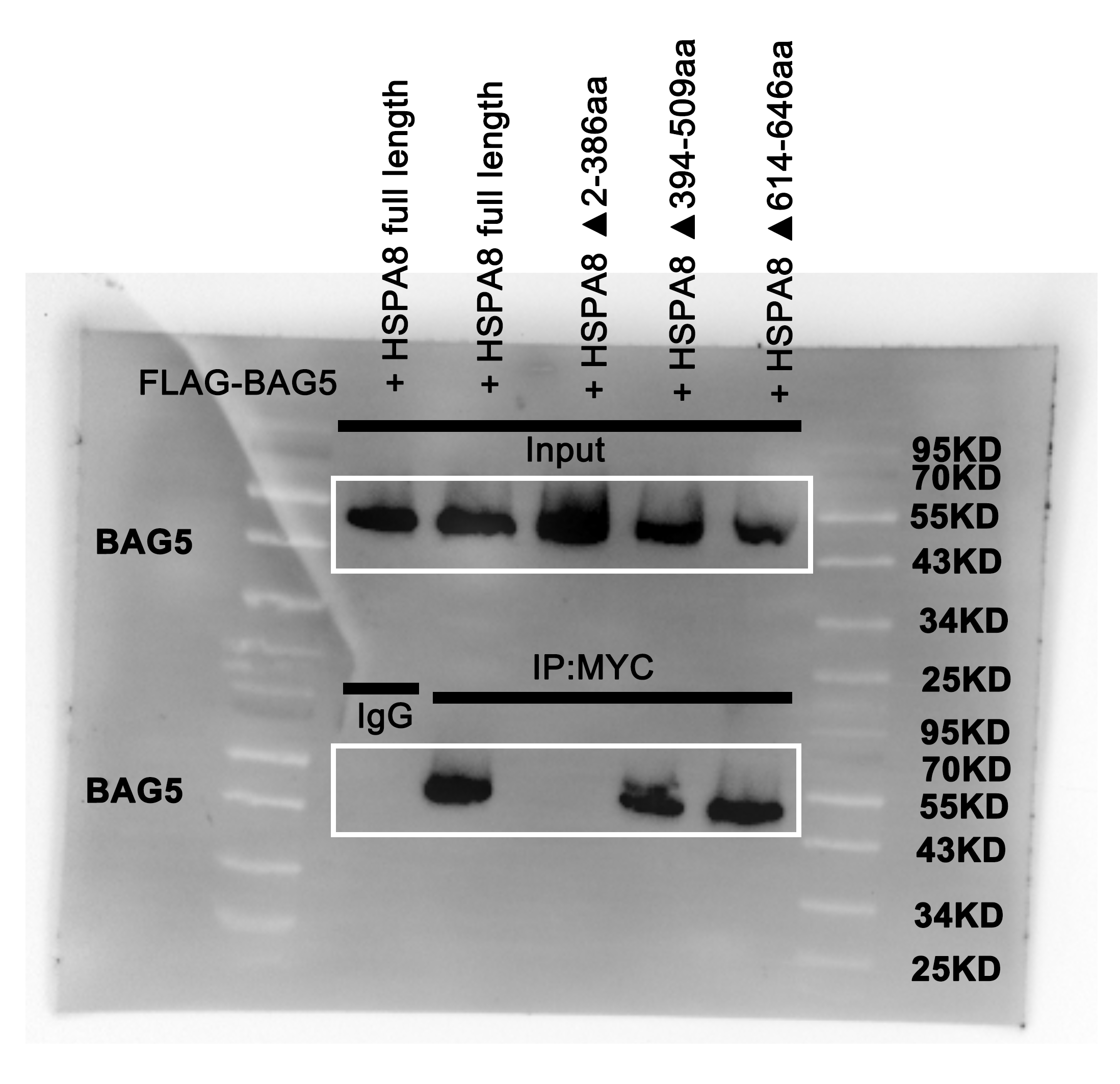

Supplement: Supplementary file 17 — Source Data Fig. 6 [file 44319_2024_112_MOESM17_ESM.zip › Figure 6/Figure 6/6H/WB BAG5.tif]

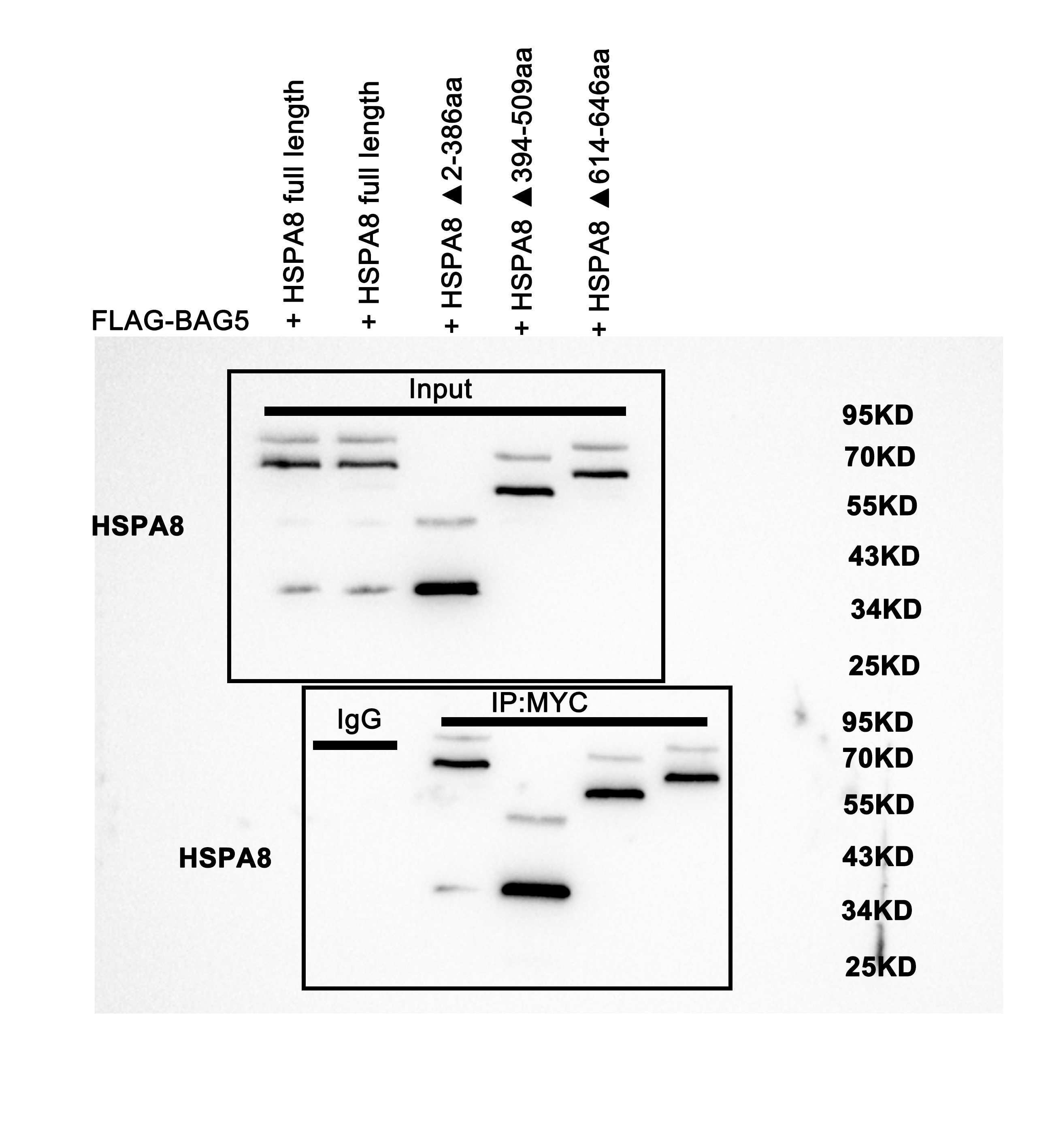

Supplement: Supplementary file 17 — Source Data Fig. 6 [file 44319_2024_112_MOESM17_ESM.zip › Figure 6/Figure 6/6H/WB HSPA8.tif]

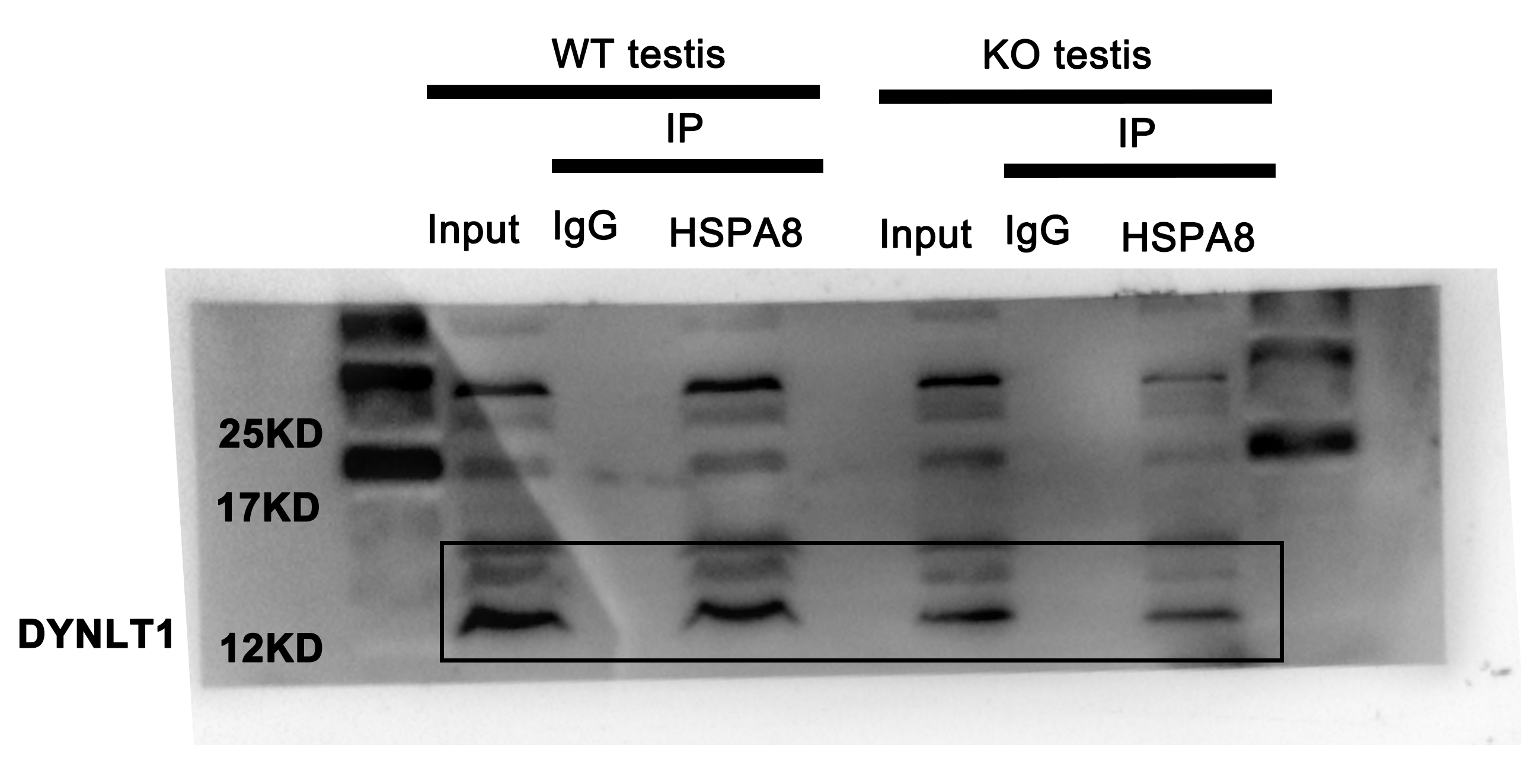

Supplement: Supplementary file 18 — Source Data Fig. 7 [file 44319_2024_112_MOESM18_ESM.zip › Figure 7/Figure 7/7F/WB DYNLT1.tif]

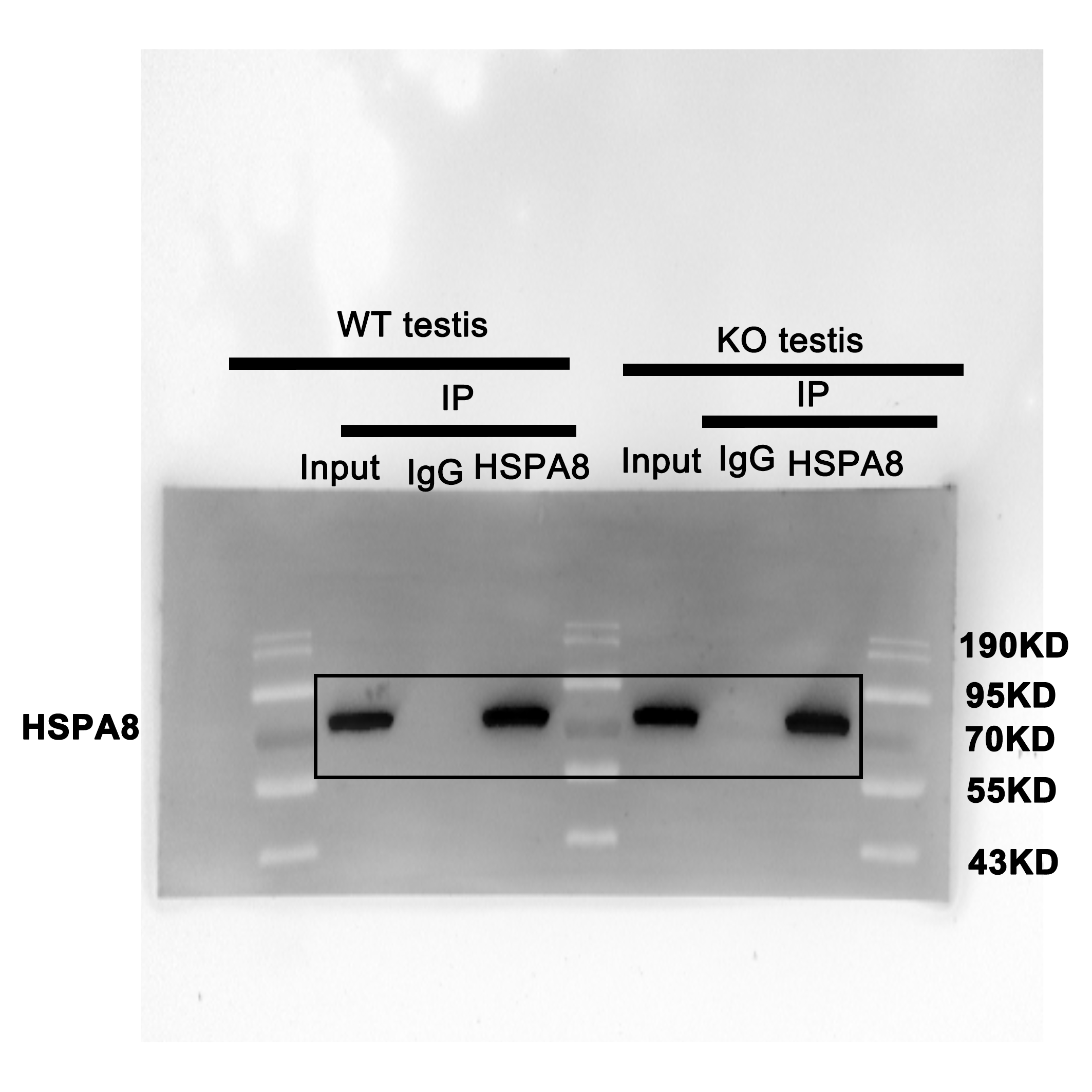

Supplement: Supplementary file 18 — Source Data Fig. 7 [file 44319_2024_112_MOESM18_ESM.zip › Figure 7/Figure 7/7F/WB HSPA8.tif]

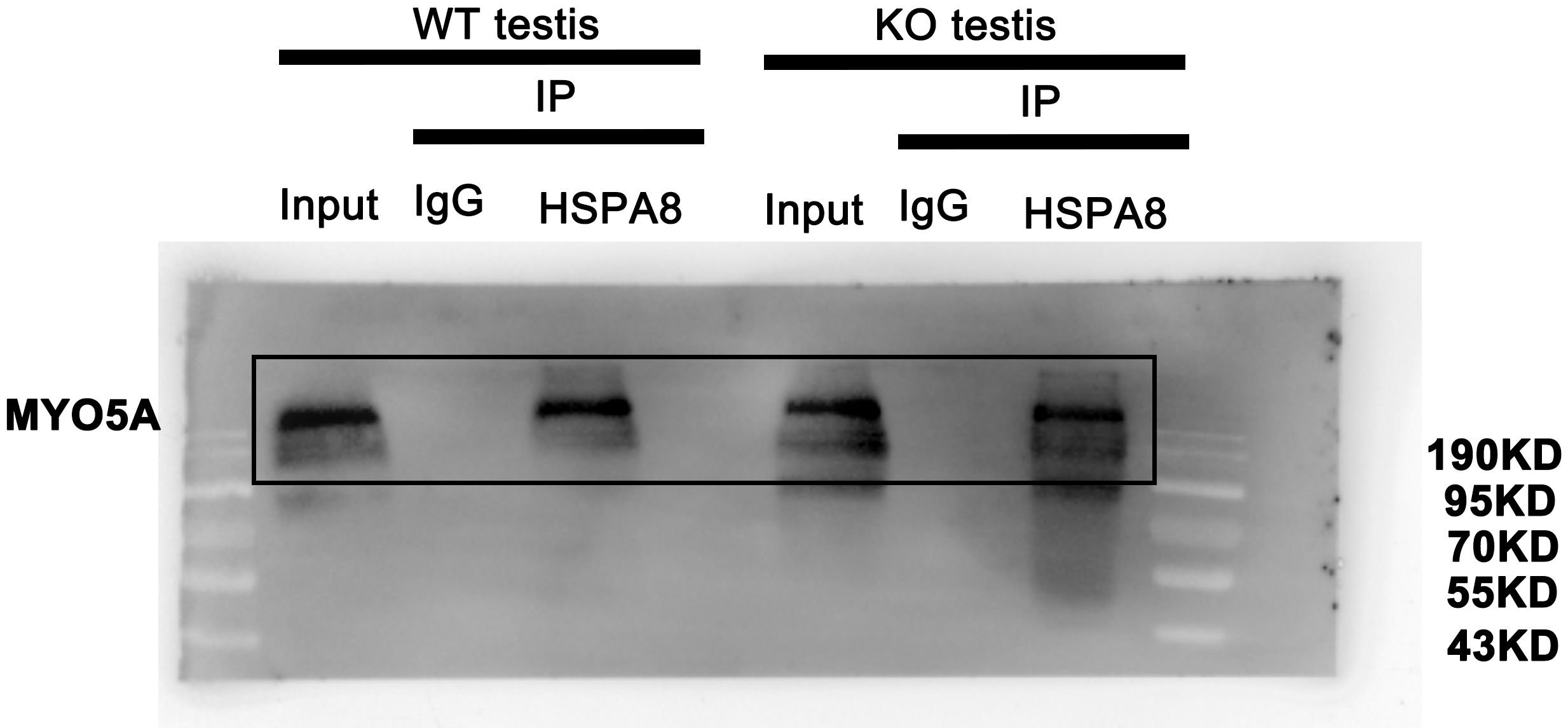

Supplement: Supplementary file 18 — Source Data Fig. 7 [file 44319_2024_112_MOESM18_ESM.zip › Figure 7/Figure 7/7F/WB MYO5A.tif]

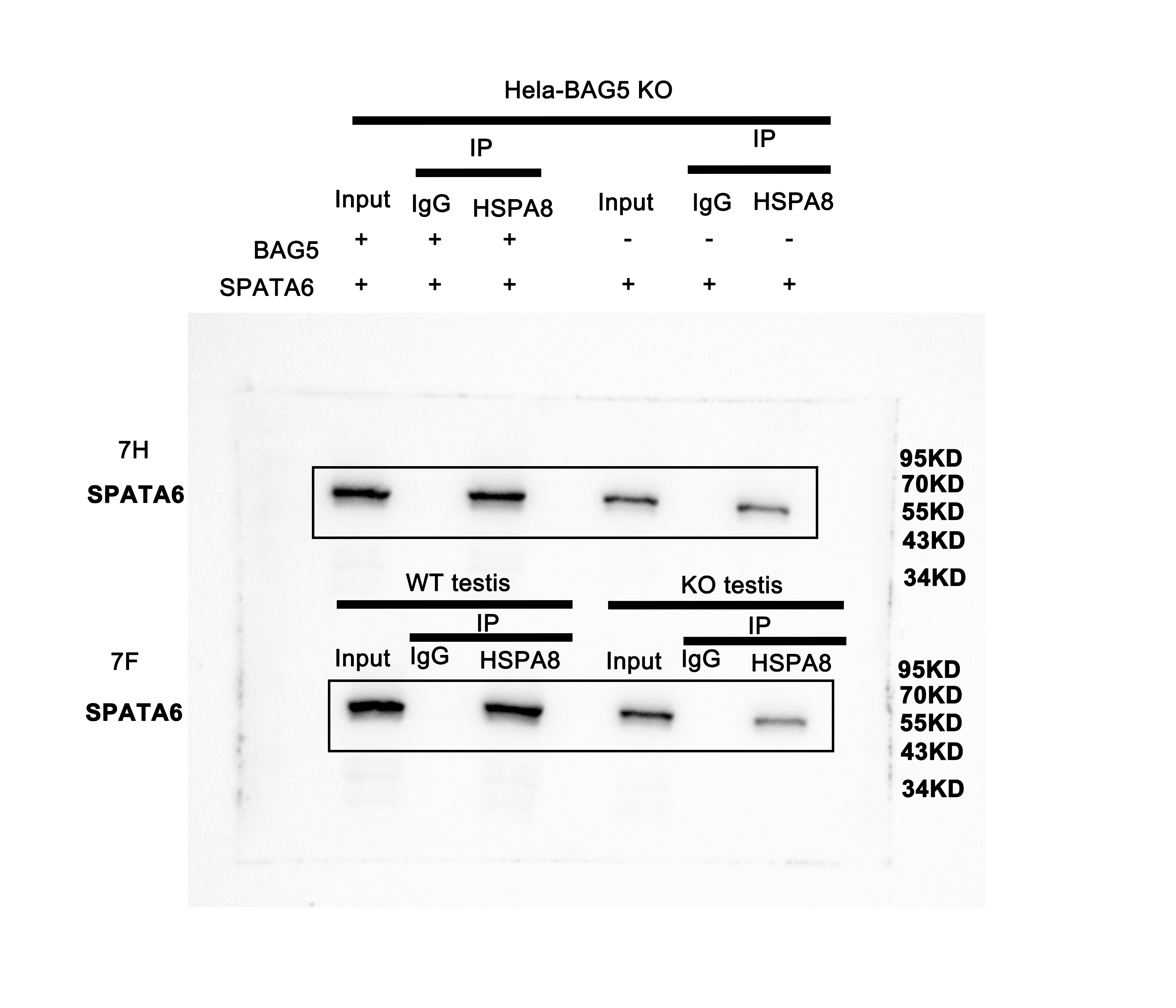

Supplement: Supplementary file 18 — Source Data Fig. 7 [file 44319_2024_112_MOESM18_ESM.zip › Figure 7/Figure 7/7F/WB SPATA6.tif]

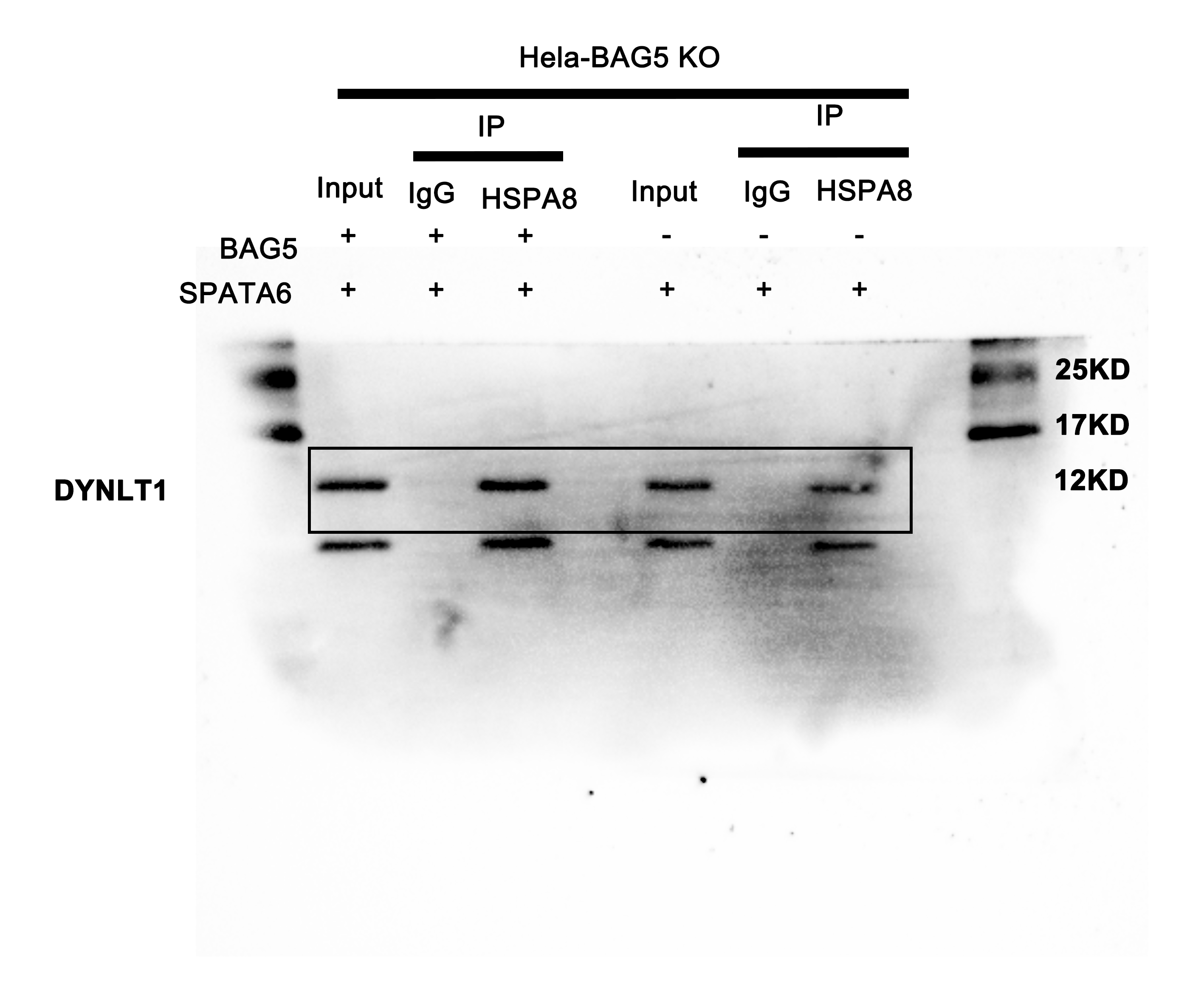

Supplement: Supplementary file 18 — Source Data Fig. 7 [file 44319_2024_112_MOESM18_ESM.zip › Figure 7/Figure 7/7H/WB DYNLT1.tif]

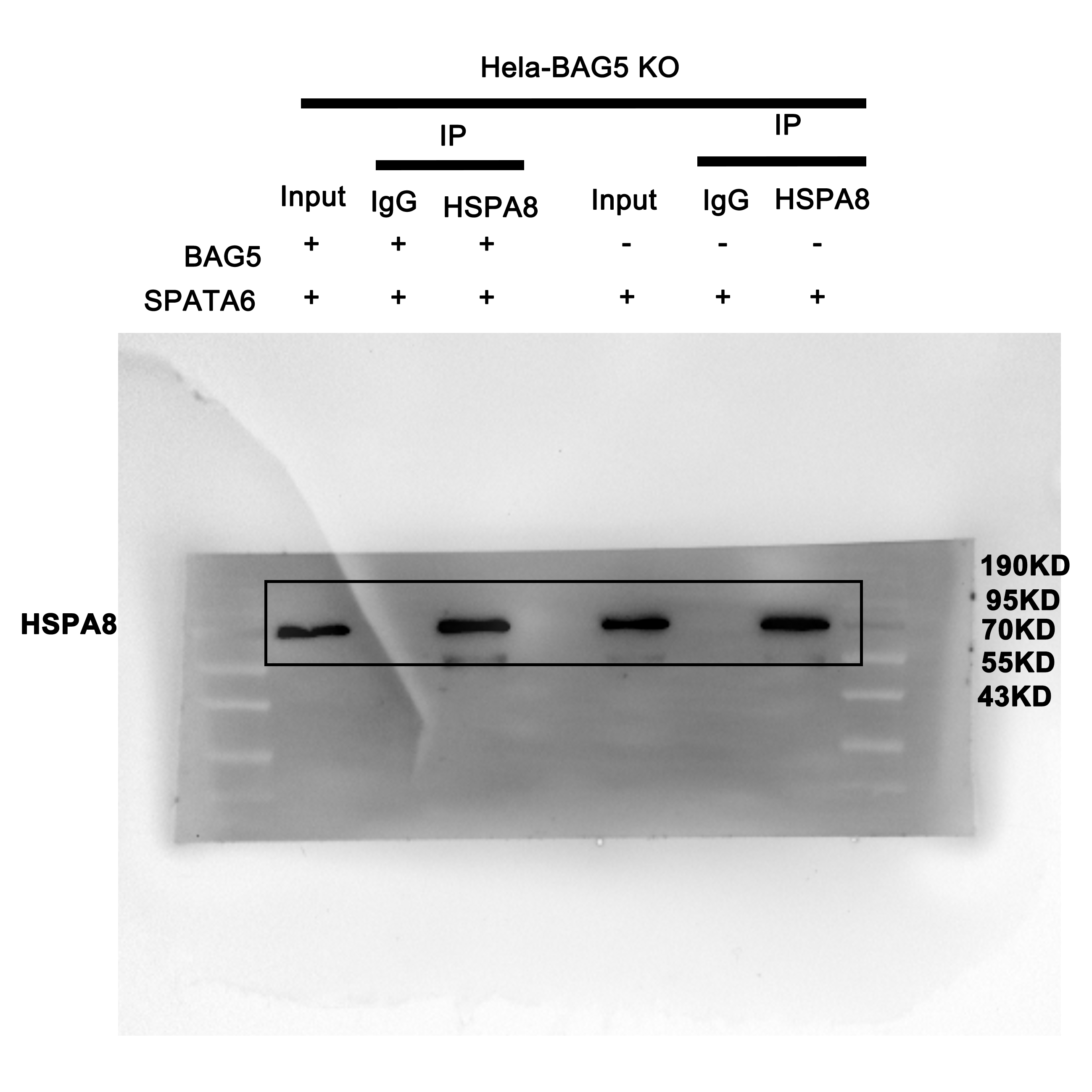

Supplement: Supplementary file 18 — Source Data Fig. 7 [file 44319_2024_112_MOESM18_ESM.zip › Figure 7/Figure 7/7H/WB HSPA8.tif]

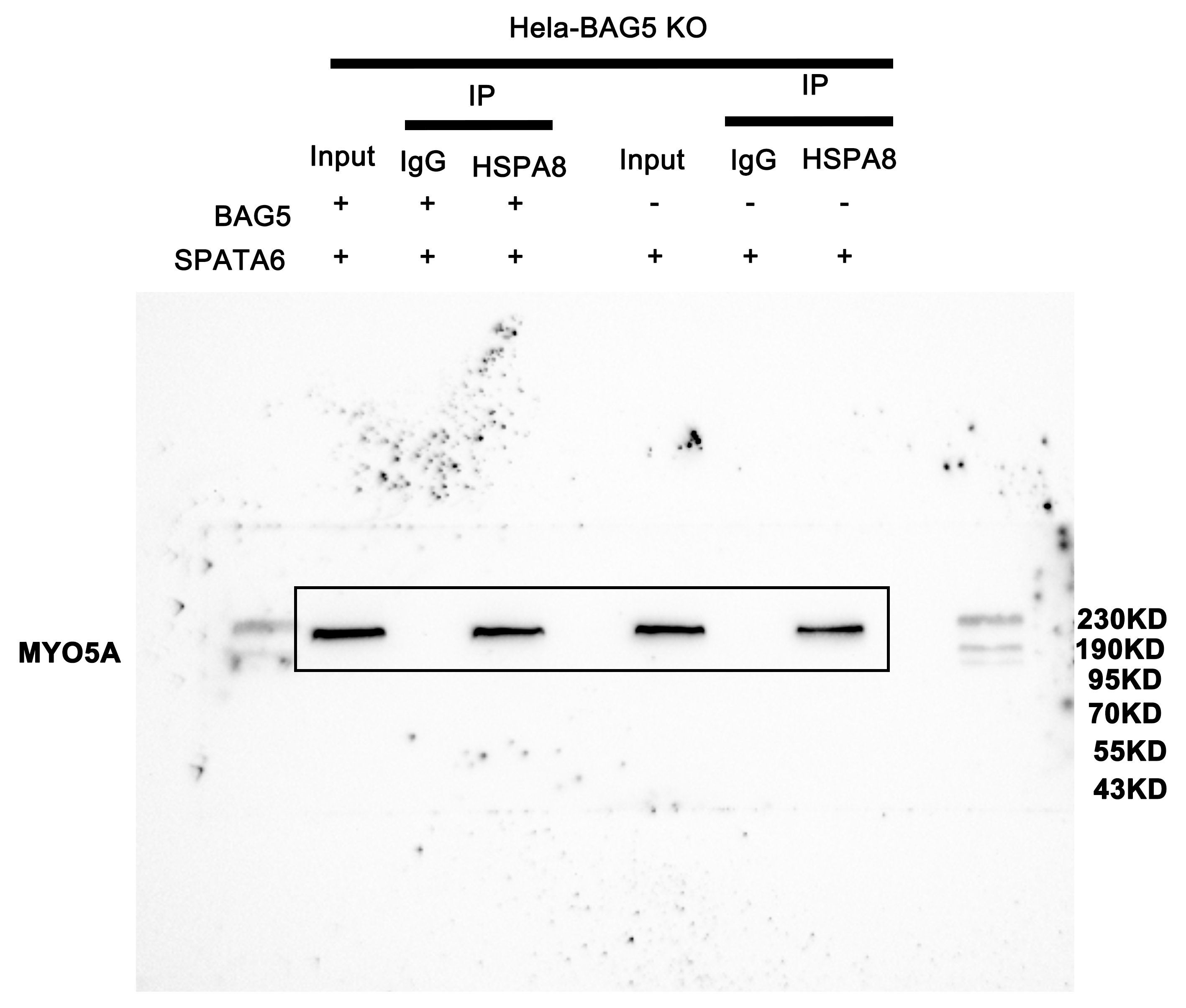

Supplement: Supplementary file 18 — Source Data Fig. 7 [file 44319_2024_112_MOESM18_ESM.zip › Figure 7/Figure 7/7H/WB MYO5A.tif]

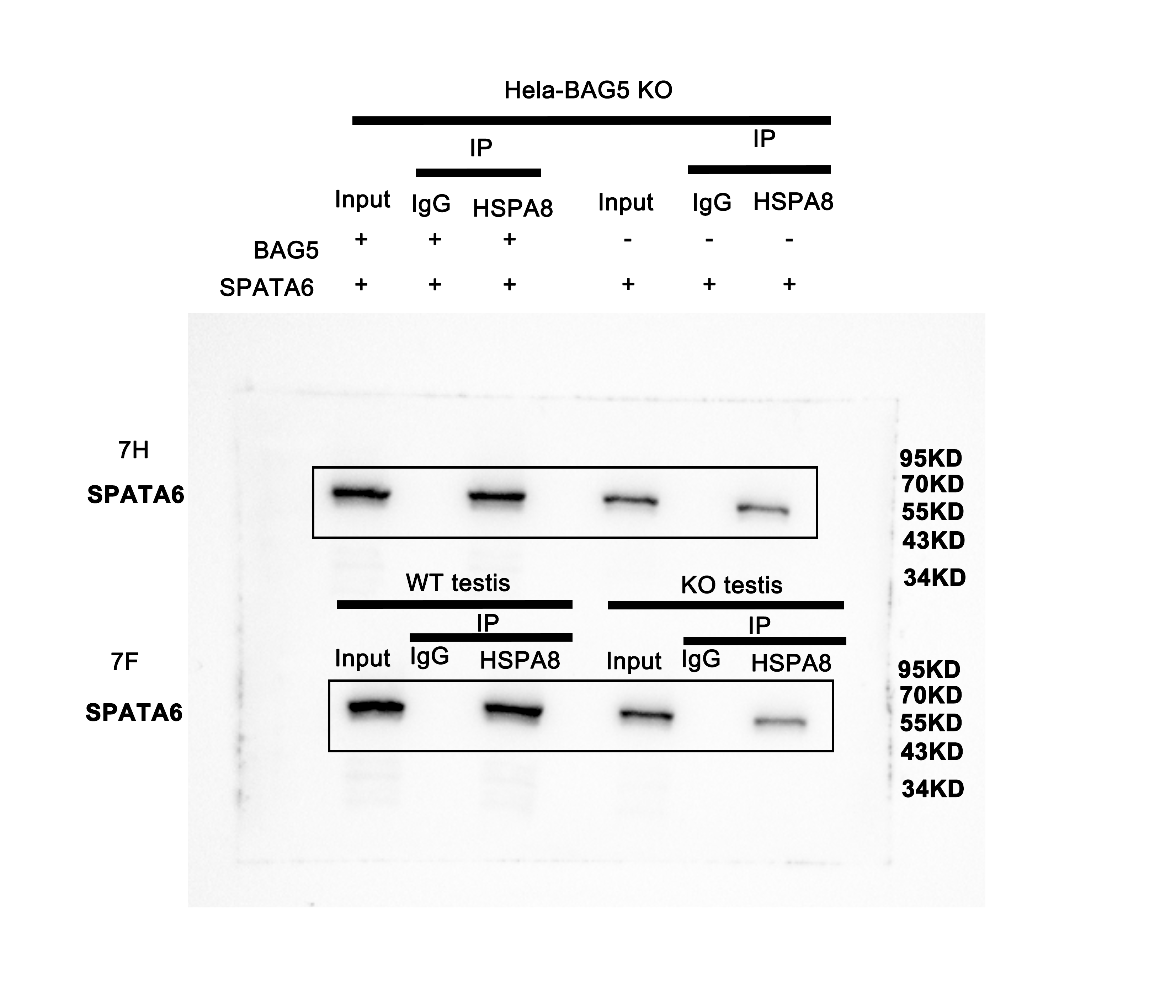

Supplement: Supplementary file 18 — Source Data Fig. 7 [file 44319_2024_112_MOESM18_ESM.zip › Figure 7/Figure 7/7H/WB SPATA6.tif]

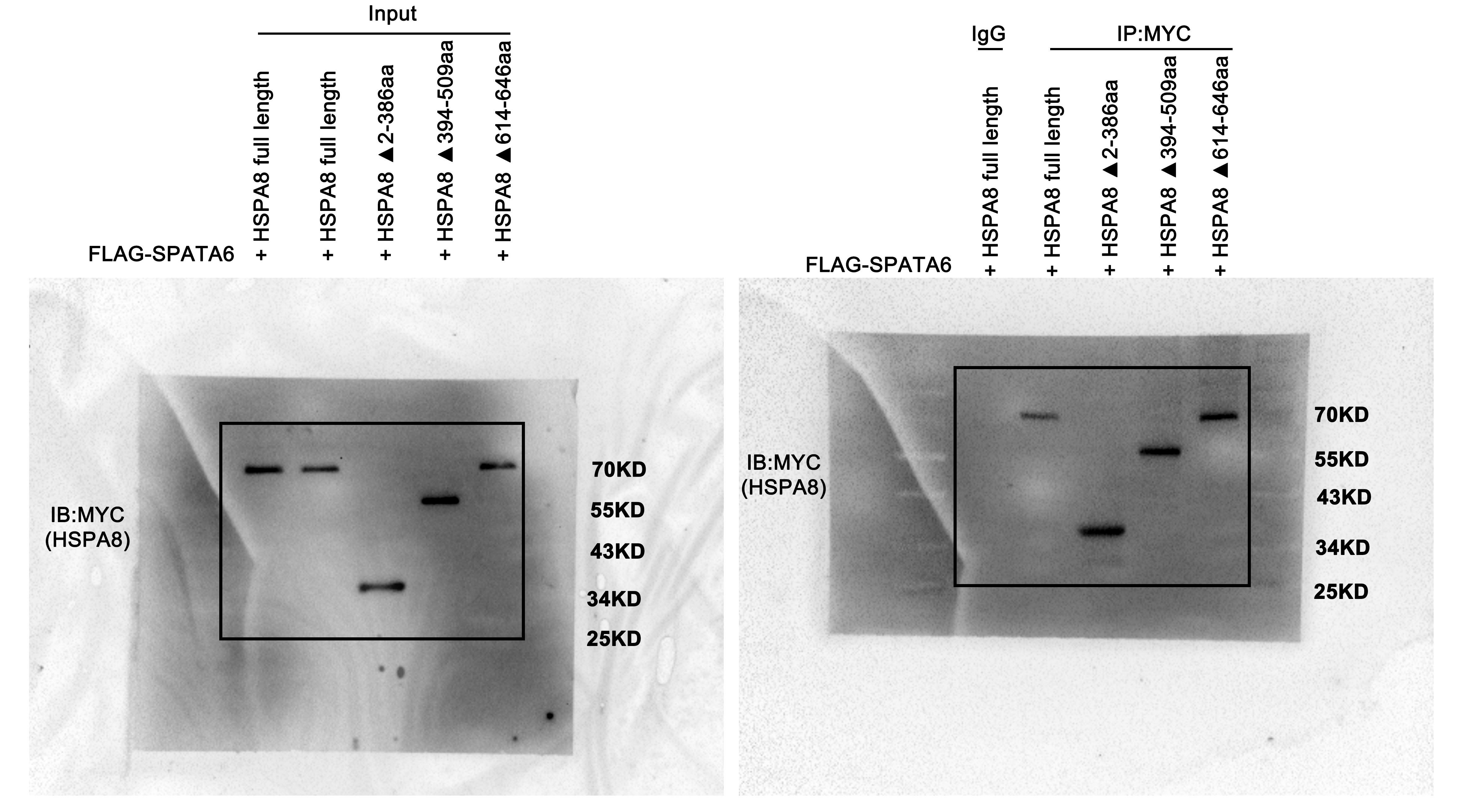

Supplement: Supplementary file 19 — Source Data Fig. 8 [file 44319_2024_112_MOESM19_ESM.zip › Figure 8/Figure 8/8A/WB HSPA8.tif]

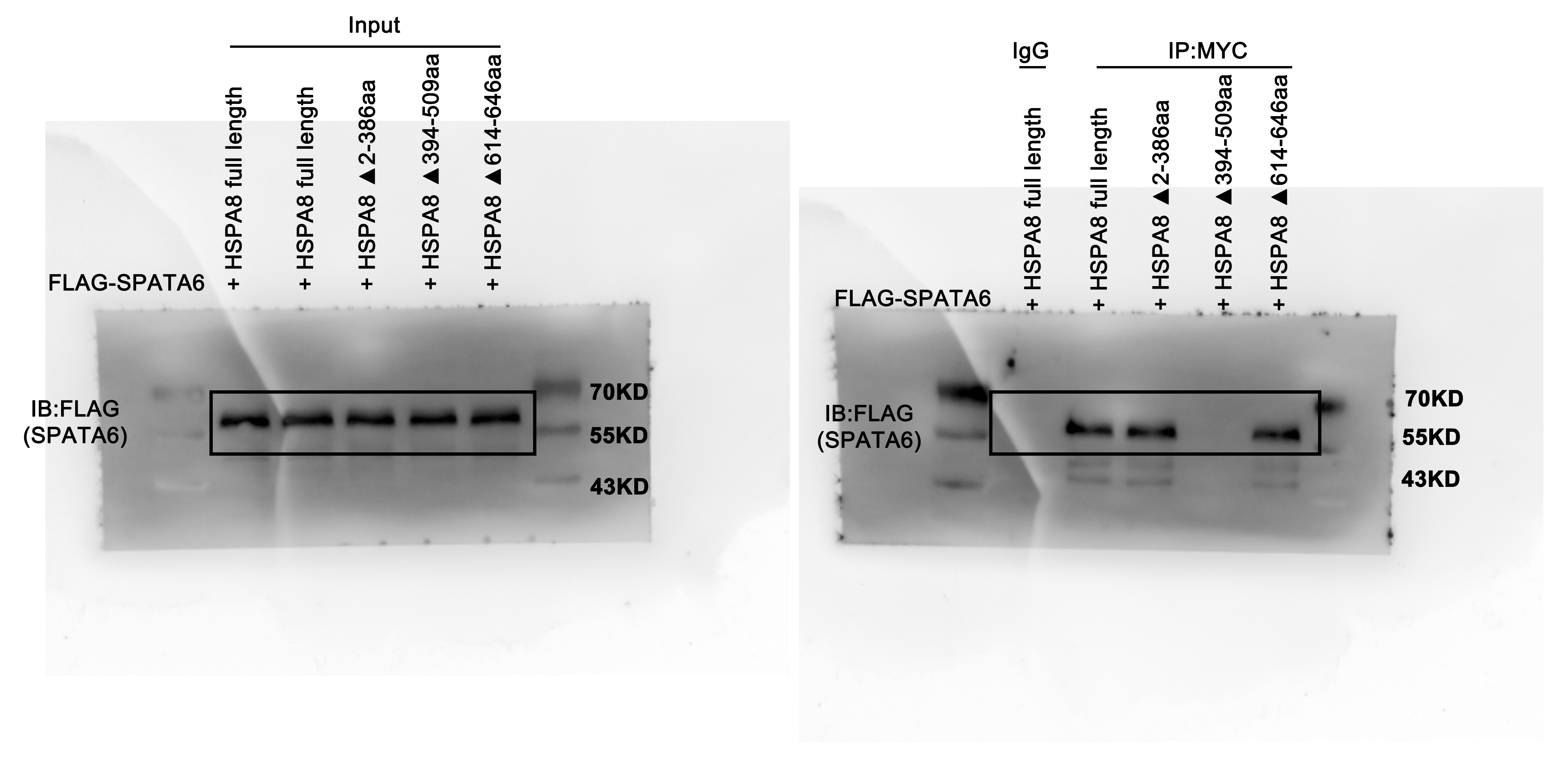

Supplement: Supplementary file 19 — Source Data Fig. 8 [file 44319_2024_112_MOESM19_ESM.zip › Figure 8/Figure 8/8A/WB SPATA6.tif]

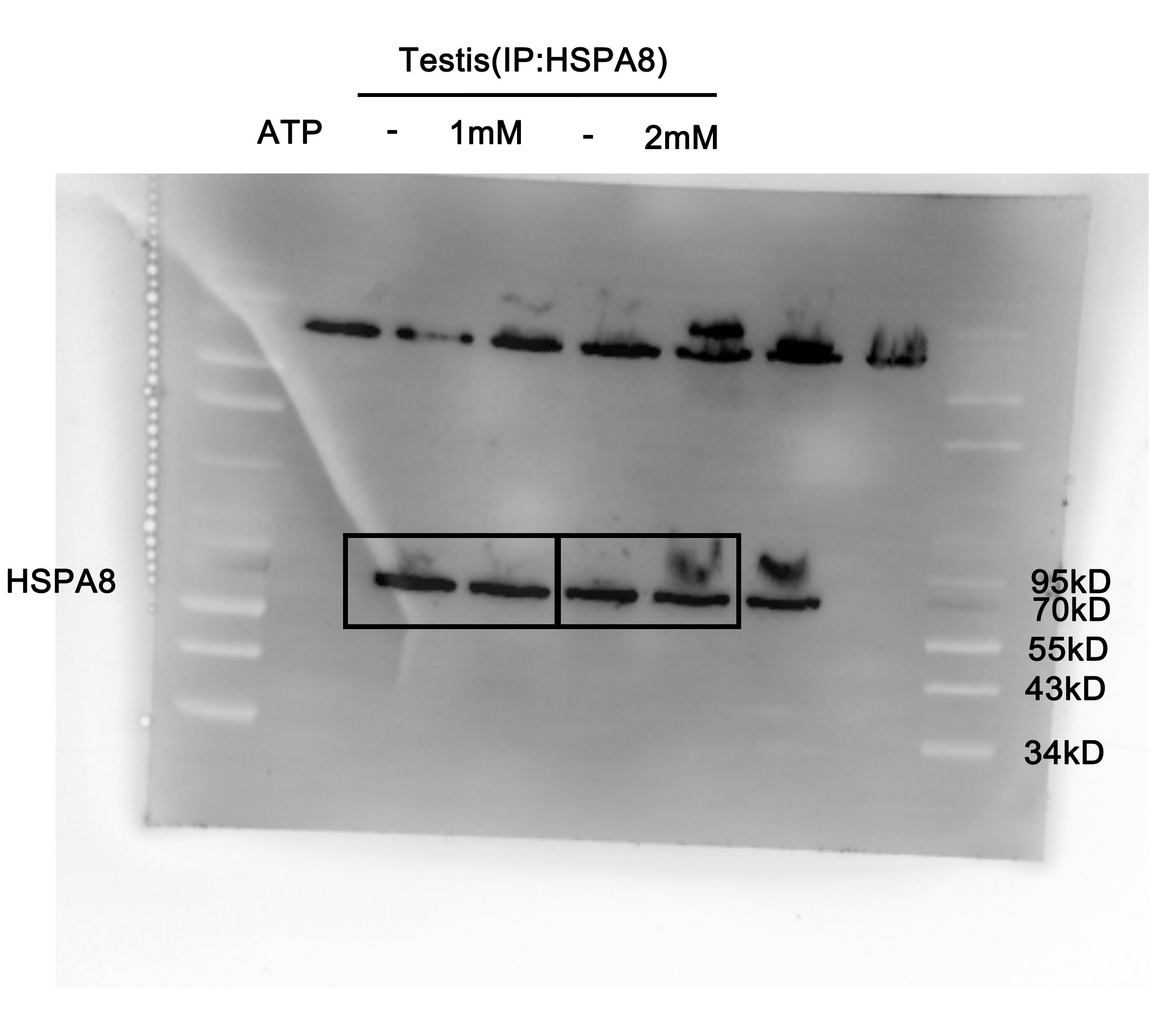

Supplement: Supplementary file 19 — Source Data Fig. 8 [file 44319_2024_112_MOESM19_ESM.zip › Figure 8/Figure 8/8B/WB HSPA8.tif]

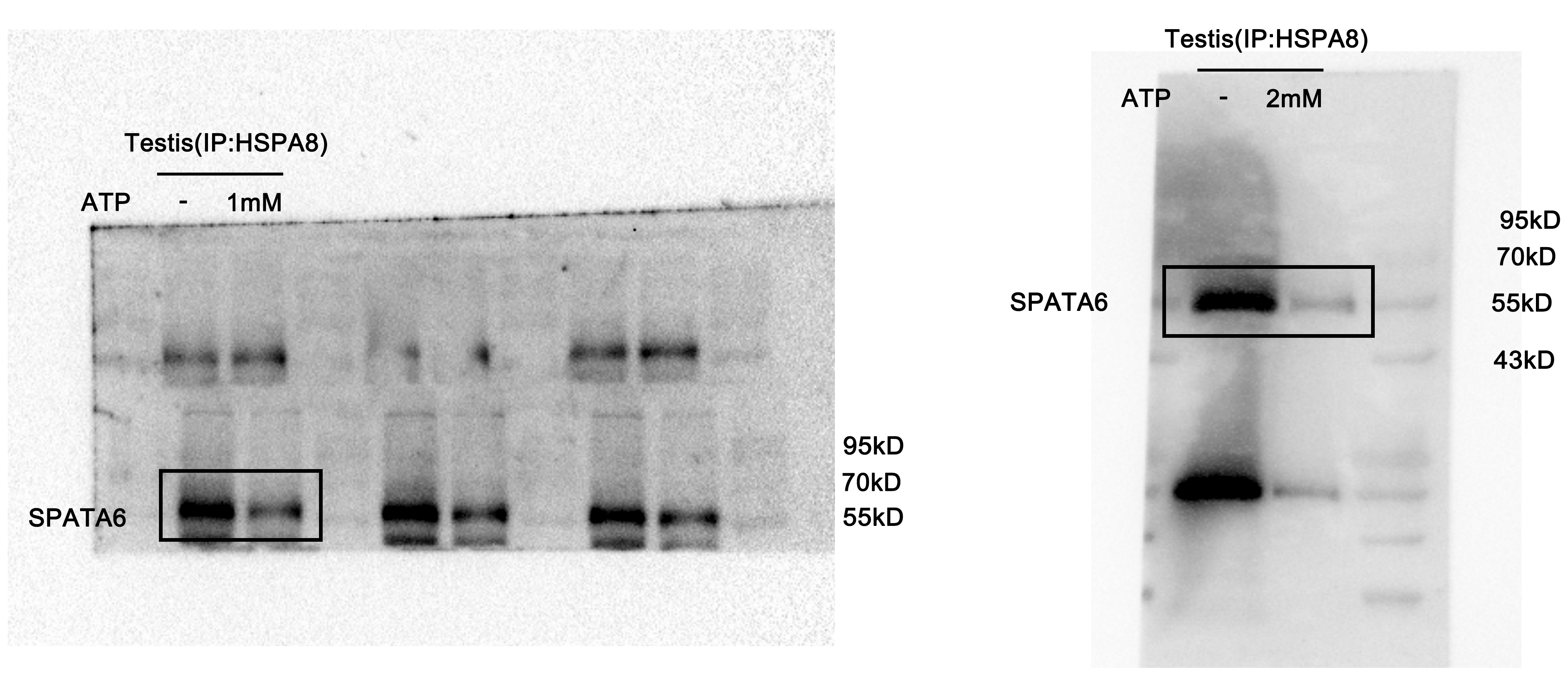

Supplement: Supplementary file 19 — Source Data Fig. 8 [file 44319_2024_112_MOESM19_ESM.zip › Figure 8/Figure 8/8B/WB SPATA6.tif]

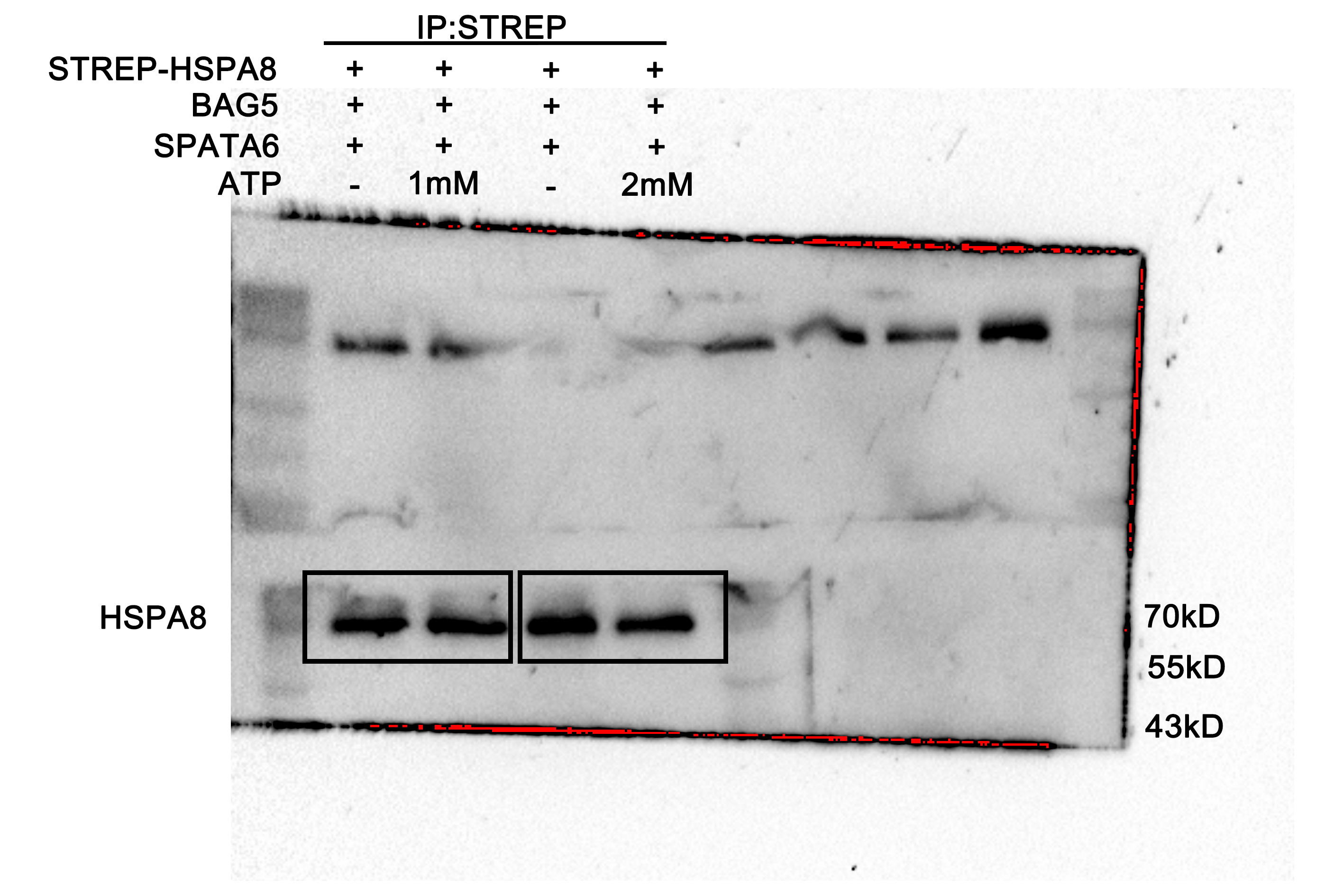

Supplement: Supplementary file 19 — Source Data Fig. 8 [file 44319_2024_112_MOESM19_ESM.zip › Figure 8/Figure 8/8D/WB HSPA8.tif]

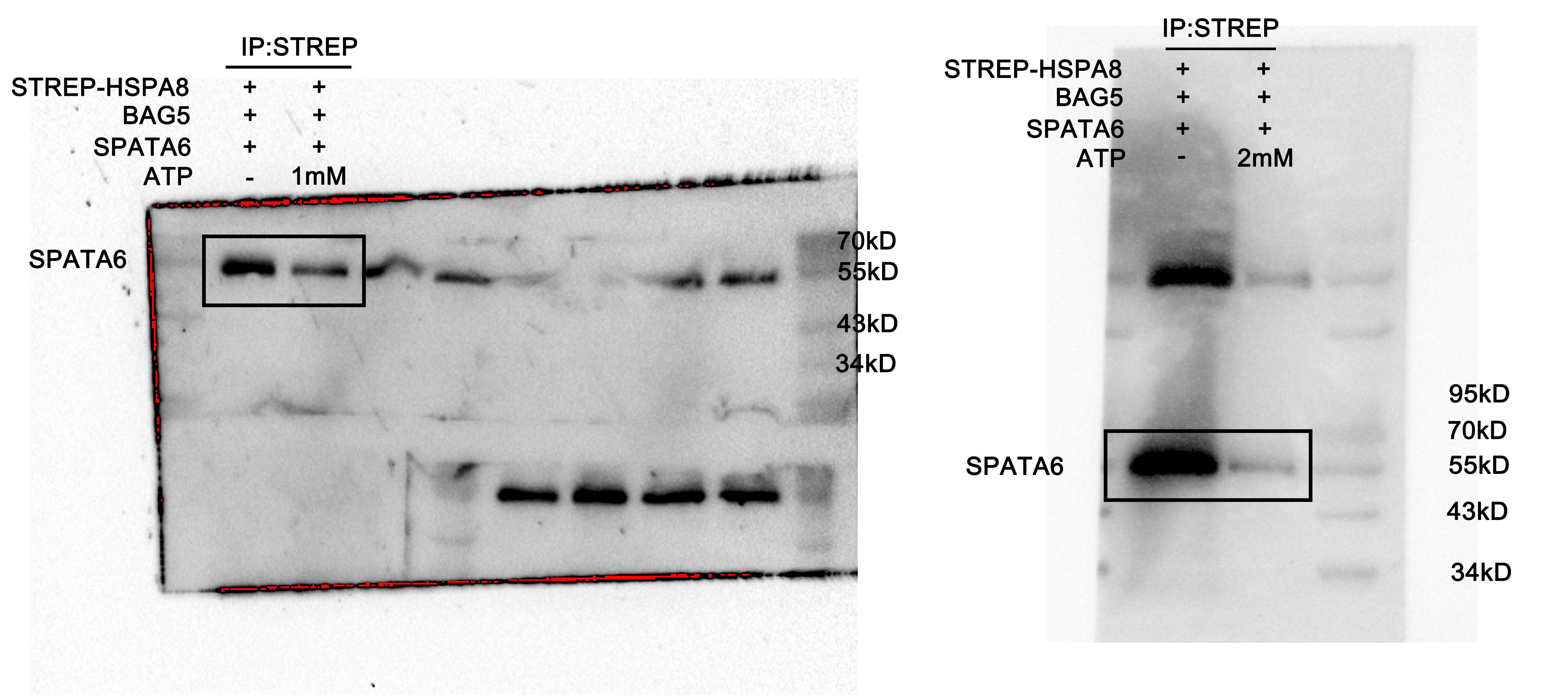

Supplement: Supplementary file 19 — Source Data Fig. 8 [file 44319_2024_112_MOESM19_ESM.zip › Figure 8/Figure 8/8D/WB SPATA6.tif]

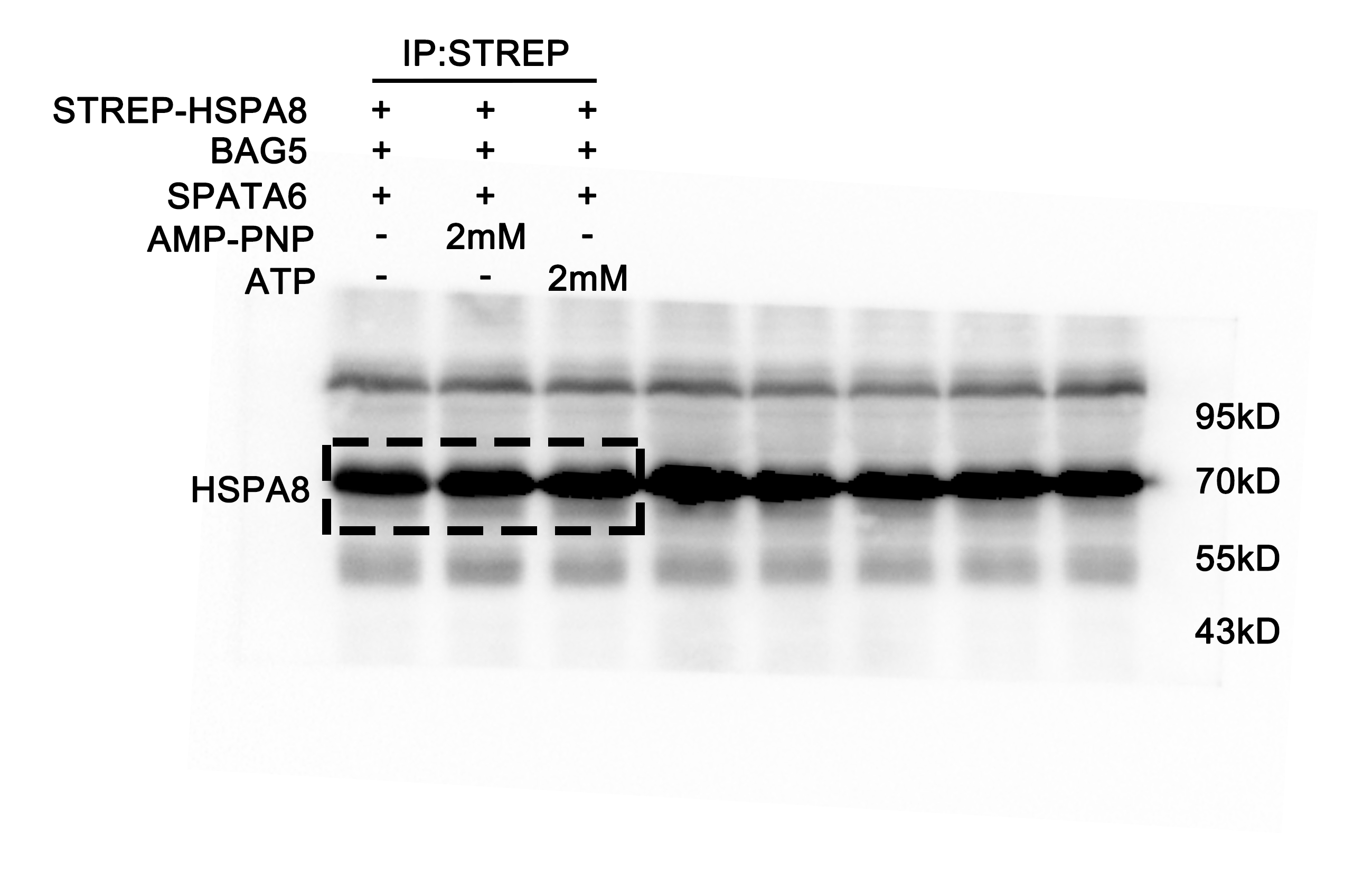

Supplement: Supplementary file 19 — Source Data Fig. 8 [file 44319_2024_112_MOESM19_ESM.zip › Figure 8/Figure 8/8F/WB HSPA8.tif]

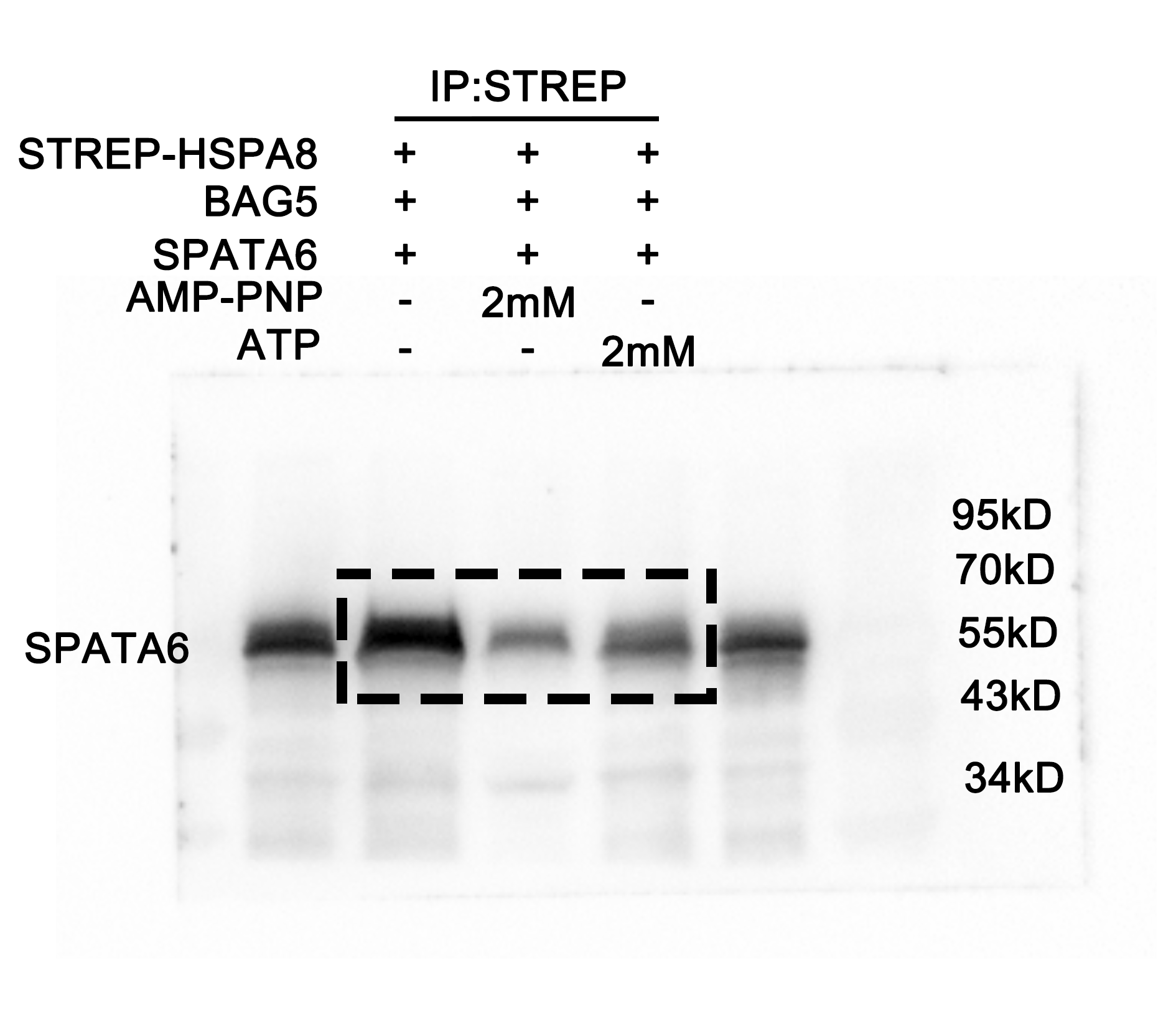

Supplement: Supplementary file 19 — Source Data Fig. 8 [file 44319_2024_112_MOESM19_ESM.zip › Figure 8/Figure 8/8F/WB SPATA6.tif]

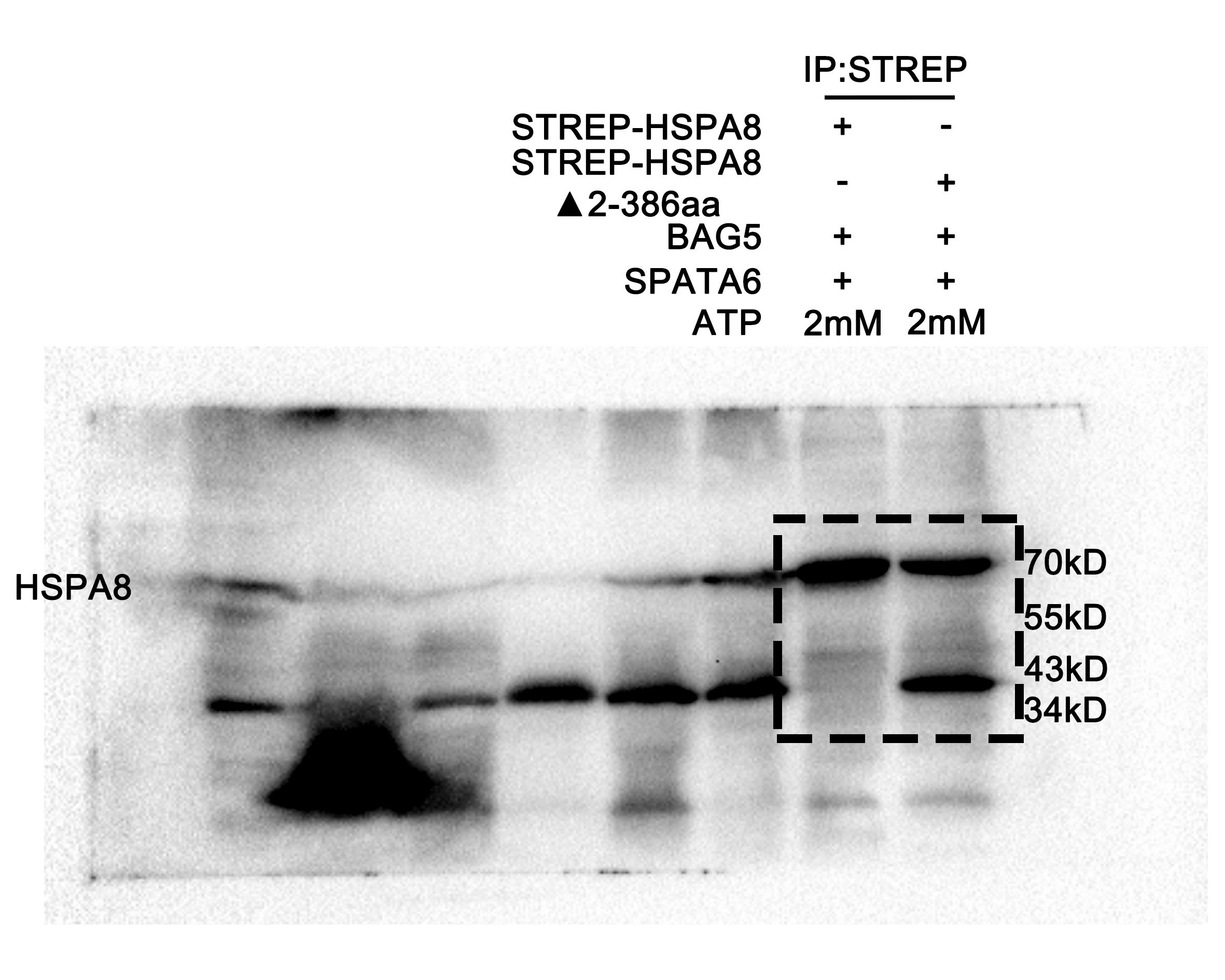

Supplement: Supplementary file 19 — Source Data Fig. 8 [file 44319_2024_112_MOESM19_ESM.zip › Figure 8/Figure 8/8H/WB HSPA8.tif]

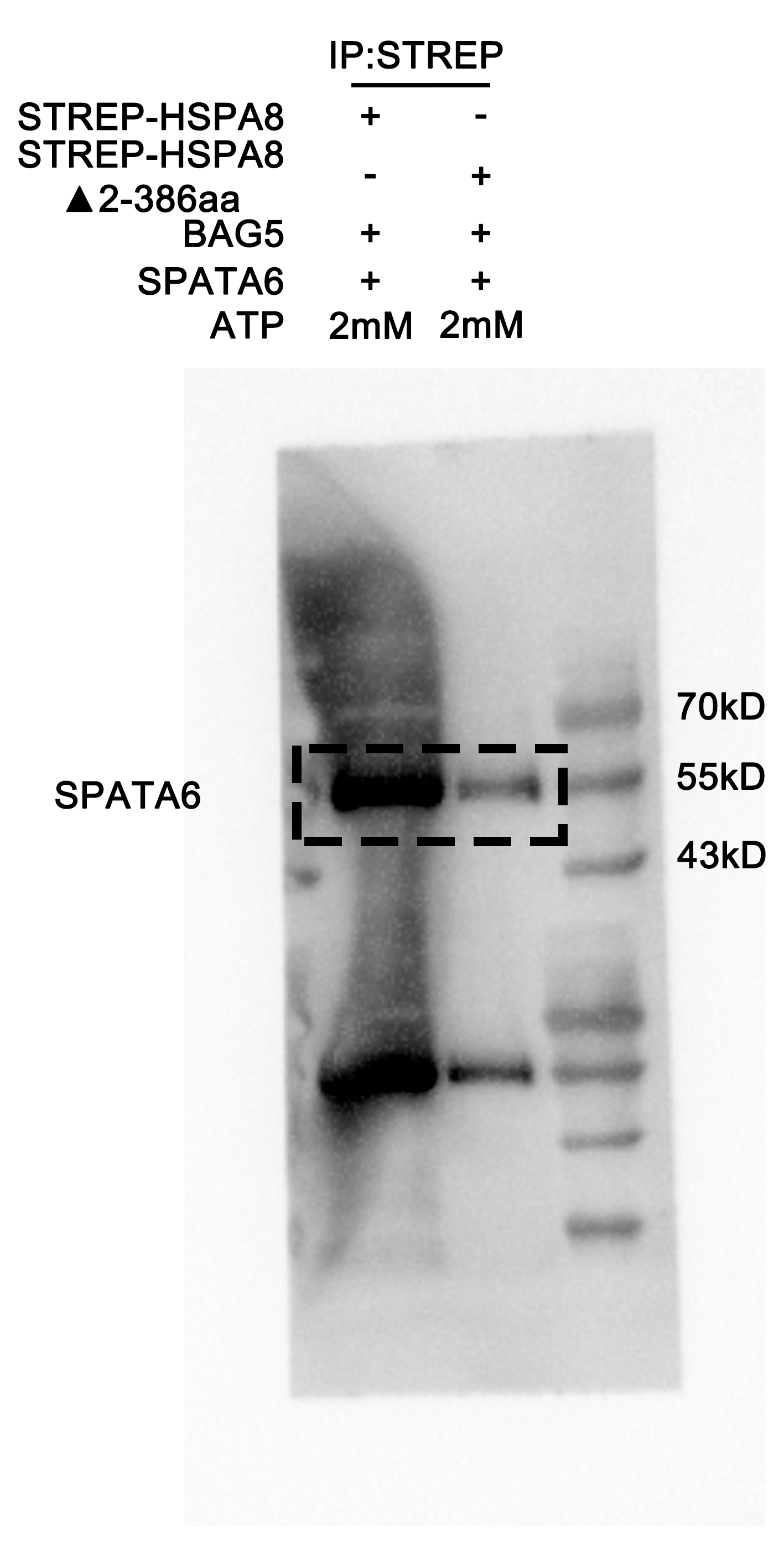

Supplement: Supplementary file 19 — Source Data Fig. 8 [file 44319_2024_112_MOESM19_ESM.zip › Figure 8/Figure 8/8H/WB SPATA6.tif]

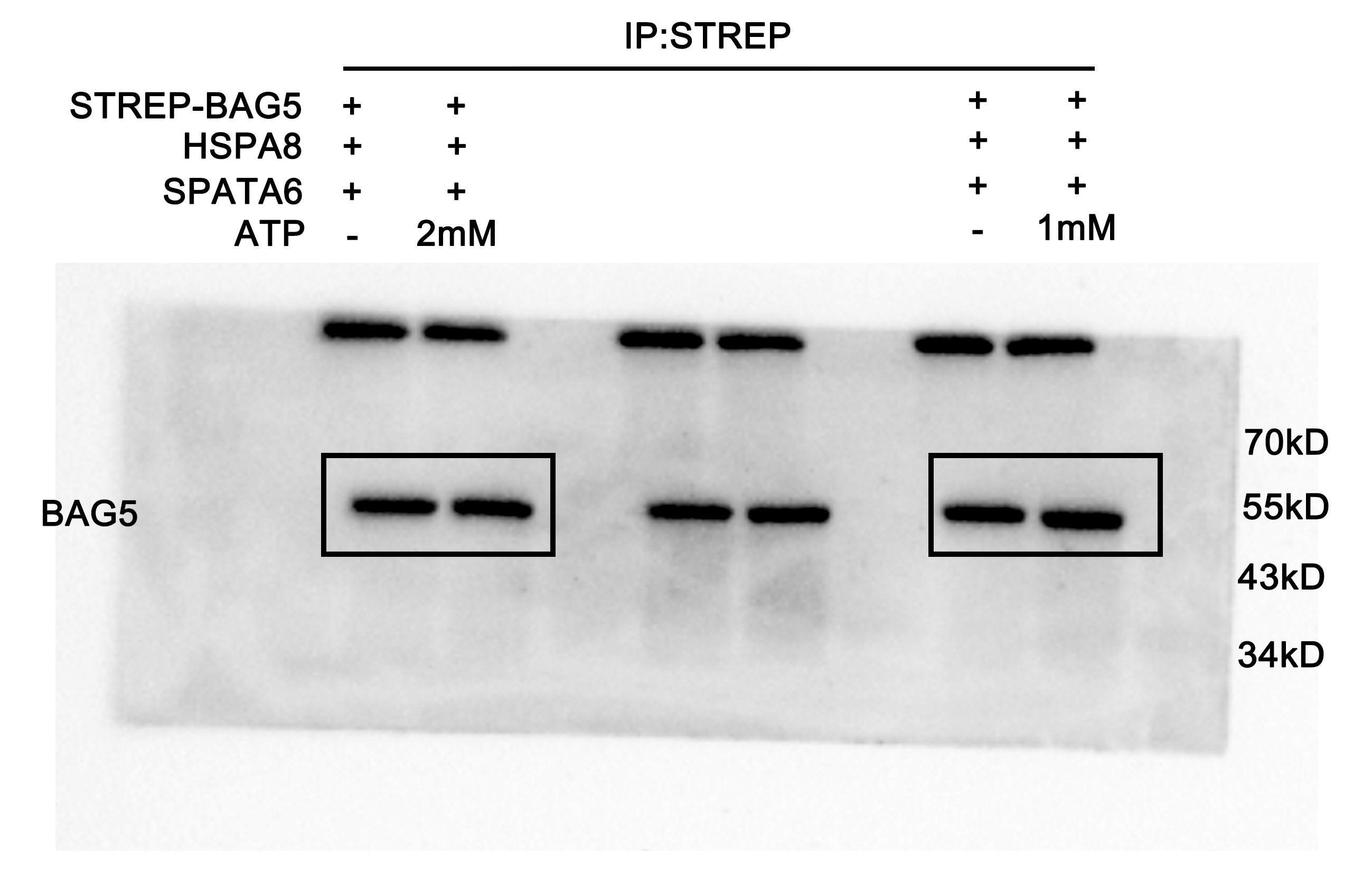

Supplement: Supplementary file 19 — Source Data Fig. 8 [file 44319_2024_112_MOESM19_ESM.zip › Figure 8/Figure 8/8J/WB BAG5.tif]

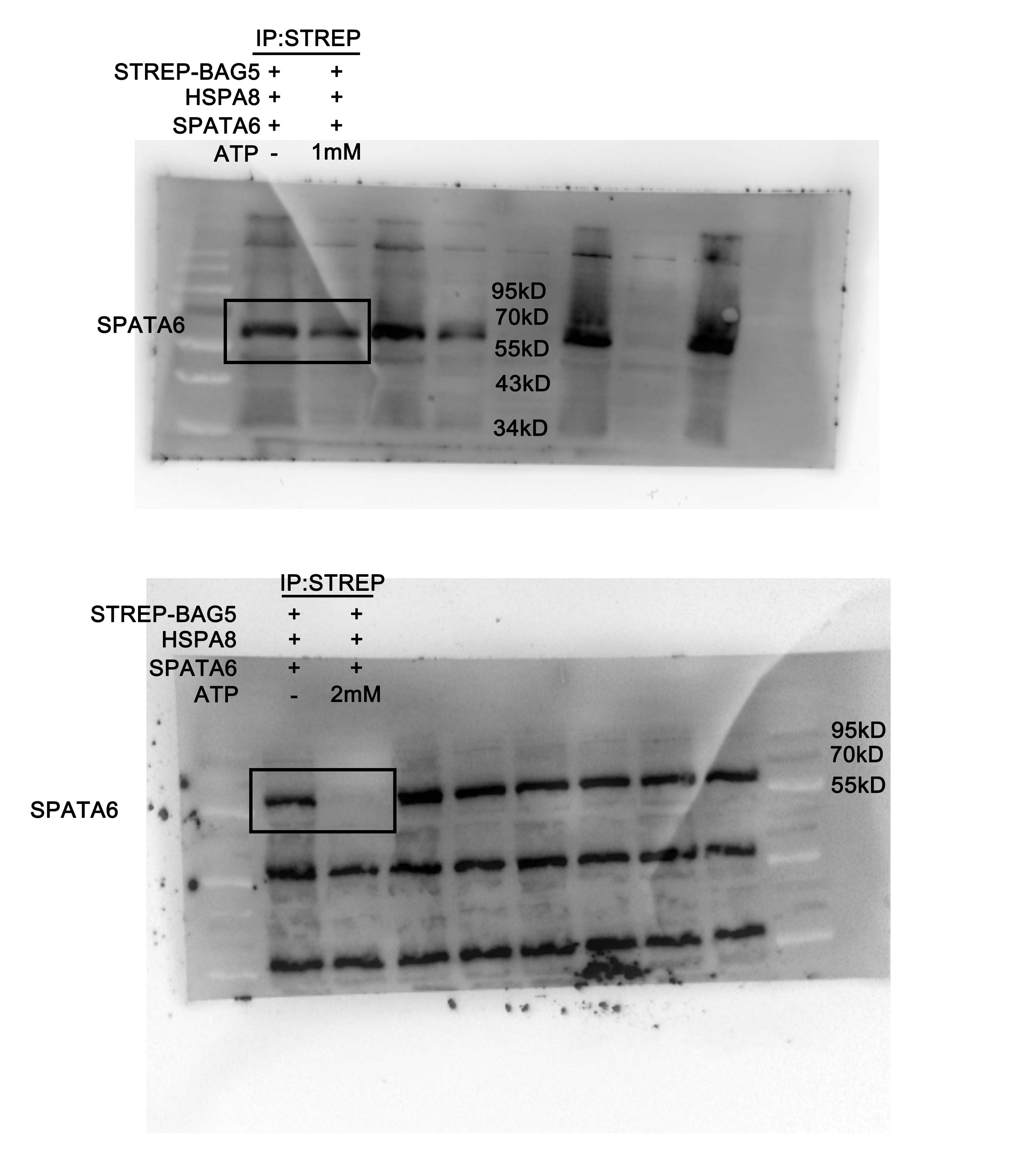

Supplement: Supplementary file 19 — Source Data Fig. 8 [file 44319_2024_112_MOESM19_ESM.zip › Figure 8/Figure 8/8J/WB SPATA6.tif]
