# Supplementary material for: BAG5 regulates HSPA8-mediated protein folding required for sperm head-tail coupling apparatus assembly
Source: EMBO Rep. 2024 Mar 7;25(4):23. doi: 10.1038/s44319-024-00112-x (PMC11015022; doi:10.1038/s44319-024-00112-x)
Supplement: Supplementary file 21 — Expanded View Figures [file 44319_2024_112_MOESM21_ESM.pdf]

Expanded View Figures

Figure EV1. BAG5 interacts with SAPTA6 and is expressed in testes with high conservation among nine vertebrate species.

(**A**) A list of representative SPATA6-interacting partners identified from adult WT mouse testes. (**B**) qRT-PCR analyses of *Bag5* mRNA levels in various mouse tissues. *Bag5* is highly expressed in mouse testes. Data were presented as mean ± SEM. (**C**) Representative western blot shows the expression pattern of BAG5 in various mouse tissues. GAPDH served as the loading control. (**D**) Representative microscopy images of BAG5 (red) and α-TUB (green) immunodetection from adult WT mice are shown on stage IX–X (left) and stage IV–VI (right) seminiferous tubules. Nuclei were stained with DAPI (white). Scale bars = 25 μm. (**E**) Phylogenetic tree of BAG5 orthologs among nine vertebrate species. (**F**) Amino acid sequence similarities among nine vertebrate species. Data information: Data in (**B**–**D**) represent results from three independent biological replicate experiments. Source data are available online for this figure.

 © The Author(s)

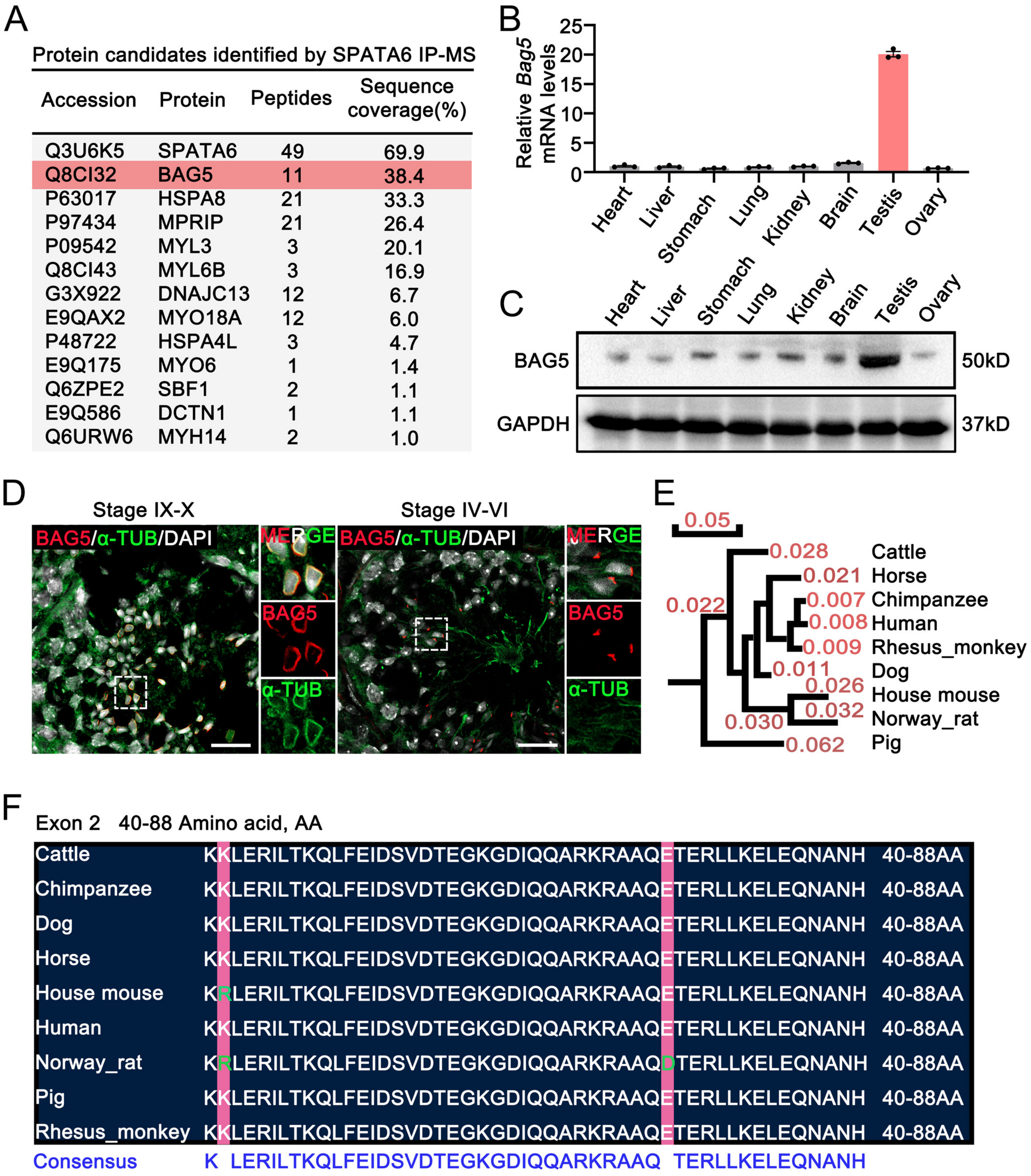

Accession	Protein	Peptides	Sequence coverage(%)
Q3U6K5	SPATA6	49	69.9
Q8CI32	BAG5	11	38.4
P63017	HSPA8	21	33.3
P97434	MPRIP	21	26.4
P09542	MYL3	3	20.1
Q8CI43	MYL6B	3	16.9
G3X922	DNAJC13	12	6.7
E9QAX2	MYO18A	12	6.0
P48722	HSPA4L	3	4.7
E9Q175	MYO6	1	1.4
Q6ZPE2	SBF1	2	1.1
E9Q586	DCTN1	1	1.1
Q6URW6	MYH14	2	1.0

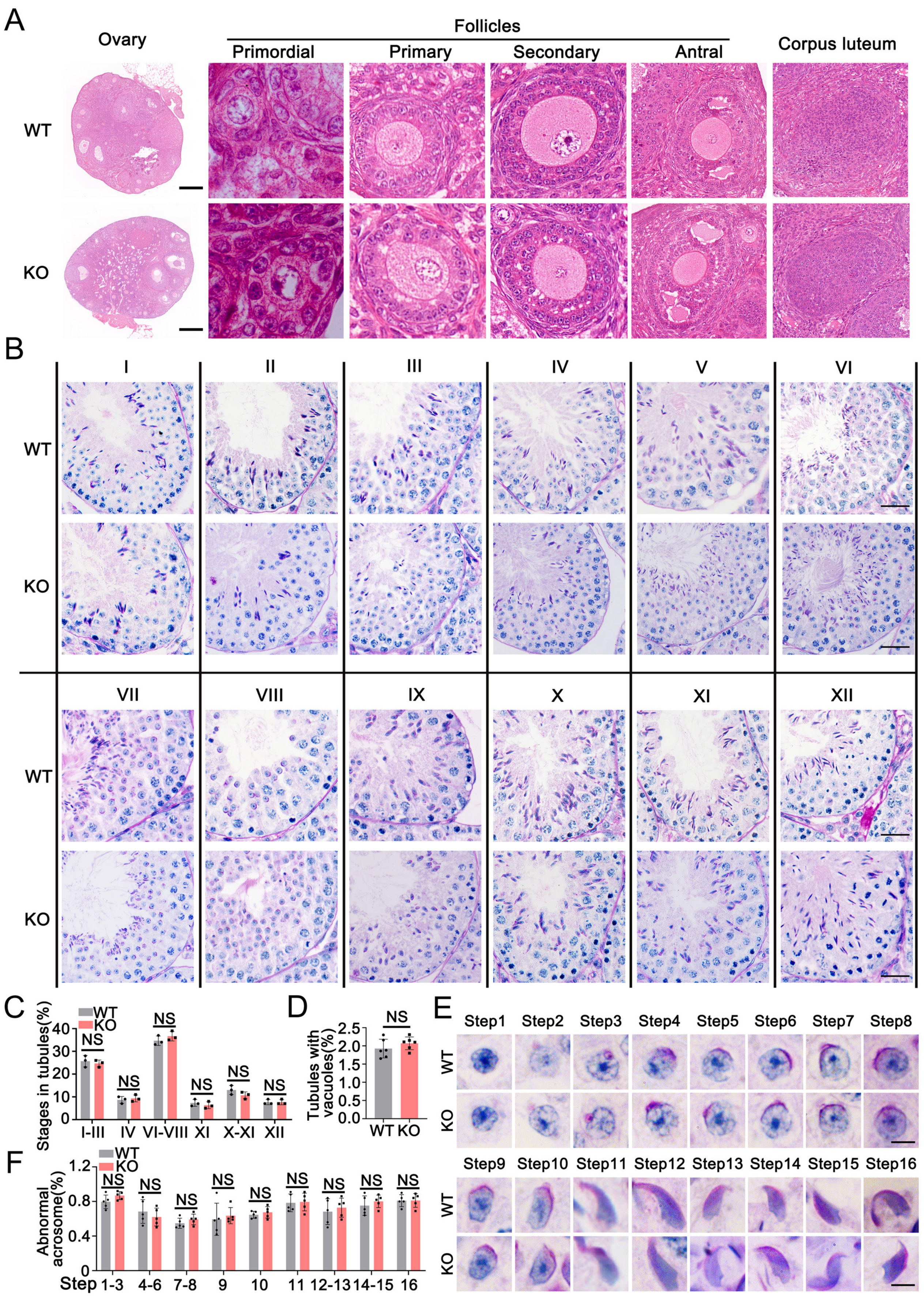
A
Ovary
Follicles
Primordial
Primary
Secondary
Antral
Corpus luteum
WT
KO
B
I
II
III
IV
V
VI
VII
VIII
IX
X
XI
XII
WT
KO
C
WT
KO
Stages in tubules(%)
NS
I-III
IV
VI-VIII
XI
X-XI
XII
D
Tubules with vacuoles(%)
NS
WT KO
E
Step1 Step2 Step3 Step4 Step5 Step6 Step7 Step8
Step9 Step10 Step11 Step12 Step13 Step14 Step15 Step16
WT
KO
F
WT
KO
Abnormal acrosome(%)
NS
Step 1-3 4-6 7-8 9 10 11 12-13 14-15 16

    © The Author(s)

Figure EV2. Histological analyses of ovaries and testes in WT and KO mice.

(**A**) HE staining of WT and KO ovaries. The development of primordial follicle, primary follicle, secondary follicle, and antral follicle was normal in KO ovaries. Scale bars = 400 μm. (**B**) PAS-staining of WT and KO mouse testes. The development of seminiferous epithelia was divided into 12 stages. Scale bars = 50 μm. (**C**) Quantifying the percentage of seminiferous tubules from WT and KO mice at different stages is shown. Data were presented as mean ± SD. NS not significant. *P* values (Student *t*-test, two-sided). (**D**) Quantifying the percentage of seminiferous tubules with vacuoles in WT and KO mice is shown. Data were presented as mean ± SD. NS not significant. *P* values (Student *t*-test, two-sided). (**E**) Representative images of sperm morphology at different steps of spermatids in WT and KO mice are shown. Scale bars = 5 μm. (**F**) Quantifying the ratio of different steps of spermatids with abnormal acrosome in WT and KO mice is shown. Data were presented as mean ± SD. NS not significant. *P* values (Student *t*-test, two-sided). Data information: Data in (**A–C**) represent results from three independent biological replicate experiments. Data in (**D**) represent results from six independent replicate experiments (two technical and three biological replicates). Data in (**E**, **F**) represent results from five independent biological replicate experiments. Source data are available online for this figure.

© The Author(s)

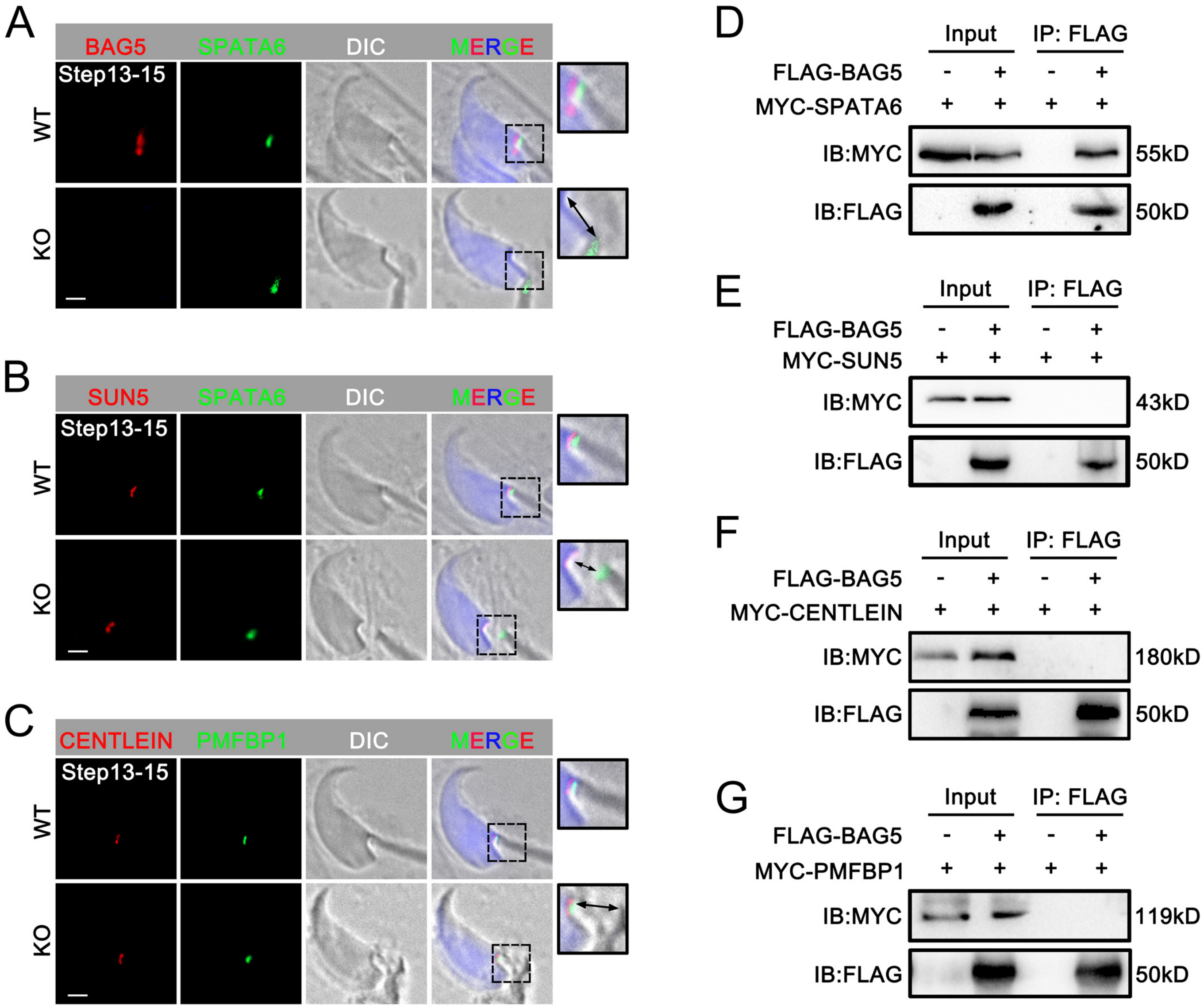

Figure EV3. The analyses of acephalic spermatozoa-related protein expression in WT and KO mice.

(**A**) Representative immunofluorescence images of BAG5 (red) and SPATA6 (green) in steps 13–15 of spermatids from WT and KO mice are shown. Nuclei were stained with DAPI (blue). The double arrow represents the separation of sperm head and tail. Scale bar = 2 μm. (**B**) Representative immunofluorescence images of SUN5 (red) and SPATA6 (green) in steps 13–15 of spermatids from WT and KO mice are shown. Nuclei were stained with DAPI (blue). The double arrow represents the separation of sperm head and tail. Scale bar = 2 μm. (**C**) Representative immunofluorescence images of CENTLEIN (red) and PMFBP1 (green) in steps 13–15 of spermatids from WT and KO mice are shown. Nuclei were stained with DAPI (blue). The double arrow represents the separation of sperm head and tail. Scale bar = 2 μm. (**D–G**) In vitro Co-IP assays to examine the interaction between FLAG-BAG5 and MYC-SPATA6 (**D**), MYC-SUN5 (**E**), MYC-CENTLENIN (**F**), and MYC-PMFBP1 (**G**). Data information: Data in (**A–G**) represent results from three independent biological replicate experiments. Source data are available online for this figure.

 © The Author(s)

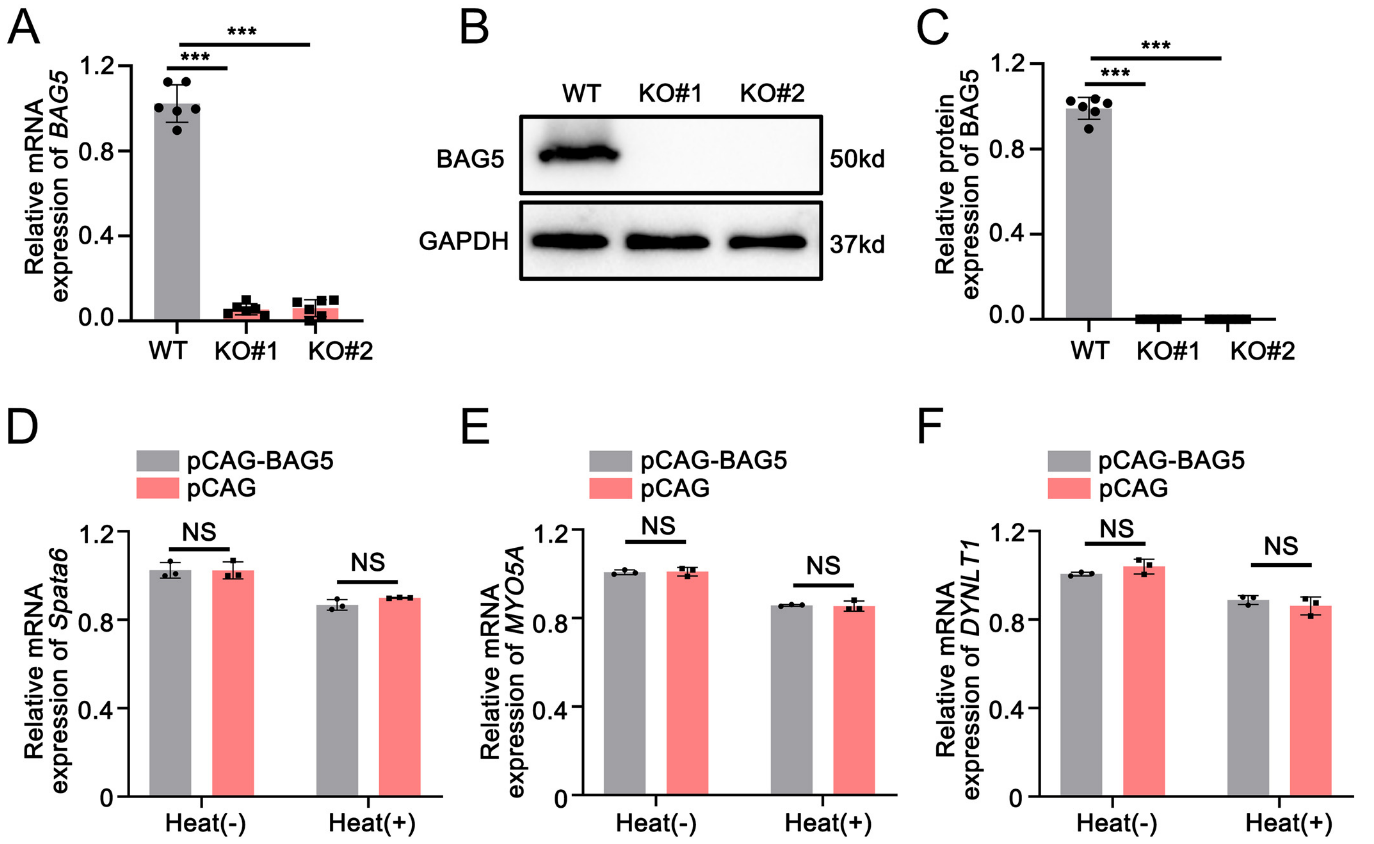

Figure EV4. Establishment of *BAG5* KO cell line.

(**A**) Histogram shows the relative *BAG5* mRNA expression level in WT and two *BAG5* KO HeLa cell lines. Data were presented as mean ± SD. *P* values (Student *t*-test, two-sided). ****P* < 0.001. (**B**) The representative images of the protein expression of BAG5 in WT and two *BAG5*-KO HeLa cell lines are shown. (**C**) The BAG5 protein expression level is quantified in WT and two *BAG5* KO HeLa cell lines in (**B**). Data were presented as mean ± SD. *P* values (Student *t*-test, two-sided). ****P* < 0.001. (**D–F**) Histograms showing the mRNA expression level of genes (*Spata6, Myo5a*, and *Dynlt1*) in Fig. 5E. NS not significant. Data were presented as mean ± SD. *P* values (Student *t*-test, two-sided). Data information: Data in (**A–C**) represent results from six independent experiments (two technical and three biological replicates). Data in (**D–F**) represent results from three independent biological replicate experiments. Source data are available online for this figure.

© The Author(s)

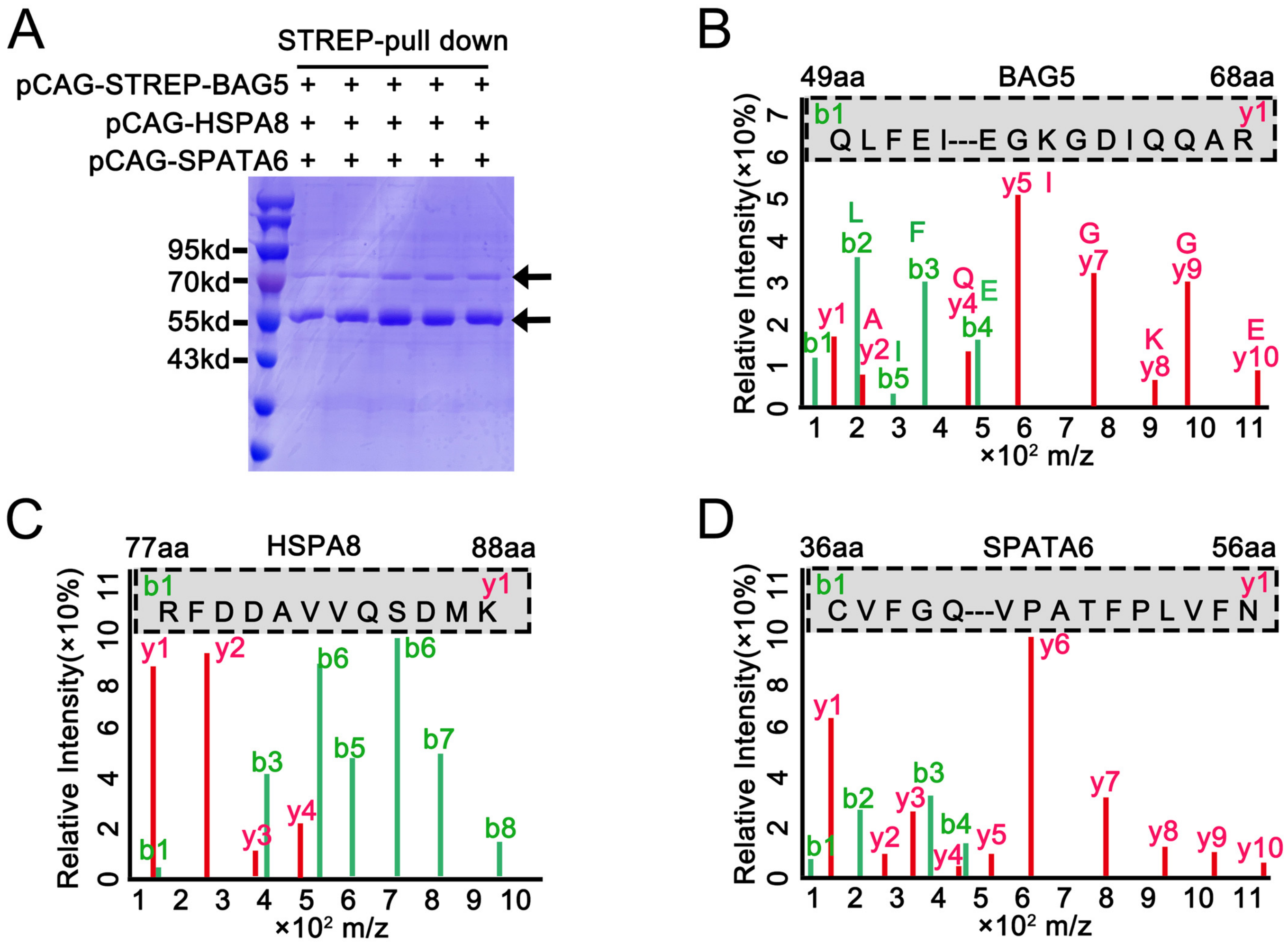

E

Mass spectrometric analysis of complex

Uniprot Acc.No.	Name	#Peptides	#PSMs	MW[kDa]	Score
Q8CI32	Bag5	27	89	50.9	323.31
P63017	Hspa8	23	36	70.9	295.49
Q3U6K5	Spata6	12	19	55.1	264.13

Figure EV5. Analyses of the BAG5-HSPA8-SPATA6 protein complex.

(**A**) The representative images of Coomassie brilliant blue staining from Strep-tag affinity chromatography purified protein complexes are shown after co-transfected with pCAG-STREP-BAG5, pCAG-HSPA8, and pCAG-SPATA6 plasmids. The arrows indicated the protein molecular weights of 70 kd and 55 kd. STREP-BAG5 indicated protein molecular weight 55 KD, STREP 5 KD plus BAG5 50 KD. (**B–D**) Representative MS spectra for specific peptides of BAG5 (**B**), HSPA8 (**C**), and SPATA6 (**D**) proteins. (**E**) The table shows the proteins detected by mass spectrometry of STREP-pull-down complexes. BAG5 protein molecular weight was 50 KD in the UniProt database. Data information: Data in (**A**) represent results from three independent biological replicates experiments. Source data are available online for this figure.

 © The Author(s)
