# Supplementary material for: BAG5 regulates HSPA8-mediated protein folding required for sperm head-tail coupling apparatus assembly
Source: EMBO Rep. 2024 Mar 7;25(4):23. doi: 10.1038/s44319-024-00112-x (PMC11015022; doi:10.1038/s44319-024-00112-x)
Supplement: Supplementary file 20 — Peer Review File [file 44319_2024_112_MOESM20_ESM.pdf]

EMBO reports

BAG5 regulates HSPA8-mediated protein folding required for sperm head-tail coupling apparatus assembly

Shiming Gan, Shumin Zhou, Jinzhe Ma, Mengneng Xiong, Wenjing Xiong, Xu Fan, Kuan Liu, Yiqian Gui, Bei Chen, Beibei Zhang, Xiaoli Wang, Fengli Wang, Zhean Li, Wei Yan, Meisheng Ma, and Shuiqiao Yuan

Corresponding author(s): Shiming Gan (Shiminggan@hust.edu.cn)

Review Timeline:

Submission Date:	7th Aug 23
Editorial Decision:	4th Oct 23
Revision Received:	27th Nov 23
Editorial Decision:	23rd Jan 24
Revision Received:	1st Feb 24
Accepted:	22nd Feb 24

Editor: Martina Rembold

Transaction Report:

(Note: With the exception of the correction of typographical or spelling errors that could be a source of ambiguity, letters and reports are not edited. Depending on transfer agreements, referee reports obtained elsewhere may or may not be included in this compilation. Referee reports are anonymous unless the Referee chooses to sign their reports.)

Dear Dr. Yuan

Thank you for the submission of your research manuscript to our journal. I apologize for the delay in handling your manuscript, but we have now received the two referee reports that are copied below.

As you will see, the referees acknowledge that the findings are potentially interesting, but they also raise a number of concerns that need to be addressed.

Given the constructive comments and support from the referees, we would like to invite you to revise your manuscript with the understanding that the referee concerns (as detailed above and in their reports) must be fully addressed and their suggestions taken on board. Please address all referee concerns in a complete point-by-point response. Acceptance of the manuscript will depend on a positive outcome of a second round of review. It is EMBO Reports policy to allow a single round of revision only and acceptance or rejection of the manuscript will therefore depend on the completeness of your responses included in the next, final version of the manuscript.

We realize that it is difficult to revise to a specific deadline. In the interest of protecting the conceptual advance provided by the work, we recommend a revision within 3 months (January 1st). Please discuss the revision progress ahead of this time with the editor if you require more time to complete the revisions.

I am also happy to discuss the revision further via e-mail or a video call, if you wish.

*****IMPORTANT NOTE:
We perform an initial quality control of all revised manuscripts before re-review. Your manuscript will FAIL this control and the handling will be DELAYED if the following APPLIES:

1) A data availability section providing access to data deposited in public databases is missing. If you have not deposited any data, please add a sentence to the data availability section that explains that.

2) Your manuscript contains statistics and error bars based on n=2. Please use scatter blots in these cases. No statistics should be calculated if n=2.

When submitting your revised manuscript, please carefully review the instructions that follow below. Failure to include requested items will delay the evaluation of your revision.********

When submitting your revised manuscript, we will require:

1) a .docx formatted version of the manuscript text (including legends for main figures, EV figures and tables). Please make sure that the changes are highlighted to be clearly visible.

2) individual production quality figure files as .eps, .tif, .jpg (one file per figure).
Please download our Figure Preparation Guidelines (figure preparation pdf) from our Author Guidelines pages https://www.embopress.org/page/journal/14693178/authorguide for more info on how to prepare your figures.

3) a .docx formatted letter INCLUDING the reviewers' reports and your detailed point-by-point responses to their comments. As part of the EMBO Press transparent editorial process, the point-by-point response is part of the Review Process File (RPF), which will be published alongside your paper.

4) a complete author checklist, which you can download from our author guidelines (<https://www.embopress.org/page/journal/14693178/authorguide>). Please insert information in the checklist that is also reflected in the manuscript. The completed author checklist will also be part of the RPF.

5) Please note that all corresponding authors are required to supply an ORCID ID for their name upon submission of a revised manuscript (<https://orcid.org/>). Please find instructions on how to link your ORCID ID to your account in our manuscript tracking system in our Author guidelines (<https://www.embopress.org/page/journal/14693178/authorguide#authorshipguidelines>)

6) We replaced Supplementary Information with Expanded View (EV) Figures and Tables that are collapsible/expandable online. A maximum of 5 EV Figures can be typeset. EV Figures should be cited as 'Figure EV1, Figure EV2" etc... in the text and their respective legends should be included in the main text after the legends of regular figures.

- For the figures that you do NOT wish to display as Expanded View figures, they should be bundled together with their legends in a single PDF file called *Appendix*, which should start with a short Table of Content. Appendix figures should be referred to in the main text as: "Appendix Figure S1, Appendix Figure S2" etc. See detailed instructions regarding expanded view here: <https://www.embopress.org/page/journal/14693178/authorguide#expandedview>

- Additional Tables/Datasets should be labeled and referred to as Table EV1, Dataset EV1, etc. Legends have to be provided in a separate tab in case of .xls files. Alternatively, the legend can be supplied as a separate text file (README) and zipped together with the Table/Dataset file.

7) Before submitting your revision, primary datasets (and computer code, where appropriate) produced in this study need to be deposited in an appropriate public database (see < https://www.embopress.org/page/journal/14693178/authorguide#dataavailability>).

Specifically, we would kindly ask you to provide public access to the proteomics dataset.

Please remember to provide a reviewer password if the datasets are not yet public.

The accession numbers and database should be listed in a formal "Data Availability " section (placed after Materials & Method) that follows the model below (see also < https://www.embopress.org/page/journal/14693178/authorguide#dataavailability>). Please note that the Data Availability Section is restricted to new primary data that are part of this study.

Data availability

The datasets (and computer code) produced in this study are available in the following databases:

- RNA-Seq data: Gene Expression Omnibus GSE46843 (https://www.ncbi.nlm.nih.gov/geo/query/acc.cgi?acc=GSE46843)
- [data type]: [name of the resource] [accession number/identifier/doi] ([URL or identifiers.org/DATABASE:ACCESSION])

*** Note - All links should resolve to a page where the data can be accessed. ***

8) At EMBO Press we ask authors to provide source data for the main figures. Our source data coordinator will contact you to discuss which figure panels we would need source data for and will also provide you with helpful tips on how to upload and organize the files.

Additional information on source data and instruction on how to label the files are available <https://www.embopress.org/page/journal/14693178/authorguide#sourcedata>.

9) The journal requires a statement specifying whether or not authors have competing interests (defined as all potential or actual interests that could be perceived to influence the presentation or interpretation of an article). In case of competing interests, this must be specified in your disclosure statement. Further information: https://www.embopress.org/competing-interests

10) Figure legends and data quantification:
The following points must be specified in each figure legend:

- the name of the statistical test used to generate error bars and P values,
- the number (n) of independent experiments (please specify technical or biological replicates) underlying each data point,
- the nature of the bars and error bars (s.d., s.e.m.)

- If the data are obtained from n {less than or equal to} 5, show the individual data points in addition to the SD or SEM.
- If the data are obtained from n {less than or equal to} 2, use scatter blots showing the individual data points.

Discussion of statistical methodology can be reported in the materials and methods section, but figure legends should contain a basic description of n, P and the test applied.

See also the guidelines for figure legend preparation:
https://www.embopress.org/page/journal/14693178/authorguide#figureformat

- Please also include scale bars in all microscopy images and define their size in the legend, not the image.

11) Our journal encourages inclusion of *data citations in the reference list* to directly cite datasets that were re-used and obtained from public databases. Data citations in the article text are distinct from normal bibliographical citations and should directly link to the database records from which the data can be accessed. In the main text, data citations are formatted as follows: "Data ref: Smith et al, 2001" or "Data ref: NCBI Sequence Read Archive PRJNA342805, 2017". In the Reference list,

data citations must be labeled with "[DATASET]". A data reference must provide the database name, accession number/identifiers and a resolvable link to the landing page from which the data can be accessed at the end of the reference. Further instructions are available at <https://www.embopress.org/page/journal/14693178/authorguide#referencesformat>.

12) As part of the EMBO publication's Transparent Editorial Process, EMBO Reports publishes online a Review Process File to accompany accepted manuscripts. This File will be published in conjunction with your paper and will include the referee reports, your point-by-point response and all pertinent correspondence relating to the manuscript.

You are able to opt out of this by letting the editorial office know (emboreports@embo.org). If you do opt out, the Review Process File link will point to the following statement: "No Review Process File is available with this article, as the authors have chosen not to make the review process public in this case."

We would also welcome the submission of cover suggestions, or motifs to be used by our Graphics Illustrator in designing a cover.

I look forward to seeing a revised form of your manuscript when it is ready and please let me know if you have questions or comments regarding the revision.

Yours sincerely,

Martina Rembold, PhD
Senior Editor
EMBO reports

****

Referee #1:

In this study the authors investigate the chaperone requirements for connecting piece assembly and the relationship between BAG5, HSPAs and SPATA6. The authors establish a fertility defect for BAG5 mice and reveal mechanistic insight for molecular function that will also be valuable for understanding the role of BAG5 in other tissues, such as the heart (cardiac myopathy). The paper builds on previous work showing that disrupting SPATA6, a protein required for connecting piece assembly, can lead to acephalic spermatozoa.

The immunoprecipitation, mass spectrometry and validation approach shown in Fig 1 is well explained, and the fertility data of the BAG5 knockout mice are compelling.

I am not convinced by the data investigating colocalization of BAG5 with ER chaperones (in Fig 1 H-J and pg 7). The staining data is not sufficiently high resolution to discriminate between the ER and cytosol (and other organelles). Whilst it is possible that BAG5 is recruited to the cytosolic face of the ER, further evidence is required to demonstrate genuine import into the ER lumen in the absence of a targeting signal. HEK293 cells will also lack some of the specialist machinery of spermatids. The text on pg 7 should be revised to moderate the conclusions.

Similarly, I am not convinced by the HSPA8 co-localisation data and explanation (Fig 7E and pg 15). According to multiple papers, HSPA8 is cytosolically localised. The interpretation of the data therefore requires moderating. One HSPA8 role is in chaperone mediated autophagy to promote import of substrates into lysosomes. Have the authors considered that HSPA8 may be participating in the development of the acrosome, where some ER chaperones can accumulate?

Pg 11: The authors state that "Together, these data hint that BAG5 interacts with SPATA6 to regulate the attachment of Cp and Sc to HTCA during spermiogenesis." However, there are other possibilities and primary data is lacking in full support of this assertion. For example, BAG5 could be working indirectly on unidentified clients in spermatids via the Hsp70 cycle, or could be influencing proteostasis through its role as a ubiquitin system modulator.

On page 13, why were HeLa cells chosen for the BAG5 cell line knockout? A myocyte (muscle) cell line might have been better to identify more physiologically/disease relevant protein interaction network.

The interaction experiments in the presence of ATP (pg 17 and Fig 8) support the notion that the NBD domain of HSPA8 is required for the phenotype, but a more thorough experiment would be to include non-hydrolysable analogs and to employ a nucleotide binding domain mutant.

Minor point: there are some spelling mistakes and grammar issues. The paper would benefit from additional editing for English language.

Referee #2:

Review comments

The authors discovered that BAG5 complexes are essential for the head-tail coupling apparatus. They found that BAG5 regulates the chaperone protein HSPA8 and its loss causes abnormal protein misfolding. The present paper is a very excellent in the view of revealing BAG5 is essential for male fertility and also the molecular mechanisms underlying the development of the head-tail coupling apparatus. In addition, there is no major problem with the main experimental results or story, so I think it should be published in an EMBO report. However, the authors may be mistaken about cytoplasmic behavior in spermiogenesis. Due to this, I cannot ignore the schematic diagram in Fig 4E, movie EV3 and 4, as they might spread false information. In addition, discussion is very long and boring as it was just a list of contents from the past literature. Discussion should be systematically focused on information directly related to this literature. An excellent set of data is ruined by a boring discussion.

Major concerns
The diagram in Fig 4E, movies EV3 and EV4 must be modified. I have no complaints about head, acrosome, HTCA, and flagellar behavior. But the behavior of cytoplasm is totally strange. To my knowledge, I only know the following literature that systematically describes cytoplasmic behavior in spermatids. The authors should read page 20-27 in the book. Diagrams and movies lack views of cytoplasmic invagination, nuclear movement toward the cell surface, cytoplasmic lobes, and residual bodies. This paper could be read by many people, but I cannot ignore the possibility of spreading incorrect views, even schematic models.
Russell, L., Ettlin, R., Sinha Hikim, A., and Clegg, E. (1990). Histological and histopathological evaluation of the testis. 1990 (Cache River Press).

As mentioned in the preamble, the discussion is too long with a lot of listing of past literature contents. Please put yourself in the reader's shoes and make the contents more informative.

Minor concerns
It is difficult to see blue on a black background. I think the blue color should be changed to another color. Figs. 1C, 1J, 4A and 7F are particularly difficult to see.

The pictures in Fig. 1E are too small. In contrast, Fig. 1F is too big and not so important. In addition, Fig. 1E contains data from KO mice even though they will be generated later. To demonstrate the significant data clearly, Fig 1E should be enlarged to show the localization of BAG5 in basal plate. This should be performed even if KO and 1F pictures are removed. In addition, steps 9 and 14 spermatids are too different in form. Therefore, the steps for spermatids should be the same.

Endpieces in Fig. 3H are cross-sections of principal piece because they have fibrous sheath. In my experience, it is very difficult to find an endpiece with a clear axonemal structure in TEM. The endpiece of this figure should be removed.

All KO spermatids are oriented abnormally in Fig 4A (right-most) schematic. However, this figure may be misleading because less than 15% of all sperm are oriented abnormally. In addition, the author has not clarified experimentally the orientation of the sperm flagellum. Since the sperm flagellum orientation may be normal, the flagellum orientation should also be examined if this schematic is included.

Although this is a matter of personal interest, I suspect that cytoplasmic invagination does not occur in KO spermatids when looking at TEM images in Fig. 4C. As observed in WT spermatids, the tail end of centrosome should be close to cell surface. This is caused by cytoplasmic invagination. Since no cytoplasmic surface is seen at the posterior end of HTCA in KO spermatozoa, there must be an abnormality in cytoplasmic invagination.

What is "M" in Fig. 8H? There is no explanation (maybe manchette).
ER can be observed outside the manchette in Fig. 8H. Do you have any evidence for it?

POINT-BY-POINT RESPONSE TO REVIEWERS

Our specific comments to each reviewer follow. Please note: Reviewer comments are in *italics*. Our responses are in blue font. The comments from the Reviewers have not been edited. We thank all Reviewers' comments, which enabled us to significantly improve the manuscript and highlight the importance of our study.

REVIEWER COMMENTS

Referee #1:
In this study the authors investigate the chaperone requirements for connecting piece assembly and the relationship between BAG5, HSPAs and SPATA6. The authors establish a fertility defect for BAG5 mice and reveal mechanistic insight for molecular function that will also be valuable for understanding the role of BAG5 in other tissues, such as the heart (cardiac myopathy). The paper builds on previous work showing that disrupting SPATA6, a protein required for connecting piece assembly, can lead to acephalic spermatozoa.
The immunoprecipitation, mass spectrometry and validation approach shown in Fig 1 is well explained, and the fertility data of the BAG5 knockout mice are compelling.

Thank you very much for your evaluation and valuable comments, which enabled us to improve the quality of the manuscript.

I am not convinced by the data investigating colocalization of BAG5 with ER chaperones (in Fig 1 H-J and pg 7). The staining data is not sufficiently high resolution to discriminate between the ER and cytosol (and other organelles). Whilst it is possible that BAG5 is recruited to the cytosolic face of the ER, further evidence is required to demonstrate genuine import into the ER lumen in the absence of a targeting signal. HEK293 cells will also lack some of the specialist machinery of spermatids. The text on pg 7 should be revised to moderate the conclusions.

Thank you for these helpful comments. To provide more conclusive evidence that BAG5 is located in the ER, we extracted the ER proteins from the mouse testes using the commercial Kit. The new results showed that BAG5 was indeed localized in the ER. We have shown these results as a new **Figure 1I** and revised the text accordingly (**See lines 162-164**). We have also revised the conclusions based on the additional experimental results (**See lines 164-167** with yellow color highlighted).

Similarly, I am not convinced by the HSPA8 co-localisation data and explanation (Fig 7E and pg 15). According to multiple papers, HSPA8 is cytosolically localised. The interpretation of the data therefore requires moderating.

Thanks for pointing out this issue. We agreed with the notion that HSPA8 is cytosolically localized. We have repeated the immunostaining experiments for HSPA8 and BAG5

localization in HEK293 cells and testicular sections and found that HSPA8 was indeed colocalized with BAG5 in the cytoplasm of HEK293 cells and the manchette of elongating spermatids, respectively (See new **Figure 7E** and **lines 363-365** with yellow color highlighted).

One HSPA8 role is in chaperone mediated autophagy to promote import of substrates into lysosomes. Have the authors considered that HSPA8 may be participating in the development of the acrosome, where some ER chaperones can accumulate?

Thank you for your constructive thoughts. As we observed in **Figure EV2E-F** of this study, the acrosome developmental process was normal after the knockout of BAG5, which indicates that HSPA8-mediated protein folding activated by BAG5 was not associated with acrosome development.

Pg 11: The authors state that "Together, these data hint that BAG5 interacts with SPATA6 to regulate the attachment of Cp and Sc to HTCA during spermiogenesis." However, there are other possibilities and primary data is lacking in full support of this assertion. For example, BAG5 could be working indirectly on unidentified clients in spermatids via the Hsp70 cycle, or could be influencing proteostasis through its role as a ubiquitin system modulator.

Thanks for pointing out this issue. We agree with the other possible roles of BAG5 in spermiogenesis raised by this Reviewer, and it provided us with a future direction to investigate the underlying molecular regulation of BAG5 on spermatid development in a separate study. To clarify the statement in the current study, we have revised the text description in **lines 279-280** accordingly.

On page 13, why were HeLa cells chosen for the BAG5 cell line knockout? A myocyte (muscle) cell line might have been better to identify more physiologically/disease relevant protein interaction network.

Thank you for raising this question. We also agree that the myocyte (muscle) cell line may be better for studying physiologically/disease-relevant protein interaction networks. However, the current study mainly focuses on the function of BAG5 in the reproductive system; therefore, we chose the reproductive system cell lines (such as HeLa) to verify the protein interaction *in vitro*.

The interaction experiments in the presence of ATP (pg 17 and Fig 8) support the notion that the NBD domain of HSPA8 is required for the phenotype, but a more thorough experiment would be to include non-hydrolysable analogs and to employ a nucleotide binding domain mutant.

Thank you for your careful review and great suggestions. As suggested, we performed the additional experiments and showed the new data in **Figure 8F-I**. The new results support the point again that the NBD domain of HSPA8 is required for the phenotype. We have also

added the elaboration into text in **lines 394-399** with yellow color highlighted**.**

Minor point: there are some spelling mistakes and grammar issues. The paper would benefit from additional editing for English language.

The manuscript has been checked and polished by a professional editing service. All typos and grammar issues have been corrected as far as possible. Thank you so much for this careful review.

Referee #2:

Review comments

The authors discovered that BAG5 complexes are essential for the head-tail coupling apparatus. They found that BAG5 regulates the chaperone protein HSPA8 and its loss causes abnormal protein misfolding. The present paper is a very excellent in the view of revealing BAG5 is essential for male fertility and also the molecular mechanisms underlying the development of the head-tail coupling apparatus. In addition, there is no major problem with the main experimental results or story, so I think it should be published in an EMBO report.

Thank you very much for your appreciation of our work.

However, the authors may be mistaken about cytoplasmic behavior in spermiogenesis. Due to this, I cannot ignore the schematic diagram in Fig 4E, movie EV3 and 4, as they might spread false information.

We apologize for this mistake and appreciate that you have raised this critical comment. We have redrawn the schematic to show the cytoplasmic invagination and nuclear movement towards the cell surface in steps 6-8, and the formation and removal of cytoplasmic lobes to produce residual bodies in WT steps 15-16 of the new **Figure 4E**. Since it is difficult to visualize the developmental complex process of spermatids in spermiogenesis by 3D movie, we decided to remove the movies EV3-4 in the revision to avoid any misunderstanding for the readers. Thanks for your careful review.

In addition, discussion is very long and boring as it was just a list of contents from the past literature. Discussion should be systematically focused on information directly related to this literature. An excellent set of data is ruined by a boring discussion.

We agree with this suggestion and have rewritten the discussion section in the revision (**See lines 427-470, 494-506, and 514-525** with yellow color highlighted). Thank you!

Major concerns

The diagram in Fig 4E, movies EV3 and EV4 must be modified. I have no complaints about head, acrosome, HTCA, and flagellar behavior. But the behavior of cytoplasm is totally

strange. To my knowledge, I only know the following literature that systematically describes cytoplasmic behavior in spermatids. The authors should read page 20-27 in the book. Diagrams and movies lack views of cytoplasmic invagination, nuclear movement toward the cell surface, cytoplasmic lobes, and residual bodies. This paper could be read by many people, but I cannot ignore the possibility of spreading incorrect views, even schematic models.
Russell, L., Ettlin, R., Sinha Hikim, A., and Clegg, E. (1990). Histological and histopathological evaluation of the testis. 1990 (Cache River Press).

Thank you for your comments and helpful guidance. We have read the recommended book carefully and gained a lot of knowledge regarding the process of spermiogenesis, especially on cytoplasmic invagination. We have corrected the schematic models in the new **Figure 4E**, including the cytoplasmic invagination, nuclear movement towards the cell surface, cytoplasmic lobes, and residual bodies.

As mentioned in the preamble, the discussion is too long with a lot of listing of past literature contents. Please put yourself in the reader's shoes and make the contents more informative.

Thank you for your suggestion. We have simplified the discussion to enhance its readability (**See lines 427-470, 494-506, and 514-525**).

Minor concerns
It is difficult to see blue on a black background. I think the blue color should be changed to another color. Figs. 1C, 1J, 4A and 7F are particularly difficult to see.

Thank you for that excellent suggestion. As suggested, we changed the blue color to white color in **Figures 1C, 1J (new 1H), 4A, and 7F (new 7E)** and made the nucleus visible.

The pictures in Fig. 1E are too small. In contrast, Fig. 1F is too big and not so important. In addition, Fig. 1E contains data from KO mice even though they will be generated later. To demonstrate the significant data clearly, Fig 1E should be enlarged to show the localization of BAG5 in basal plate. This should be performed even if KO and 1F pictures are removed. In addition, steps 9 and 14 spermatids are too different in form. Therefore, the steps for spermatids should be the same.

This is a good suggestion. We have enlarged **Figure 1E** to show the clear location of BAG5 in the Manchette and Basal plate, and we moved the KO data and **Figure 1F** to **Appendix Figures S1 and S2**.

Endpieces in Fig. 3H are cross-sections of principal piece because they have fibrous sheath. In my experience, it is very difficult to find an endpiece with a clear axonemal structure in TEM. The endpiece of this figure should be removed.

Thank you for your professional advice. We took the image of the endpiece carefully again

and showed it in **Figure 3H**.

All KO spermatids are oriented abnormally in Fig 4A (right-most) schematic. However, this figure may be misleading because less than 15% of all sperm are oriented abnormally. In addition, the author has not clarified experimentally the orientation of the sperm flagellum. Since the sperm flagellum orientation may be normal, the flagellum orientation should also be examined if this schematic is included.

Thank you for your thoughtful comments. We have re-examined the orientation of the flagellum by co-immunostaining of AKAP4 (labels the fibrous sheath located in the principal piece) with ATP5A (marks the mitochondria located in the midpiece) and have shown the data in **Appendix Figure S3**. The new results showed that the midpiece (ATP5A, red) towards the basal membrane and the principal piece (AKAP4, green) close to the lumen, indicating that the orientation of the sperm flagellum was normal, as this Reviewer thought. We have, therefore, corrected the schematic diagram in **Figure 4A** (right-most) and revised the text accordingly (**See lines 243-244** with yellow color highlighted).

Although this is a matter of personal interest, I suspect that cytoplasmic invagination does not occur in KO spermatids when looking at TEM images in Fig. 4C. As observed in WT spermatids, the tail end of centrosome should be close to cell surface. This is caused by cytoplasmic invagination. Since no cytoplasmic surface is seen at the posterior end of HTCA in KO spermatozoa, there must be an abnormality in cytoplasmic invagination.

Thank you for raising this interesting question. We agree with you and have drawn the schematic model to show the abnormal cytoplasmic invagination in KO spermatids in **Figure 4E**. We have also added this assumption in the revised text (**See lines 256-261** with yellow color highlighted).

What is "M" in Fig. 8H? There is no explanation (maybe manchette).

Thank you for your careful review. "M" is "manchette". We have added the explanation in the legend of **Figure 8H**.

ER can be observed outside the manchette in Fig. 8H. Do you have any evidence for it?

Thank you for raising this critical question. To ensure we provided the correct information for the readers, we reviewed the literature on the formation of germ cell organelles during spermiogenesis. Based on the published literature, ER undergoes marked changes and structural modifications during spermiogenesis (Clermont *et al*, 1993; Hermo *et al*, 2010; Toshimori *et al*, 2001). At the acrosome phase, the ER aligns along the inside and outside of manchette microtubules in the form of a fenestrated sleeve (Clermont *et al.*, 1993; Clermont & Rambourg, 1978; Hermo *et al.*, 2010; Kaneko *et al*, 2019; Nistal *et al*, 1980). Thus, we drew the schematic diagram in **Figure 8H** to show the abundant location of the ER close to the manchette.

REFERENCES:

Clermont Y, Oko R, Louis Harkey H, Clermont Y, Hermo L (1993) Cell biology of mammalian spermiogenesis. *Cell & Molecular Biology of the Testis*

Clermont Y, Rambourg A (1978) Evolution of the endoplasmic reticulum during rat spermiogenesis. *Am J Anat* 151: 191-211

Hermo L, Pelletier RM, Cyr DG, Smith CE (2010) Surfing the wave, cycle, life history, and genes/proteins expressed by testicular germ cells. Part 2: changes in spermatid organelles associated with development of spermatozoa. *Microsc Res Tech* 73: 279-319

Kaneko T, Minohara T, Shima S, Yoshida K, Fukuda A, Iwamori N, Inai T, Iida H (2019) A membrane protein, TMCO5A, has a close relationship with manchette microtubules in rat spermatids during spermiogenesis. *Mol Reprod Dev* 86: 330-341

Nistal M, Paniagua R, Esponda P (1980) Development of the endoplasmic reticulum during human spermatogenesis. *Acta Anat (Basel)* 108: 238-249

Toshimori K, Yoshinaga K, Tanii I, Wakayama T, Saxena DK, Ohoka T (2001) Protein expression and cell organelle behavior in spermatogenic cells. *Kaibogaku Zasshi* 76: 267-279

1st Revision - Editorial Decision 23rd Jan 2024

Dear Dr. Yuan

Thank you for the submission of your revised manuscript to EMBO reports. We have now received the full set of referee reports that is copied below.

As you will see, both referees conclude that the revision has strengthened the manuscript. However, referee 2 has some remaining concerns that I kindly ask you to address. I am also happy to discuss these experiments further with you.

From the editorial side, there are also a few things that we need before we can proceed with the official acceptance of your study.

- Please update the 'Conflict of interest' paragraph to our new 'Disclosure and competing interests statement'. For more information see
https://www.embopress.org/page/journal/14693178/authorguide#conflictsofinterest

-The author Xu Fan is listed as Xu Fan in the manuscript file but as Xv Fan in the submission system. Please correct either or.

- Please remove the Author Contributions from the manuscript file and make sure that the author contributions in our online submission system are correct and up-to-date. The information you specified in the system will be automatically retrieved and typeset into the article. You can enter additional information in the free text box provided, if you wish.

- All information on funding needs to be part of Acknowledgement section and this information must match that in the submission system. The Fundamental Research Funds for the Central Universities (YCJJ20230239, 2022JYCXJJ048) and Basic Research Support Program of Huazhong University of Science and Technology (2023BR031) are missing in the submission system.

-The callout for Movie EV2 should be corrected: it should be "Movie EV1 and EV2" instead of "Movie EV1 and 2".

- The movie legends need to be removed from the manuscript file and provided as a readme.txt file and then each should be zipped together with its corresponding video.

- Table EV1-EV5 are complex tables, please rename them to Dataset EV1-EV5 and correct the callouts in the manuscript. Please add a legend to the tab "Proteins" in Dataset EV1. Table EV6 would then be Table EV1 and Table EV7 becomes Table EV2.

- The Data Availability section should only refer to datasets deposited in an external repository. Therefore, please remove the statement "The authors declare that all other original data in this article are available from the corresponding author upon reasonable request."

- Mouse experiments: please provide the reference number for approval of the mouse work.

- The following sentence should be removed from the manuscript: "Expanded View Figures and Appendix File Table of Contents for this article are available online."

- The title should be removed from p45, (before the EV figure legends).

-We perform a routine figure check on all manuscripts before publication. In this context we noticed the following issues:

A) Figure 2J - The image shown for the KO seems to be the same image as the WT, however rotated. This also applies to the source data images. Please check these images. Also, the cropped image does not fit with the bounding box for the WT.

B) It seems that some sections shown in Figure 4A have been used again in Figure EV2B (WT IX, KO IV). If this is the case, please clearly state this in the figure legend.

C) Figure EV2 B KO an WT iii images look very similar to each other. Please check these.

- Source data need to be uploaded one folder per figure for main figures and can be grouped together for EV and Appendix.

- Our production/data editors have asked you to clarify several points in the figure legends (see below). Please incorporate these changes in the manuscript and return the revised file with tracked changes with your final manuscript submission.

a) Please indicate the statistical test used for data analysis in the legends of figures 5a-b; 6a-b; 7a-b.
b) Please note that information related to n is missing in the legend of figure 5a.

c) Please note that the arrows are not defined in the legend of figure EV 5a. This needs to be rectified.

- As a standard procedure, we edit the title and abstract of manuscripts to make them more accessible to a general readership. Please find the edited versions below my signature and incorporate these in the manuscript if you agree with any of the changes.

- Finally, EMBO Reports papers are accompanied online by A) a short (1-2 sentences) summary of the findings and their significance, B) 2-3 bullet points highlighting key results and C) a synopsis image that is 550x300-600 pixels large (width x height) in PNG for JPG format. You can either show a model or key data in the synopsis image. Please note that the size is rather small and that text needs to be readable at the final size. Please send us this information along with the revised manuscript.

- On a different note, I would like to alert you that EMBO Press offers a new format for a video-synopsis of work published with us, which essentially is a short, author-generated film explaining the core findings in hand drawings, and, as we believe, can be very useful to increase visibility of the work. This has proven to offer a nice opportunity for exposure i.p. for the first author(s) of the study. Please see the following link for representative examples and their integration into the article web page:
https://www.embopress.org/video_synopses
https://www.embopress.org/doi/full/10.15252/embj.2019103932

Please let me know, should you be interested to engage in commissioning a similar video synopsis for your work. According operation instructions are available and intuitive.

We look forward to seeing a final version of your manuscript as soon as possible.

Yours sincerely,

Martina Rembold, PhD
Senior Editor
EMBO reports

****

Suggested abstract:

Teratozoospermia is a significant cause of male infertility, but the pathogenic mechanism of acephalic spermatozoa syndrome (ASS), one of the most severe teratozoospermia, remains elusive. We previously reported Spermatogenesis Associated 6 (SPATA6) as the component of the sperm head-tail coupling apparatus (HTCA) required for normal assembly of the sperm head-tail conjunction, but the underlying molecular mechanism has not been explored. Here, we find that the co-chaperone protein BAG5, expressed in step 9-16 spermatids, is essential for sperm HTCA assembly. BAG5-deficient male mice show abnormal assembly of HTCA, leading to ASS and male infertility, phenocopying SPATA6-deficient mice. In vivo and in vitro experiments demonstrate that SPATA6, cargo transport-related myosin proteins (MYO5A and MYL6) and dynein proteins (DYNLT1, DCTN1, and DNAL1) are misfolded upon BAG5 depletion. Mechanistically, we find that BAG5 forms a complex with HSPA8 and promotes the folding of SPATA6 by enhancing HSPA8's affinity for substrate proteins. Collectively, our findings reveal a novel protein-regulated network in sperm formation in which BAG5 governs the assembly of the HTCA by activating the protein-folding function of HSPA8.

****

Referee #1:

The authors have responded carefully to the points raised in review and have provided substantial new data, including a more detailed analysis of HSPA8 and the role of the NBD; and a more convincing localisation analysis of BAG5 and HSPA8. I have no further comments for the authors to address.

Referee #2:

Major points
#1
The authors mentioned that KO spermatids have abnormal cytoplasmic invagination (L265). When I observed Fig. 4C, it looks abnormal. But cytoplasmic invagination could be found in KO spermatids (Fig. S1). I think this means there are no abnormalities

in this process. You can check this process in the following paper. I recommend that you re-evaluate this process.
"CCDC183 is essential for cytoplasmic invagination around the flagellum during spermiogenesis and male fertility" by Shimada et al.
My new concerns are that TEM pictures of HTCA in KO spermatids (Fig. 4C) are unclear. I believe Fig. 4C is very critical, but the quality of the enlarged pictures is poor. That's why misunderstandings like the above can happen. I understand the difficulty of observing HTCA structure, but you should work on it until you get suitable pictures. In particular, the HTCA of KO in steps 15-16 is terrible. I think "Dc" becomes an axoneme (centrioles have triplet microtubules without central microtubules) and "Sc" becomes an annulus. I recommend changing the pictures of KO HTCA in step11-12, 13-14, 15-16.
Related to this concern, Fig. 4E should be modified again. In addition, the cytoplasm of WT sperm in step 13-14, 15-16, sperm and KO sperm in step 13-14, 15-16, sperm is strange. Cytoplasm present below the annulus should be removed (Cytoplasm is present around the flagellum, but not in such large amounts). Also, I'm still uncomfortable because the axonemes are too short. All axonemes or flagellum should extend to the edge of the figures.

#2
The authors mentioned that KO sperm have abnormal mitochondrial sheath arrangements (L499). Fig. 3H shows a cross-section of the midpiece, principal piece and endpiece. But "KO midpiece" appears to be the principal piece. Is this really a midpiece? Furthermore, both endpiece figures still depict the principal piece. You should check the following paper which describes the endpiece features. The endpiece should not have accessory structures. In addition, its legend states that all scale bars are the same size. If so, the midpiece is too small and the endpiece is too large. Given these considerations, the TEM data in this paper cannot be trusted.

I understand the difficulty of finding an endpiece, and I know that many papers incorrectly refer to a midpiece as an endpiece. In my opinion it would be very difficult to count the 9+2 structure of the end piece until a statistical analysis could be done. Based on these results, I also believe Figure 3I has a problem. My suggestion is that it would not make sense to analyze the flagellum separately into three parts, so the abnormal axonemal data in Fig 3I should be combined (midpiece + principal piece + endpiece). In addition, Figure 3H should be changed. When it is difficult to find an endpiece, only the cross-section of the principal piece is sufficient. Since mitochondrial sheaths are so slim in spermatozoa (Fig. 3B and 3E), I also think there might be no mitochondrial sheath in KO spermatozoa. For this reason, mitochondrial staining of spermatozoa is necessary to reveal abnormal mitochondrial sheath arrangements to state L499.
"Axonemal doublet microtubules can split into two complete singlets in human sperm flagellum tips" by Zabeo et al.

Minor points
L145: Manchette is only observed in spermatids. "manchette during spermiogenesis and basal plate of spermatozoa" might be better.

L213, 221, Fig. 3B, 3E: A cytoplasmic droplet (CD)-like structure is used by the authors to explain abnormal phenotypes observed in spermatozoa. The cytoplasmic droplet is a normal morphological structure in WT sperm, however. "Excess residual cytoplasm" might be suitable for this abnormality. You should check the following paper.
"An investigation of excess residual cytoplasm in human spermatozoa and its distinction from the cytoplasmic droplet" by Rengan et al.

There is no steps or stages in Fig. 1E and S1. This information is necessary for these figures.

Fig. 2D: β-ACTIN seems strange. You should use ACTB or β-actin.

Fig. 2G figure includes vas deferens. This information should be included in the figure legend.

Fig. 8D, F, H, J: The authors use "IP:STREP". But I think immunoprecipitation was not performed in these studies. In addition, STREP-IP was used in the figure legend.

Fig. 8L: BAG5 does not localize to the basal plate in spermatids with manchettes structure. BAG5 can be localized on the basal plate only during late spermatids and mature spermatozoa (Fig. 1D, 1E, 1F). Therefore, the pink dots (BAG5) on the basal plate in the "Assemble" picture should be removed. Instead, they should be placed on the basal plate in the "Normal spermatozoa" picture.

BAG5 changes their location from manchette to basal plate during spermiogenesis (Fig. 1D, 1E). The authors should mention about that in discussion part.

RESPONSE TO REVIEWER COMMENTS AND EDITORIAL REQUIREMENTS

REVIEWER COMMENTS

Referee #1:
The authors have responded carefully to the points raised in review and have provided substantial new data, including a more detailed analysis of HSPA8 and the role of the NBD; and a more convincing localisation analysis of BAG5 and HSPA8. I have no further comments for the authors to address.
RESPONSE: Thank you very much for your appreciation of this revised manuscript.

Referee #2:
Major points
#1
The authors mentioned that KO spermatids have abnormal cytoplasmic invagination (L265). When I observed Fig. 4C, it looks abnormal. But cytoplasmic invagination could be found in KO spermatids (Fig. S1). I think this means there are no abnormalities in this process. You can check this process in the following paper. I recommend that you re-evaluate this process. "CCDC183 is essential for cytoplasmic invagination around the flagellum during spermiogenesis and male fertility" by Shimada et al.
RESPONSE: Thank you for your professional guidance in helping us to improve our knowledge of cytoplasmic invagination. As you suggested, we re-evaluated the process of cytoplasmic invagination in our *Bag5* KO mouse spermatids and found that ~63% of *Bag5* KO spermatids showed abnormal cytoplasmic invagination by quantification of TEM images (see **Appendix Figure S4** and lines 305-307). To avoid any inappropriate description of cytoplasmic invagination, we have also read two papers (PMID: 37882665 and 36881620) on the cytoplasmic invagination process published by Dr Masahito Ikawa's group (the first author is Keisuke Shimada) and have cited them in the Discussion section of this revision (see lines 536-542).

My new concerns are that TEM pictures of HTCA in KO spermatids (Fig. 4C) are unclear. I believe Fig. 4C is very critical, but the quality of the enlarged pictures is poor. That's why misunderstandings like the above can happen. I understand the difficulty of observing HTCA structure, but you should work on it until you get suitable pictures. In particular, the HTCA of KO in steps 15-16 is terrible. I think "Dc" becomes an axoneme (centrioles have triplet microtubules without central microtubules) and "Sc" becomes an annulus. I recommend changing the pictures of KO HTCA in step11-12, 13-14, 15-16.
RESPONSE: Thank you for your careful review and good suggestions. As you suggested, we have retaken the TEM images and carefully re-evaluated the abnormal HTCA in Bag5 KO. In this revision, we have replaced the representative image of KO HTCA in steps 11-12, 13-14, and 15-16 in **Figure 4C** and provided new original data. We sincerely hope you will find these new images satisfactory and suitable for publication.

Related to this concern, Fig. 4E should be modified again. In addition, the cytoplasm of WT sperm in step 13-14, 15-16, sperm and KO sperm in step 13-14, 15-16, sperm is strange. Cytoplasm present below the annulus should be removed (Cytoplasm is present around the flagellum, but not in such large amounts). Also, I'm still uncomfortable because the axonemes are too short. All axonemes or flagellum should extend to the edge of the figures.
RESPONSE: Thank you for your helpful comments. Following your suggestion, we removed the cytoplasm below the annulus in **Figure 4E** (steps 13-14, 15-16, spermatozoa in WT and KO) and extended all axonemes or flagellum to the edge in **Figure 4E**.

#2
The authors mentioned that KO sperm have abnormal mitochondrial sheath arrangements (L499). Fig. 3H shows a cross-section of the midpiece, principal piece and endpiece. But "KO midpiece" appears to be the principal piece. Is this really a midpiece?
RESPONSE: Thank you for your careful review. In this revision, we performed an immunofluorescent experiment using ATP5A, a marker of mitochondria, to assess the mitochondrial sheath arrangements in *Bag5* KO spermatozoa. We found that ATP5A was located in excess residual cytoplasm in KO spermatozoa, whereas the ATP5A-positive signal distribution was in the midpiece of WT spermatozoa (see new **Figure 3J**). This new result further confirmed that the mitochondrial sheath was lost in the midpiece of *Bag5* KO spermatozoa. We have thus replaced the image of KO midpiece with new one in **Figure 3H** and also revised the description in lines 261-270.

Furthermore, both endpiece figures still depict the principal piece. You should check the following paper which describes the endpiece features. The endpiece should not have accessory structures.
RESPONSE: Thank you for helping us with the correction. As you suggested, we have reviewed the paper (PMID: 30959570) and learned a lot from it. Therefore, in the new **Figure 3H**, we have removed the pictures of the endpiece with the incorrect features and shown only images of the middle and principal pieces.

In addition, its legend states that all scale bars are the same size. If so, the midpiece is too small and the endpiece is too large. Given these considerations, the TEM data in this paper cannot be trusted.
RESPONSE: Thank you for pointing out this issue. We have re-examined the original TEM images and corrected the scale bars in **Figure 3H**.

I understand the difficulty of finding an endpiece, and I know that many papers incorrectly refer to a midpiece as an endpiece. In my opinion it would be very difficult to count the 9+2 structure of the end piece until a statistical analysis could be done. Based on these results, I also believe Figure 3I has a problem. My suggestion is that it would not make sense to analyze the flagellum separately into three parts, so the abnormal axonemal data in Fig 3I should be combined (midpiece + principal piece + endpiece).
RESPONSE: We agree with you and thank you for your constructive suggestion. In the revision, we have only performed the statistical analysis of the combined abnormal sperm

axonemal flagella with midpiece + principal pieces between WT and KO mice (see **new Figure 3I**).

In addition, Figure 3H should be changed. When it is difficult to find an endpiece, only the cross-section of the principal piece is sufficient. Since mitochondrial sheaths are so slim in spermatozoa (Fig. 3B and 3E), I also think there might be no mitochondrial sheath in KO spermatozoa. For this reason, mitochondrial staining of spermatozoa is necessary to reveal abnormal mitochondrial sheath arrangements to state L499.
"Axonemal doublet microtubules can split into two complete singlets in human sperm flagellum tips" by Zabeo et al.
RESPONSE: As responded to above, we have removed the endpiece and replaced the KO midpiece in **Figure 3H** to make the description more accurate. We also performed an immunofluorescence experiment using ATP5A, a marker for mitochondria, to assess the arrangement of the mitochondrial sheath in *Bag5* KO spermatozoa and confirmed that the mitochondrial sheath is absent in the midpiece of *Bag5* KO spermatozoa. We have added the corresponding statements in lines 263-270 of the revised manuscript.

Minor points
L145: Manchette is only observed in spermatids. "manchette during spermiogenesis and basal plate of spermatozoa" might be better.
RESPONSE: We agree with it and have revised the description as suggested (see lines 178-180).

L213, 221, Fig. 3B, 3E: A cytoplasmic droplet (CD)-like structure is used by the authors to explain abnormal phenotypes observed in spermatozoa. The cytoplasmic droplet is a normal morphological structure in WT sperm, however. "Excess residual cytoplasm" might be suitable for this abnormality. You should check the following paper.
"An investigation of excess residual cytoplasm in human spermatozoa and its distinction from the cytoplasmic droplet" by Rengan et al.
RESPONSE: Agreed! We have read the article carefully and revised Figures 3B, E and the text accordingly (see lines 246, 252, 254).

There is no steps or stages in Fig. 1E and S1. This information is necessary for these figures.
RESPONSE: Thank you for your careful review. We have added the steps in **Figure 1E** and **Appendix Figure S1**.

Fig. 2D: β-ACTIN seems strange. You should use ACTB or β-actin.
RESPONSE: Done as suggested.Thanks!

Fig. 2G figure includes vas deferens. This information should be included in the figure legend.
RESPONSE: The 'vas deferens' information was added in the figure legend as suggested. Thanks again!

Fig. 8D, F, H, J: The authors use "IP: STREP". But I think immunoprecipitation was not performed in these studies. In addition, STREP-IP was used in the figure legend.
RESPONSE: We now used "STREP-Pull down" to replace the "IP: STREP" to make the description more accurate in the revised Figure 8D, F, H, and J. We also changed the figure legend accordingly.

Fig. 8L: BAG5 does not localize to the basal plate in spermatids with manchettes structure. BAG5 can be localized on the basal plate only during late spermatids and mature spermatozoa (Fig. 1D, 1E, 1F). Therefore, the pink dots (BAG5) on the basal plate in the "Assemble" picture should be removed. Instead, they should be placed on the basal plate in the "Normal spermatozoa" picture.
RESPONSE: This is a great suggestion. It was done as suggested. Thanks!

BAG5 changes their location from manchette to basal plate during spermiogenesis (Fig. 1D, 1E). The authors should mention about that in discussion part.
RESPONSE: Done as suggested (see lines 572-577). Thank you very much for your constructive suggestions and diligent efforts during the last and this round review process.

EDITORIAL REQUEST
From the editorial side, there are also a few things that we need before we can proceed with the official acceptance of your study.
- Please update the 'Conflict of interest' paragraph to our new 'Disclosure and competing interests statement'. For more information see https://www.embopress.org/page/journal/14693178/authorguide#conflictsofinterest
RESPONSE: We have updated it as required.

-The author Xu Fan is listed as Xu Fan in the manuscript file but as Xv Fan in the submission system. Please correct either or.
RESPONSE: Corrected!

- Please remove the Author Contributions from the manuscript file and make sure that the author contributions in our online submission system are correct and up-to-date. The information you specified in the system will be automatically retrieved and typeset into the article. You can enter additional information in the free text box provided, if you wish.
RESPONSE: We have removed the Author Contribution section from the manuscript.

- All information on funding needs to be part of Acknowledgement section and this information must match that in the submission system. The Fundamental Research Funds for the Central Universities (YCJJ20230239, 2022JYCXJJ048) and Basic Research Support Program of Huazhong University of Science and Technology (2023BR031) are missing in the submission system.
RESPONSE: All funding information has been added to the revised manuscript.

-The callout for Movie EV2 should be corrected: it should be "Movie EV1 and EV2" instead of "Movie EV1 and 2".
RESPONSE: Corrected!

- The movie legends need to be removed from the manuscript file and provided as a readme.txt file and then each should be zipped together with its corresponding video.
RESPONSE: Done as required.

- Table EV1-EV5 are complex tables, please rename them to Dataset EV1-EV5 and correct the callouts in the manuscript. Please add a legend to the tab "Proteins" in Dataset EV1. Table EV6 would then be Table EV1 and Table EV7 becomes Table EV2.
RESPONSE: Done as required.

- The Data Availability section should only refer to datasets deposited in an external repository. Therefore, please remove the statement "The authors declare that all other original data in this article are available from the corresponding author upon reasonable request."
RESPONSE: Done as required.

- Mouse experiments: please provide the reference number for approval of the mouse work.
RESPONSE: We have provided the reference number (S2795) for approval of the mouse work.

- The following sentence should be removed from the manuscript: "Expanded View Figures and Appendix File Table of Contents for this article are available online."
RESPONSE: Done as required.

- The title should be removed from p45, (before the EV figure legends).
RESPONSE: Done as required.

-We perform a routine figure check on all manuscripts before publication. In this context we noticed the following issues:
A) Figure 2J - The image shown for the KO seems to be the same image as the WT, however rotated. This also applies to the source data images. Please check these images. Also, the cropped image does not fit with the bounding box for the WT.
RESPONSE: Thank you for the careful checking. We have replaced the KO image with a new one and provided new raw data.

B) It seems that some sections shown in Figure 4A have been used again in Figure EV2B (WT IX, KO IV). If this is the case, please clearly state this in the figure legend.
RESPONSE: We have corrected **Figure 4A** (WT IX, KO IV) to ensure that there are no similar parts to **Figure EV2B** and provided the original data in the revision.

C) Figure EV2 B KO an WT iii images look very similar to each other. Please check these.

RESPONSE: We have replaced the **Figure EV2B** KO III image with a new one and provided the original data.

- Source data need to be uploaded one folder per figure for main figures and can be grouped together for EV and Appendix.
RESPONSE: Done as required.

- Our production/data editors have asked you to clarify several points in the figure legends (see below). Please incorporate these changes in the manuscript and return the revised file with tracked changes with your final manuscript submission.
a) Please indicate the statistical test used for data analysis in the legends of figures 5a-b; 6a-b; 7a-b.
RESPONSE: As required, We added a statistical test method in the legends of Figures 5A-B, 6A-B, and 7A-B.

b) Please note that information related to n is missing in the legend of figure 5a.
RESPONSE: Done as required.

c) Please note that the arrows are not defined in the legend of figure EV 5a. This needs to be rectified.
RESPONSE: We have pointed out the definition of arrows in the legend of Figure EV 5A.

- As a standard procedure, we edit the title and abstract of manuscripts to make them more accessible to a general readership. Please find the edited versions below my signature and incorporate these in the manuscript if you agree with any of the changes.
RESPONSE: We agree with these changes and incorporate the new abstract in the revised manuscript. Thank you very much for your work.

- Finally, EMBO Reports papers are accompanied online by A) a short (1-2 sentences) summary of the findings and their significance, B) 2-3 bullet points highlighting key results and C) a synopsis image that is 550x300-600 pixels large (width x height) in PNG for JPG format. You can either show a model or key data in the synopsis image. Please note that the size is rather small and that text needs to be readable at the final size. Please send us this information along with the revised manuscript.
RESPONSE: The synopsis image is provided in the Figure folder and named ‘synopsis’.

- On a different note, I would like to alert you that EMBO Press offers a new format for a video-synopsis of work published with us, which essentially is a short, author-generated film explaining the core findings in hand drawings, and, as we believe, can be very useful to increase visibility of the work. This has proven to offer a nice opportunity for exposure i.p. for the first author(s) of the study. Please see the following link for representative examples and their integration into the article web page:
https://www.embopress.org/video_synopses
https://www.embopress.org/doi/full/10.15252/embj.2019103932

Please let me know, should you be interested to engage in commissioning a similar video synopsis for your work. According operation instructions are available and intuitive.
RESPONSE: Thank you for giving us this opportunity, but we are not interested in engaging in doing it.

We look forward to seeing a final version of your manuscript as soon as possible.

Yours sincerely,

Martina Rembold, PhD
Senior Editor
EMBO reports

****
Suggested abstract:

Teratozoospermia is a significant cause of male infertility, but the pathogenic mechanism of acephalic spermatozoa syndrome (ASS), one of the most severe teratozoospermia, remains elusive. We previously reported Spermatogenesis Associated 6 (SPATA6) as the component of the sperm head-tail coupling apparatus (HTCA) required for normal assembly of the sperm head-tail conjunction, but the underlying molecular mechanism has not been explored. Here, we find that the co-chaperone protein BAG5, expressed in step 9-16 spermatids, is essential for sperm HTCA assembly. BAG5-deficient male mice show abnormal assembly of HTCA, leading to ASS and male infertility, phenocopying SPATA6-deficient mice. In vivo and in vitro experiments demonstrate that SPATA6, cargo transport-related myosin proteins (MYO5A and MYL6) and dynein proteins (DYNLT1, DCTN1, and DNAL1) are misfolded upon BAG5 depletion. Mechanistically, we find that BAG5 forms a complex with HSPA8 and promotes the folding of SPATA6 by enhancing HSPA8's affinity for substrate proteins. Collectively, our findings reveal a novel protein-regulated network in sperm formation in which BAG5 governs the assembly of the HTCA by activating the protein-folding function of HSPA8.
RESPONSE: Great abstract. We agree with this abstract and use it in the revised manuscript. Thanks again.

Dr. Shiming Gan
Huazhong University of Science and Technology
Institute of Reproductive Health
No.13 Hangkong Road
Wuhan, Hubei 430030
China

Dear Dr. Gan,

Thank you for the submission of your revised manuscript. The revision and new experiments were evaluated once more by referee #2 who is now all positive and supports publication.

I am now very pleased to accept your manuscript for publication in the next available issue of EMBO reports. Thank you for your contribution to our journal.

Your manuscript will be processed for publication by EMBO Press. It will be copy edited and you will receive page proofs prior to publication. Please note that you will be contacted by Springer Nature Author Services to complete licensing and payment information.

You may qualify for financial assistance for your publication charges - either via a Springer Nature fully open access agreement or an EMBO initiative. Check your eligibility: https://www.embopress.org/page/journal/14693178/authorguide#chargesguide

Should you be planning a Press Release on your article, please get in contact with embo_production@springernature.com as early as possible in order to coordinate publication and release dates.

If you have any questions, please do not hesitate to contact the Editorial Office. Thank you for your contribution to EMBO Reports.

Yours sincerely,

Martina Rembold, PhD
Senior Editor
EMBO reports

Referee #2:

The authors revised the paper adequately. I have no claim for this paper other than Fig. 4C. TEM pictures for steps 15-16 look like another step for both WT and KO. During steps 15-16, the annulus of spermatids moves toward the border of both the midpiece and principal piece. However, it is localized around the HTCA in these pictures. In addition, a mitochondrial sheath should be formed in these steps. You should change the pictures in steps 15-16 to the correct ones.
Other points were very well done. I think this paper must be published in a journal. It was a pleasure to review the paper. Thank you.

>>> Please note that it is EMBO Reports policy for the transcript of the editorial process (containing referee reports and your response letter) to be published as an online supplement to each paper. If you do NOT want this, you will need to inform the Editorial Office via email immediately. More information is available here: https://www.embopress.org/transparent-process#Review_Process

EMBO Press Author Checklist

Corresponding Author Name: Shuiqiao Yuan	
Journal Submitted to: EMBO Reports	
Manuscript Number: EMBOR-2023-57948	

USEFUL LINKS FOR COMPLETING THIS FORM
The EMBO Journal - Author Guidelines
EMBO Reports - Author Guidelines
Molecular Systems Biology - Author Guidelines
EMBO Molecular Medicine - Author Guidelines

Reporting Checklist for Life Science Articles (updated January
This checklist is adapted from Materials Design Analysis Reporting (MDAR) Checklist for Authors. MDAR establishes a minimum set of requirements in transparent reporting in the life sciences (see Statement of Task: 10.31222/osf.io/9sm4x). Please follow the journal's guidelines in preparing your
Please note that a copy of this checklist will be published alongside your article.

Abridged guidelines for figures
1. Data
The data shown in figures should satisfy the following conditions:
- ➞ the data were obtained and processed according to the field's best practice and are presented to reflect the results of the experiments in an accurate and unbiased manner.
- ➞ ideally, figure panels should include only measurements that are directly comparable to each other and obtained with the same assay.
- ➞ plots include clearly labeled error bars for independent experiments and sample sizes. Unless justified, error bars should not be shown for technical
- ➞ if n<5, the individual data points from each experiment should be plotted. Any statistical test employed should be justified.
- ➞ Source Data should be included to report the data underlying figures according to the guidelines set out in the authorship guidelines on Data

2. Captions
Each figure caption should contain the following information, for each panel where they are relevant:
- ➞ a specification of the experimental system investigated (eg cell line, species name).
- ➞ the assay(s) and method(s) used to carry out the reported observations and measurements.
- ➞ an explicit mention of the biological and chemical entity(ies) that are being measured.
- ➞ an explicit mention of the biological and chemical entity(ies) that are altered/varied/perturbed in a controlled manner.
- ➞ the exact sample size (n) for each experimental group/condition, given as a number, not a range;
- ➞ a description of the sample collection allowing the reader to understand whether the samples represent technical or biological replicates (including how many animals, litters, cultures, etc.).
- ➞ a statement of how many times the experiment shown was independently replicated in the laboratory.
- ➞ definitions of statistical methods and measures:
  - common tests, such as t-test (please specify whether paired vs. unpaired), simple χ2 tests, Wilcoxon and Mann-Whitney tests, can be unambiguously identified by name only, but more complex techniques should be described in the methods section;
  - are tests one-sided or two-sided?
  - are there adjustments for multiple comparisons?
  - exact statistical test results, e.g., P values = x but not P values < x;
  - definition of 'center values' as median or average;
  - definition of error bars as s.d. or s.e.m.

Please complete ALL of the questions below.
Select "Not Applicable" only when the requested information is not relevant for your study.

Materials

Newly Created Materials	Information included in the manuscript?	In which section is the information available? (Reagents and Tools Table, Materials and Methods, Figures, Data Availability Section)
New materials and reagents need to be available; do any restrictions apply?	Not Applicable	Material and Methods

Antibodies	Information included in the manuscript?	In which section is the information available? (Reagents and Tools Table, Materials and Methods, Figures, Data Availability Section)
For **antibodies** provide the following information:<br>- Commercial antibodies: RRID (if possible) or supplier name, catalogue number and or/clone number<br>- Non-commercial: RRID or citation	Yes	Material and Methods (Supplementary Table 7)

DNA and RNA sequences	Information included in the manuscript?	In which section is the information available? (Reagents and Tools Table, Materials and Methods, Figures, Data Availability Section)
**Short novel DNA or RNA including primers, probes**: provide the sequences.	Yes	Material and Methods (Supplementary Table 6)

Cell materials	Information included in the manuscript?	In which section is the information available? (Reagents and Tools Table, Materials and Methods, Figures, Data Availability Section)
**Cell lines:** Provide species information, strain. Provide accession number in repository **OR** supplier name, catalog number, clone number, and/**OR** RRID.	Yes	Material and Methods
**Primary cultures:** Provide species, strain, sex of origin, genetic modification status.	Not Applicable	Material and Methods
Report if the cell lines were recently **authenticated** (e.g., by STR profiling) and tested for mycoplasma contamination.	Not Applicable	

Experimental animals	Information included in the manuscript?	In which section is the information available? (Reagents and Tools Table, Materials and Methods, Figures, Data Availability Section)
**Laboratory animals or Model organisms:** Provide species, strain, sex, age, genetic modification status. Provide accession number in repository **OR** supplier name, catalog number, clone number, **OR** RRID.	Yes	Material and Methods
**Animal observed in or captured from the field:** Provide species, sex, and age where possible.	Not Applicable	
Please detail **housing and husbandry conditions**.	Yes	Material and Methods

Plants and microbes	Information included in the manuscript?	In which section is the information available? (Reagents and Tools Table, Materials and Methods, Figures, Data Availability Section)
**Plants:** provide species and strain, ecotype and cultivar where relevant, unique accession number if available, and source (including location for collected wild specimens).	Not Applicable	
**Microbes:** provide species and strain, unique accession number if available, and source.	Not Applicable	

Human research participants	Information included in the manuscript?	In which section is the information available? (Reagents and Tools Table, Materials and Methods, Figures, Data Availability Section)
If collected and within the bounds of privacy constraints report on age, sex and gender or ethnicity for all study participants.	Not Applicable	

Core facilities	Information included in the manuscript?	In which section is the information available? (Reagents and Tools Table, Materials and Methods, Figures, Data Availability Section)
If your work benefited from core facilities, was their service mentioned in the acknowledgments section?	Yes	Material and Methods

Design

Study protocol	Information included in the manuscript?	In which section is the information available? (Reagents and Tools Table, Materials and Methods, Figures, Data Availability Section)
If study protocol has been **pre-registered, provide DOI in the manuscript**. For clinical trials, provide the trial registration number **OR** cite DOI.	Not Applicable	
Report the **clinical trial registration number** (at ClinicalTrials.gov or equivalent), where applicable.	Not Applicable	

Laboratory protocol	Information included in the manuscript?	In which section is the information available? (Reagents and Tools Table, Materials and Methods, Figures, Data Availability Section)
Provide DOI OR other citation details if **external detailed step-by-step protocols** are available.	Not Applicable	

Experimental study design and statistics	Information included in the manuscript?	In which section is the information available? (Reagents and Tools Table, Materials and Methods, Figures, Data Availability Section)
Include a statement about **sample size** estimate even if no statistical methods were used.	Yes	Material and Methods, Figures, Data Availability Section
Were any steps taken to minimize the effects of subjective bias when allocating animals/samples to treatment (e.g. **randomization procedure**)? If yes, have they been described?	Not Applicable	
Include a statement about **blinding** even if no blinding was done.	Not Applicable	
Describe **inclusion/exclusion criteria** if samples or animals were excluded from the analysis. Were the criteria pre-established?<br><br>If sample or data points were omitted from analysis, report if this was due to attrition or intentional exclusion and provide justification.	Not Applicable	
For every figure, are **statistical tests** justified as appropriate? Do the data meet the assumptions of the tests (e.g., normal distribution)? Describe any methods used to assess it. Is there an estimate of variation within each group of data? Is the variance similar between the groups that are being statistically compared?	Yes	Material and Methods, Figures, Data Availability Section

Sample definition and in-laboratory replication	Information included in the manuscript?	In which section is the information available? (Reagents and Tools Table, Materials and Methods, Figures, Data Availability Section)
In the figure legends: state number of times the experiment was **replicated** in laboratory.	Yes	Material and Methods, Figure legends
In the figure legends: define whether data describe **technical or biological replicates**.	Yes	Material and Methods, Figure legends

Ethics

Ethics	Information included in the manuscript?	In which section is the information available? (Reagents and Tools Table, Materials and Methods, Figures, Data Availability Section)
Studies involving **human participants**: State details of **authority granting ethics approval** (IRB or equivalent committee(s), provide reference number for approval.	Not Applicable	
Studies involving **human participants**: Include a statement confirming that **informed consent** was obtained from all subjects and that the experiments conformed to the principles set out in the WMA Declaration of Helsinki and the Department of Health and Human Services Belmont Report.	Not Applicable	
Studies involving **human participants:** For publication of **patient photos**, include a statement confirming that consent to publish was obtained.	Not Applicable	
Studies involving experimental **animals**: State details of **authority granting ethics approval** (IRB or equivalent committee(s), provide reference number for approval. Include a statement of compliance with ethical regulations.	Yes	Material and Methods
Studies involving **specimen and field samples:** State if relevant **permits** obtained, provide details of authority approving study; if none were required, explain why.	Not Applicable	

Dual Use Research of Concern (DURC)	Information included in the manuscript?	In which section is the information available? (Reagents and Tools Table, Materials and Methods, Figures, Data Availability Section)
Could your study fall under dual use research restrictions? Please check biosecurity documents and list of **select agents and toxins** (CDC): https://www.selectagents.gov/sat/list.htm	Not Applicable	
If you used a select agent, is the security level of the lab appropriate and reported in the manuscript?	Not Applicable	
If a study is subject to dual use research of concern regulations, is the name of the **authority granting approval and reference number** for the regulatory approval provided in the manuscript?	Not Applicable	

Reporting

The MDAR framework recommends adoption of discipline-specific guidelines, established and endorsed through community initiatives. Journals have their own policy about requiring specific guidelines and recommendations to complement MDAR.

Adherence to community standards	Information included in the manuscript?	In which section is the information available? (Reagents and Tools Table, Materials and Methods, Figures, Data Availability Section)
State if relevant guidelines or checklists (e.g., **ICMJE, MIBBI, ARRIVE, PRISMA**) have been followed or provided.	Not Applicable	
For **tumor marker prognostic studies,** we recommend that you follow the **REMARK** reporting guidelines (see link list at top right). See author guidelines, under 'Reporting Guidelines'. Please confirm you have followed these guidelines.	Not Applicable	
For **phase II and III randomized controlled trials**, please refer to the **CONSORT** flow diagram (see link list at top right) and submit the CONSORT checklist (see link list at top right) with your submission. See author guidelines, under 'Reporting Guidelines'. Please confirm you have submitted this list.	Not Applicable	

Data Availability

Data availability	Information included in the manuscript?	In which section is the information available? (Reagents and Tools Table, Materials and Methods, Figures, Data Availability Section)
Have **primary datasets** been deposited according to the journal's guidelines (see 'Data Deposition' section) and the respective accession numbers provided in the Data Availability Section?	Yes	Data Availability
Were **human clinical and genomic datasets** deposited in a public access-controlled repository in accordance to ethical obligations to the patients and to the applicable consent agreement?	Not Applicable	
Are **computational models** that are central and integral to a study available without restrictions in a machine-readable form? Were the relevant accession numbers or links provided?	Not Applicable	
If publicly available data were reused, provide the respective **data citations in the reference list**.	Not Applicable	
